# Metabolomics analyses of traditional Chinese medicine formula *Shuang Huang Lian* by UHPLC-QTOF-MS/MS

**DOI:** 10.1186/s13020-022-00610-x

**Published:** 2022-05-30

**Authors:** Gang Xu, Yachun Shu, Yan Xu

**Affiliations:** 1 Department of Chemistry, Cleveland State University, Cleveland, OH 44115 USA; 2 Department of Pharmacy, The First Affiliated Hospital of Nanjing University of Chinese Medicine, Nanjing, 210029 Jiangsu People’s Republic of China

**Keywords:** *Shuang Huang Lian*, Metabolomics analyses, UHPLC-QTOF-MS/MS, Traditional Chinese medicine formula, Upper respiratory tract infections

## Abstract

**Background:**

*Shuang Huang Lian* (SHL) is a traditional Chinese medicine (TCM) formula made from Lonicerae Japonicae Flos, Forsythiae Fructus, and Scutellariae Radix. Despite the widespread use of SHL in clinical practice for treating upper respiratory tract infections (URTIs), the complete component fingerprint and the pharmacologically active components in the SHL formula remain unclear. The objective of this study was to develop an untargeted metabolomics method for component identification, quantitation, pattern recognition, and cross-comparison of various SHL preparation forms (i.e., granule, oral liquid, and tablet).

**Methods:**

Ultra-high-performance liquid chromatography and quadrupole time-of-flight tandem mass spectrometry (UHPLC-QTOF-MS/MS) together with bioinformatics were used for chemical profiling, identification, and quantitation of SHL. Multivariate data analyses such as principal component analysis (PCA) and partial least squares discriminant analysis (PLS-DA) were performed to assess the correlations among the three SHL preparation forms and the reproducibility of the technical and biological replicates.

**Results:**

A UHPLC-QTOF-MS/MS-based untargeted metabolomics method was developed and applied to analyze three SHL preparation forms, consisting of 178 to 216 molecular features. Among the 95 common molecular features from the three SHL preparation forms, quantitative analysis was performed using a single exogenous reference internal standard. Forty-seven of the 95 common molecular features have been identified using various databases. Among the 47 common components, there were 17 flavonoids, 7 oligopeptides, 5 terpenoids, 2 glycosides, 2 cyclohexanecarboxylic acids, 2 spiro compounds, 2 lipids, 2 glycosylglycerol derivatives, and 8 various compounds such as alkyl caffeate ester, aromatic ketone, benzaldehyde, benzodioxole, benzofuran, chalcone, hydroxycoumarin, and purine nucleoside. Five of the 47 common components were designated by the Chinese Pharmacopoeia as the quality markers of medicinal plants of SHL, and 15 were previously reported to have pharmacological activities. Distinct patterns of the three SHL preparation forms were observed in the PCA and PLS-DA plots.

**Conclusions:**

The developed method is reliable and reproducible, which is useful for the profiling, component identification, quantitation, quality assessment of various SHL preparation forms and may apply to the analysis of other TCM formulas.

**Supplementary Information:**

The online version contains supplementary material available at 10.1186/s13020-022-00610-x.

## Background

Traditional Chinese medicine (TCM) has been used to prevent and treat various diseases for over 2500 years. *Shuang Huang Lian* (SHL) is a modern TCM formula that has been widely used in Asian countries as a remedy for fever, cough, sore throat, and upper respiratory tract infections (URTIs) [1–4]. SHL inhibits the respiratory syncytial virus (RSV), para-influenza I–IV, and 23 kinds of pathogenic bacteria such as *Staphylococcus aureus* and *Pseudomonas aeruginosa*, etc. in vitro cell culture studies [5–8]. Moreover, SHL had been recommended by the Chinese Guidelines for Diagnosis and Treatment of Influenza (2011) for the treatment of influenza [9]. Currently, SHL is widely used in clinical practice to treat various respiratory diseases, including acute URTIs [3, 4, 9, 10].

SHL is comprised of the alcohol–water extracts of Lonicerae Japonicae Flos (the dried buds of Lonicera japonica Thunb*.*), Forsythiae Fructus [the dried fruits of Forsythia suspense (Thunb.) Vahl], and Scutellariae Radix (the dried roots of Scutellaria baicalensis Georgi.) with a ratio of 1:2:1 [11–13]. Nowadays, various preparation forms of SHL are made and commercially available, such as granules, tablets, oral liquid, powder for injection, etc. [13]. Although the widespread use of SHL by practitioners of complementary and alternative medicine and its efficacy for treating URTIs, the pharmacologically active components and the molecular mechanisms of SHL remain unclear. Therefore, it is necessary to explore the pharmacologically active components of SHL first, then to uncover the molecular mechanisms in support of evidence-based medicine. In this work, we intend to address the first task.

The analytical methods currently available for SHL, including CE, LC-PDA, LC-ECD, and LC–MS, have mainly targeted analyses for quantitation of a few marker components that may not even be the bioactive components of the herbal medicine formula [1, 11, 14–18]. Although there were a few reports on the determination of multi-components in either SHL powder for injection or oral liquid using high-resolution LC–MS [2, 9, 12, 19, 20], the study of chemical components of SHL is still limited. There is neither a complete component fingerprint of the SHL formula nor a comparative analysis on various SHL preparation forms.

SHL is a mixture of three herbal extracts containing hundreds of compounds, and these compounds can further react with each other to form new compounds. In this work, we have developed an untargeted metabolomics workflow for profiling, component identification, semi-quantitation, pattern recognition, and cross-comparison of various SHL preparation forms (i.e., granule, oral liquid, and tablet), which is based on the uses of ultra-high-performance liquid chromatography and quadrupole time-of-flight tandem mass spectrometry (UHPLC-QTOF-MS/MS) for data acquisition and bioinformatics for data analysis. We have also performed both database search and literature mining to retrieve the antiviral, antibacterial, and other pharmacologically active components of the SHL formula, which can be used for the network pharmacology study to unravel the molecular mechanisms of the SHL formula and discover lead compounds for new therapeutic agents [21, 22].

## Materials and methods

### Chemicals and reagents

Ammonium hydroxide and formic acid were purchased from Sigma-Aldrich (St. Louis, MO, USA). Acetonitrile and methanol (Optima™ LC/MS grade) were purchased from Fisher Scientific (Bridgewater, NJ, USA). Deionized water was obtained from an in-house Barnstead Nanopure® water purification system (Thermo Scientific, Waltham, MA, USA) with a resistivity meter reading of 18.2 MΩ-cm. Etoposide-d3 used as the internal standard (IS) was purchased from Toronto Research Chemicals (Toronto, Ontario, Canada).

*Shuang Huang Lian* tablets (Batch number: 1406003) were purchased from Harbin Sanctity Biological Pharmaceutical (Harbin, Heilongjiang, China). *Shuang Huang Lian* granule (Batch number: 151230) was purchased from Harbin Children Pharmaceutical Factory (Harbin, Heilongjiang, China). *Shuang Huang Lian* oral liquid (Batch number: 15065022) was purchased from Henan Fusen Pharmaceutical (Nanyang, Henan, China).

### Preparation of internal standard, SHL, and QC samples

The stock solution of etoposide-d3 (IS) was prepared by dissolving 1.00 mg powder in 1.00 mL of methanol to a 1.00 mg/mL concentration. The working solution of IS was prepared by a 1/10 dilution of the stock solution in methanol to a concentration of 0.100 mg/mL (169 µM).

Two *Shuang Huang Lian* tablets (0.530 g/tablet), one package of *Shuang Huang Lian* granules (5.00 g/package), and 10.0 mL *Shuang Huang Lian* oral liquid lyophilized using a Freezone 4.5 L Freeze Dry System (Labconco, Kansas City, MO, USA), which were all equivalent to 15.0 g raw herbal pieces according to the manufacturers’ instructions, were transferred to three identical 50.0 mL volumetric flasks (SIBATA Scientific Technology, Kaohsiung, Taiwan), then, 20.0 mL deionized water was added to soak for 60 min. After soaking, 20.0 mL deionized water was added. After being mixed by swirling, the solution was sonicated for 30.0 min using an FS30 Ultrasonic Cleaner (Fisher Scientific, Pittsburg, PA, USA) at 55 °C. Deionized water was added to the mark of the flask and mixed by inverting after the solution cooled down to room temperature. The solution in each flask was allowed to settle for 30.0 min before use. Then, 3.00 mL supernatant was transferred to a borosilicate glass test tube (16 × 100 mm) (Fisher Scientific, Hanover Park, IL, USA) followed by the addition of 6.90 mL methanol and 0.100 mL IS working solution. After vortexing for 30 s using a MaxiMix I Vortex Mixer (Thermo Scientific, Waltham, MA, USA), 1.00 mL of solution was transferred to a 1.50 mL microcentrifuge tube (VWR, Radnor, PA, USA), which was centrifuged at 18,000×*g* for 10 min at 4 °C using a Sorvall ST 40R centrifuge (Thermo Scientific, Waltham, MA, USA). The supernatant (600 µL) was then transferred to a 1.80-mL LC glass vial (ThermoFisher Scientific, West Palm Beach, FL, USA) and subjected to the UHPLC-MS/MS analysis.

QC samples (600 µL) could be prepared by mixing 200 µL of each of the three SHL sample solutions and used with each batch analysis by monitoring the selectivity and reproducibility of the 47 commonly identified compounds throughout the analysis.

### Assessment of sample matrix effects

The matrix effects were assessed in terms of absolute matrix factors (MFs) for each SHL preparation form at both positive and negative ionization mode by spiking the IS into the sample solution. The MFs of the IS were determined by the mean peak area of the IS spiked at a fixed concentration (1.69 µM) in an extracted sample matrix over that of the IS spiked at the concentration in a blank solution (70% methanol) in each ionization mode.

### Method validation

The selectivity and reproducibility of the UHPLC-QTOF-MS/MS method were assessed by replicate measurements of three SHL preparation forms. PCA and PLS-DA score plots were constructed to visualize the closeness of the replicate measurements of each SHL preparation form and the differences among the three SHL preparation forms. The intra-day coefficient variation (CV) was determined by the concentrations of triplicate measurements of the 47 commonly identified compounds in the same sample within the same day, whereas the inter-day CV was determined by the concentrations of three parallel measurements of the 47 commonly identified compounds in three identical samples in 3 separate days.

### UHPLC-QTOF-MS/MS system

The UHPLC-QTOF-MS/MS system used in this work consisted of Agilent 1290 Infinity UHPLC modules (Agilent Corp., Santa Clara, CA, USA) coupled with Agilent 6540 QTOF Mass Spectrometer (Agilent Corp., Santa Clara, CA, USA). The UHPLC modules included a solvent reservoir, a degasser, a G4220A binary pump, a G1330B thermostat, a G4226A autosampler, a G1316C thermostatted column compartment, and a G4212A diode-array detector. The mass spectrometer was equipped with an Agilent Jet Stream electrospray ionization (AJS-ESI) probe. The UHPLC column outlet was connected to the mass spectrometer using polyether ether ketone (PEEK) tubing (0.0625 in. o.d. × 0.00500 in. i.d.).

Liquid chromatographic separation was achieved using gradient elution on a Waters ACQUITY UPLC® BEH C18 (2.1 mm i.d. × 100 mm, 1.7 µm, 130 Å) column (Waters, Milford, MA, USA) with an inline VHP filter (0.5 µm, stainless steel) from Upchurch Scientific (Oak Harbor, WA, USA). This column had a pressure tolerance of 18,000 psi, a pH range of 1–12, and a temperature range of 20–90 °C. The mobile phase used for the positive electrospray ionization (ESI^+^) mode acquisition was composed of (A) 0.1% formic acid aqueous solution and (B) 0.1% formic acid in acetonitrile. The mobile phase used for the negative electrospray ionization (ESI^−^) mode acquisition was composed of (A) 0.1% ammonium hydroxide aqueous solution and (B) 0.1% ammonium hydroxide in acetonitrile. The gradient elution profile was as follows: 0–4 min, 5% B; 4–7 min, 5–10% B; 7–20 min, 10–15% B; 20–30 min, 15–22% B; 30–35 min, 22–35% B; 35–40 min, 35–50% B; 40–45 min, 50–70% B; 45–50 min, 70–90% B; 50–52 min, 5% B; 52–60 min, 5% B. The flow rate was at 0.200 mL/min. The column temperature was at 60 °C. The sample injection volume was 5.00 μL. Before sample analysis, the column was equilibrated with a mobile phase at the initial gradient for 1 h at a flow rate of 0.200 mL/min.

The Agilent 6540 QTOF Mass Spectrometer was operated at both positive and negative ESI modes. The LC–MS/MS data were acquired using Agilent MassHunter Data Acquisition software (Version: B.05.01) with auto MS/MS acquisition mode. The operation conditions of the AJS-ESI source were as follows: drying gas (N_2_) temperature, 350 °C; drying gas flow rate, 10.0 L/min; nebulizer gas (N_2_) pressure, 35 psi; sheath gas (N_2_) temperature, 325 °C; sheath gas flow rate, 11.0 L/min; capillary voltage, 4000 V; nozzle voltage, 500 V; fragmentor voltage, 100 V; skimmer voltage, 65 V; octopole radio-frequency voltage (OCT RF V), 750 V. The collision energies (CE) were set at 10, 20, and 40 eV. The MS scan range was from 50 to 1800 m/z with a scan rate of 5 spectra/s. The MS/MS scan range was from 50 to 1800 m/z with a scan rate of 4 spectra/s. To maintain the mass accuracy, the mass spectrometer was tuned using the Agilent tuning mix solution before analysis, and the reference mass solution was used for real-time mass correction and validation at m/z 121.0509 and m/z 922.0098 for the positive ion mode, and m/z 112.9856 and m/z 1033.9881 for the negative ionization mode, throughout the data acquisition process (Additional file 1: Appendix S1).

### Data processing and component identification

Data acquired from the samples of three SHL preparation forms at either positive or negative ionization mode by Agilent MassHunter Data Acquisition software were saved as (.d) files; then evaluated with Agilent MassHunter Qualitative Analysis software (Version: B.06.00) for peak shape, signal to noise ratio, retention time and mass shifts (vs. the spiked IS). The (.d) files were further processed by Agilent MassHunter Profinder software (Version: B.06.00) for batch recursive analysis. The data files were grouped by positive and negative ion modes in three preparation forms. The molecular features were extracted with a peak height threshold of 1,000 counts, possible ion adducts [M+H]^+^, [M+Na]^+^, [M+NH_4_]^+^ for positive ion mode and [M−H]^−^ for negative ion mode, isotope model of common organic molecules, charge state up to two, a retention time window of 0.10% + 0.60 min, and a mass window of 20.00 ppm + 2.00 mDa for the alignment of the IS in each data group with the same polarity. The post-processing filter was set at 3 out of 3 replicate measurements for each SHL preparation form at the same polarity. The molecular feature extraction and find-by-ion data files using the Agilent MassHunter Profinder software were exported as compound exchange files (.cefs).

Each (.cef) file exported from the Agilent MassHunter Profinder software and its corresponding (.d) file were imported to the Agilent MassHunter Qualitative Analysis software to extract MS/MS data along with its MS data using the “Find by Formula” function under “Method Explorer”. The extracted data file for each sample run was then exported as a new (.cef) file for further data processing. All new (.cef) files of replicates measurements of each SHL preparation form at the same polarity exported from Agilent MassHunter Qualitative Analysis software were imported to Agilent Mass Profiler Professional (MPP) software (Version: B.13.1.1) for molecular formula generation and compound identification using the “ID Browser” function to search the Agilent METLIN AM database. To generate molecular formulas with the extracted molecular features, the selection and cut-off limit of elements were as follows: carbon (3–156); hydrogen (0–180); oxygen (0–40); nitrogen (0–20); sulfur (0–14); chlorine (0–12); fluorine (0–48); bromine (0–10); phosphorus (0–9); and silicon (0–15) [23]. The top 5 identified compounds with the highest scores for each molecular formula were cross-checked with the Traditional Chinese Medicine Integrated Database (TCMID) [24] and the Traditional Chinese Medicine Systems Pharmacology (TCMSP) Database and Analysis Platform before the final annotation [25, 26]. For the analysis of fragmentation pathways of MS/MS spectra, Agilent MassHunter Molecular Structure Correlator (MSC) (Version: 8.1) was first used to correlate the accurate mass MS/MS fragment ions for precursor ions in forms of proton adducts, and the unresolved fragmentation patterns were analyzed by an open-source software SIRIUS + CSI:FingerID GUI (Version 4.9.12) [27].

### Statistical analysis and pattern recognition

Principal component analysis (PCA) and partial least squares discriminant analysis (PLS-DA) were performed on the MetaboAnalyst 4.0 online platform [28, 29]. In detail, the (.csv) files of replicate measurements of each SHL preparation form at the same polarity were exported from the Agilent MassHunter Profinder, which carried the data of mass, retention time, and peak area. The (.csv) files of MS peak list data were then combined as one (.zip) file and uploaded to the MetaboAnalyst platform. A mass tolerance of 0.025 Da and a retention time tolerance of 30.0 s were chosen for compound alignment. The data were filtered with the “Interquartile Range (IQR)” model to identify and remove variables from baseline noises and improve the accuracy of the results. Data normalization was performed using the IS reference feature (i.e., mass, retention time, and peak area). All data were log-transformed and auto-scaled. The 2D PCA and PLS-DA score plots were constructed. For PLS-DA, the variable importance in projection (VIP) scores were calculated as a weighted sum of the squared correlation between the PLS-DA components and the original variable, summarizing each variable's contribution and influence to this model [30, 31].

### Global semiquantitative analysis

Global semiquantitative analysis was carried out using the (.d) files with the same polarity of the replicate measurements of each SHL preparation form obtained by the Agilent MassHunter Acquisition software and the corresponding combined data files (.cef) with the identities obtained by the Agilent MPP software. The (.d) and (.cef) files were imported into Agilent MassHunter Quantitative Analysis software (Version: B.06.00). The retention time window was set at 0.6 min in the method setup task. The m/z of IS adducts, [IS+NH_4_]^+^ and [M−H]^−^, were chosen for the positive and negative ionization modes and flagged. Other chemical components were set as targets relative to the IS, and the ionization polarities were identified. After validating the method setup, global semiquantitative analysis was performed based on the peak area ratio of each target to the IS. The results were exported as an Excel file for reporting.

## Results and discussion

### Optimization of the UHPLC-QTOF-MS/MS method

The choices of mobile-phase pair for gradient elution and column for separation were investigated. The data (not shown) indicated that the acetonitrile–water pair had lower back pressure and gave better analyte resolution than those of the methanol–water pair on C18 columns. Therefore, the acetonitrile–water pair was selected as the mobile-phase pair for the method. In addition, 0.1% formic acid or 0.1% ammonium hydroxide was added to the mobile phase pair to facilitate the protonation or deprotonation of the analytes for mass spectrometric detection of the analytes in positive or negative ionization mode. It was also found that the Waters ACQUITY UPLC® BEH C18 column (2.1 mm i.d. × 100 mm, 1.7 µM, 130 Å) gave greater separation efficiency, larger signal-to-noise ratio, and better peak shape than those of the Agilent ZORBAX Extend-C18 Rapid Resolution HT column (2.1 mm i.d. × 50 mm, 1.8 µM, 80 Å); therefore, the former was adopted for the method.

Both positive and negative ionization modes were applied to the analyses of SHL samples using QTOF-MS/MS, and comprehensive information about the SHL components was obtained. The fragmentor voltage that plays a vital role in generating fragments in the auto MS/MS acquisition mode was examined using three voltage settings of 100 V, 120 V, and 150 V. The voltage of 100 V that generated fragments matched the literature reports [9] and therefore adopted for the method. The collision energy was set at 10, 20, and 40 eV to correspond with those of the MS and MS/MS spectra in the METLIN AM database.

### Internal standard and matrix effects of various SHL preparation forms

An exogenous stable isotope-labeled compound, etoposide-d3, was chosen as the IS for multiple purposes in this work, including corrections of retention time and mass shifts in the analysis of mass chromatographic data, assessment of sample matrix effect, peak normalization in multivariate data analysis, global semi-quantitative analysis, and cross-comparison of the common multi-components in various SHL preparation forms. Etoposide is a synthetic compound, and its stable isotope etoposide-d3 does not occur as an endogenous compound in plant products. The use of etoposide-d3 as the IS eliminated the potential interference from endogenous compounds in sample matrices.

Our experimental data (not shown) indicated no chromatographic and mass spectrometric interferences on the IS detection from the solution blanks and the samples of the three SHL preparation forms. The matrix effects of the SHL samples on the mass spectrometric detection of the IS were quantified by MFs. As shown in Table 1, the MFs were 0.91–0.93, 0.95–0.97, and 0.89–0.93, respectively, for the SHL granule, oral liquid, and tablet preparation forms by mass spectrometric detections in both positive and negative ionization modes. These MF values were close to 1.0, indicating no significant signal suppression on the detection of the IS by the sample matrices.

**Table 1:** Matrix effects of SHL samples on mass spectrometric detection of the IS

SHL sample	ESI mode	PA_IS_^a^ in extracted sample matrix ± SD^b^	PA_IS_ in solution ± SD	MF_IS_^c^ ± SD
Granules	+	(6.95 ± 0.05) × 10^5^	(7.6 ± 0.2) × 10^5^	0.91 ± 0.02
	−	(2.05 ± 0.08) × 10^6^	(2.2 ± 0.1) × 10^6^	0.93 ± 0.06
Oral liquid	+	(7.4 ± 0.2) × 10^5^	(7.6 ± 0.2) × 10^5^	0.97 ± 0.04
	−	(2.09 ± 0.02) × 10^6^	(2.2 ± 0.1) × 10^6^	0.95 ± 0.04
Tablet	+	(7.1 ± 0.1) × 10^5^	(7.6 ± 0.2) × 10^5^	0.93 ± 0.03
	−	(1.96 ± 0.05) × 10^6^	(2.2 ± 0.1) × 10^6^	0.89 ± 0.05

[IS] = 1.69 µM

^a^PA_IS_ = mean peak area of the spiked IS

^b^SD = standard deviation

^c^MF_IS_ = (PA_IS_ in the extracted sample matrix)**/**(PA_IS_ in the solution)

### Untargeted and targeted metabolomics analyses of SHL formula

**Fig. 1:**
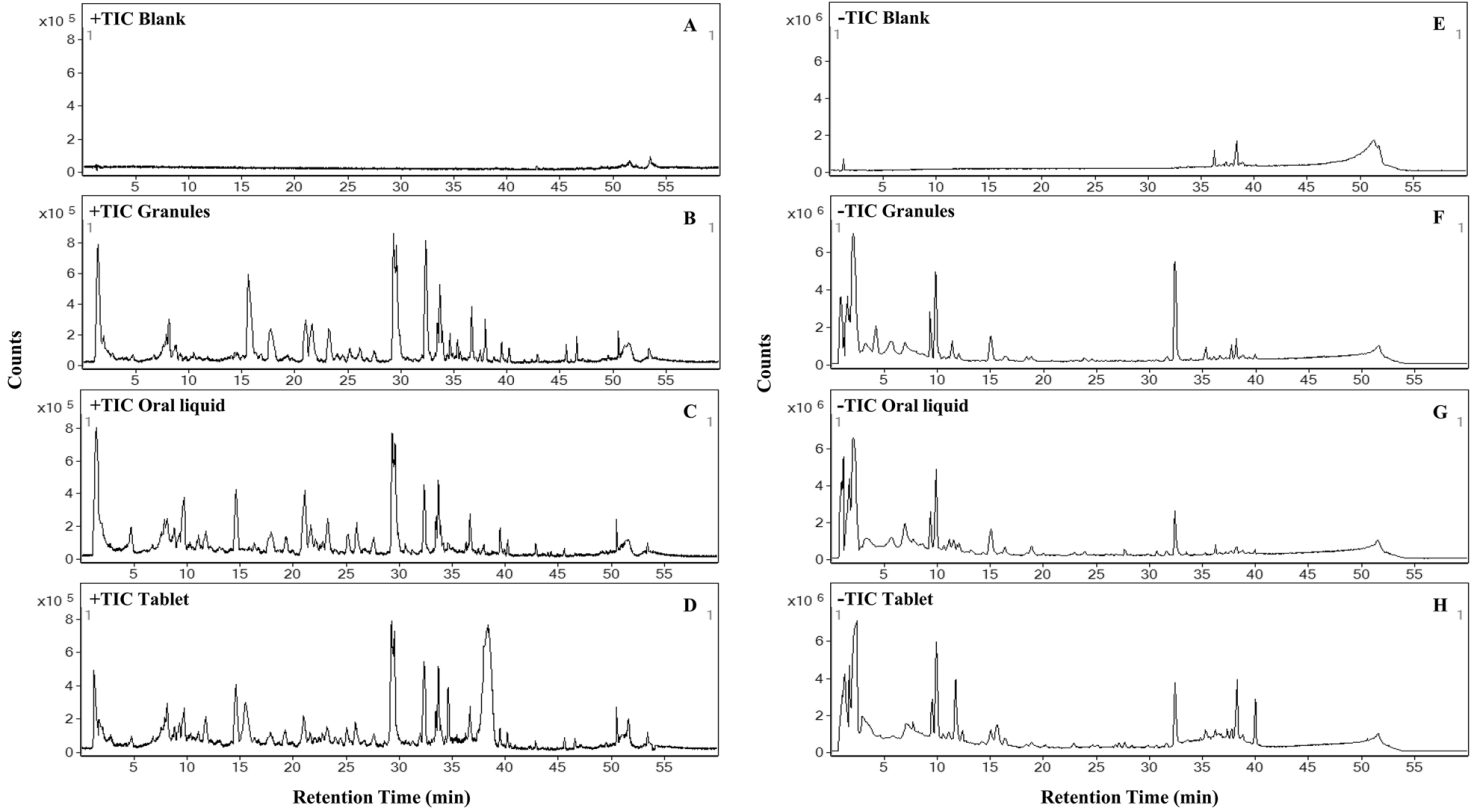
The representative total-ion chromatograms (TICs) of the solution blank and the samples of the three SHL preparation forms by both positive ionization mode (**A**–**D**) and negative ionization mode (**E**–**H**)

For untargeted metabolomics analysis of various SHL preparation forms, triplicate samples were prepared for each SHL preparation form (i.e., granule, oral liquid, and tablet) and the solution blank (i.e., 70% methanol). A total of twelve samples were analyzed using the UHPLC-QTOF-MS/MS method. The mass chromatograms with MS and MS/MS data were acquired from the twelve samples by both positive and negative ESI modes. The representative total-ion-current (TIC) chromatograms were shown in Fig. 1. Using the chromatographic and mass spectrometric data obtained from the untargeted metabolomics profiling, we achieved component identification, global semi-quantitative analysis, and cross-comparison of common components among various SHL preparation forms, as well as multivariate analysis.

**Fig. 2:**
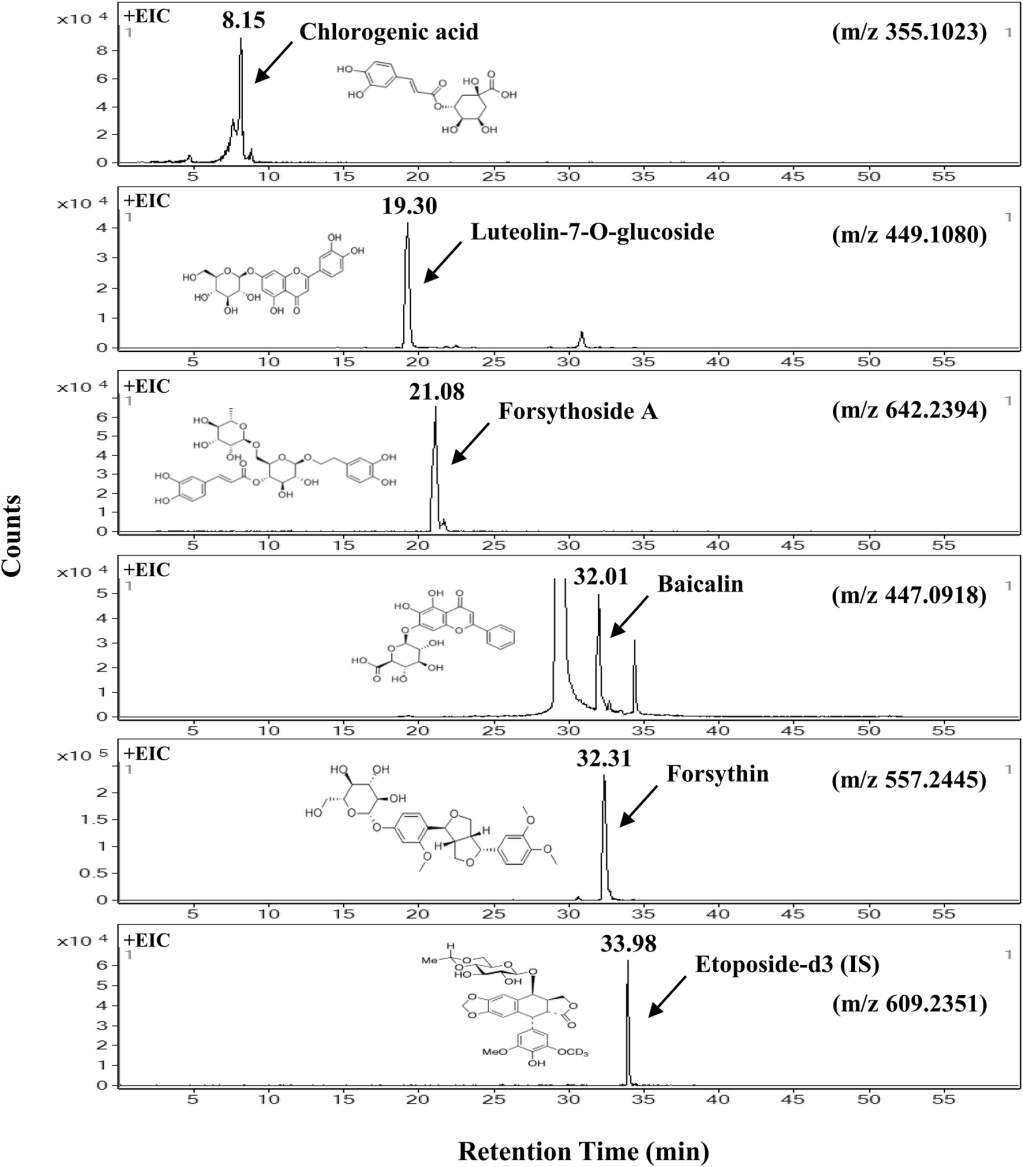
The representative extracted-ion chromatograms (EICs) of five Q markers of SHL formula and the IS at a concentration of 1.69 µM

Targeted metabolomics analysis of the SHL formula was illustrated by the extracted ion chromatograms (EICs) (Fig. 2). As per the Chinese Pharmacopoeia [13], there are five non-volatile, water-soluble quality markers (Q-markers) in the herbs of SHL formula (i.e., chlorogenic acid, luteolin-7-*O*-glucoside, forsythoside A, baicalin, and forsythin). As shown in Fig. 2, these Q-markers could be easily targeted and extracted simultaneously by the UHPLC-MS/MS method developed. They can be used for quality assessment and detection of counterfeited SHL products.

### Identification of components in three SHL preparation forms

**Fig. 3:**
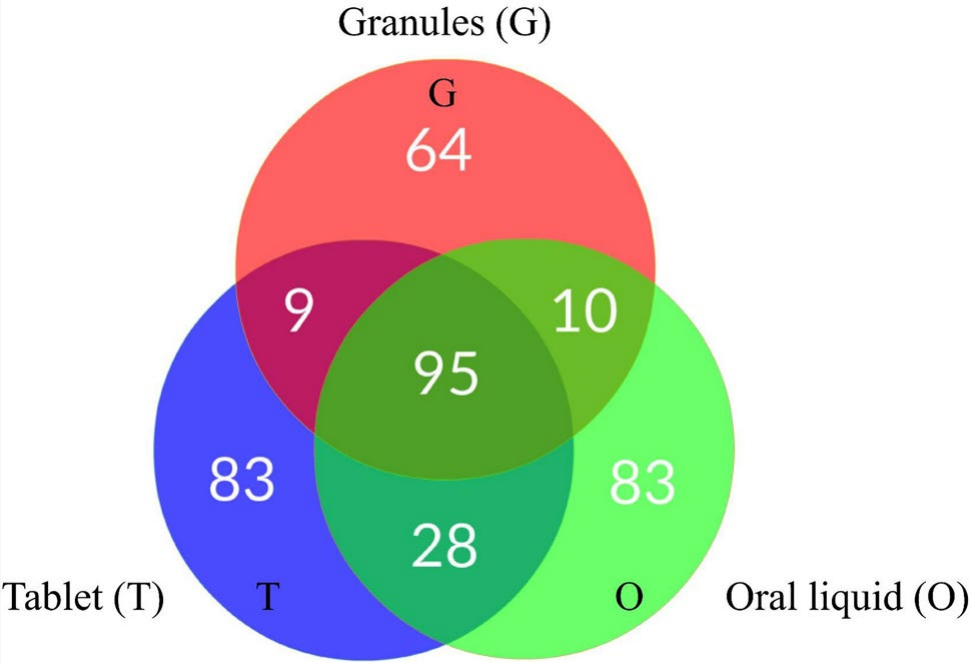
The Venn diagram of the components found in each SHL preparation form

Identification of chemical components in each SHL preparation form was performed per the procedures described in “Materials and methods” section. The list of components in each SHL preparation form was obtained after subtracting the background components in the solution blanks (Additional file 4: Table S1, Additional file 5: Table S2, Additional file 6: Table S3, Additional file 7: Table S4, Additional file 8: Table S5 and Additional file 9: Table S6). The numbers of components identified with both chemical names and formulas and the components unidentified but with formulas in each SHL preparation form were given in Additional file 10: Table S7. As seen in Additional file 10: Table S7, the total chemical components found in three SHL preparation forms were 178, 216, and 215 for granule, oral liquid, and tablet, respectively. Among the 95 components commonly found in the three preparation forms (Fig. 3), 47 of them were identified with both chemical names and formulas (Table 2), and the other 48 were unidentified (or identified only with formulas) (Additional file 11: Table S8). Among the 47 common components, there were 17 flavonoids, 7 oligopeptides, 5 terpenoids, 2 glycosides, 2 cyclohexanecarboxylic acids, 2 spiro compounds, 2 lipids, 2 glycosylglycerol derivatives, and 8 various compounds such as alkyl caffeate ester, aromatic ketone, benzaldehyde, benzodioxole, benzofuran, chalcone, hydroxycoumarin, and purine nucleoside. The mass spectra of the 47 commonly identified components were shown in Additional file 2: Fig. S1. The fragmentation pathways of the commonly identified compounds (Additional file 3: Fig. S2) were proposed using Agilent MSC software via a systematic bond-breaking approach [32] which was applied to most of the precursor ions as proton adducts, and the unresolved fragmentation patterns were analyzed using SIRIUS + CSI:FingerID GUI by the combined analysis of isotope patterns in MS spectra and fragmentation patterns in MS/MS spectra together with the web search in molecular structure databases on CSI:FingerID [33, 34].

**Table 2:** The common chemical components identified with names and formulas in all three SHL preparation forms

No	Formula	Name	t_R_ (min)	Observed mass	Database mass	Precursor ion, m/z	MS/MS quantifier, m/z	MS/MS qualifier, m/z
1	C_21_H_20_O_13_	Tagetiin	1.71	480.0895	480.0916	479.0815, [M−H]^−^	315.0344	139.0030
2	C_28_H_16_O_5_	Naphthofluorescein	4.61	432.1022	432.1031	431.0940, [M−H]^−^	268.0365	239.0332
3	C_21_H_26_N_4_O_8_	Trp-Glu-Glu	7.88	462.1731	462.1751	485.1633, [M+Na]^+^	339.1063	213.0327
4	C_9_H_6_O_3_	Umbelliferone	8.15	162.0316	162.0316	163.0388, [M+H]^+^	63.0232	89.0393
5	C_16_H_18_O_9_	Chlorogenic acid^a^	8.15	354.0950	354.0951	355.1023, [M+H]^+^	89.0391	163.0388
6	C_10_H_10_O_4_	Methyl caffeate	9.75	194.0579	194.0582	195.0652, [M+H]^+^	77.0387	95.0491
7	C_10_H_12_O_5_	Danielone	9.75	212.0683	212.0682	213.0755, [M+H]^+^	107.0491	151.0391
8	C_8_H_6_O_3_	Piperonal	9.76	150.0318	150.0319	151.0391, [M+H]^+^	51.0228	77.0383
9	C_16_H_22_O_10_	Geniposidic acid	9.76	374.1211	374.1213	397.1106, [M+Na]^+^	235.0573	255.0855
10	C_16_H_18_O_8_	*p*-Coumaroyl quinic acid	10.48	338.1003	338.1002	339.1078, [M+H]^+^	91.0544	147.0435
11	C_16_H_22_O_9_	Tarennoside	11.72	358.1264	358.1264	359.1337, [M+H]^+^	197.0811	127.0390
12	C_15_H_26_N_6_O_6_	Asp-Arg-Pro	11.72	386.1912	386.1916	385.1831, [M−H]^−^	153.0919	59.0145
13	C_16_H_28_N_6_O_8_	Arg-Glu-Glu	11.72	432.1969	432.1960	431.1894, [M−H]^−^	269.0449	387.0756
14	C_10_H_12_O_4_	Paeonilactone B	11.78	196.0736	196.0732	197.0808, [M+H]^+^	127.0386	53.0386
15	C_20_H_27_N_5_O_6_	Thr-Gln-Trp	15.15	433.1945	433.1943	434.2017, [M+H]^+^	85.0283	145.0490
16	C_20_H_24_N_4_O_6_	Pro-Trp-Asp	15.15	416.1680	416.1696	434.2017, [M+NH_4_]^+^	295.1026	285.1343
17	C_15_H_21_N_5_O_8_	Asp-Glu-His	16.34	399.1396	399.1390	417.1734, [M+NH_4_]^+^	285.1301	85.0284
18	C_16_H_18_N_6_O_4_	2-Phenylaminoadenosine	16.50	358.1394	358.1387	357.1315, [M−H]^−^	151.0398	136.0177
19	C_27_H_30_O_16_	Rutin	17.73	610.1536	610.1537	611.1611, [M+H]^+^	303.0497	465.1027
20	C_21_H_18_O_12_	Luteolin 3′-glucuronide	18.13	462.0814	462.0797	463.0872, [M+H]^+^	287.0550	123.0080
21	C_21_H_20_O_11_	Luteolin-7-*O*-glucoside^a^	19.30	448.1007	448.1006	449.1080, [M+H]^+^	287.0548	153.0178
22	C_21_H_26_O_12_	Plumieride	21.08	470.1424	470.1423	471.1499, [M+H]^+^	163.0387	325.0912
23	C_29_H_36_O_15_	Forsythoside A^a^	21.08	624.2046	624.2054	642.2394, [M+NH_4_]^+^	471.1486	163.0385
24	C_13_H_28_N_6_O_8_	Zwittermicin A	22.64	396.1975	396.1979	395.1906, [M−H]^−^	263.1487	101.0251
25	C_20_H_20_O_5_	Morachalcone A	23.24	340.1309	340.1313	341.1384, [M+H]^+^	137.0592	291.1008
26	C_26_H_32_O_11_	Brusatol	23.24	520.1943	520.1945	538.2284, [M+NH_4_]^+^	235.0961	175.0754
27	C_27_H_30_O_14_	Isofurcatain 7-*O*-glucoside	25.01	578.1637	578.1637	579.1708, [M+H]^+^	271.0600	433.1131
28	C_25_H_24_O_12_	Apigenin 7-(3″,4″-diacetylglucoside)	25.96	516.1268	516.1269	517.1342, [M+H]^+^	163.0394	337.0914
29	C_21_H_20_O_10_	Isovitexin	29.87	432.1059	432.1058	433.1135, [M+H]^+^	271.0608	123.0080
30	C_27_H_34_O_11_	Undulatone	30.67	534.2073	534.2065	533.2000, [M−H]^−^	371.1487	356.1261
31	C_21_H_18_O_11_	Baicalin^a^	32.01	446.0846	446.0849	447.0918, [M+H]^+^	271.0602	123.0079
32	C_27_H_34_O_11_	Forsythin^a^	32.31	534.2097	534.2101	552.2445, [M+NH_4_]^+^	355.1527	189.0910
33	C_22_H_20_O_12_	Hispidulin 7-glucuronide	33.08	476.0957	476.0957	477.1030, [M+H]^+^	301.0706	286.0474
34	C_21_H_18_O_10_	Chrysin 7-glucuronide	33.44	430.0901	430.0902	431.0974, [M+H]^+^	255.0660	153.0179
35	C_22_H_20_O_11_	Wogonin 7-glucuronide	33.69	460.1007	460.1008	461.1080, [M+H]^+^	285.0756	270.0522
36	C_21_H_18_O_11_	Apigenin 7-glucuronide	34.37	446.0845	446.0845	447.0918, [M+H]^+^	271.0597	73.0286
37	C_16_H_12_O_6_	Kaempferide	36.46	300.0638	300.0629	301.0710, [M+H]^+^	286.0462	184.0002
38	C_15_H_10_O_5_	Baicalein	36.68	270.0531	270.0530	271.0604, [M+H]^+^	123.0085	68.9975
39	C_21_H_24_O_6_	Kadsurin A	37.82	372.1575	372.1573	390.1916, [M+NH_4_]^+^	137.0600	355.1549
40	C_16_H_12_O_5_	Wogonin	39.51	284.0686	284.0674	285.0758, [M+H]^+^	270.0536	77.0387
41	C_17_H_14_O_6_	5,3′-Dihydroxy-7,4′-dimethoxy-4-phenylcoumarin	39.88	314.0791	314.0792	315.0863, [M+H]^+^	71.0129	285.0407
42	C_19_H_18_O_8_	Skullcapflavone II	40.19	374.1001	374.0999	375.1075, [M+H]^+^	345.0596	197.0086
43	C_15_H_22_O_2_	Eremophilenolide	45.47	234.1622	234.1623	235.1696, [M+H]^+^	57.0704	180.1141
44	C_24_H_50_NO_7_P	PE (19:0/0:0)	46.58	495.3325	495.3329	496.3399, [M+H]^+^	184.0732	104.1073
45	C_19_H_38_O_4_	1-Monopalmitin	50.89	330.2774	330.2769	331.2846, [M+H]^+^	67.0538	57.0694
46	C_51_H_84_O_15_	1,2-Di-(9Z,12Z,15Z-octadecatrienoyl)-3-(galactosyl-alpha-1-6-galactosyl-beta-1)-glycerol	51.06	936.5809	936.5810	954.6148, [M+NH_4_]^+^	614.4875	335.2578
47	C_45_H_74_O_10_	1,2-Di-(9Z,12Z,15Z-octadecatrienoyl)-3-*O*-Beta-d-galactosyl-sn-glycerol	51.30	774.5282	774.5282	792.5616, [M+NH_4_]^+^	614.4787	336.2604

^a^Q markers

A comparison of the components identified in SHL oral liquid done in the current work with the Agilent METLIN AM database and a reported one done with an in-house library [9] showed that there were 216 components detected by the present work (Additional file 6: Table S3 and Additional file 7: Table S4) whereas 170 components seen in the reported one [9]. Between the two-component sets, there were 27 identical formulas, 11 annotated with the same names (i.e., baicalein, baicalin, chlorogenic acid, chrysin 7-glucuronide, forsythin, forsythoside A, luteolin-7-*O*-glucoside, rutin, skullcapflavone II, wogonin, and wogonin 7-glucuronide), and 16 annotated with different names. One possible explanation for the discrepancy between the two-component sets might be the databases (commercial vs. in-house) and the different MS/MS spectra matching criteria used. Nevertheless, the component sets identified in the current and previous work provided valuable information for the quality control and further investigation of the SHL formula. For unequivocal identification of components in the SHL formula, component isolation and comparison with authentic standards by additional analytical work are needed.

### Global semi-quantitative analysis and cross-comparison among the three preparation forms

Global semi-quantitative analysis was performed on the 47 common components identified in the three SHL preparation forms using the UHPLC-QTOF-MS/MS method developed with an exogenous stable isotope-labeled IS (etoposide-d3). The concentrations detected (µM) were back-calculated to the amounts (µg) that were equivalent to 15.0-g raw herbal pieces, and the reproducibilities of the UHPLC-QTOF-MS/MS method were assessed by the coefficient of variation (CV) (Table 3). As shown in Table 3, the amounts of the 47 common components identified in the three SHL preparation forms were obtained, which could be cross-compared among the three preparation forms. If a CV ≤ 15% was adopted, the least acceptable coverages for the 47 common components in three SHL preparation forms were 87% for intra-day assay and 89% for inter-day assay, respectively, which were better than the recommended value**s** (at least 70% at CV ≤ 15%) [35], indicating thtablee good reproducibility of the analytical method. To make this approach practical for accurate quantitative assessment of multi-components in SHL, conversion factors of the detector responses between each analyte and the IS should be calculated.

**Table 3:** Global semi-quantitative analysis of the 47 common components identified in three SHL preparation forms

No	Formula	Name	Intra-day (n = 3)			Inter-day (n = 3)		
			G ± SD (CV%) (µg^b^)	O ± SD (CV%) (µg^b^)	T ± SD (CV%) (µg^b^)	G ± SD (CV%) (µg^b^)	O ± SD (CV%) (µg^b^)	T ± SD (CV%) (µg^b^)
1	C_21_H_20_O_13_	Tagetiin	44 ± 8 (18)	61 ± 3 (5)	101 ± 5 (5)	49 ± 10 (20)	60 ± 3 (5)	103 ± 4 (4)
2	C_28_H_16_O_5_	Naphthofluorescein	27 ± 7 (26)	39 ± 14 (37)	76 ± 4 (6)	41 ± 13 (31)	43 ± 12 (28)	75 ± 3 (5)
3	C_21_H_26_N_4_O_8_	Trp-Glu-Glu	52 ± 4 (8)	53 ± 3 (5)	28 ± 3 (11)	55 ± 7 (12)	57 ± 6 (11)	30 ± 3 (11)
4	C_9_H_6_O_3_	Umbelliferone	31 ± 1 (3)	26 ± 2 (7)	54 ± 3 (6)	31 ± 2 (5)	25.3 ± 0.6 (2)	61 ± 7 (11)
5	C_16_H_18_O_9_	Chlorogenic Acid^a^	207 ± 19 (9)	143 ± 15 (11)	305 ± 31 (10)	200 ± 19 (9)	142 ± 10 (7)	299 ± 43 (15)
6	C_10_H_10_O_4_	Methyl caffeate	2.52 ± 0.09 (3)	42 ± 3 (7)	33 ± 3 (10)	2.55 ± 0.03 (1)	43 ± 3 (7)	32.7 ± 0.6 (2)
7	C_10_H_12_O_5_	Danielone	5.3 ± 0.1 (2)	79 ± 10 (13)	71 ± 3 (4)	5.42 ± 0.08 (1)	79 ± 5 (6)	70 ± 5 (6)
8	C_8_H_6_O_3_	Piperonal	2.05 ± 0.05 (3)	37 ± 4 (12)	32 ± 4 (11)	1.8 ± 0.3 (15)	39 ± 6 (14)	32 ± 3 (9)
9	C_16_H_22_O_10_	Geniposidic acid	8.6 ± 0.2 (2)	127 ± 3 (3)	72 ± 7 (10)	8.3 ± 0.7 (8)	130 ± 5 (4)	70 ± 6 (9)
10	C_16_H_18_O_8_	*p*-Coumaroyl quinic acid	51 ± 7 (14)	4 ± 1 (32)	11 ± 1 (9)	49 ± 6 (12)	4 ± 1 (38)	11 ± 1 (9)
11	C_16_H_22_O_9_	Tarennoside	3.9 ± 0.2 (4)	82.0 ± 0.8 (1)	241.00 ± 0.07 (0.03)	4.0 ± 0.1 (3)	82 ± 1 (1)	239 ± 3 (1)
12	C_15_H_26_N_6_O_6_	Asp-Arg-Pro	40 ± 4 (11)	63 ± 12 (19)	91 ± 7 (8)	51 ± 18 (36)	66 ± 10 (15)	89 ± 6 (7)
13	C_16_H_28_N_6_O_8_	Arg-Glu-Glu	8 ± 1 (14)	11.8 ± 0.7 (6)	1.3 ± 0.1 (9)	7 ± 2 (29)	11.8 ± 0.7 (6)	1.3 ± 0.1 (9)
14	C_10_H_12_O_4_	Paeonilactone B	2.79 ± 0.03 (1)	51.25 ± 0.09 (0.2)	136 ± 6 (4)	2.78 ± 0.03 (1)	51.0 ± 0.4 (1)	137 ± 5 (3)
15	C_20_H_27_N_5_O_6_	Thr-Gln-Trp	43 ± 4 (10)	42 ± 7 (16)	64 ± 2 (3)	46 ± 6 (13)	45 ± 8 (17)	63 ± 3 (5)
16	C_20_H_24_N_4_O_6_	Pro-Trp-Asp	34 ± 4 (12)	33 ± 6 (19)	55 ± 2 (3)	35 ± 3 (8)	32 ± 5 (15)	54 ± 2 (3)
17	C_15_H_21_N_5_O_8_	Asp-Glu-His	25 ± 1 (4)	35 ± 1 (4)	36 ± 5 (15)	27 ± 4 (13)	35 ± 1 (4)	34 ± 5 (16)
18	C_16_H_18_N_6_O_4_	2-Phenylaminoadenosine	110 ± 11 (10)	36 ± 6 (17)	108 ± 2 (2)	110 ± 11 (10)	37 ± 5 (14)	107 ± 2 (1)
19	C_27_H_30_O_16_	Rutin	316.4 ± 0.6 (0.2)	39 ± 4 (9)	55 ± 1 (2)	321 ± 8 (2)	39 ± 2 (6)	55 ± 1 (2)
20	C_21_H_18_O_12_	Luteolin 3′-glucuronide	107 ± 10 (9)	122 ± 6 (5)	111 ± 11 (10)	128 ± 18 (14)	109 ± 15 (14)	131 ± 20 (15)
21	C_21_H_20_O_11_	Luteolin-7-*O*-glucoside^a^	64 ± 4 (6)	178 ± 3 (2)	294 ± 6 (2)	62 ± 2 (3)	178.4 ± 0.8 (0.5)	285 ± 9 (3)
22	C_21_H_26_O_12_	Plumieride	324 ± 17 (5)	462 ± 8 (2)	200 ± 11 (6)	327 ± 7 (2)	471 ± 11 (2)	199 ± 11 (6)
23	C_29_H_36_O_15_	Forsythoside A^a^	290 ± 24 (8)	398 ± 12 (3)	172 ± 2 (1)	285 ± 9 (3)	414 ± 17 (4)	171 ± 1 (0.4)
24	C_13_H_28_N_6_O_8_	Zwittermicin A	5.8 ± 0.3 (5)	28 ± 2 (6)	1.3 ± 0.1 (11)	6 ± 1 (16)	22 ± 5 (20)	1.5 ± 0.2 (16)
25	C_20_H_20_O_5_	Morachalcone A	98.8 ± 0.3 (0.3)	97 ± 9 (10)	66 ± 2 (3)	98.7 ± 0.3 (0.3)	99 ± 7 (8)	64 ± 4 (6)
26	C_26_H_32_O_11_	Brusatol	258 ± 14 (5)	227 ± 10 (4)	164 ± 2 (1)	261 ± 2 (1)	226 ± 5 (2)	164 ± 2 (1)
27	C_27_H_30_O_14_	Isofurcatain 7-*O*-glucoside	2.7 ± 0.3 (10)	85.7 ± 0.4 (0.5)	178 ± 2 (1)	2.7 ± 0.2 (8)	85.8 ± 0.3 (0.4)	175 ± 4 (2)
28	C_25_H_24_O_12_	Apigenin 7-(3″,4″-diacetylglucoside)	14.3 ± 0.1 (1)	92 ± 1 (1)	98 ± 2 (2)	14.3 ± 0.1 (1)	91.4 ± 0.9 (1)	98 ± 1 (1)
29	C_21_H_20_O_10_	Isovitexin	256 ± 17 (6)	162 ± 1 (0.6)	138 ± 7 (5)	257 ± 11 (4)	161 ± 2 (2)	138 ± 5 (3)
30	C_27_H_34_O_11_	Undulatone	23 ± 2 (7)	82 ± 6 (8)	91 ± 25 (28)	23 ± 2 (7)	84 ± 6 (7)	91 ± 25 (27)
31	C_21_H_18_O_11_	Baicalin^a^	62 ± 2 (4)	30 ± 3 (9)	229 ± 17 (8)	56 ± 5 (8)	32 ± 2 (5)	221 ± 9 (4)
32	C_27_H_34_O_11_	Forsythin^a^	1051 ± 90 (9)	463 ± 17 (4)	898 ± 31 (3)	1056 ± 25 (2)	463 ± 11 (2)	895 ± 26 (3)
33	C_22_H_20_O_12_	Hispidulin 7-glucuronide	20 ± 2 (12)	12.17 ± 0.05 (0.4)	59 ± 3 (5)	21 ± 2 (10)	12.16 ± 0.04 (0.4)	58 ± 3 (4)
34	C_21_H_18_O_10_	Chrysin 7-glucuronide	376 ± 25 (7)	306 ± 12 (4)	461 ± 22 (5)	382 ± 20 (5)	308 ± 10 (3)	456 ± 18 (4)
35	C_22_H_20_O_11_	Wogonin 7-glucuronide	888 ± 75 (8)	840 ± 52 (6)	1278 ± 37 (3)	905 ± 61 (7)	852 ± 42 (5)	1268 ± 31 (2)
36	C_21_H_18_O_11_	Apigenin 7-glucuronide	47 ± 2 (4)	21.9 ± 0.8 (3)	86 ± 1 (1)	48 ± 2 (3)	21.9 ± 0.5 (2)	85 ± 2 (2)
37	C_16_H_12_O_6_	Kaempferide	22 ± 1 (4)	34.6 ± 0.1 (0.4)	37.2 ± 0.3 (1)	21.7 ± 0.7 (3)	34.2 ± 0.4 (1)	36 ± 2 (4)
38	C_15_H_10_O_5_	Baicalein	275 ± 14 (5)	212 ± 4 (2)	264 ± 26 (10)	278 ± 6 (2)	213 ± 5 (2)	271 ± 10 (4)
39	C_21_H_24_O_6_	Kadsurin A	50.1 ± 0.7 (1)	20 ± 1 (6)	31 ± 1 (3)	50.2 ± 0.5 (1)	19 ± 1 (5)	31.6 ± 0.8 (2)
40	C_16_H_12_O_5_	Wogonin	119 ± 6 (5)	161 ± 4 (3)	169 ± 1 (1)	118 ± 3 (2)	161 ± 3 (2)	166 ± 3 (2)
41	C_17_H_14_O_6_	5,3′-Dihydroxy-7,4′-dimethoxy-4-phenylcoumarin	12.8 ± 0.7 (5)	22.3 ± 0.8 (4)	28 ± 1 (5)	12 ± 1 (8)	21 ± 2 (7)	27 ± 3 (10)
42	C_19_H_18_O_8_	Skullcapflavone II	27 ± 2 (9)	13 ± 1 (8)	73 ± 6 (8)	28 ± 1 (4)	13.0 ± 0.7 (5)	72 ± 4 (6)
43	C_15_H_22_O_2_	Eremophilenolide	4.4 ± 0.3 (7)	4.6 ± 0.5 (10)	6.5 ± 0.6 (9)	4.4 ± 0.2 (5)	4.7 ± 0.4 (8)	6.3 ± 0.5 (7)
44	C_24_H_50_NO_7_P	PE (19:0/0:0)	147 ± 16 (11)	13.9 ± 0.4 (3)	99 ± 5 (5)	150 ± 13 (9)	14.0 ± 0.3 (2)	98 ± 4 (4)
45	C_19_H_38_O_4_	1-Monopalmitin	13 ± 2 (12)	11 ± 1 (10)	17.4 ± 0.9 (5)	13 ± 1 (9)	11.1 ± 0.9 (8)	17.2 ± 0.7 (4)
46	C_51_H_84_O_15_	1,2-Di-(9Z,12Z,15Z-octadecatrienoyl)-3-(galactosyl-alpha-1-6-galactosyl-beta-1)-glycerol	88 ± 3 (3)	16.8 ± 0.9 (5)	75 ± 1 (2)	89 ± 2 (2)	16.6 ± 0.7 (4)	74 ± 1 (1)
47	C_45_H_74_O_10_	1,2-Di-(9Z,12Z,15Z-octadecatrienoyl)-3-*O*-Beta-d-galactosyl-sn-glycerol	68 ± 6 (9)	54.8 ± 0.4 (1)	62 ± 4 (6)	69 ± 5 (8)	58 ± 6 (11)	58 ± 7 (12)

*G* granules, *O* oral liquid, *T* tablet

^a^Q markers

^b^Per equivalent to 15.0 g of raw herbal pieces

**Table 4:** Pharmacologically active components found in SHL formula

No.	Name	PubChem CID	CAS	Reported pharmacological activity
1	Chlorogenic Acid^a^	1794427	327-97-9	Antioxidant; antithrombotic; anti-influenza [37]; anti-bacterial [38]
2	Luteolin-7-*O*-glucoside^a^	5280637	5373-11-5	Antioxidant; anti-inflammatory [39]
3	Forsythoside A^a^	5281773	79916-77-1	Anti-pyretic [40]
4	Baicalin^a^	64982	21967-41-9	Anti-viral [41]
5	Forsythin^a^	101712	487-41-2	Regulation of lipid [42]
6	Umbelliferone	5281426	93-35-6	Antioxidant; anti-cancer [43]
7	Piperonal	8438	120-57-0	Antiobesity [44]
8	Methyl caffeate	689075	3843-74-1	Antihyperglycemic and antidiabetic [45]
9	Danielone	146167	90426-22-5	Antifungal activity [46]
10	Geniposidic acid	443354	27741-01-1	Anti-tumor promoting activity [47]
11	Rutin	5280805	1340-08-5	Antimycobacterial [48]
12	Luteolin 3′-glucuronide	10253785	53527-42-7	Flavonoid, as a sedative and digestive [49]
13	Plumieride	72319	511-89-7	Immunostimulatory activity [50]
14	Brusatol	73432	14907-98-3	Anti-cancer (pancreatic cancer) [51]
15	Isovitexin	162350	29702-25-8	Anti-cancer [52]
16	Kaempferide	5281666	491-54-3	Protects against myocardial ischemia/reperfusion injury [53]
17	Baicalein	5281605	491-67-8	Anti-cancer (non-small cell lung cancer) [54]
18	Wogonin	5281703	632-85-9	Anti-cancer (lymphoma) [55]
19	Skullcapflavone II	124211	55084-08-7	Attenuates ovalbumin-induced allergic rhinitis [56]
20	Zwittermicin A	44474866	155547-95-8	Antibiotic, suppressing plant disease [57]

^a^Q-markers

### Multivariate analysis

Multivariate analysis was performed on the UHPLC-MS data obtained from the samples of three SHL preparation forms. The unsupervised principal component analysis (PCA) score plot [36] was first constructed to assess the similarities of chemical components among the three SHL preparation forms and the precision of replicate sample measurements of each preparation form, then the supervised partial least squares discriminant analysis (PLS-DA) score plot was established for pattern recognition of the three SHL preparation forms.

**Fig. 4:**
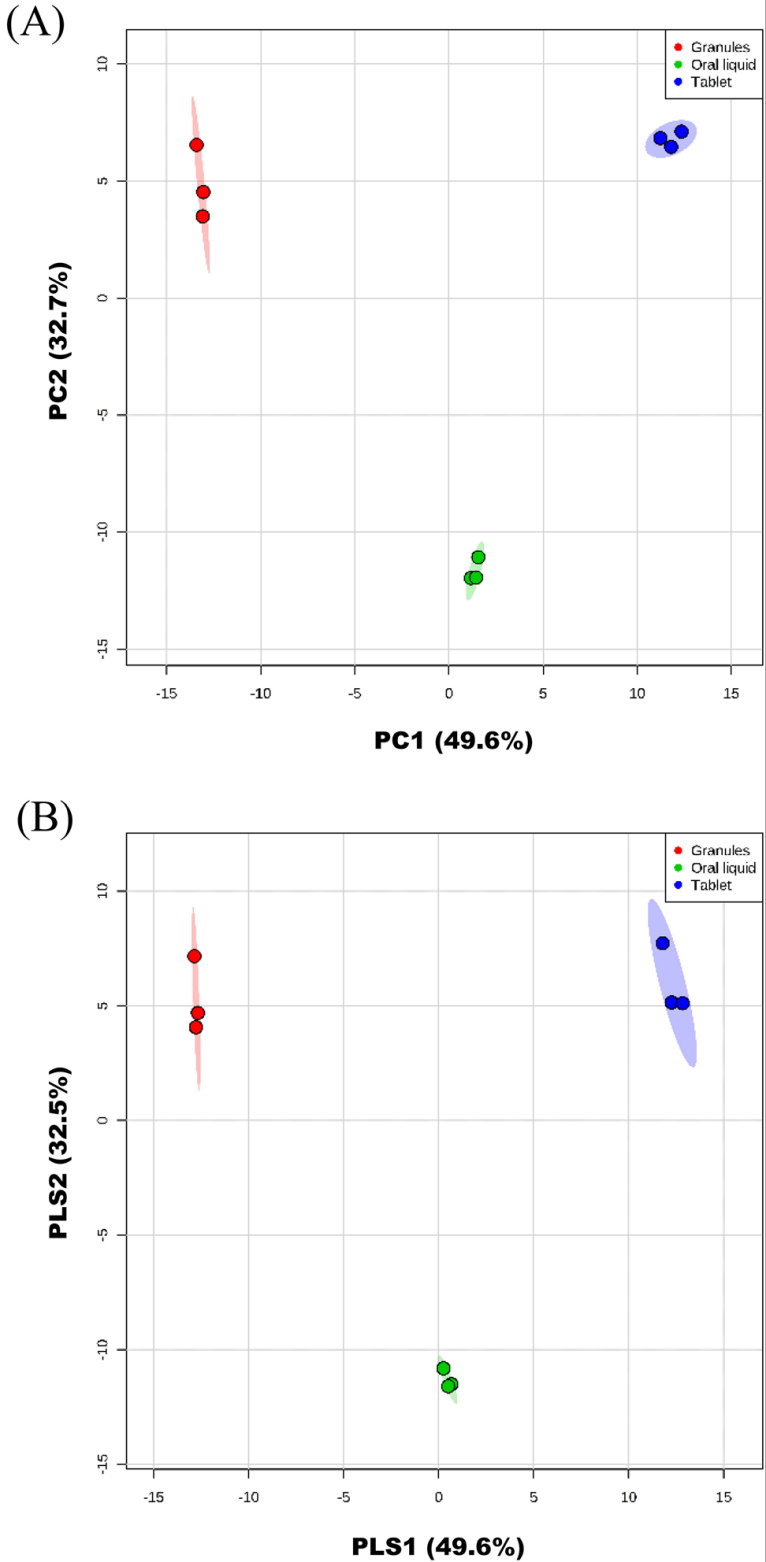
Multivariate data analysis. **A** The 2D PCA score plot, and **B** the 2D PLS-DA score plot of the three SHL preparation forms

**Fig. 5:**
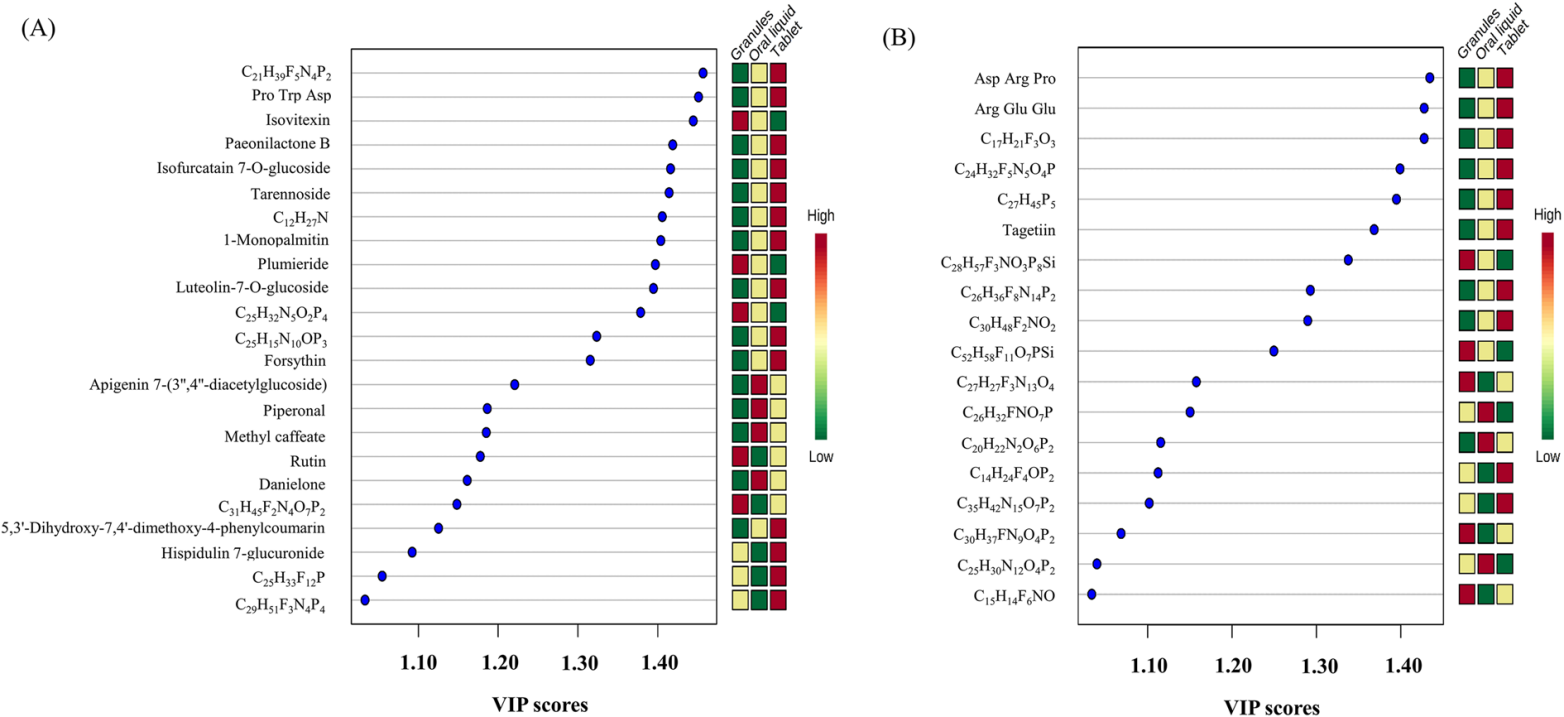
The common components found in all three SHL preparation forms with VIP scores ≥ 1.00. **A** Positive ionization mode, and **B** negative ionization mode

As shown in the PCA score plot (Fig. 4A), the variations of the chemical components among the three SHL forms were evident. The principal component 1 (PC1) and principal component 2 (PC2) scores were 49.6% and 32.7%, respectively, accounting for 82.7% of the total variance. The close grouping of replicate measurements of each preparation form in the PCA score plot indicated excellent precision of the analytical method. The PLS-DA score plot (Fig. 4B) confirmed the finding of the PCA score plot. It displayed distinctive patterns of the three SHL preparation forms, which could be used for product differentiation and recognition. Among the 95 components commonly found in the three SHL preparation forms, the components with variable importance in projection (VIP) scores > 1.00 were considered to contribute to the significant variations in the PLS-DA score plot. These components were listed in Fig. 5, including 23 detected by the positive ionization mode (Fig. 5A) and 18 detected by the negative ionization mode (Fig. 5B), and their VIP scores were tabulated in Additional file 12: Table S9.

### Pharmacologically active components in SHL formula

Despite significant variations in the chemical compositions of the granule, oral liquid, and tablet forms of SHL formula, these preparation forms have been used interchangeably in clinical practices to treat the same illnesses. Therefore, it is rational to think that the pharmacologically active components were among the 47 components commonly identified in all three SHL preparation forms. In contrast, the unique components in each SHL preparation form may come from the different geographic origins, agricultural and industrial pollutions of the herbs, and the byproducts associated with the unique manufacturing conditions.

The pharmacological activities of the 47 commonly identified chemical components were explored through database searching and text mining. Twenty out of 47 were found to have various pharmacological activities (Table 4), including anti-bacterial, anti-viral, antipyretic, anti-inflammatory, and anti-influenza activities, and immunostimulatory, anti-cancer, anti-oxidative and antibiotic [37–57], etc. These pharmacologically active components may serve alone or in combination as lead compounds for new drug development and used as ligands for retrieval of protein targets for the mechanistic study of SHL formula in treating URTIs or other related diseases.

## Conclusions

A UHPLC-QTOF-MS/MS method has been implemented for untargeted and targeted metabolomics analyses of the SHL formula. This method is accurate and precise and can be used for component profiling, identification, semi-quantitative analysis, and cross-comparison among different TCM preparation forms. In this work, the chemical components of the SHL formula in three preparation forms (i.e., granule, oral liquid, and tablet) were obtained, the 47 common components were identified and quantitated, and the pharmacologically active components were investigated. PCA and PLS-DA were performed to assess and visualize the correlations and differences among the three SHL preparation forms and the reproducibility of technical and biological replicates. This method is useful for component fingerprinting, quality assessment, and counterfeit detection of SHL formulas and related products.

## Supplementary Information

**Additional file 1: Appendix S1.** Preparation of MS tuning mix and reference mass solutions.

**Additional file 2: Figure S1.** The average MS/MS spectra of the 47 commonly identified components in all three SHL preparation forms for all collision energies (10, 20, and 40 eV) by their predominant ESI modes.

**Additional file 3: Figure S2.** The proposed fragmentation pathways of the commonly identified compounds.

**Additional file 4: Table S1.** The chemical components identified with both names and formulas in SHL granule preparation form.

**Additional file 5: Table S2.** The chemical components identified only with formulas in SHL granule preparation form.

**Additional file 6: Table S3.** The chemical components identified with both names and formulas in SHL oral liquid preparation form.

**Additional file 7: Table S4.** The chemical components identified with only formulas in SHL oral liquid preparation form.

**Additional file 8: Table S5.** The chemical components identified with both names and formulas in SHL tablet preparation form.

**Additional file 9: Table S6.** The chemical components identified with only formulas in SHL tablet preparation form.

**Additional file 10: Table S7.** The chemical components found in each SHL preparation forms.

**Additional file 11: Table S8.** The common chemical components unidentified (or identified with formulas only) in all three SHL preparation forms.

**Additional file 12: Table S9.** The common components found in all three SHL preparation forms with VIP scores > 1.00.

## Data Availability

Data beyond those in Additional files are available upon request.

## Abbreviations

SHL: *Shuang Huang Lian*
TCM: Traditional Chinese medicine
URTIs: Upper respiratory tract infections
UHPLC-QTOF-MS/MS: Ultra-high-performance liquid chromatography and quadrupole time-of-flight tandem mass spectrometry
PCA: Principal component analysis
PLS-DA: Partial least squares discriminant analysis
RSV: Respiratory syncytial virus
MFs: Matrix factors
CV: Coefficient variation
AJS-ESI: Agilent jet stream electrospray ionization
PEEK: Polyether ether ketone
CE: Collision energy
MPP: Mass profiler professional (MPP)
TCMID: Traditional Chinese medicine integrated database
TCMSP: Traditional Chinese medicine systems pharmacology
PC1: Principal component 1
PC2: Principal component 2
VIP: Variable importance in projection
Q markers: Quality markers
