# Supplementary material for: Metabolomics analyses of traditional Chinese medicine formula Shuang Huang Lian by UHPLC-QTOF-MS/MS
Source: Chin Med. 2022 May 30;17:62. doi: 10.1186/s13020-022-00610-x (PMC9150355; doi:10.1186/s13020-022-00610-x)
Supplement: Supplementary file 1 — Additional file 1: Appendix S1. Preparation of MS tuning mix and reference mass solutions. [file 13020_2022_610_MOESM1_ESM.docx]

**Appendix S1**

**Preparation of MS tuning mix solutions**

For the positive AJS ESI mode, the tuning mix solution was prepared by mixing 10.0 mL of ESI-L Low Concentration Tuning Mix (Agilent Part Number: G1969-85000), 85.5 mL of acetonitrile, 5 μL of 0.1 mM HP-0321 (included in the ESI TOF Biopolymer Analysis Reference Mass Standards Kit, Agilent Part Number: G1969-85003), and 4.5 mL of deionized water. For the negative AJS ESI mode, the tuning mix solution was prepared by mixing 2.5 mL of ESI-L Low Concentration Tuning Mix (Agilent Part Number: G1969-85000), 95.6 mL of acetonitrile, and 1.9 mL of deionized water. Typical tune mass abundances are in the range of 50,000 to 480,000 counts. If the largest abundance is exceeded 480,000 counts, a dilution of the tuning mix solution is needed to bring down the largest abundance below 480,000 counts.

**Preparation of reference mass solution**

For the Q-TOF MS internal mass correction and verification in the positive and negative AJS ESI modes, the reference mass solution was used and prepared in a 1-liter Nalgene bottle (Agilent Part Number: 9301-6460) by mixing 0.4 mL of 5.0 mM of purine and 1.0 mL of 2.5 mM HP-0921 (both included in ES-TOF Reference Mass Solution Kit, Agilent Part Number: G1969-85001), 950.0 mL of acetonitrile, and 50.0 mL of deionized water.
