# Supplementary material for: Metabolomics analyses of traditional Chinese medicine formula Shuang Huang Lian by UHPLC-QTOF-MS/MS
Source: Chin Med. 2022 May 30;17:62. doi: 10.1186/s13020-022-00610-x (PMC9150355; doi:10.1186/s13020-022-00610-x)
Supplement: Supplementary file 2 — Additional file 2: Figure S1. The average MS/MS spectra of the 47 commonly identified components in all three SHL preparation forms for all collision energies (10, 20, and 40 eV) by their predominant ESI modes. [file 13020_2022_610_MOESM2_ESM.pdf]

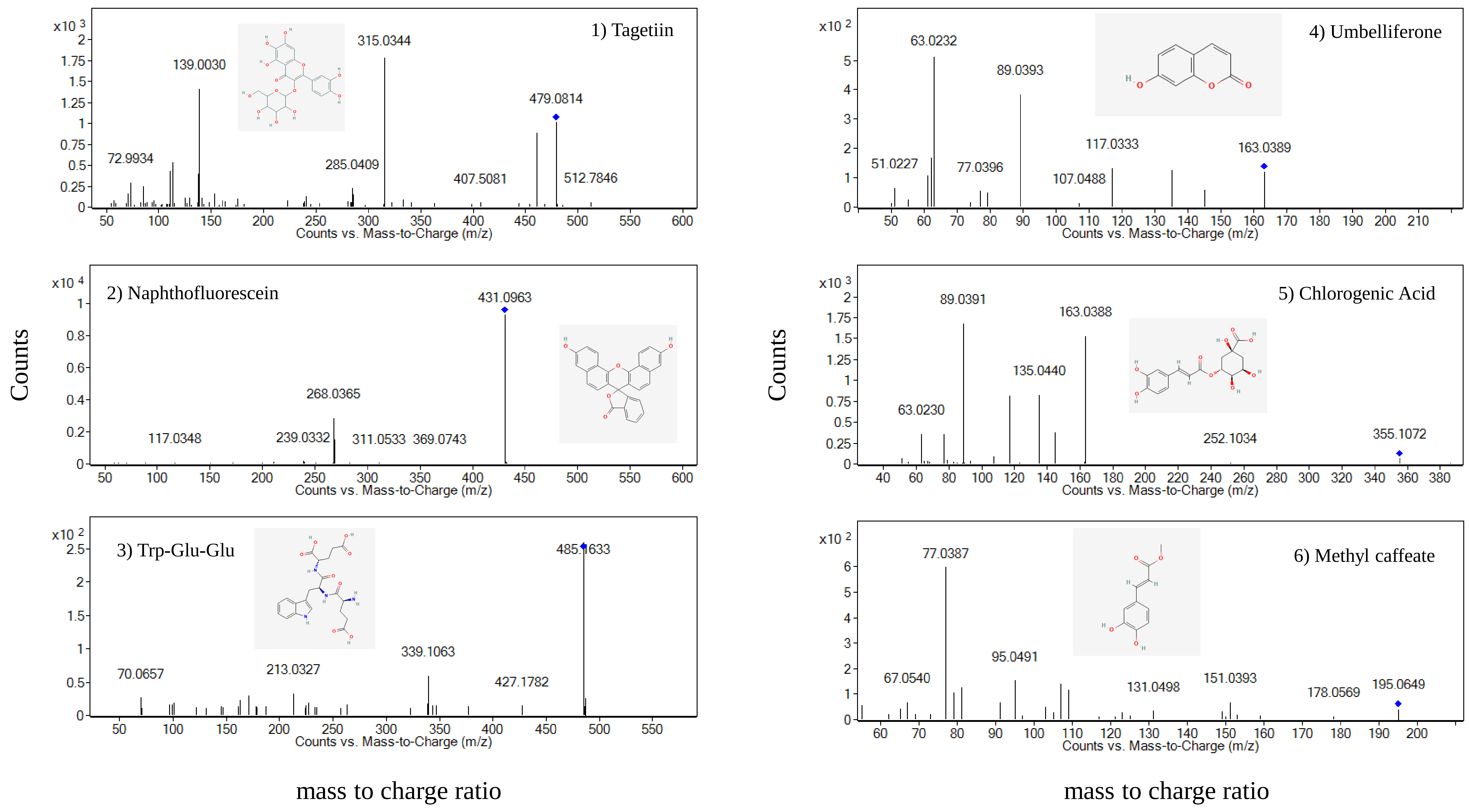

1) Tagetiin
139.0030
315.0344
479.0814
72.9934
285.0409
407.5081
512.7846
2) Naphthofluorescein
431.0963
268.0365
117.0348
239.0332
311.0533
369.0743
3) Trp-Glu-Glu
485.1633
70.0657
213.0327
339.1063
427.1782
4) Umbelliferone
63.0232
89.0393
117.0333
163.0389
51.0227
77.0396
107.0488
5) Chlorogenic Acid
89.0391
163.0388
135.0440
63.0230
252.1034
355.1072
6) Methyl caffeate
77.0387
95.0491
67.0540
131.0498
151.0393
178.0569
195.0649
Counts vs. Mass-to-Charge (m/z)
Counts
mass to charge ratio

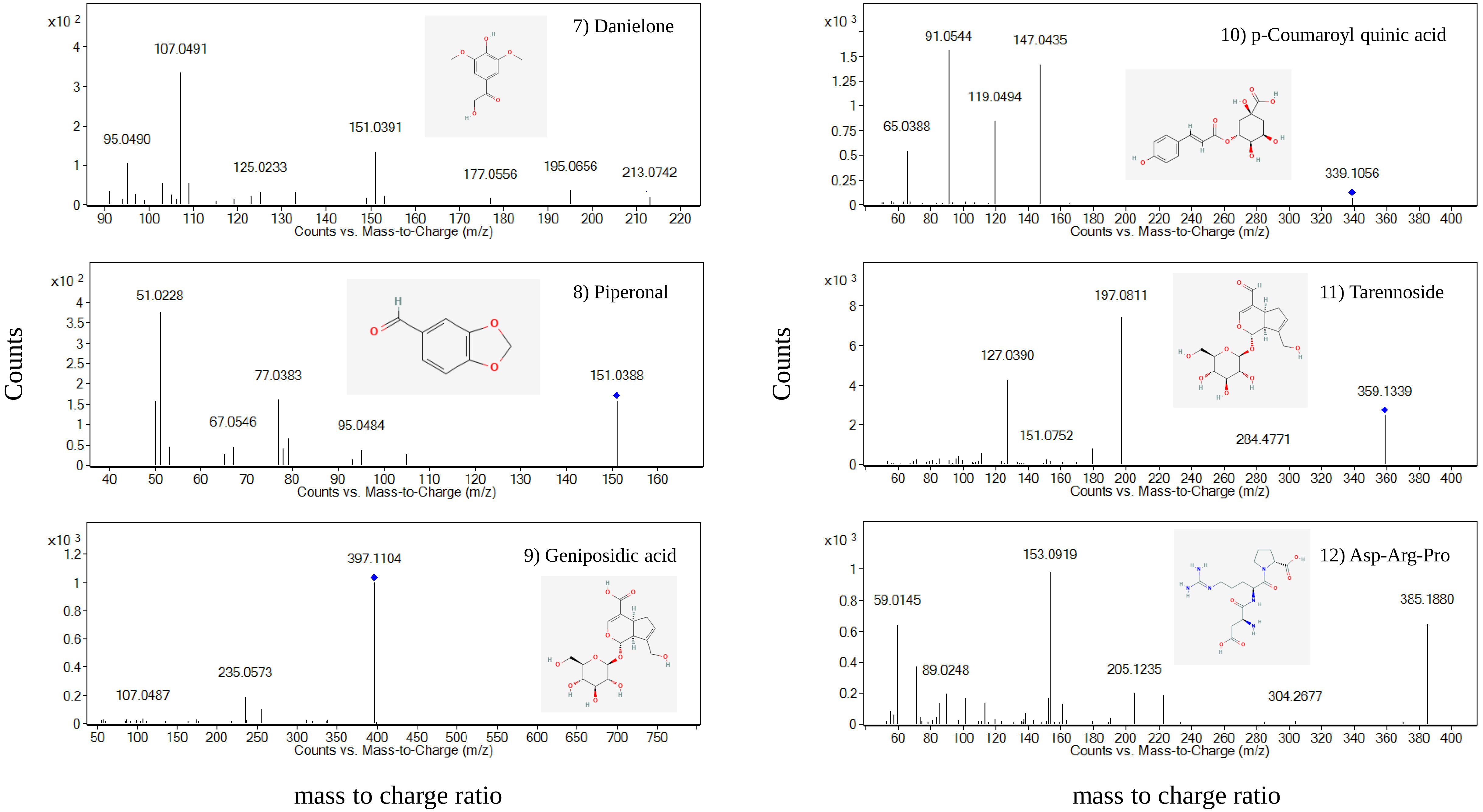
7) Danielone
x10 2
107.0491
95.0490
151.0391
125.0233
177.0556
195.0656
213.0742
Counts vs. Mass-to-Charge (m/z)
10) p-Coumaroyl quinic acid
x10 3
91.0544
147.0435
119.0494
65.0388
339.1056
Counts vs. Mass-to-Charge (m/z)
8) Piperonal
x10 2
51.0228
77.0383
67.0546
95.0484
151.0388
Counts vs. Mass-to-Charge (m/z)
11) Tarennoside
x10 3
197.0811
127.0390
151.0752
284.4771
359.1339
Counts vs. Mass-to-Charge (m/z)
9) Geniposidic acid
x10 3
397.1104
235.0573
107.0487
Counts vs. Mass-to-Charge (m/z)
12) Asp-Arg-Pro
x10 3
153.0919
59.0145
89.0248
205.1235
304.2677
385.1880
Counts vs. Mass-to-Charge (m/z)
Counts
Counts
mass to charge ratio
mass to charge ratio

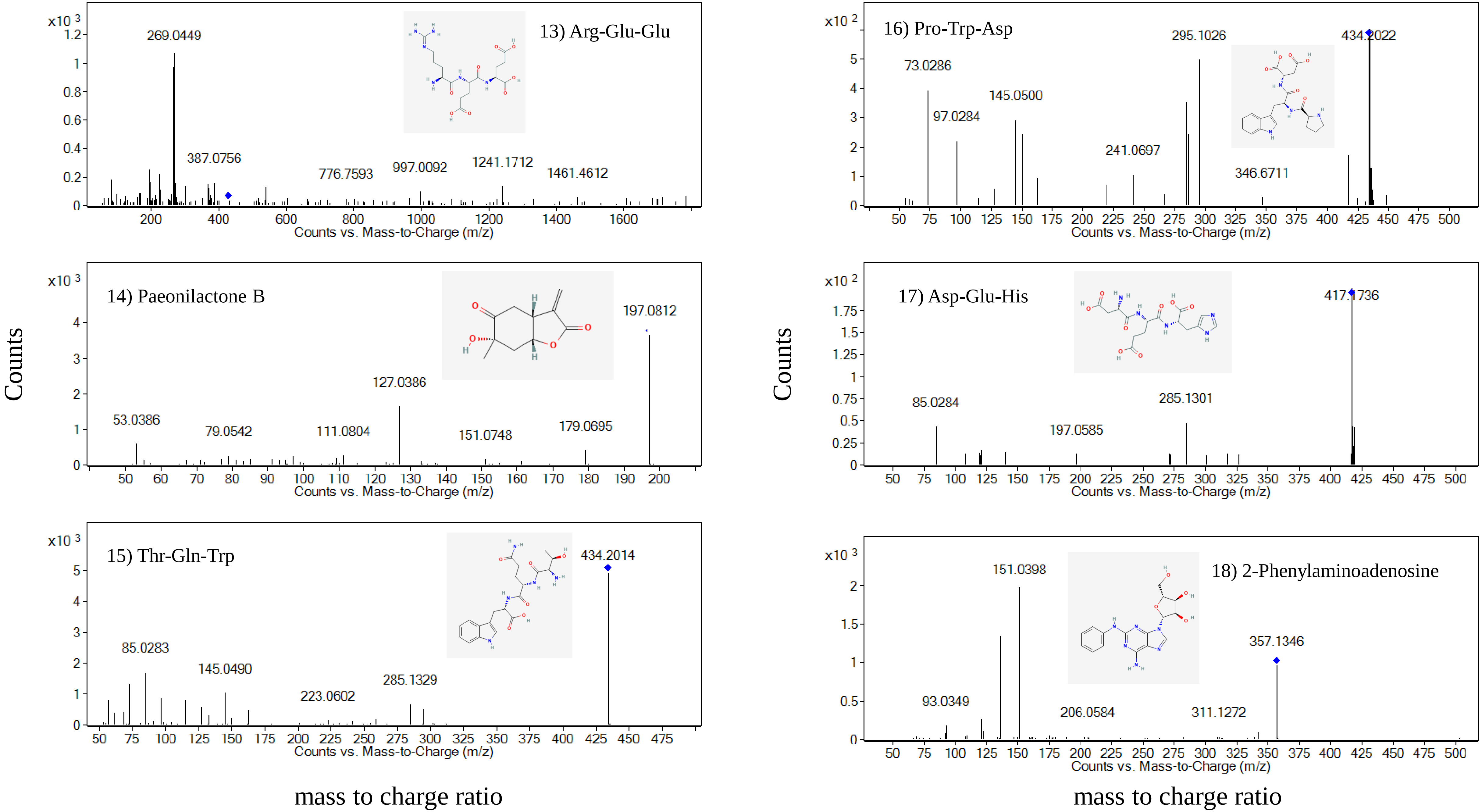

Counts
Counts
x10 3
13) Arg-Glu-Glu
269.0449
387.0756
776.7593
997.0092
1241.1712
1461.4612
Counts vs. Mass-to-Charge (m/z)
x10 2
16) Pro-Trp-Asp
73.0286
97.0284
145.0500
241.0697
295.1026
346.6711
434.2022
Counts vs. Mass-to-Charge (m/z)
x10 3
14) Paeonilactone B
53.0386
79.0542
111.0804
127.0386
151.0748
179.0695
197.0812
Counts vs. Mass-to-Charge (m/z)
x10 2
17) Asp-Glu-His
85.0284
197.0585
285.1301
417.1736
Counts vs. Mass-to-Charge (m/z)
x10 3
15) Thr-Gln-Trp
85.0283
145.0490
223.0602
285.1329
434.2014
Counts vs. Mass-to-Charge (m/z)
x10 3
18) 2-Phenylaminoadenosine
93.0349
151.0398
206.0584
311.1272
357.1346
Counts vs. Mass-to-Charge (m/z)
mass to charge ratio
mass to charge ratio

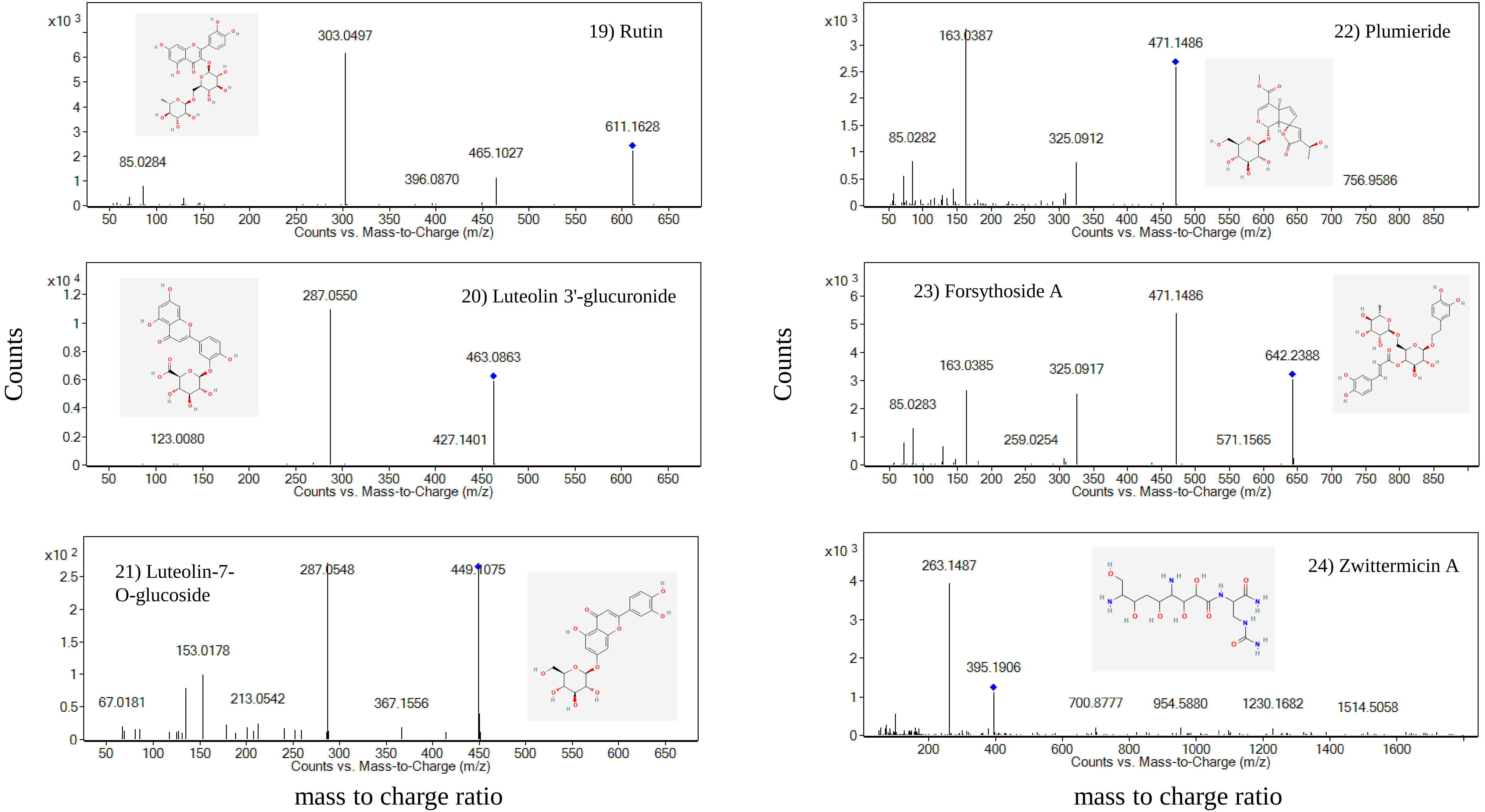
19) Rutin
x10 3
303.0497
611.1628
465.1027
396.0870
85.0284
Counts vs. Mass-to-Charge (m/z)
22) Plumieride
x10 3
163.0387
471.1486
85.0282
325.0912
756.9586
Counts vs. Mass-to-Charge (m/z)
20) Luteolin 3'-glucuronide
x10 4
287.0550
463.0863
427.1401
123.0080
Counts vs. Mass-to-Charge (m/z)
23) Forsythoside A
x10 3
471.1486
642.2388
163.0385
325.0917
85.0283
259.0254
571.1565
Counts vs. Mass-to-Charge (m/z)
21) Luteolin-7-
O-glucoside
x10 2
287.0548
449.1075
153.0178
67.0181
213.0542
367.1556
Counts vs. Mass-to-Charge (m/z)
24) Zwittermicin A
x10 3
263.1487
395.1906
700.8777
954.5880
1230.1682
1514.5058
Counts vs. Mass-to-Charge (m/z)
Counts
Counts
mass to charge ratio
mass to charge ratio

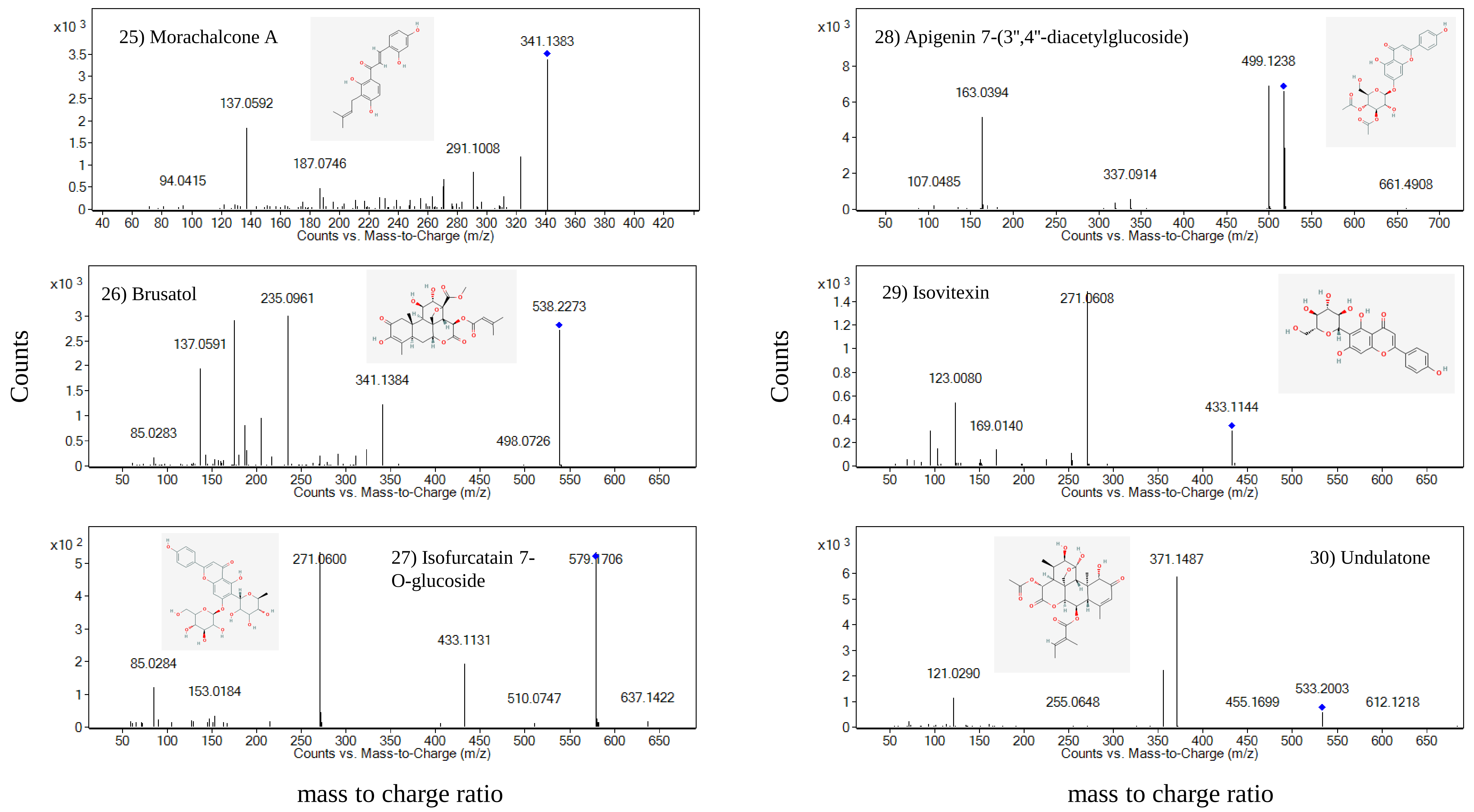

Counts
mass to charge ratio
x10 3
25) Morachalcone A
341.1383
137.0592
94.0415
187.0746
291.1008
Counts vs. Mass-to-Charge (m/z)
x10 3
26) Brusatol
235.0961
538.2273
137.0591
341.1384
85.0283
498.0726
Counts vs. Mass-to-Charge (m/z)
x10 2
27) Isofurcatain 7-O-glucoside
271.0600
579.1706
433.1131
85.0284
153.0184
510.0747
637.1422
Counts vs. Mass-to-Charge (m/z)
x10 3
28) Apigenin 7-(3",4"-diacetylglucoside)
499.1238
163.0394
107.0485
337.0914
661.4908
Counts vs. Mass-to-Charge (m/z)
x10 3
29) Isovitexin
271.0608
123.0080
169.0140
433.1144
Counts vs. Mass-to-Charge (m/z)
x10 3
30) Undulatone
371.1487
121.0290
255.0648
455.1699
533.2003
612.1218
Counts vs. Mass-to-Charge (m/z)

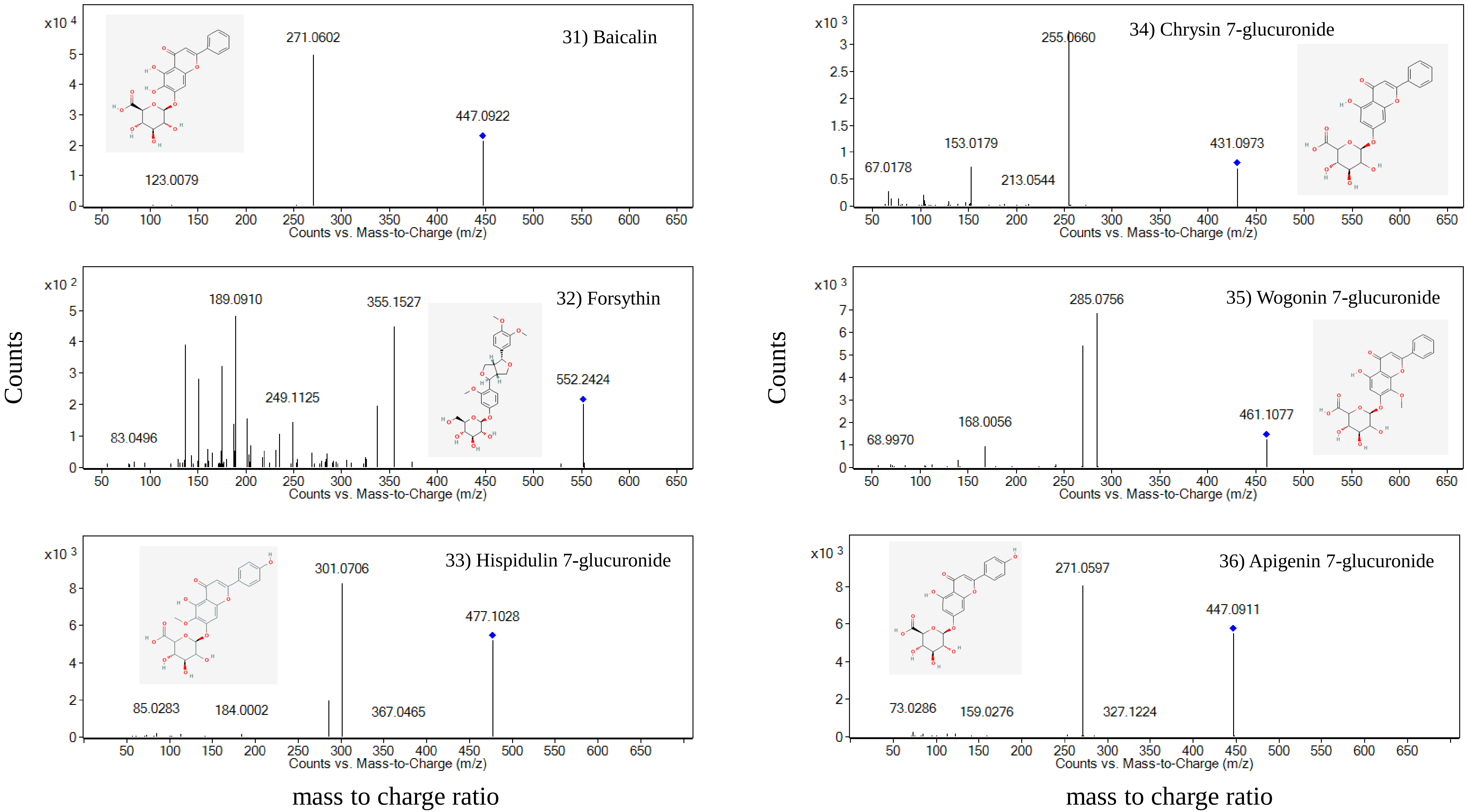

31) Baicalin
x10 4
271.0602
447.0922
123.0079
34) Chrysin 7-glucuronide
x10 3
255.0660
153.0179
431.0973
67.0178
213.0544
32) Forsythin
x10 2
189.0910
355.1527
552.2424
249.1125
83.0496
35) Wogonin 7-glucuronide
x10 3
285.0756
168.0056
461.1077
68.9970
33) Hispidulin 7-glucuronide
x10 3
301.0706
477.1028
85.0283
184.0002
367.0465
36) Apigenin 7-glucuronide
x10 3
271.0597
447.0911
73.0286
159.0276
327.1224
Counts vs. Mass-to-Charge (m/z)
Counts
mass to charge ratio

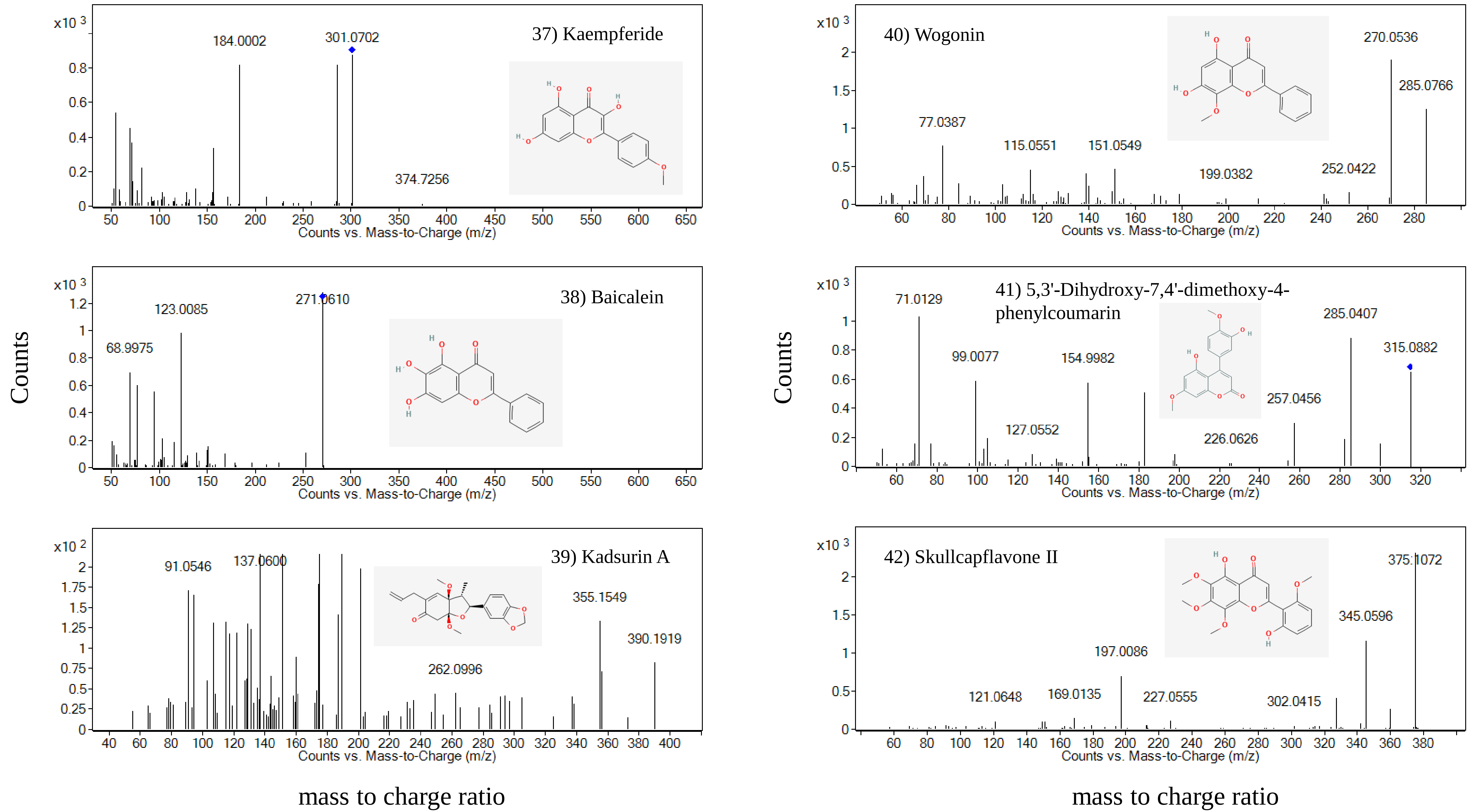

37) Kaempferide
184.0002
301.0702
374.7256
38) Baicalein
271.0610
123.0085
68.9975
39) Kadsurin A
91.0546
137.0600
355.1549
390.1919
262.0996
40) Wogonin
270.0536
285.0766
77.0387
115.0551
151.0549
199.0382
252.0422
41) 5,3'-Dihydroxy-7,4'-dimethoxy-4-phenylcoumarin
71.0129
285.0407
315.0882
99.0077
154.9982
257.0456
127.0552
226.0626
42) Skullcapflavone II
375.1072
345.0596
197.0086
121.0648
169.0135
227.0555
302.0415
Counts vs. Mass-to-Charge (m/z)
Counts
mass to charge ratio

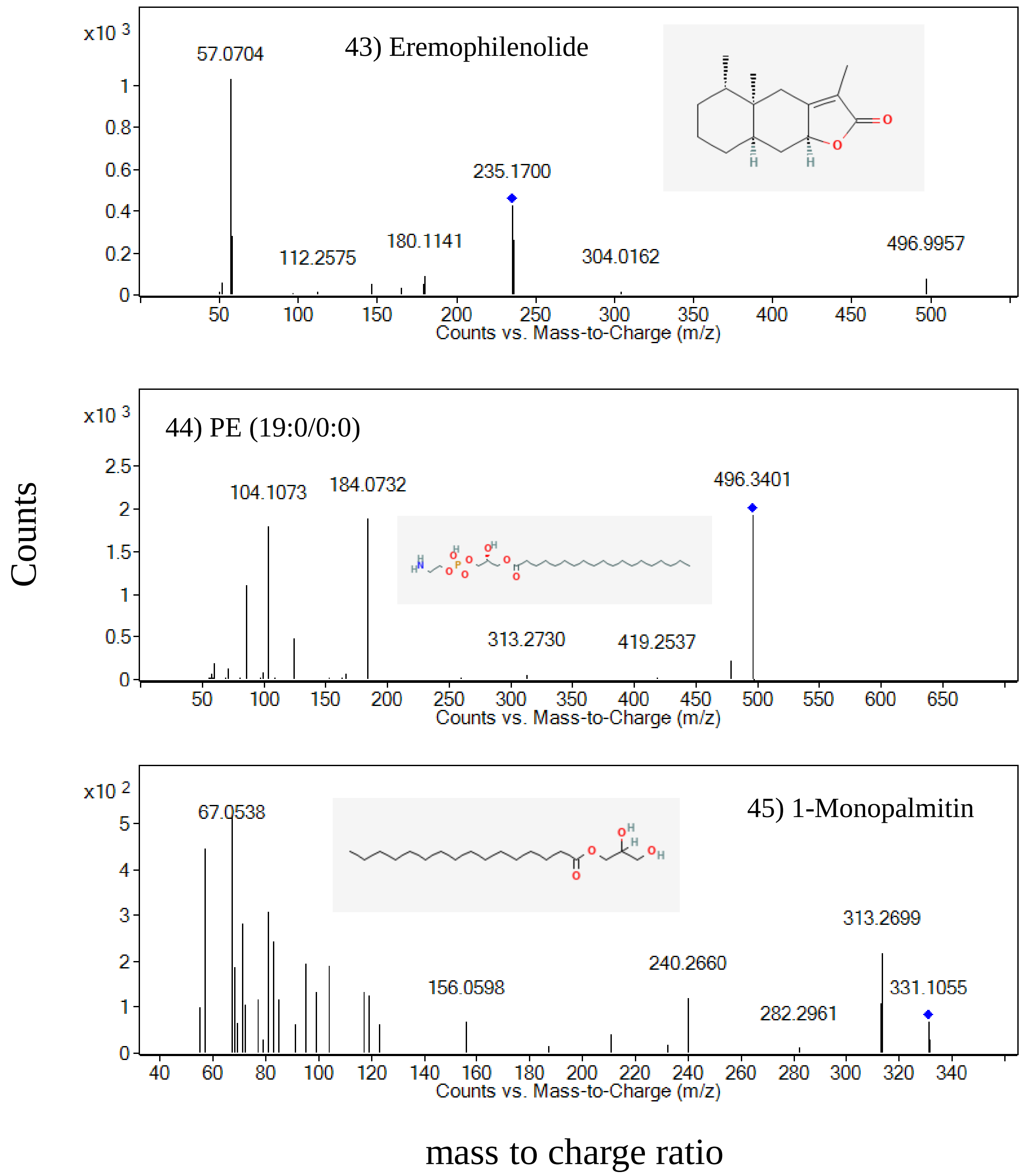

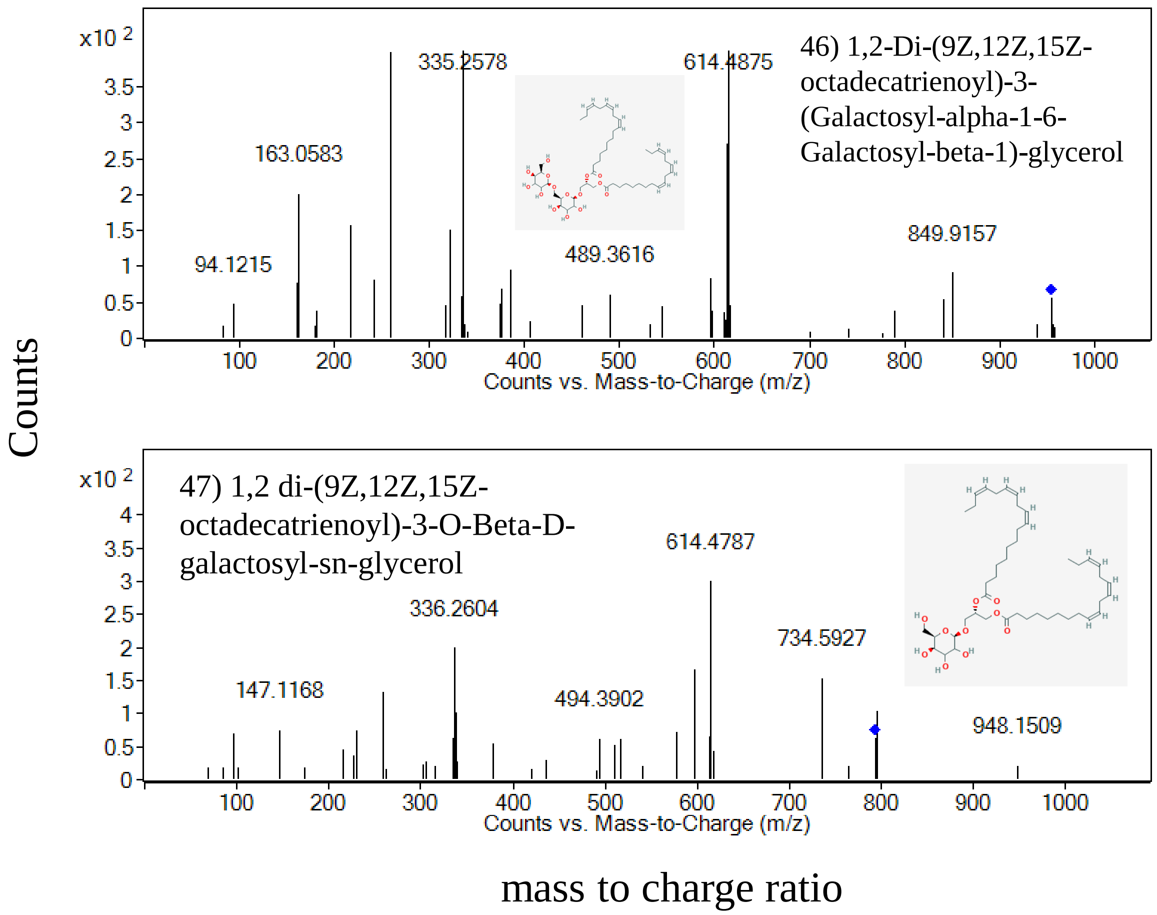

Figure legend:

Averaged MSMS spectra of 47 common chemical constituents at collision energies (10, 20, 40 eV) from their predominant ESI mode, detected and identified in all three Shuanghuanglian preparation forms.
