# Supplementary material for: Metabolomics analyses of traditional Chinese medicine formula Shuang Huang Lian by UHPLC-QTOF-MS/MS
Source: Chin Med. 2022 May 30;17:62. doi: 10.1186/s13020-022-00610-x (PMC9150355; doi:10.1186/s13020-022-00610-x)

#1 Tagetiin

1) MS/MS spectrum

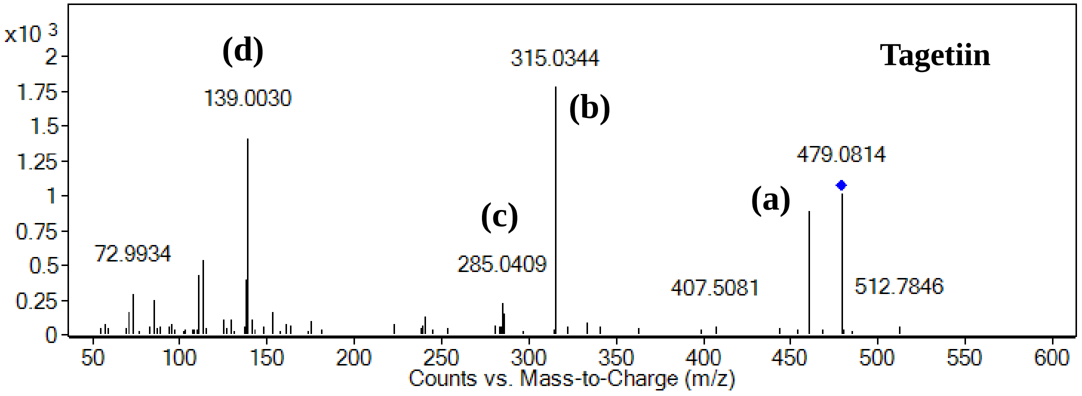

2) MS/MS fragment table

	Mass	Intensity	Weight(%)	No. of candid.	Best score
1	461.0731	841.13	28.9	6	97.9
2	315.0358	1479.51	23.8	1	71.5
3	285.0593	535.53	7.0	5	76.0
4	267.0295	455.71	5.3	4	78.5
5	443.0690	126.85	4.0	4	34.3
6	333.0466	223.50	4.0	0	0.0
7	139.0033	1231.12	3.9	1	93.7
8	284.0574	270.76	3.5	5	58.5
9	462.0949	99.81	3.5	0	0.0
10	268.0320	251.05	2.9	4	33.8

3) Fragmentation pathway

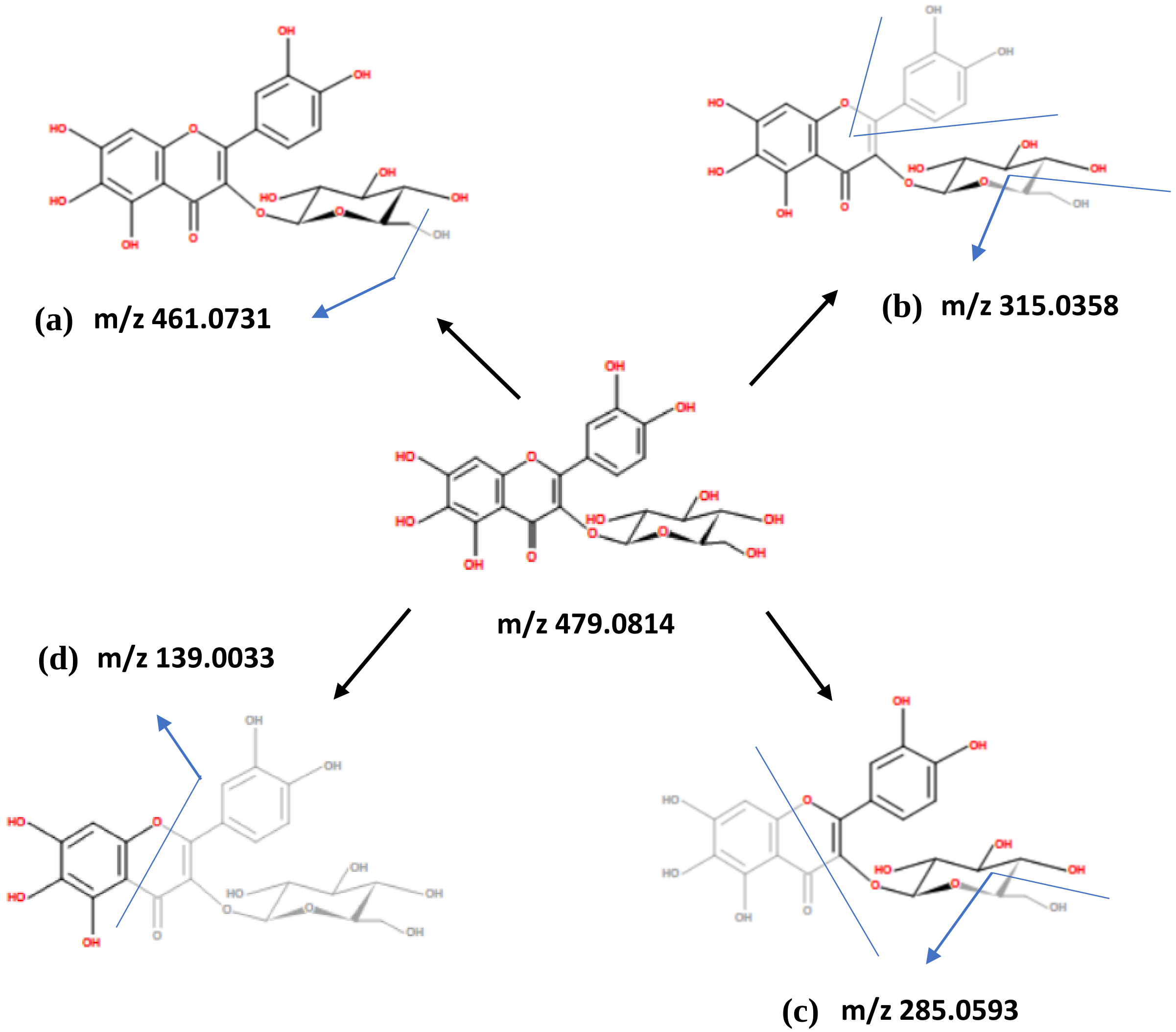

#2 Naphthofluorescein

Naphthofluorescein
$[M-H]^-$

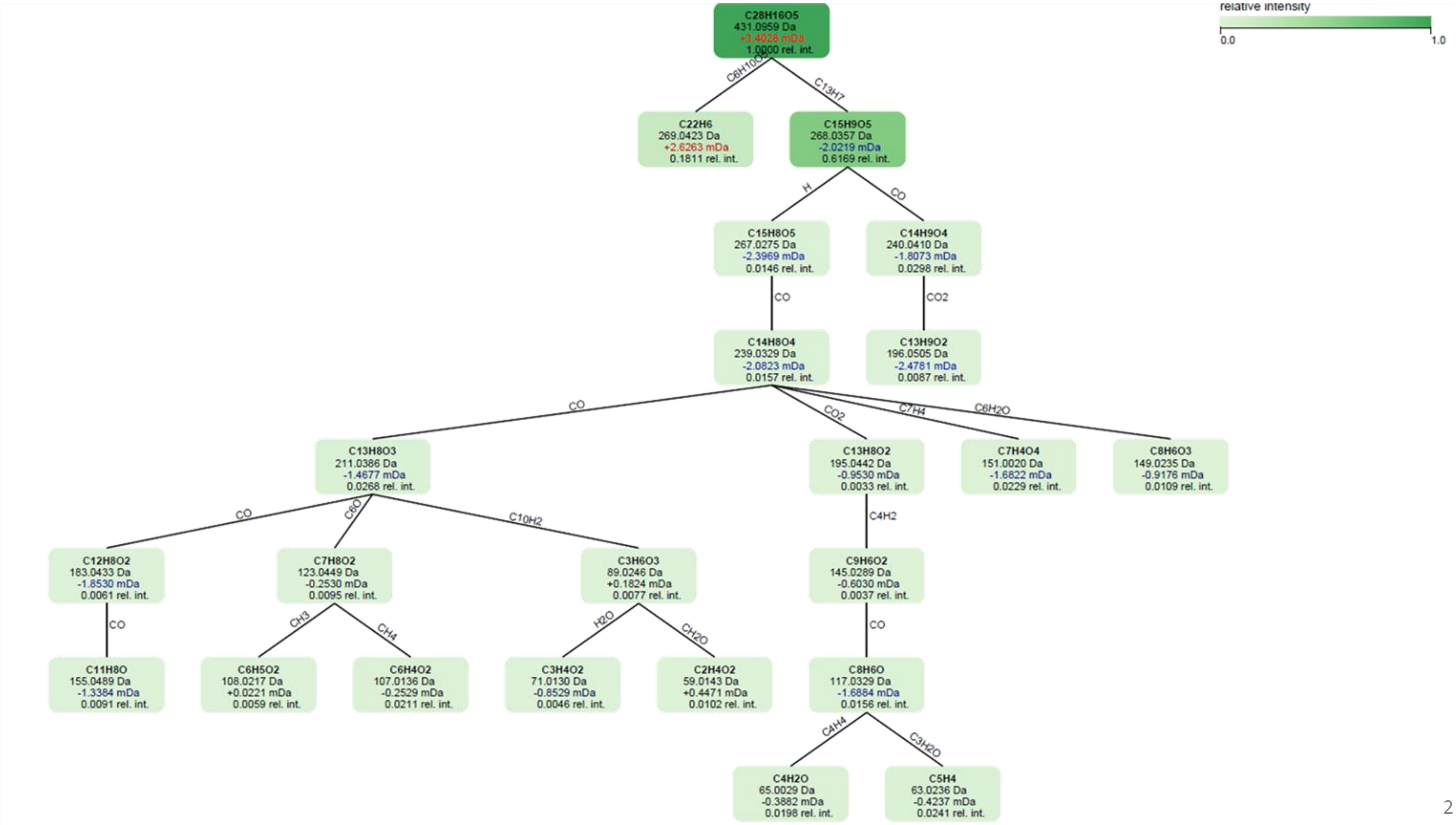

#4 Umbelliferone

1) MS/MS spectrum

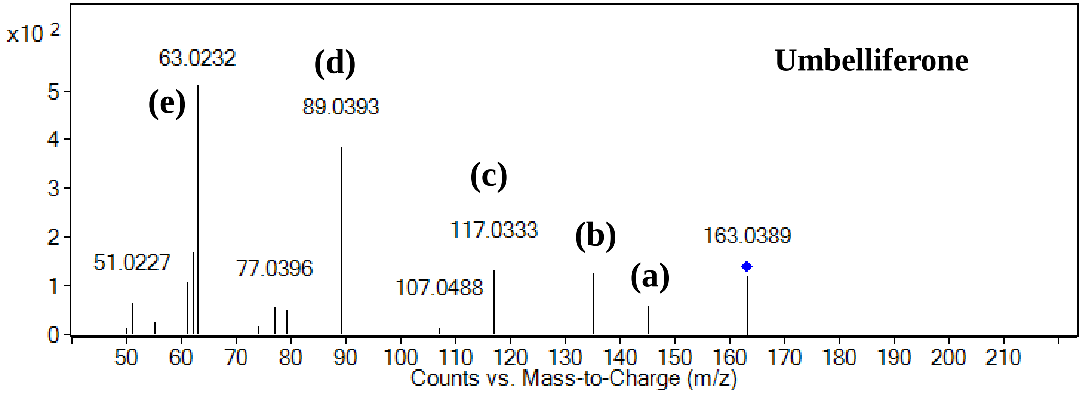

2) MS/MS fragment table

	Mass	Intensity	Weight(%)	No. of candid.	Best score
1	135.0435	720.48	26.6	5	93.2
2	89.0383	1404.81	22.5	1	63.6
3	117.0331	682.69	18.9	2	87.0
4	145.0277	401.68	17.1	3	89.6
5	63.0229	564.16	4.5	1	30.0
6	107.0491	173.87	4.0	5	89.4
7	77.0389	289.18	3.5	1	80.8
8	79.0543	76.93	1.0	3	76.4
9	135.0205	13.68	0.5	0	0.0
10	107.0654	17.36	0.4	0	0.0

3) Fragmentation pathway

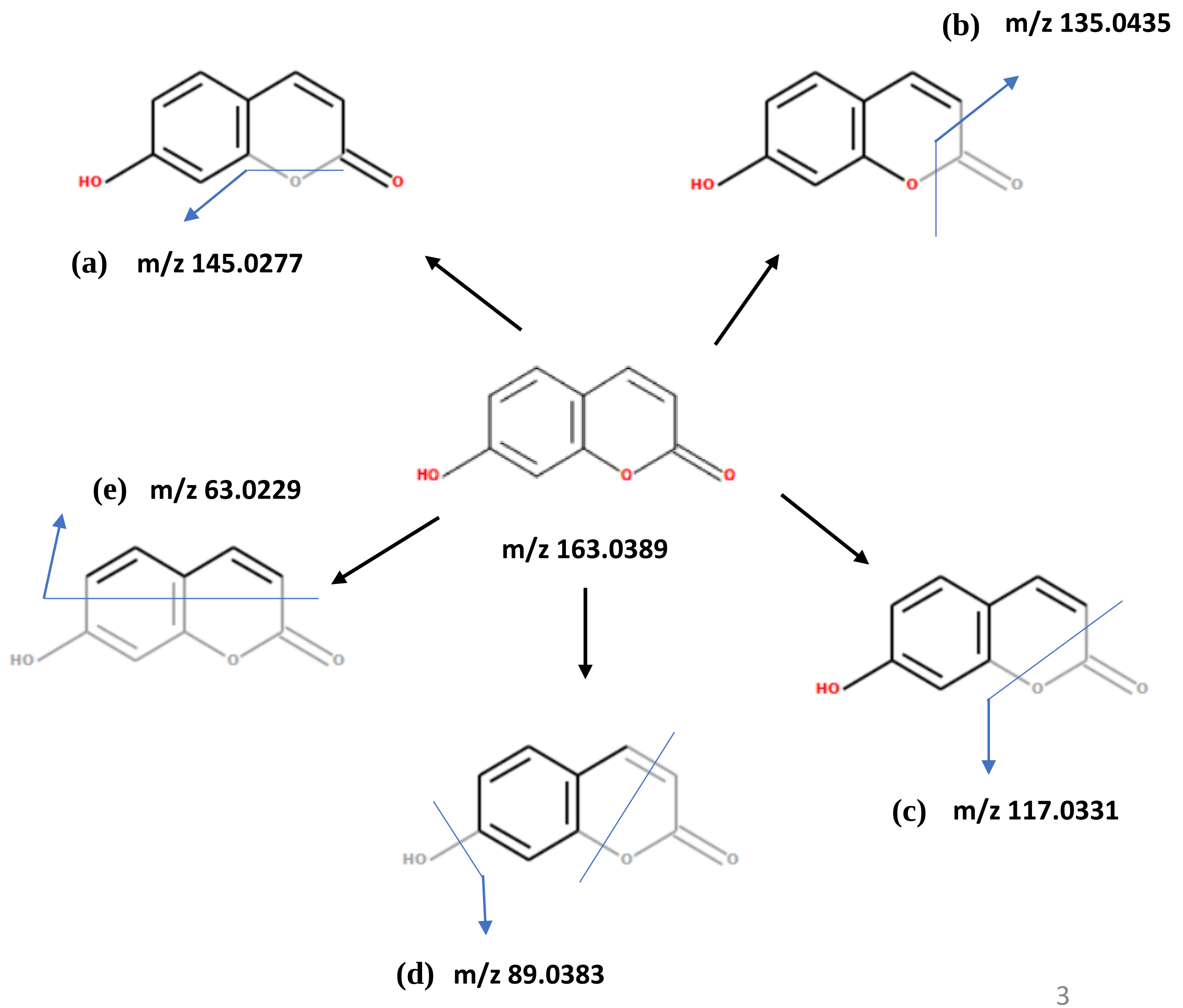

#5 Chlorogenic Acid

1) MS/MS spectrum

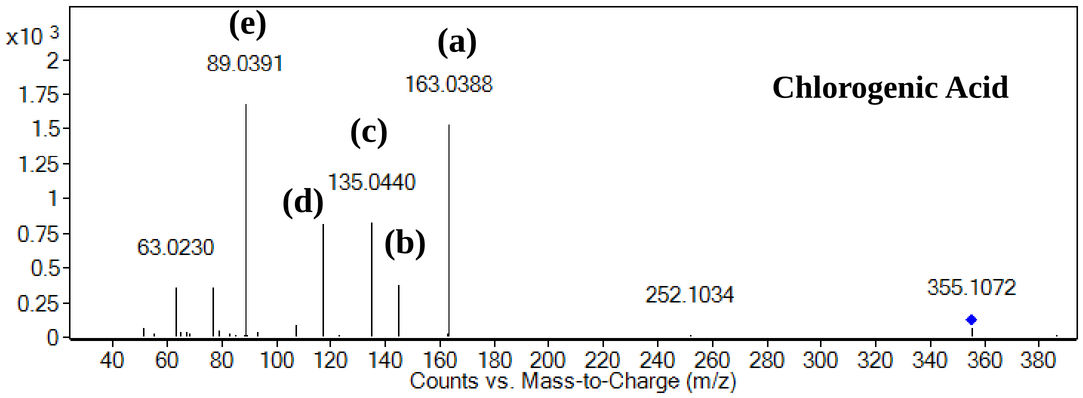

2) MS/MS fragment table

	Mass	Intensity	Weight(%)	No. of candid.	Best score
1	163.0387	16028.08	78.3	3	98.3
2	145.0278	1697.16	6.6	4	82.3
3	135.0440	1661.47	5.6	1	97.7
4	117.0332	1284.55	3.2	1	78.6
5	89.0386	792.12	1.2	2	48.3
6	79.0544	265.46	0.3	4	50.7
7	107.0487	172.10	0.4	4	75.9
8	83.0493	169.23	0.2	4	73.9
9	77.0386	136.94	0.1	4	41.0
10	163.0942	99.97	0.5	5	79.0

3) Fragmentation pathway

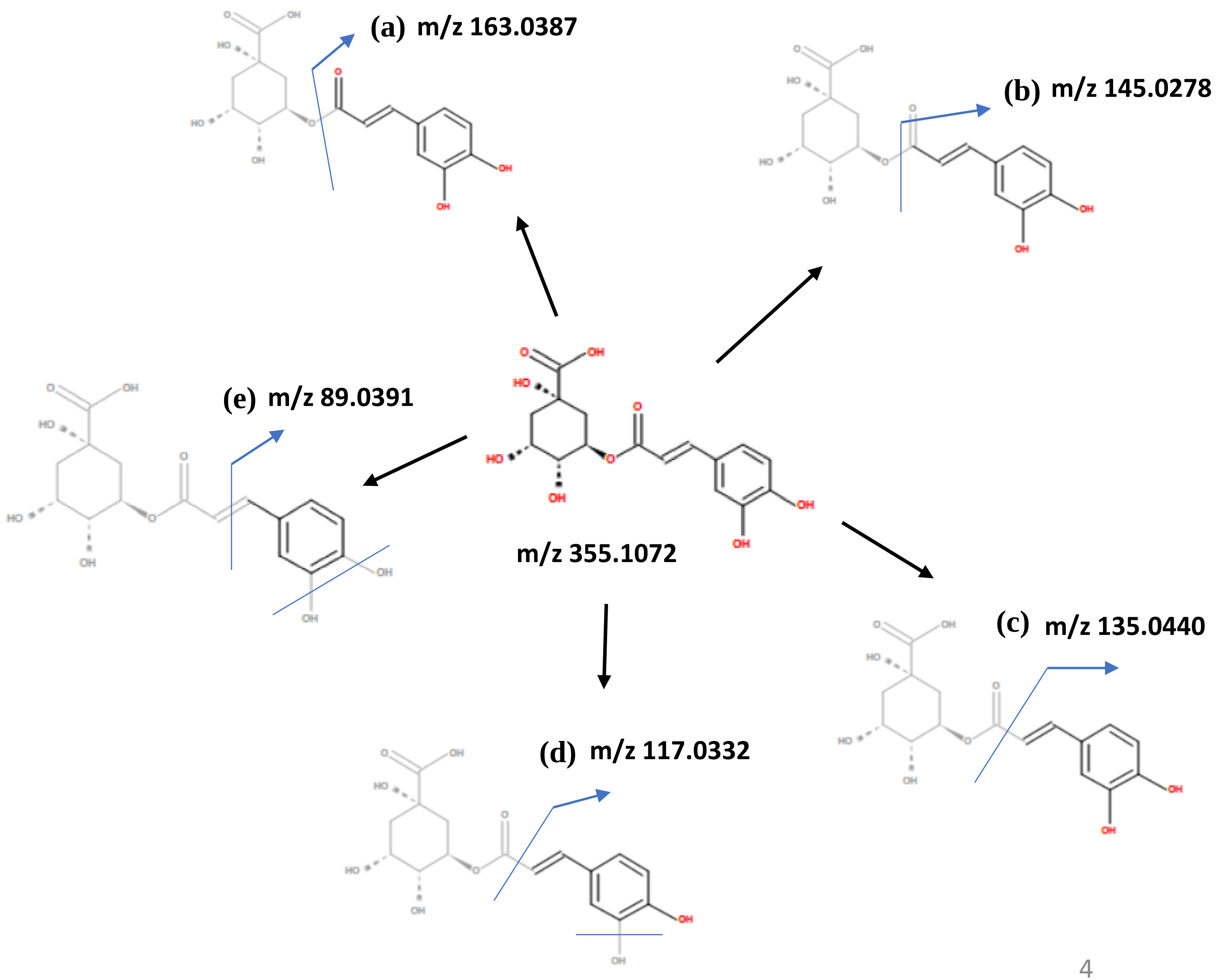

#6 Methyl caffeate

1) MS/MS spectrum

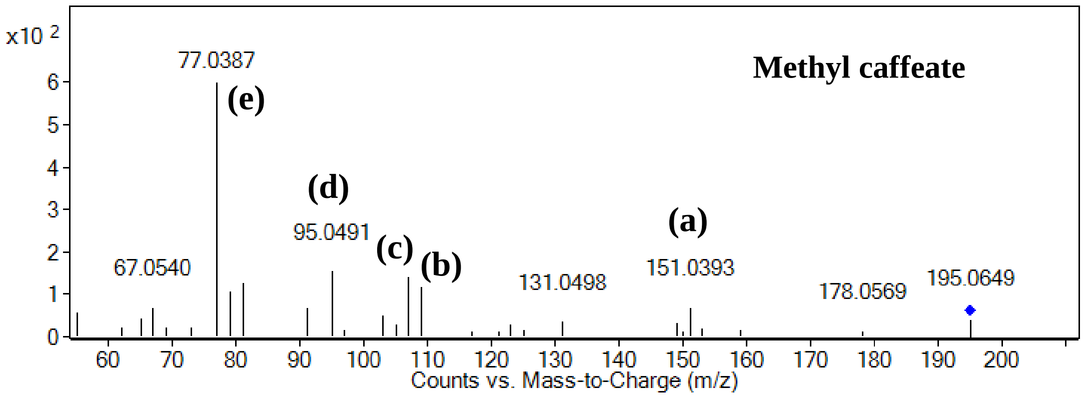

2) MS/MS fragment table

	Mass	Intensity	Weight(%)	No. of candid.	Best score
1	77.0385	564.32	19.9	4	40.9
2	107.0483	152.93	10.4	4	74.4
3	51.0227	602.11	9.3	3	40.9
4	151.0384	58.87	8.0	4	45.3
5	95.0491	137.13	7.4	5	56.0
6	177.0544	23.76	4.4	2	91.0
7	103.0546	66.55	4.2	1	71.1
8	79.0540	110.36	4.1	4	50.7
9	109.0281	54.58	3.9	4	71.3
10	121.0647	34.57	3.0	1	87.5

3) Fragmentation pathway

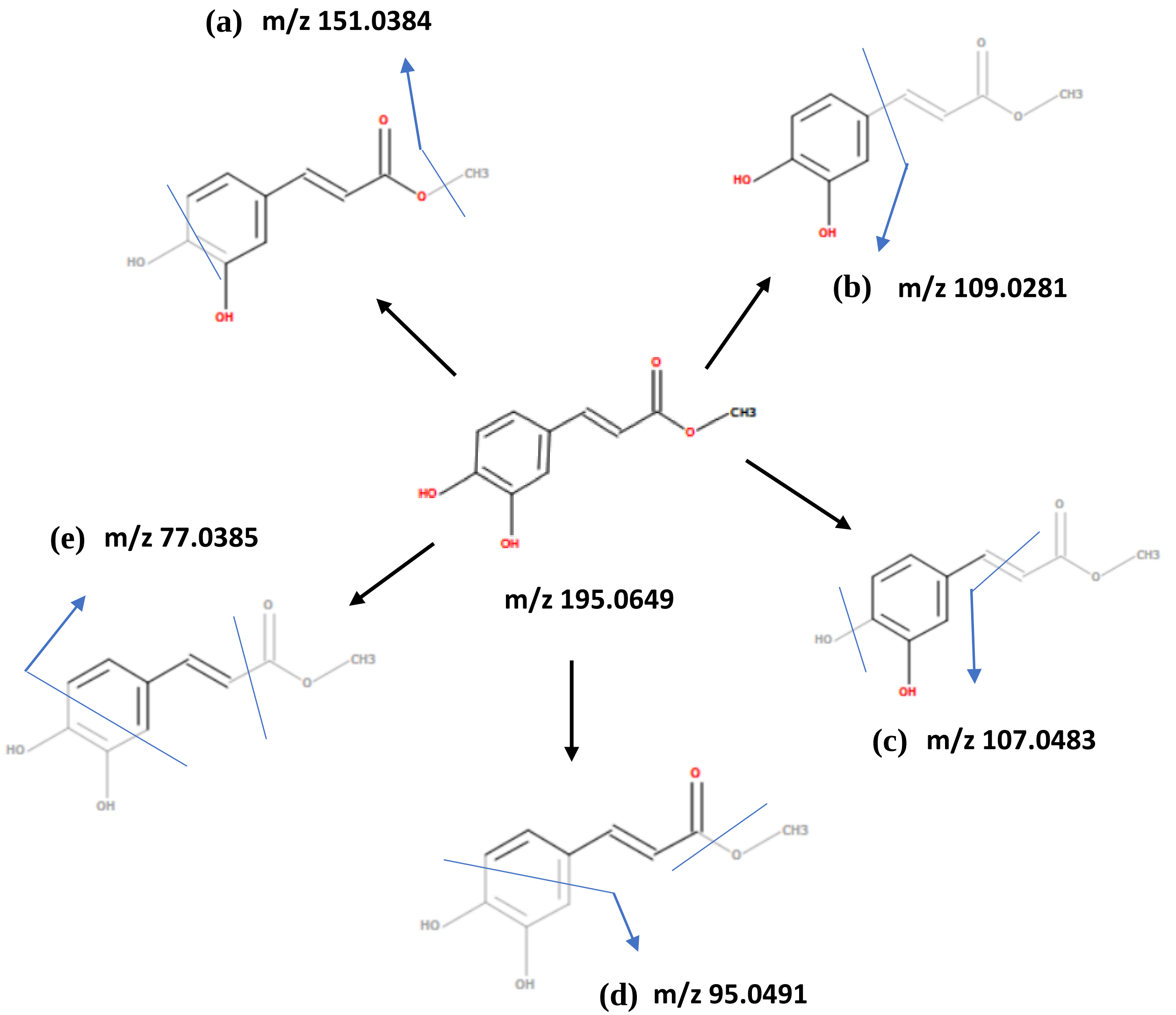

#7 Danielone

1) MS/MS spectrum

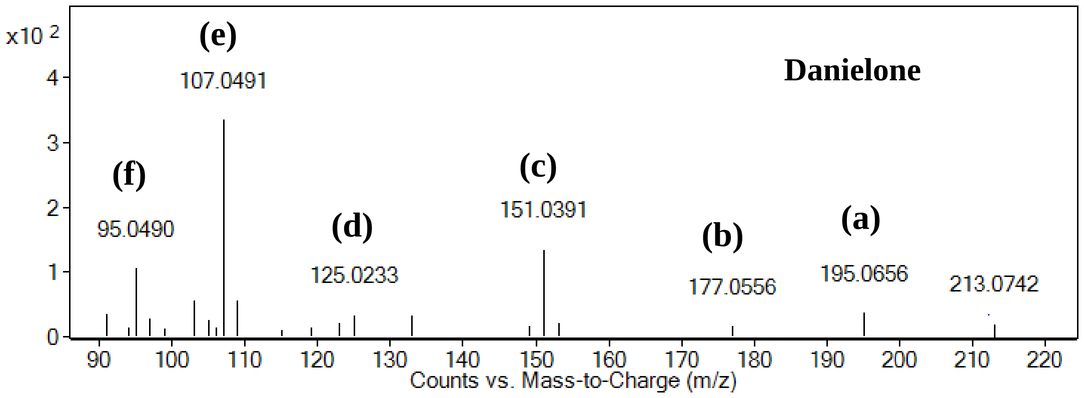

2) MS/MS fragment table

	Mass	Intensity	Weight(%)	No. of candid.	Best score
1	77.0386	639.74	16.8	0	0.0
2	107.0489	325.94	16.5	3	61.0
3	151.0390	92.62	9.4	7	91.1
4	177.0545	63.49	8.8	3	83.3
5	195.0633	39.39	6.6	3	88.1
6	51.0230	514.12	5.9	2	30.0
7	95.0493	111.08	4.4	7	36.3
8	79.0541	153.17	4.2	0	0.0
9	153.0555	33.30	3.5	11	93.0
10	125.0234	49.44	3.4	5	71.5

3) Fragmentation pathway

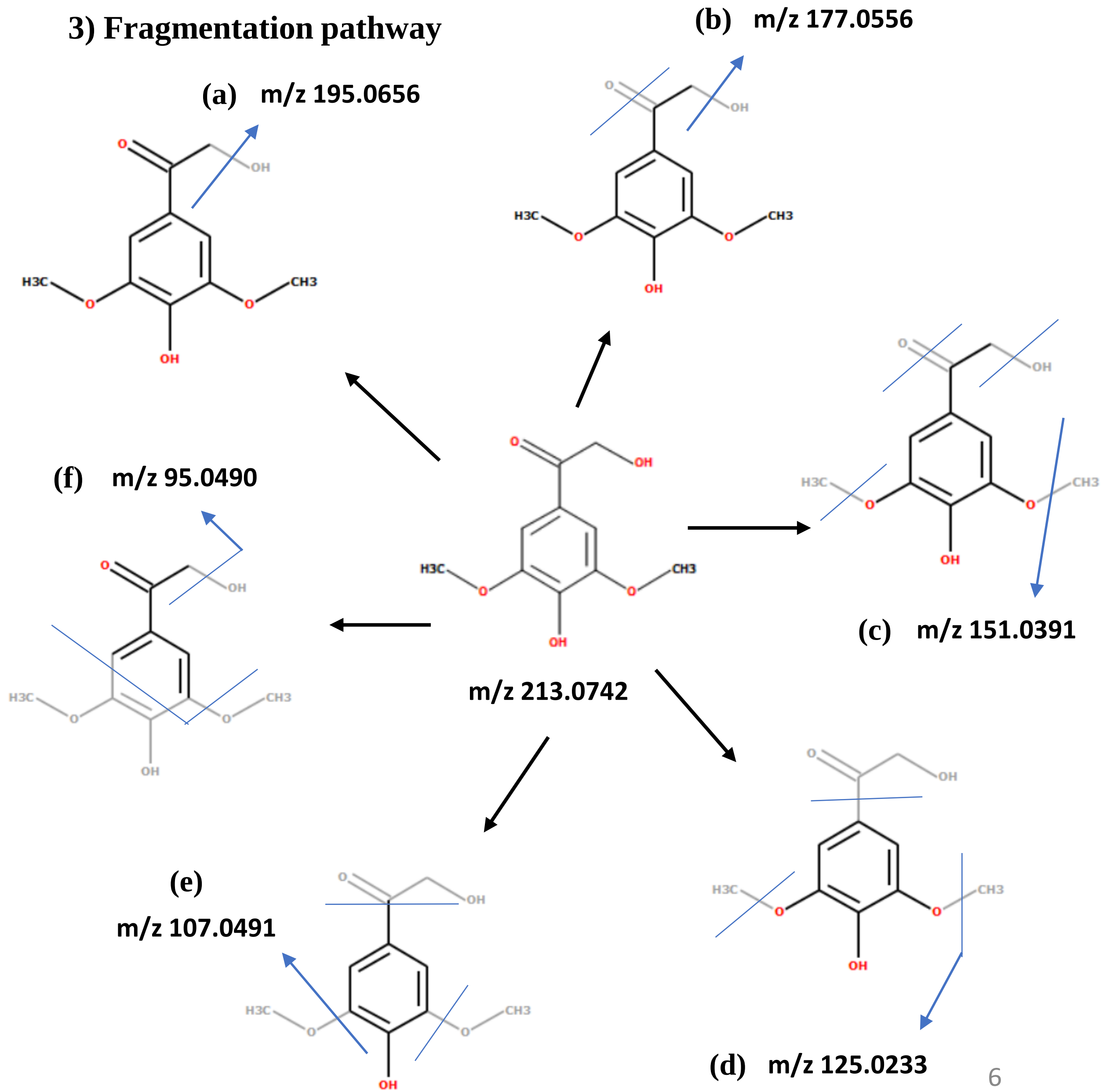

#8 Piperonal

1) MS/MS spectrum

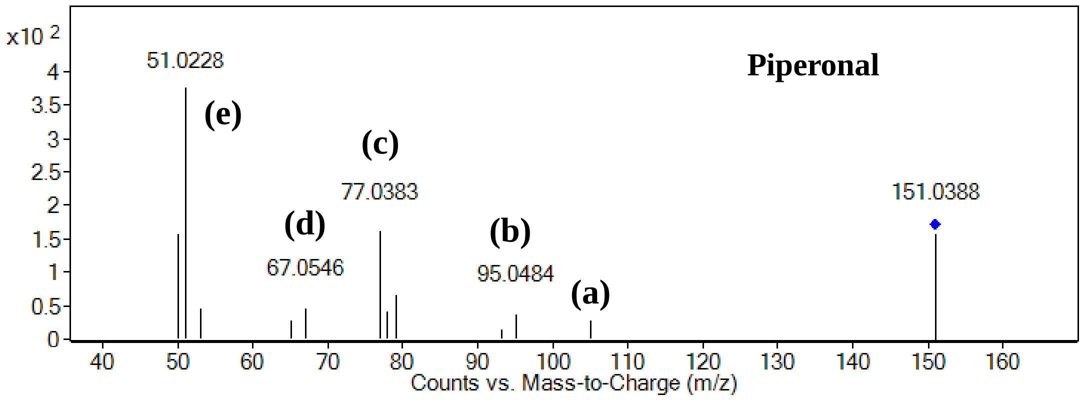

2) MS/MS fragment table

	Mass	Intensity	Weight(%)	No. of candid.	Best score
1	77.0388	921.76	31.1	1	66.3
2	95.0496	367.78	18.9	5	60.7
3	51.0232	715.74	10.6	4	38.5
4	123.0432	102.02	8.8	3	94.2
5	107.0486	101.35	6.6	3	95.6
6	67.0550	199.86	5.1	4	47.1
7	105.0325	70.58	4.4	3	93.2
8	65.0383	144.79	3.5	4	40.8
9	79.0544	51.73	1.8	2	61.1
10	106.0654	26.52	1.7	0	0.0

3) Fragmentation pathway

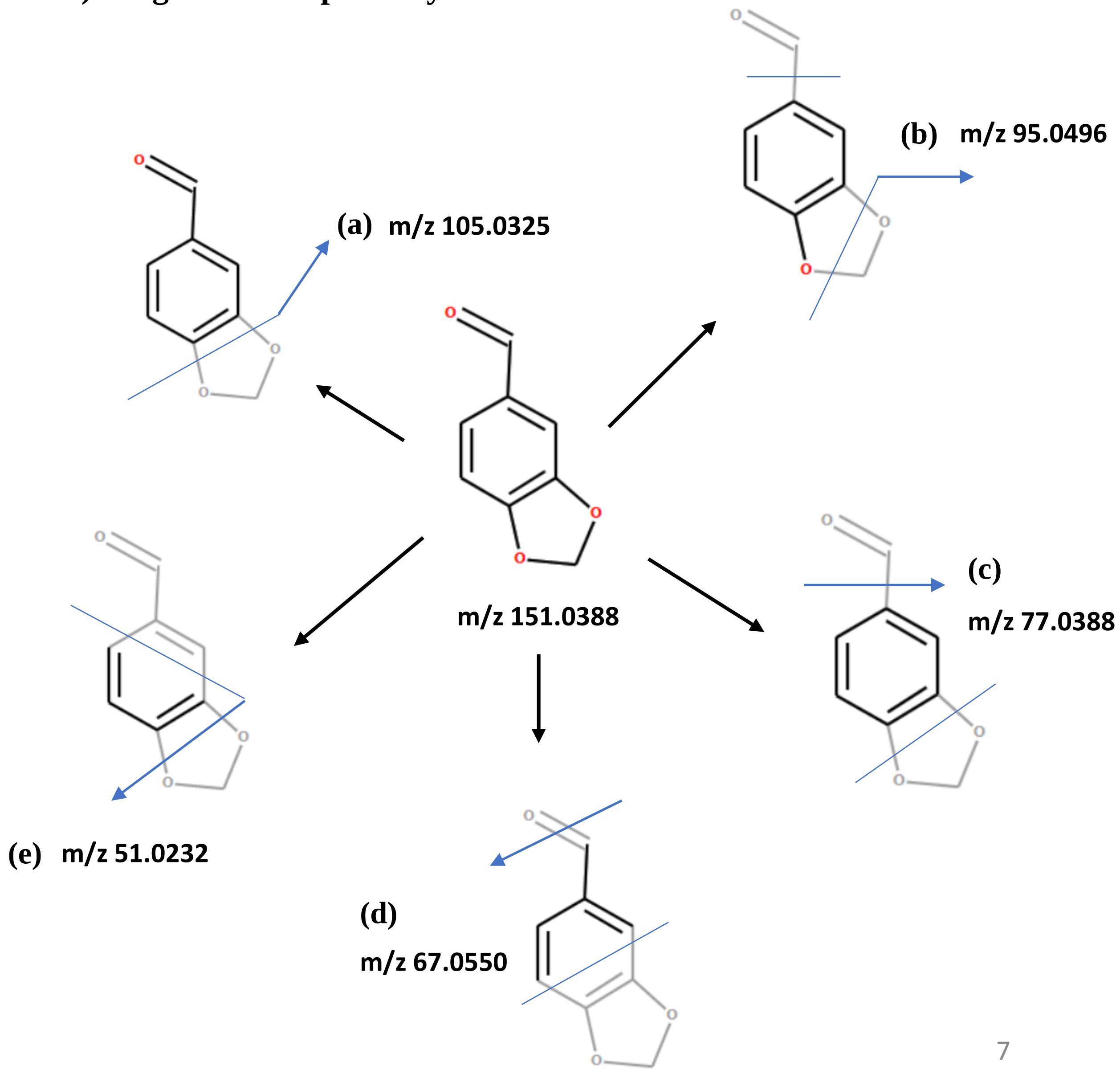

#9 Geniposidic acid

Geniposidic acid
[M+Na]$^+$

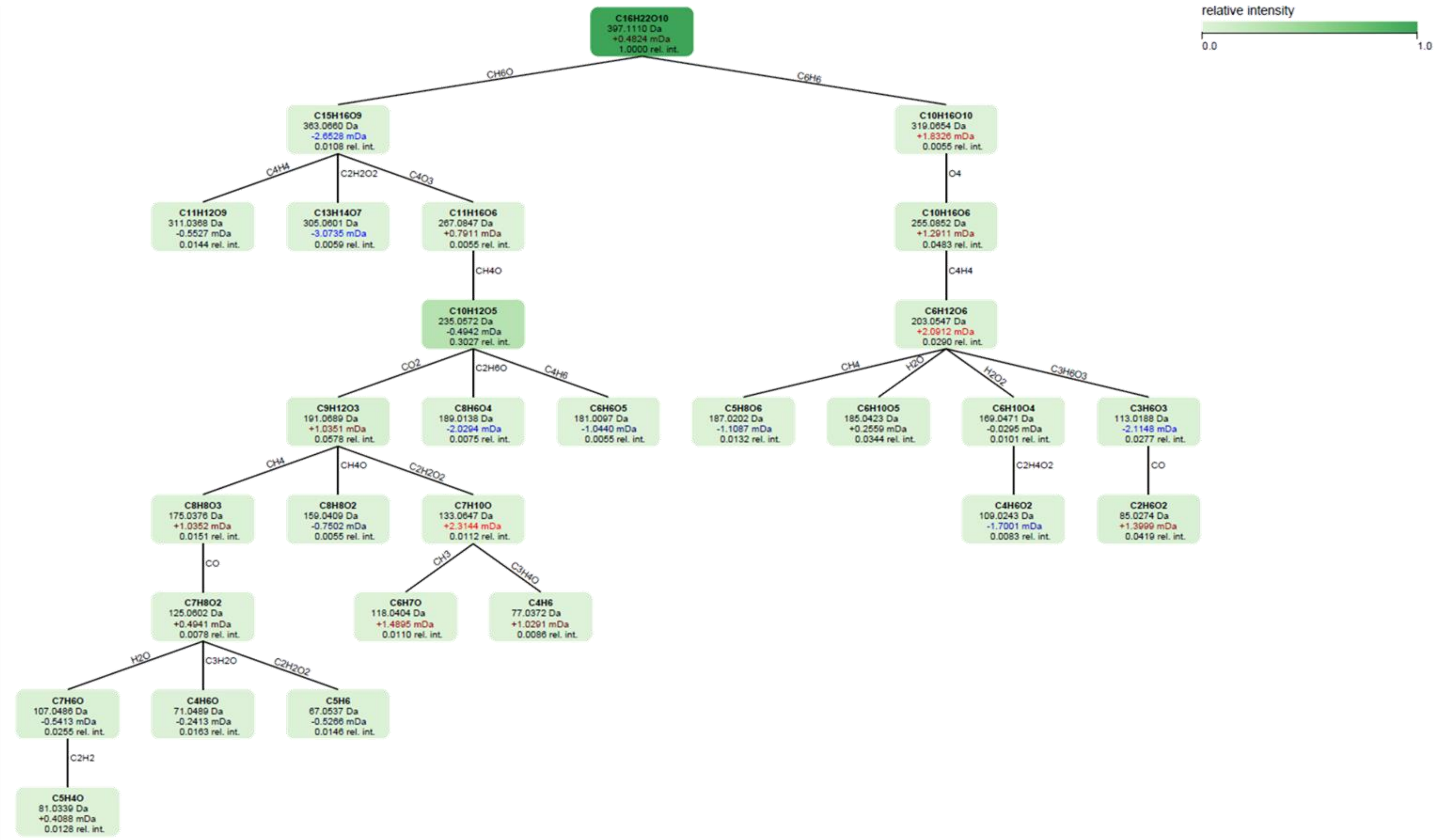

#10 p-Coumaroyl quinic acid

1) MS/MS spectrum

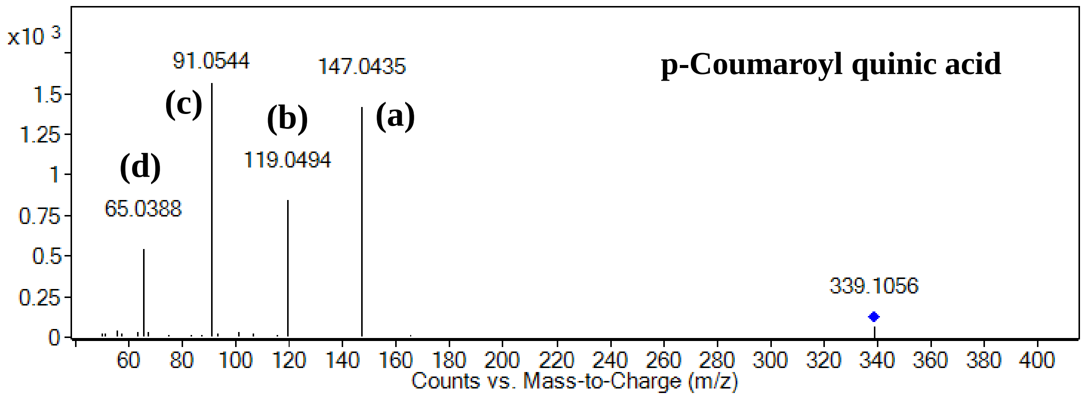

2) MS/MS fragment table

	Mass	Intensity	Weight(%)	No. of candid.	Best score
1	147.0435	1415.64	51.1	3	97.5
2	91.0544	1559.70	21.6	3	76.4
3	119.0494	841.28	19.9	1	97.5
4	65.0388	541.43	3.8	4	43.3
5	165.0532	13.48	0.6	1	93.1
6	147.0261	14.84	0.5	2	69.3
7	101.0390	28.41	0.5	1	78.3
8	67.0541	31.50	0.2	5	53.3
9	115.0535	10.40	0.2	1	75.0
10	55.0533	40.63	0.2	6	49.0

3) Fragmentation pathway

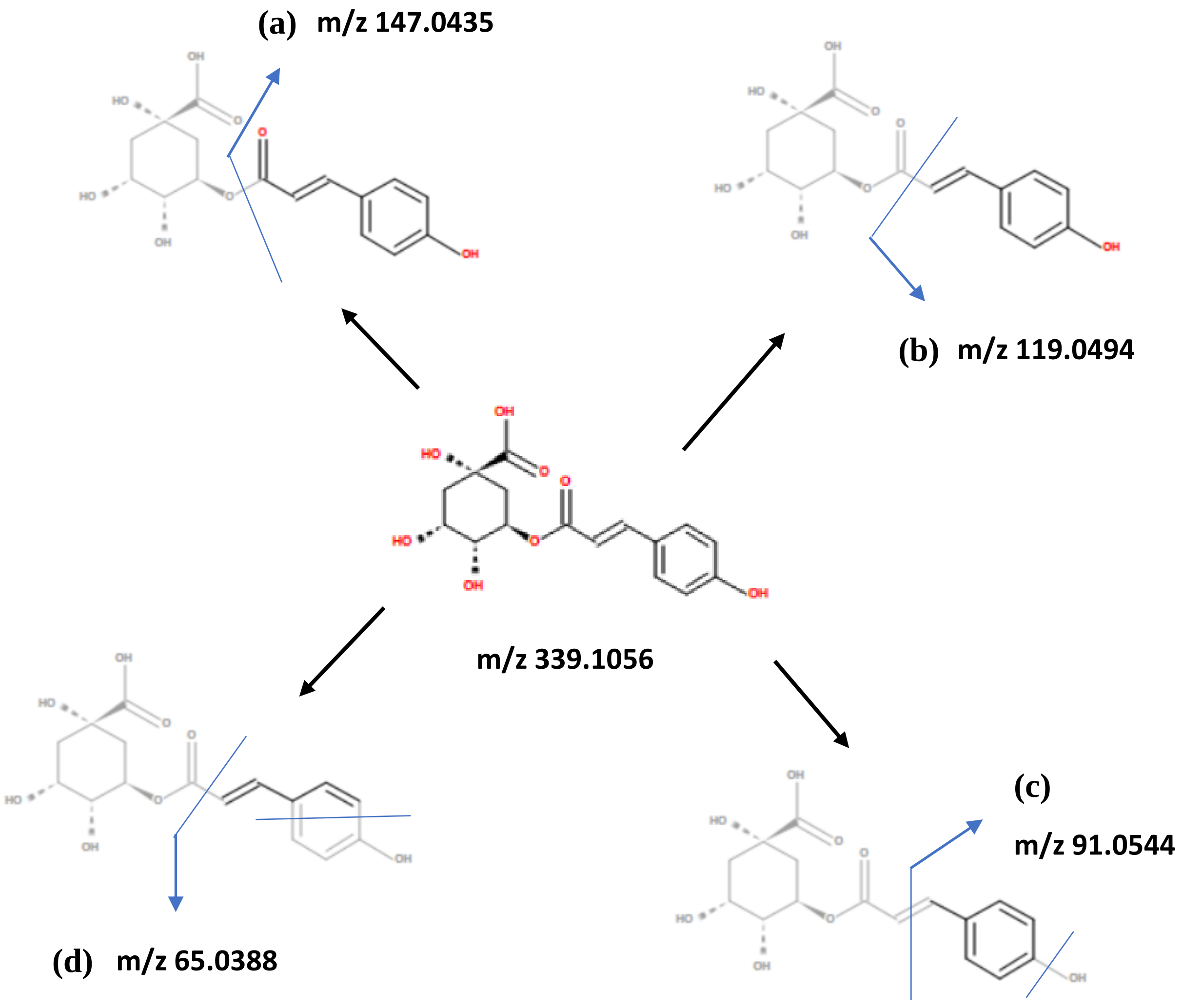

#11 Tarennoside

1) MS/MS spectrum

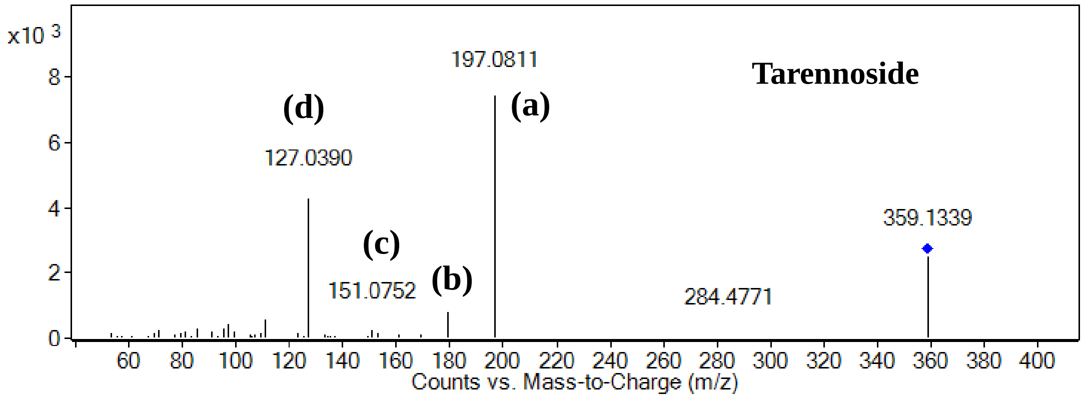

2) MS/MS fragment table

	Mass	Intensity	Weight(%)	No. of candid.	Best score
1	197.0811	7431.44	66.1	11	99.4
2	127.0390	4263.11	15.7	7	91.1
3	179.0703	782.39	5.7	10	98.5
4	111.0807	568.68	1.6	10	94.0
5	151.0752	217.86	1.1	16	97.6
6	151.0379	205.84	1.1	10	78.0
7	97.0285	430.85	0.9	7	85.4
8	153.0552	122.28	0.7	16	88.3
9	123.0439	159.98	0.6	10	83.3
10	95.0496	266.00	0.6	7	84.8

3) Fragmentation pathway

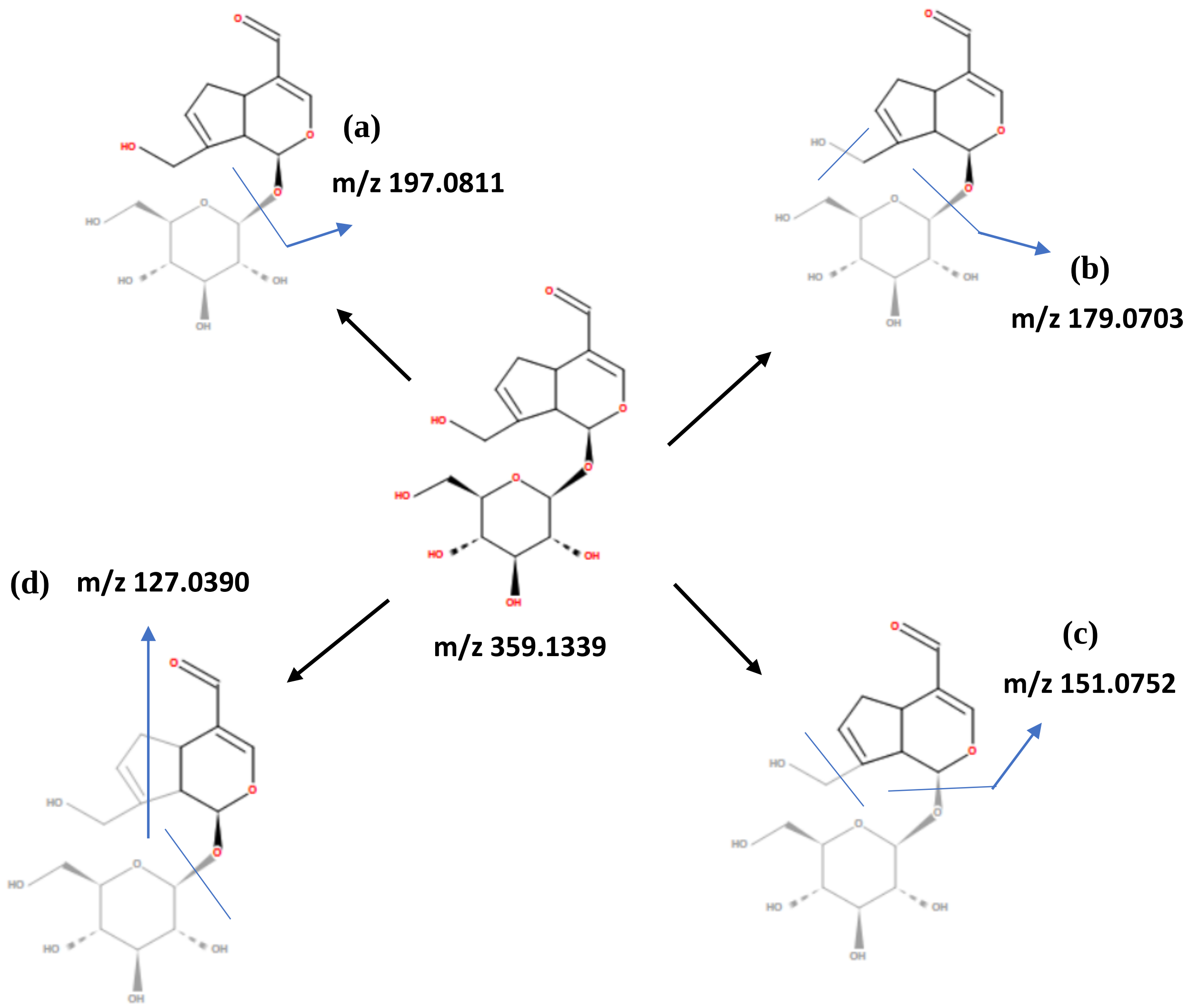

#12 Asp-Arg-Pro

1) MS/MS spectrum

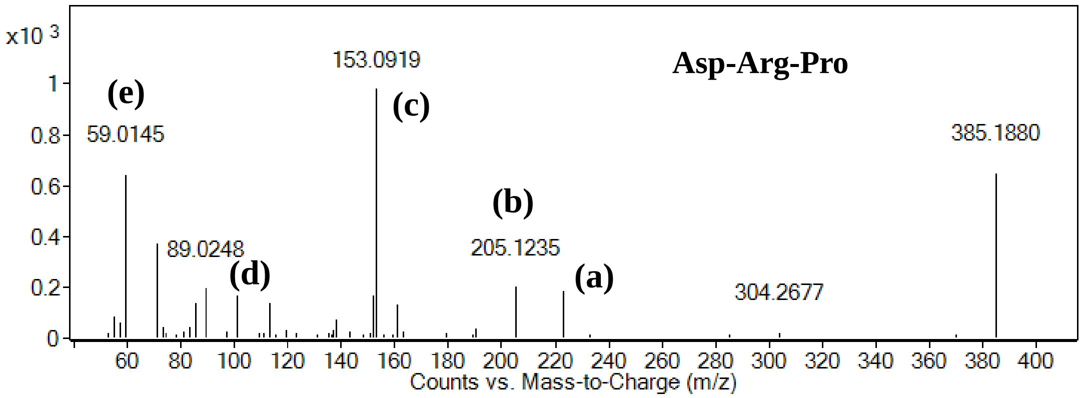

2) MS/MS fragment table

	Mass	Intensity	Weight(%)	No. of candid.	Best score
1	153.0920	2329.08	31.5	13	89.4
2	223.1336	500.30	14.4	29	95.8
3	205.1228	555.84	13.5	2	86.0
4	152.0839	618.29	8.2	12	88.3
5	161.0446	406.40	6.1	0	0.0
6	113.0247	408.56	3.0	0	0.0
7	89.0248	652.38	3.0	0	0.0
8	138.0684	255.25	2.8	8	90.2
9	101.0252	464.57	2.7	1	96.2
10	59.0143	1297.51	2.6	2	98.3

3) Fragmentation pathway

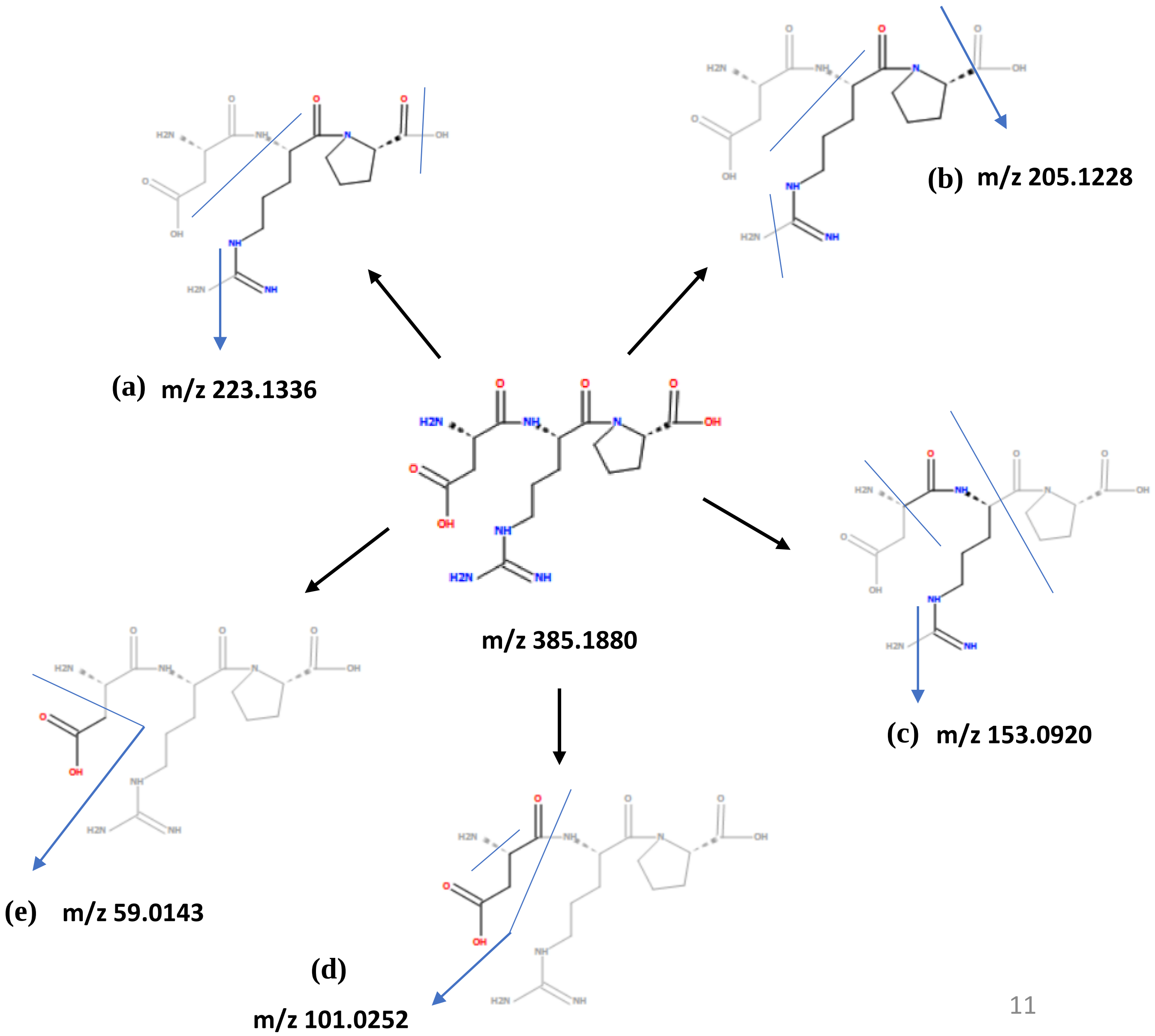

#14 Paeonilactone B

1) MS/MS spectrum

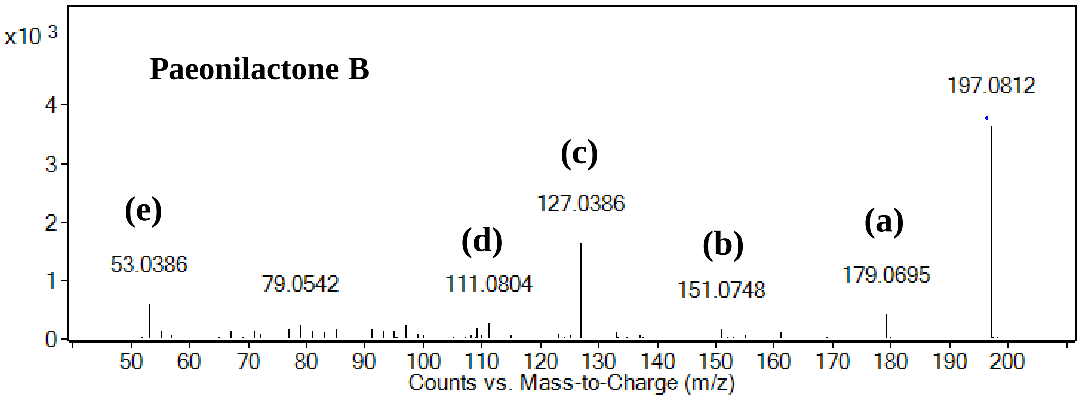

2) MS/MS fragment table

	Mass	Intensity	Weight(%)	No. of candid.	Best score
1	127.0386	1630.81	35.9	12	88.9
2	179.0695	404.52	17.7	4	96.6
3	151.0748	144.83	4.5	27	94.5
4	111.0804	244.46	4.1	10	94.2
5	161.0587	102.78	3.6	3	80.5
6	151.0374	101.77	3.2	18	76.6
7	97.0283	220.69	2.8	12	91.1
8	109.0644	167.00	2.7	10	87.0
9	53.0386	583.67	2.2	7	76.5
10	133.0638	89.44	2.2	2	76.1

3) Fragmentation pathway

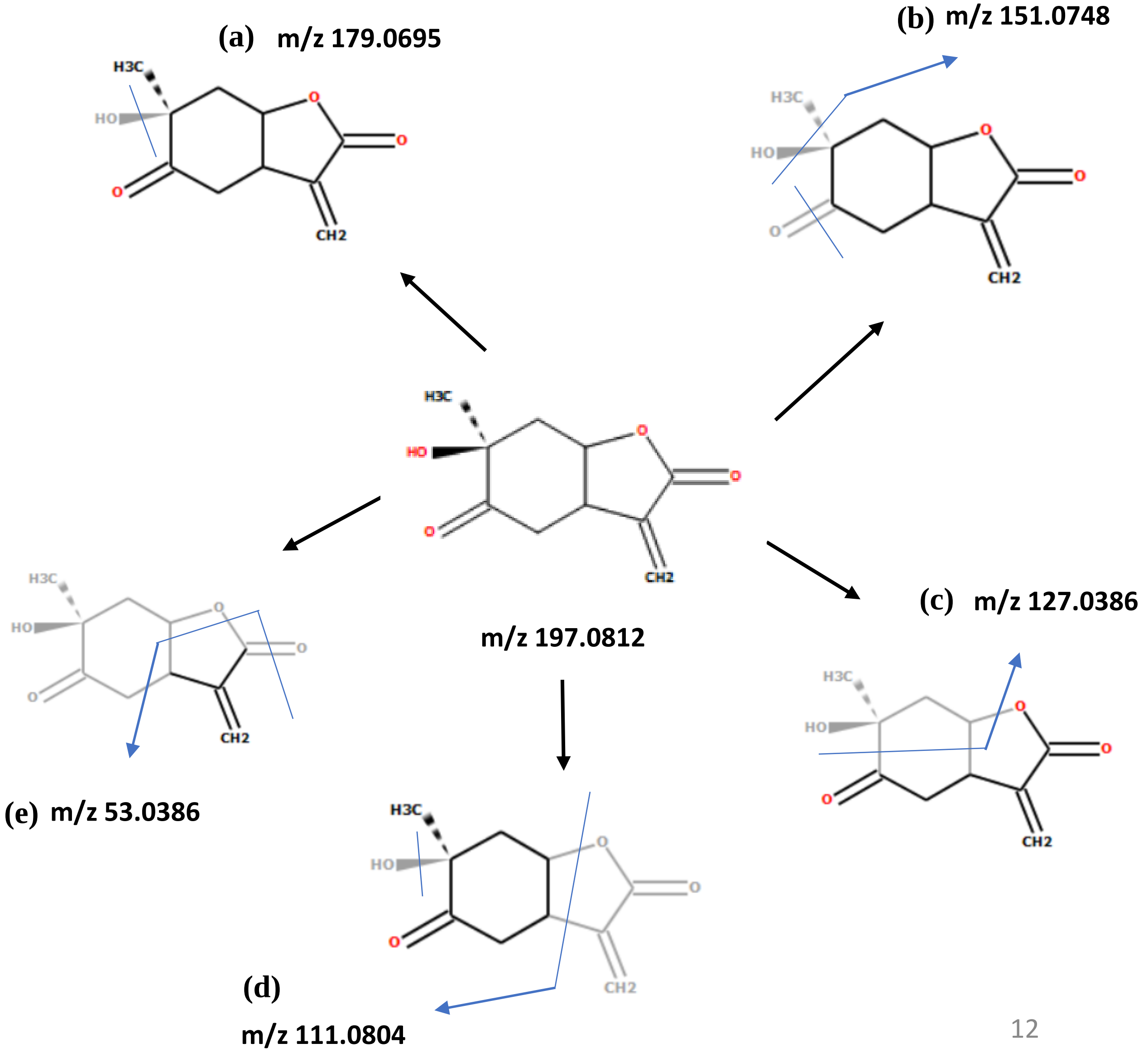

#15 Thr-Gln-Trp

1) MS/MS spectrum

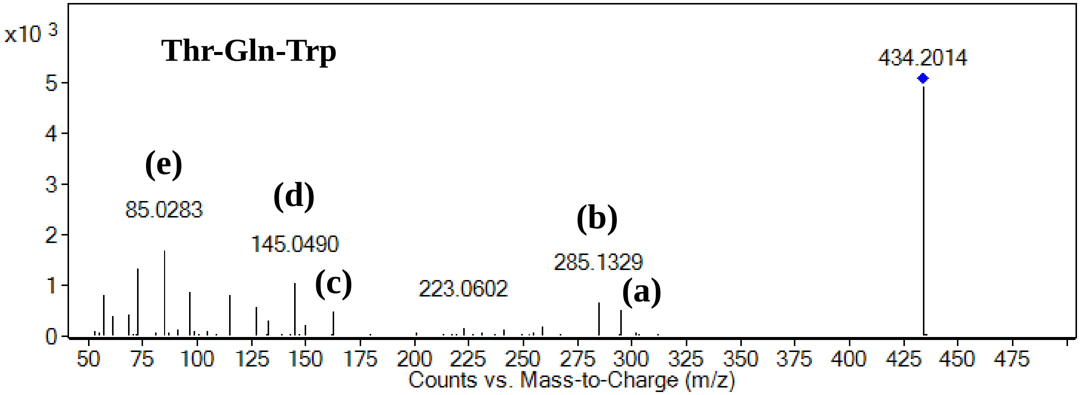

2) MS/MS fragment table

	Mass	Intensity	Weight(%)	No. of candid.	Best score
1	285.1329	659.21	22.4	22	93.1
2	295.1024	491.17	17.8	4	73.2
3	145.0490	1016.82	8.9	2	26.9
4	163.0593	474.71	5.3	3	32.1
5	85.0283	1684.16	5.1	2	94.2
6	259.0805	162.78	4.6	0	0.0
7	115.0390	792.87	4.4	1	73.6
8	127.0390	561.50	3.8	1	81.7
9	97.0284	849.54	3.3	2	94.2
10	241.0701	124.26	3.0	3	86.0

3) Fragmentation pathway

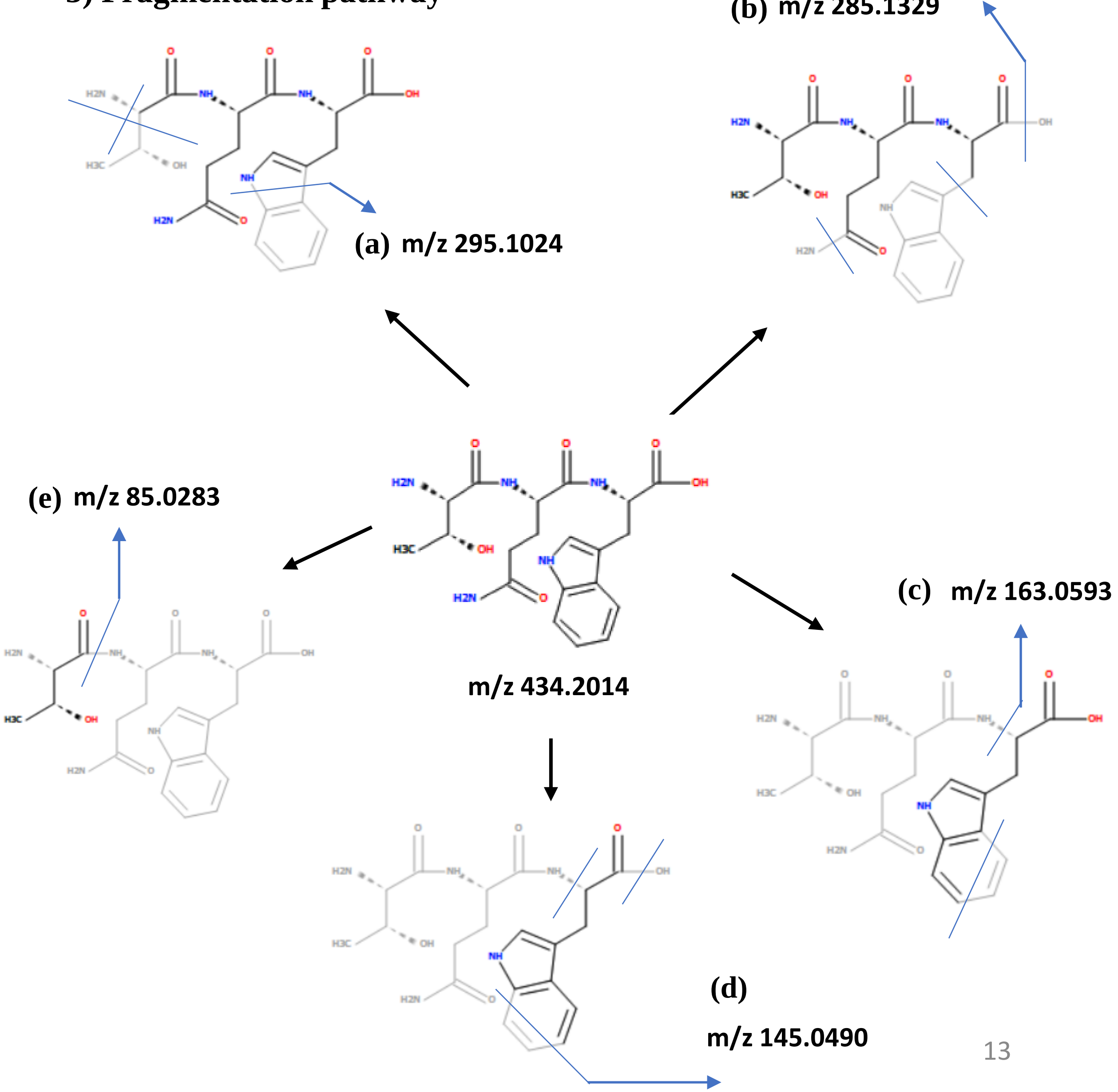

#16 Pro-Trp-Asp

Pro-Trp-Asp
$[M+NH_4]^+$

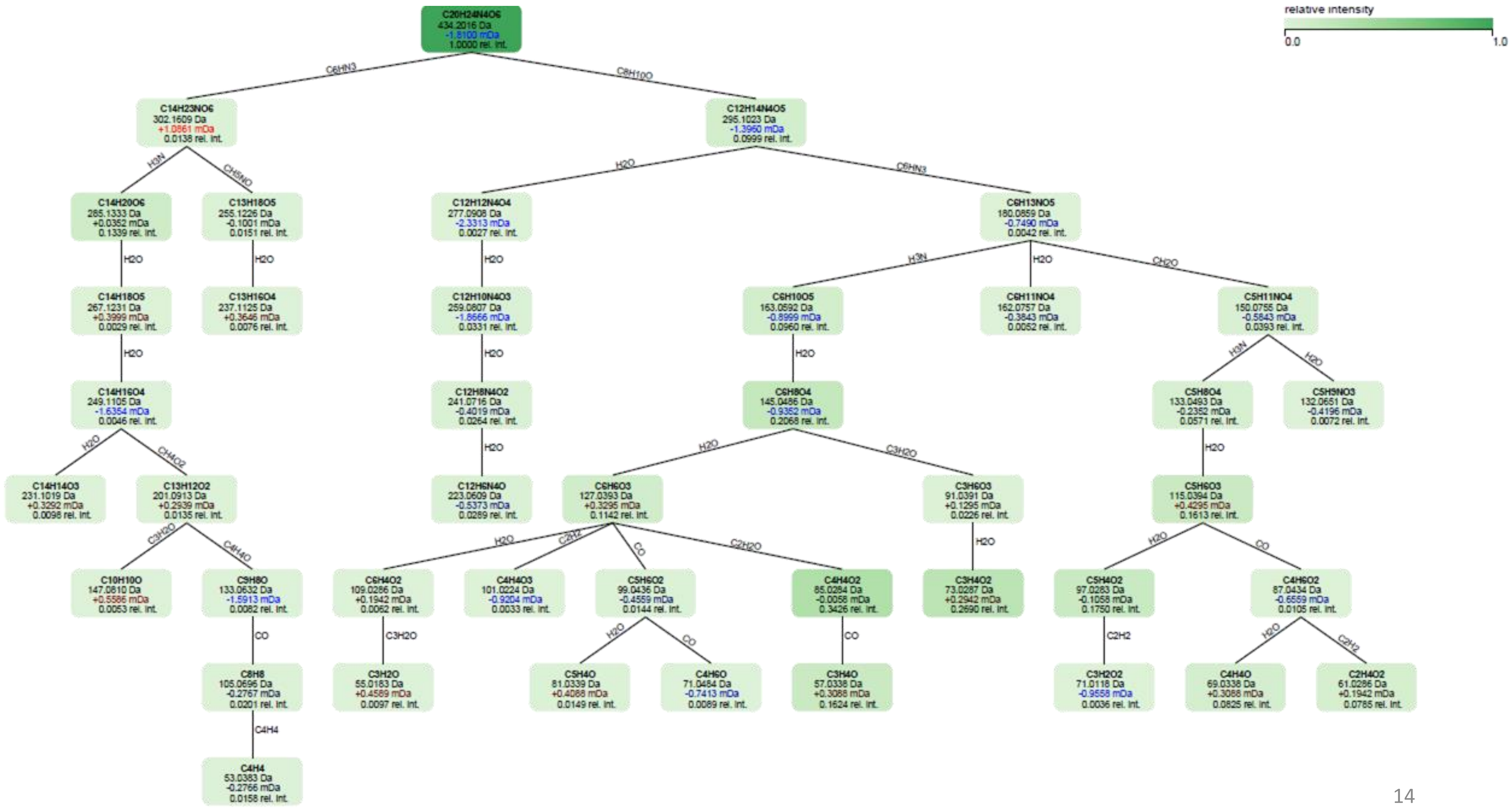

#17 Asp-Glu-His

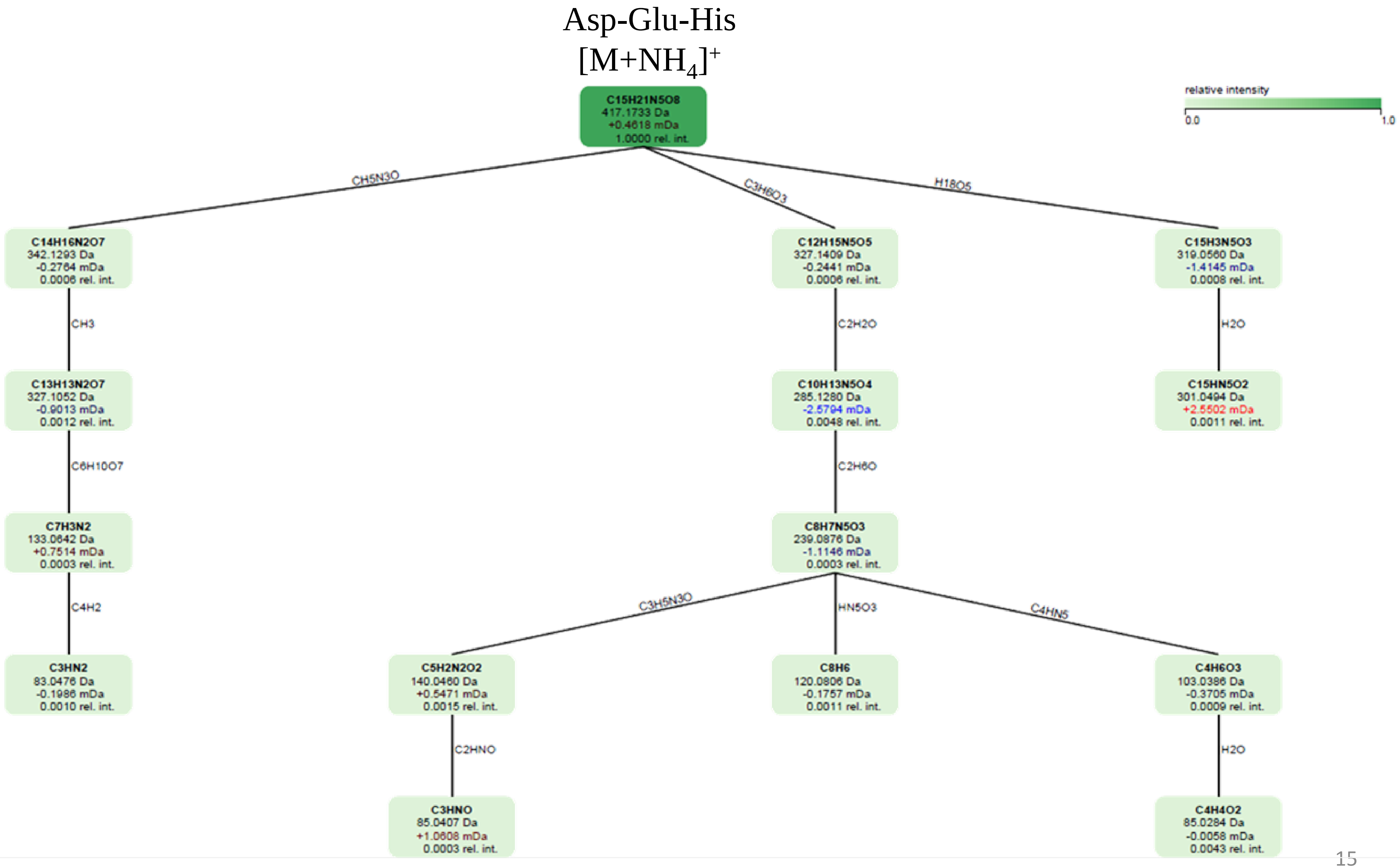

#18 2-Phenylaminoadenosine

1) MS/MS spectrum

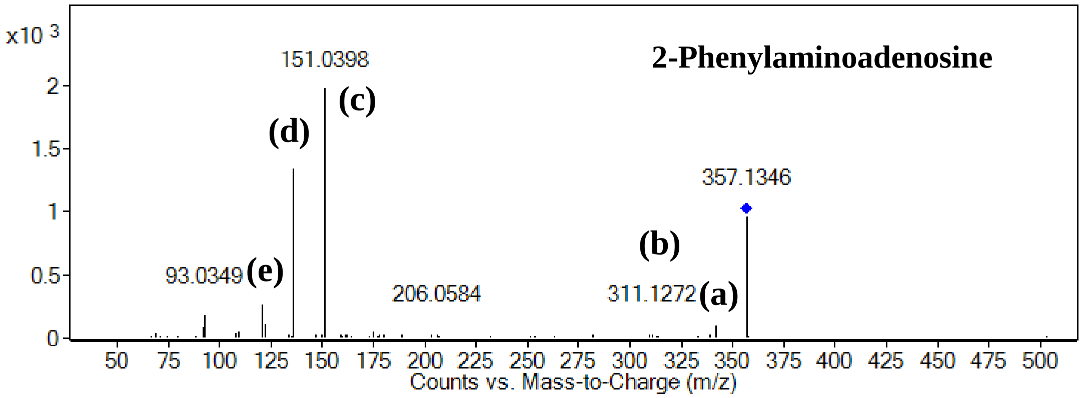

2) MS/MS fragment table

	Mass	Intensity	Weight(%)	No. of candid.	Best score
1	151.0402	2414.82	42.1	3	70.9
2	136.0164	1561.92	22.1	1	63.3
3	342.1095	192.63	17.2	1	82.1
4	311.1260	35.48	2.6	6	97.6
5	121.0297	205.85	2.3	2	70.1
6	122.0364	128.84	1.5	2	80.6
7	175.0764	46.24	1.1	4	64.6
8	296.1032	15.66	1.0	7	90.6
9	162.0316	46.72	0.9	1	72.8
10	256.0730	18.10	0.9	3	33.9

3) Fragmentation pathway

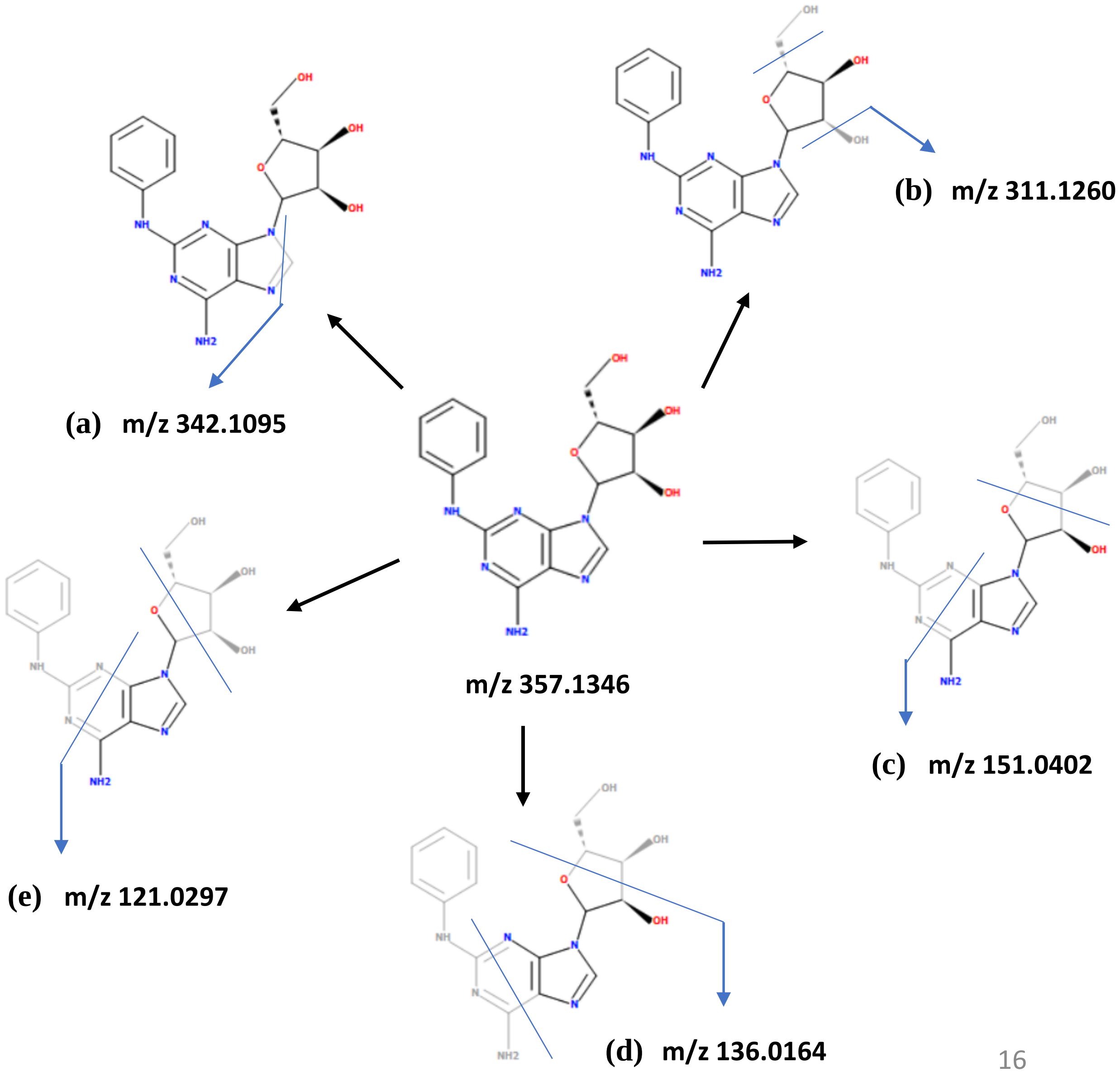

#19 Rutin

1) MS/MS spectrum

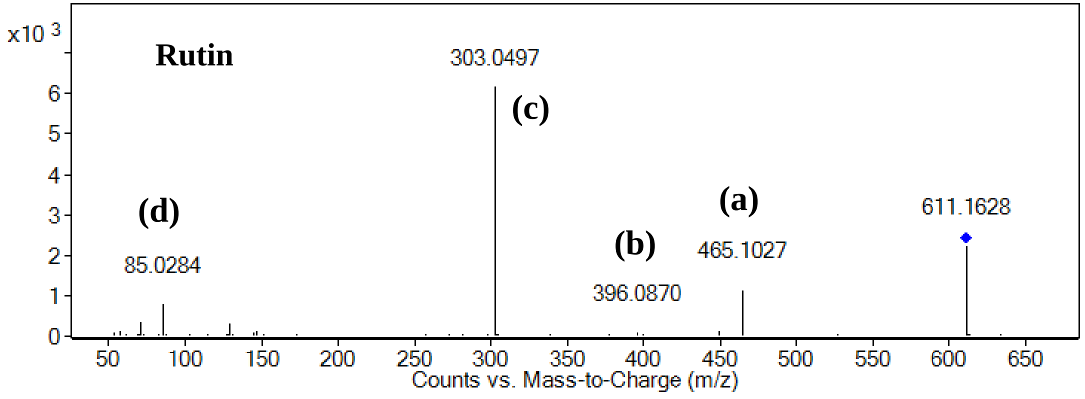

2) MS/MS fragment table

	Mass	Intensity	Weight(%)	No. of candid.	Best score
1	303.0497	6165.74	63.9	3	99.5
2	465.1027	1112.35	27.2	5	99.6
3	449.1082	104.83	2.4	11	99.4
4	396.0870	56.75	1.0	3	74.4
5	85.0284	768.60	0.6	14	78.8
6	129.0545	277.20	0.5	13	94.2
7	305.0784	40.77	0.4	0	0.0
8	303.2721	40.71	0.4	0	0.0
9	303.0097	40.30	0.4	0	0.0
10	339.1370	31.25	0.4	0	0.0

3) Fragmentation pathway

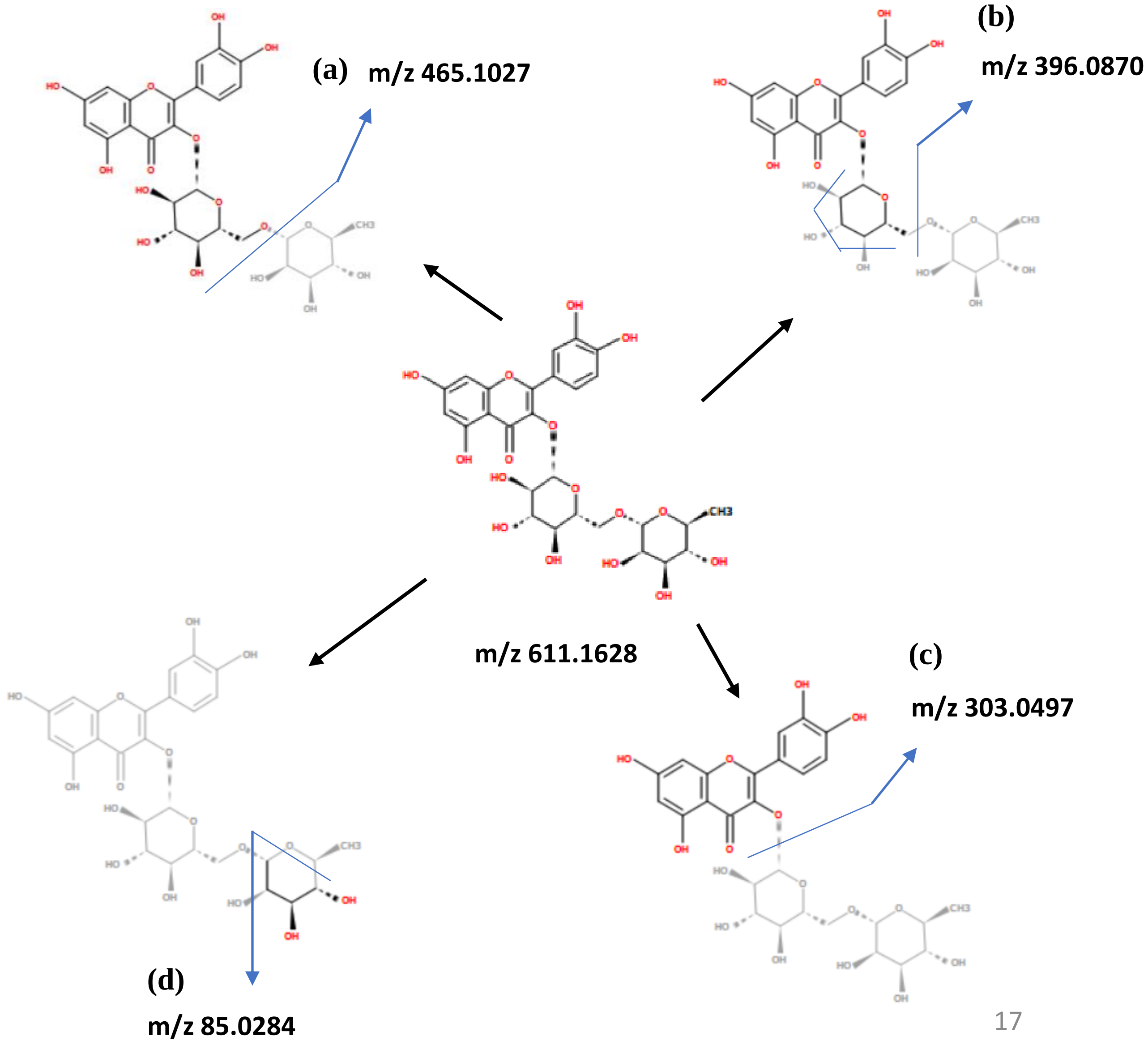

#20 Luteolin 3'-glucuronide

1) MS/MS spectrum

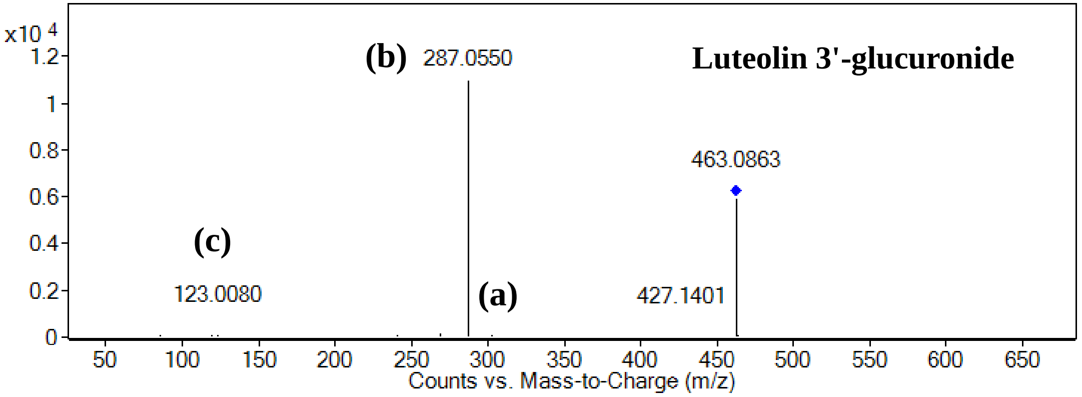

2) MS/MS fragment table

	Mass	Intensity	Weight(%)	No. of candid.	Best score
1	287.0550	10956.83	95.9	4	99.6
2	269.0436	98.05	0.8	4	89.3
3	287.2357	46.57	0.4	0	0.0
4	287.0104	45.55	0.4	0	0.0
5	241.0491	56.54	0.3	3	66.0
6	303.0507	33.86	0.3	5	77.6
7	287.1296	25.56	0.2	0	0.0
8	287.2706	18.98	0.2	0	0.0
9	287.1061	17.75	0.2	4	23.8
10	288.0922	17.10	0.2	0	0.0
11	123.0080	78.43	0.1	1	87.1

3) Fragmentation pathway

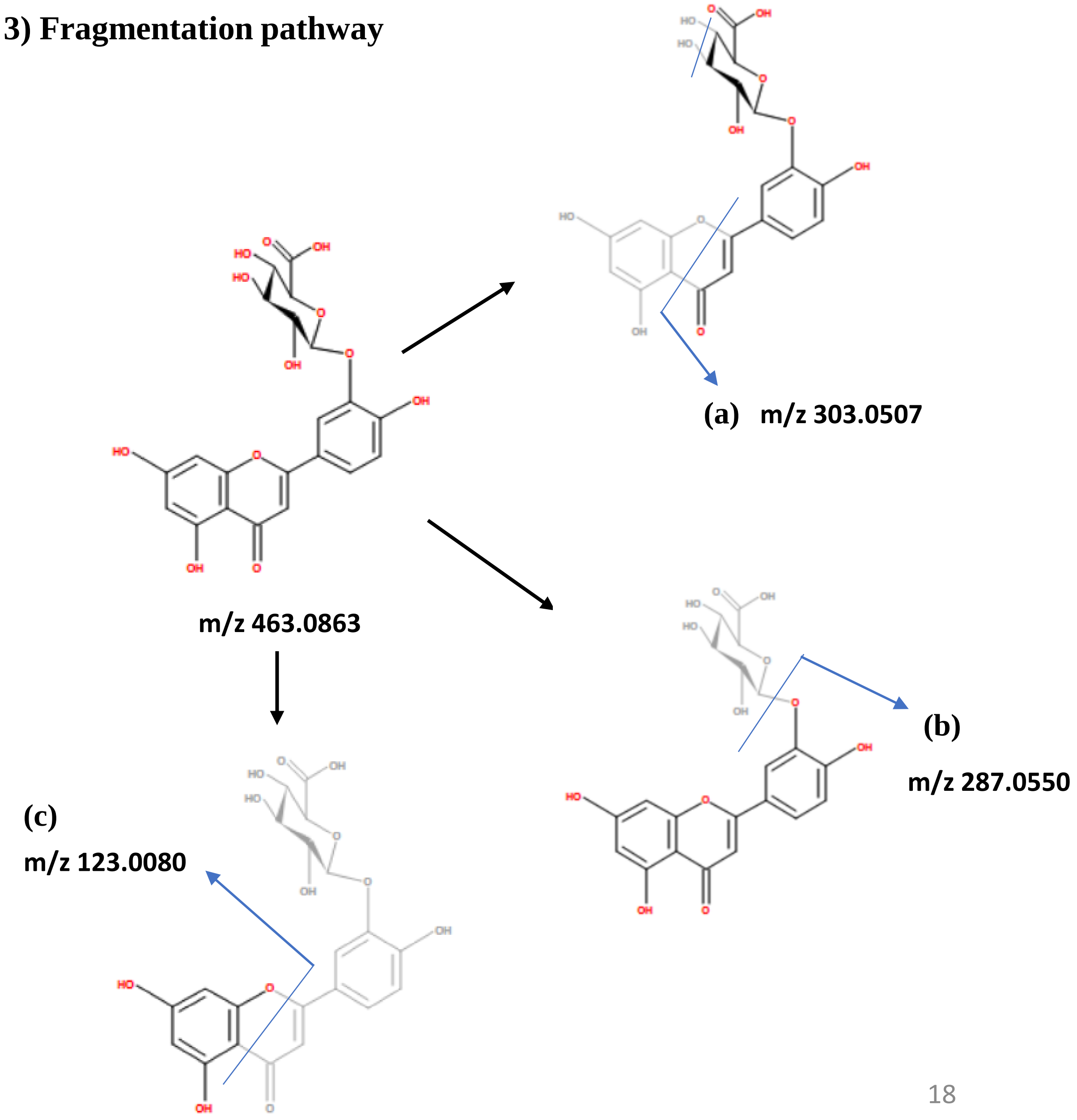

#21 Luteolin-7-O-glucoside

1) MS/MS spectrum

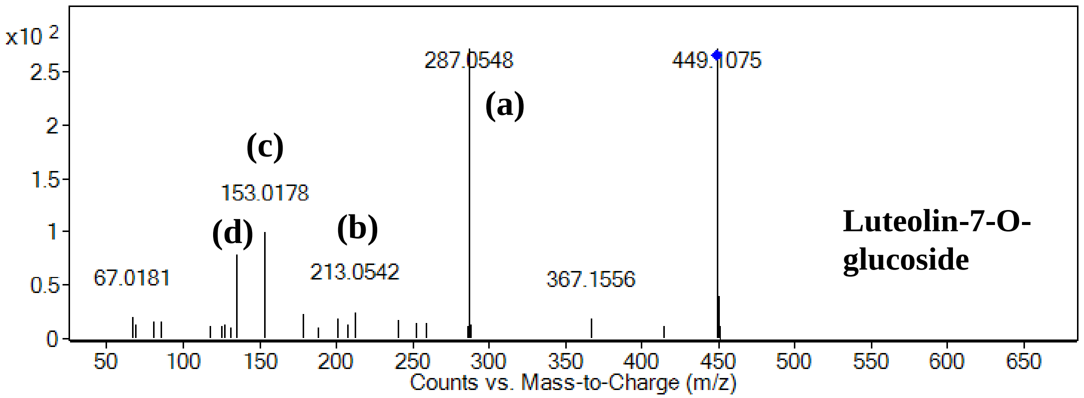

2) MS/MS fragment table

	Mass	Intensity	Weight(%)	No. of candid.	Best score
1	287.0548	11110.35	96.7	4	99.5
2	287.1295	63.08	0.5	0	0.0
3	287.0164	31.72	0.3	0	0.0
4	367.1556	17.59	0.3	0	0.0
5	153.0178	98.94	0.2	2	93.6
6	414.0862	11.78	0.2	0	0.0
7	135.0429	77.86	0.2	5	89.8
8	286.9947	16.62	0.1	0	0.0
9	287.2568	16.35	0.1	0	0.0
10	287.1507	14.46	0.1	0	0.0
11	213.0542	24.23	0.1	3	40.7

3) Fragmentation pathway

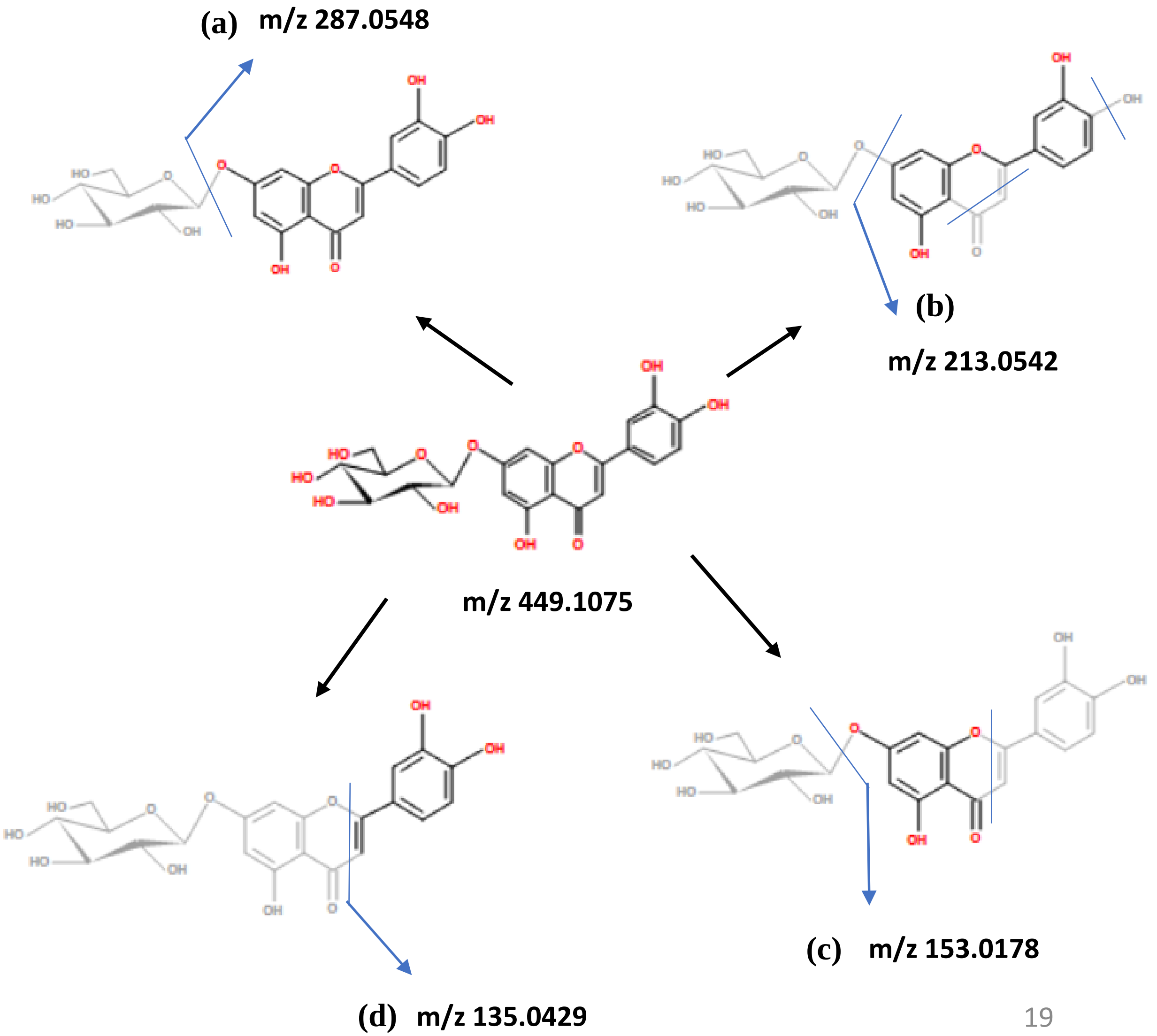

#22 Plumieride

1) MS/MS spectrum

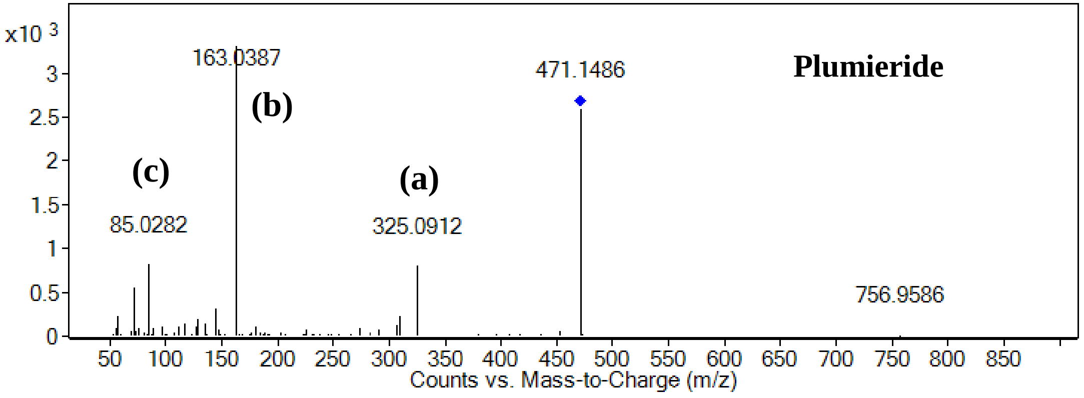

2) MS/MS fragment table

	Mass	Intensity	Weight(%)	No. of candid.	Best score
1	163.0387	1049.69	56.2	12	87.3
2	325.0898	44.83	9.5	41	82.5
3	135.0428	107.89	4.0	8	84.5
4	145.0280	80.95	3.4	4	78.4
5	117.0336	100.69	2.8	2	76.4
6	309.0984	13.58	2.6	44	93.9
7	240.2665	21.88	2.5	0	0.0
8	85.0278	144.29	2.1	22	92.9
9	89.0389	129.60	2.1	0	0.0
10	146.0318	34.80	1.5	4	42.0

3) Fragmentation pathway

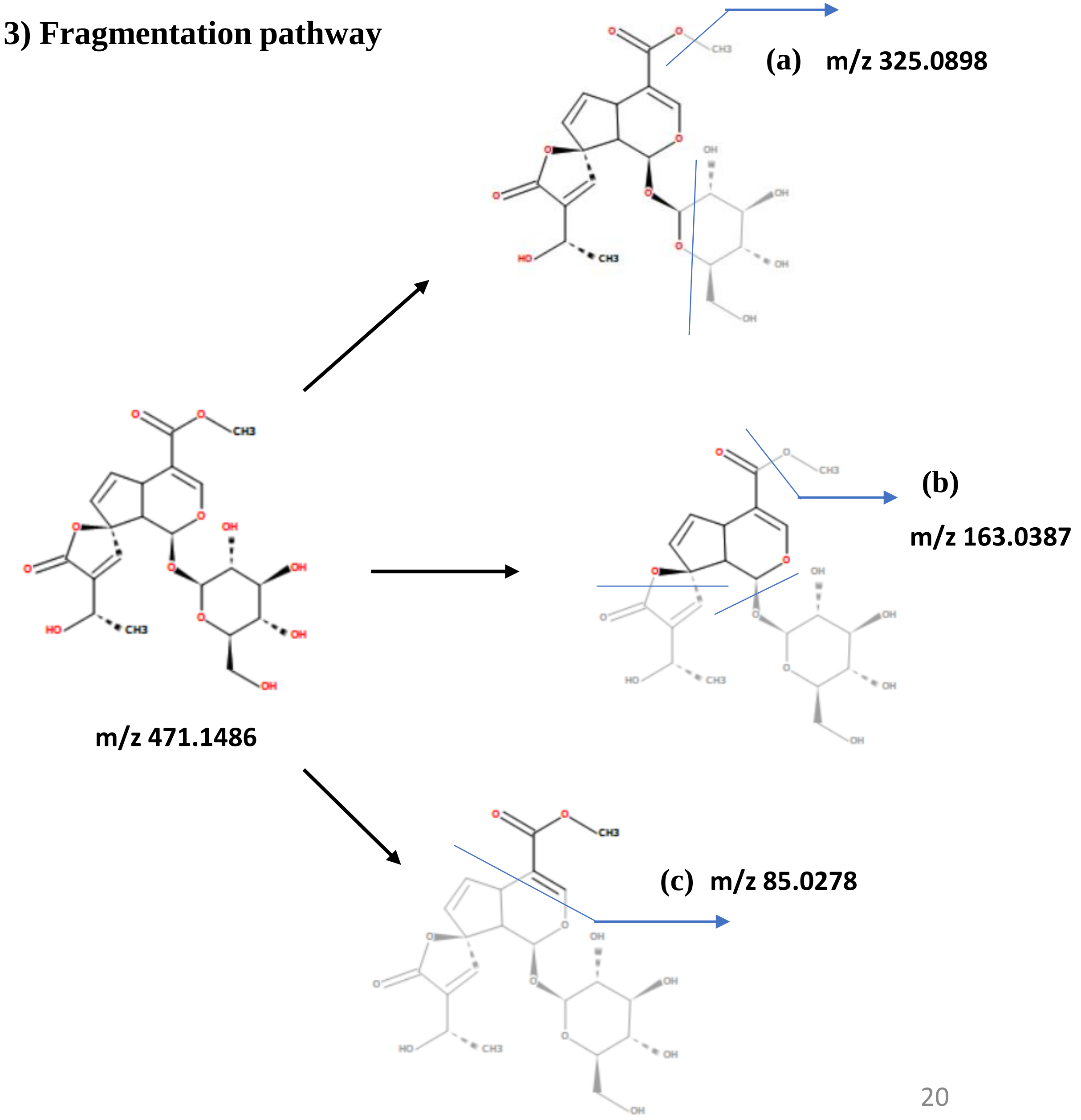

#23 Forsythoside A

Forsythoside A
$[M+NH_4]^+$

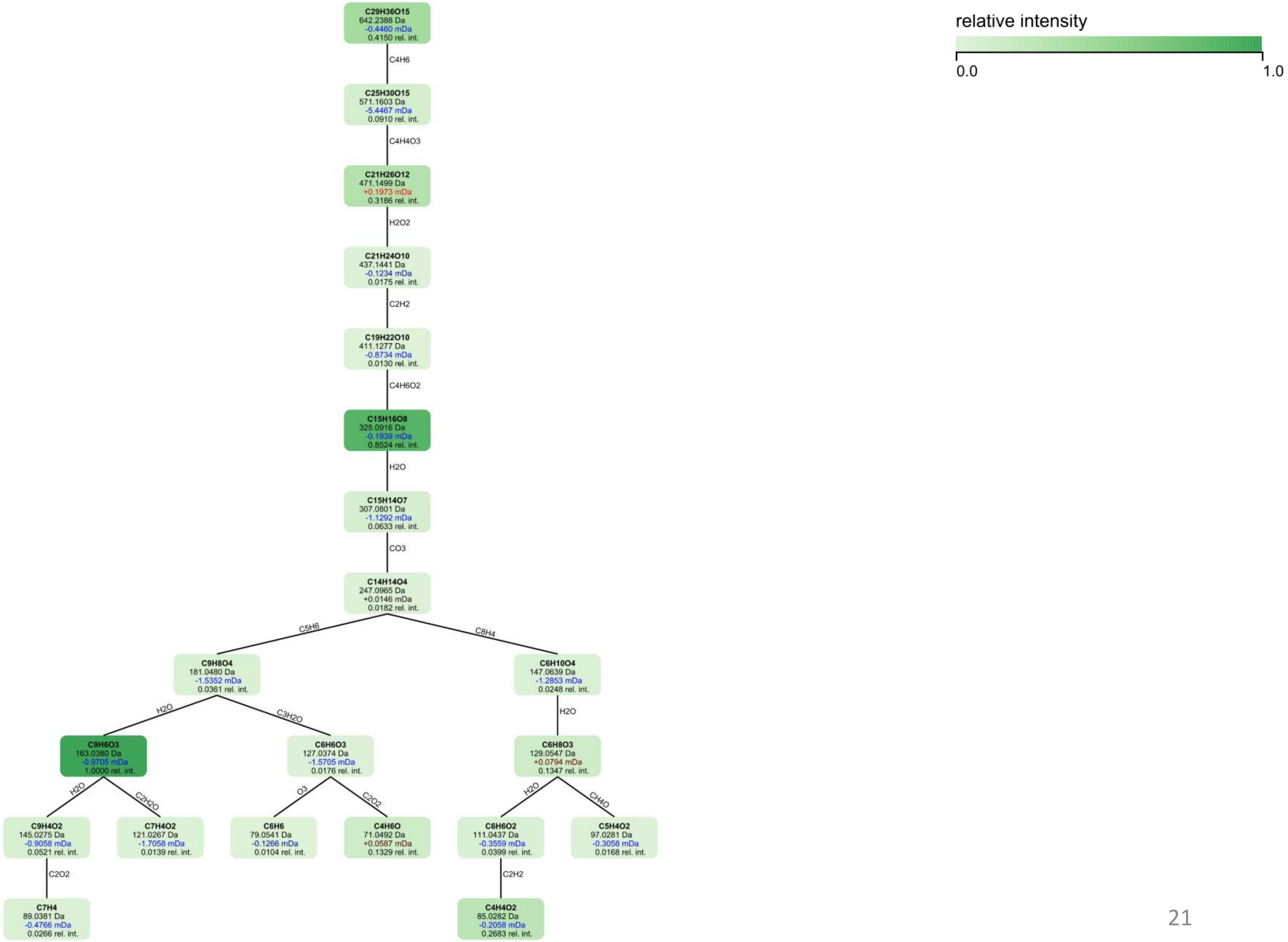

#24 Zwittermicin A

1) MS/MS spectrum

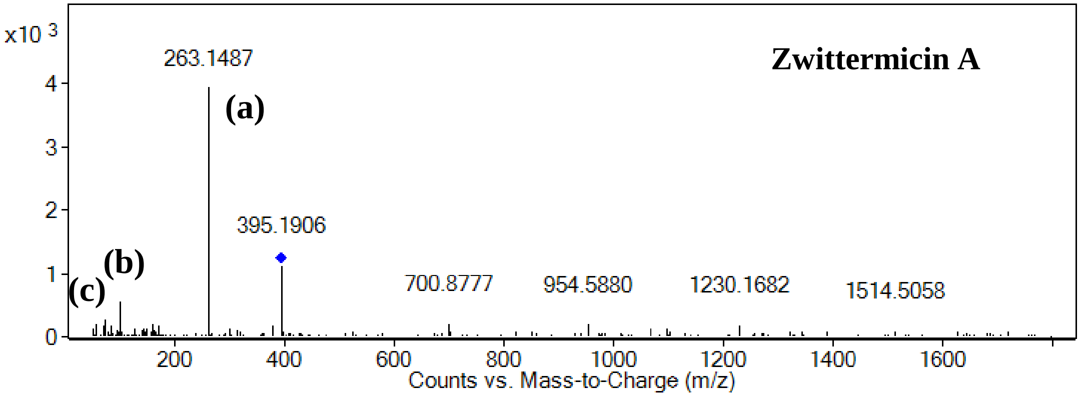

2) MS/MS fragment table

	Mass	Intensity	Weight(%)	No. of candid.	Best score
1	263.1492	1002.95	74.0	38	96.0
2	101.0250	392.33	4.3	2	96.4
3	161.0458	95.65	2.6	0	0.0
4	233.0633	33.98	2.0	0	0.0
5	71.0142	317.71	1.7	6	97.2
6	297.0395	17.69	1.7	0	0.0
7	253.2551	23.01	1.6	0	0.0
8	113.0243	100.04	1.4	3	92.7
9	291.0515	14.12	1.3	0	0.0
10	73.0300	218.01	1.2	6	96.7

3) Fragmentation pathway

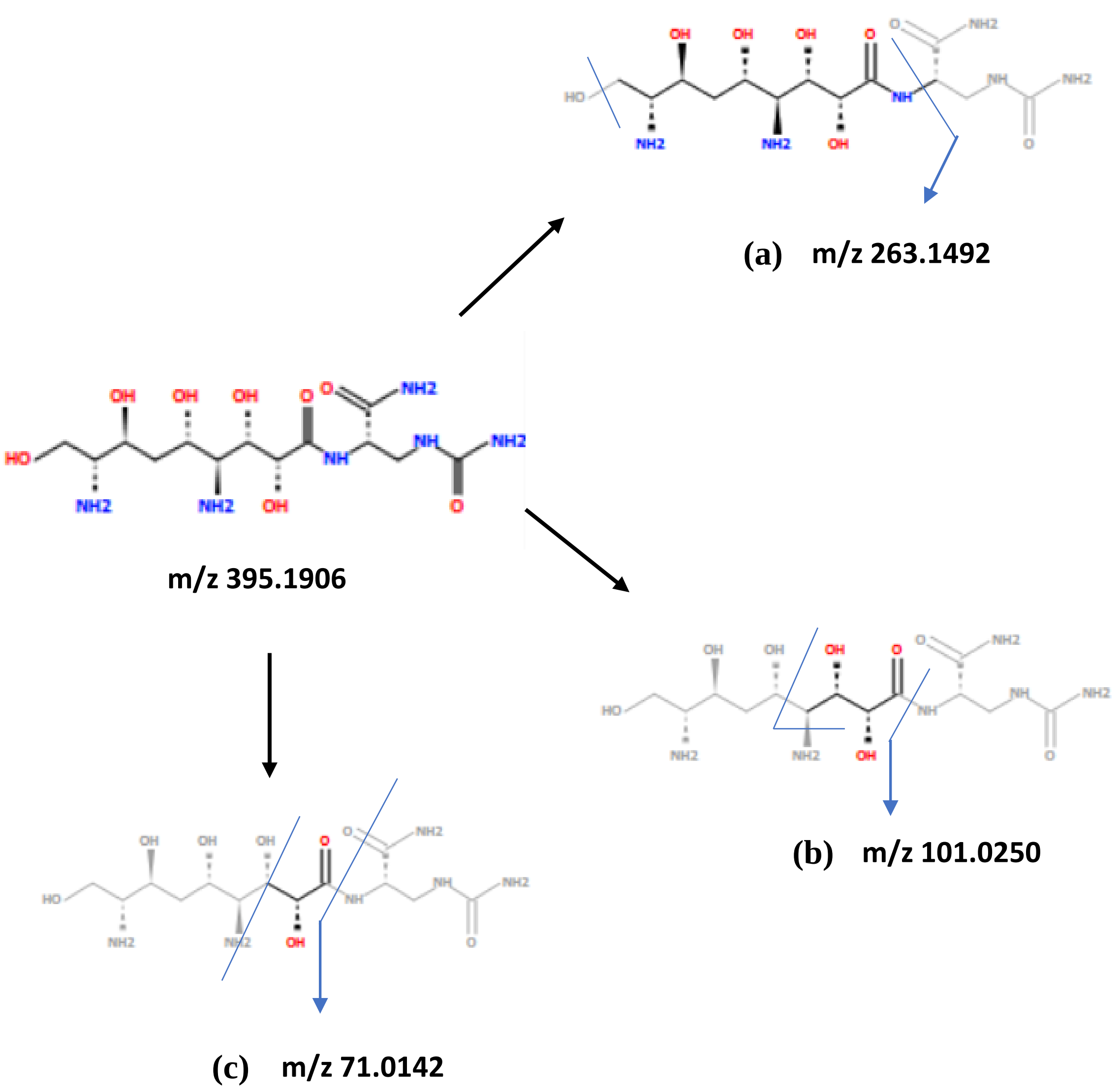

#25 Morachalcone A

1) MS/MS spectrum

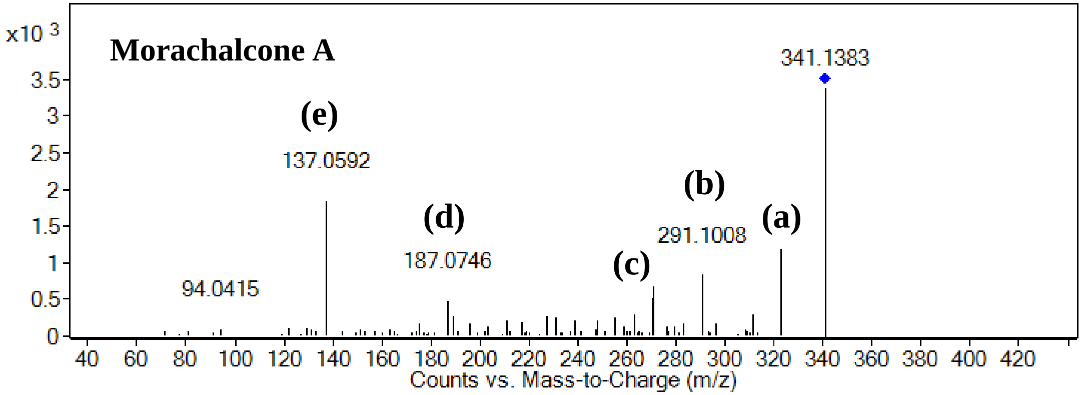

2) MS/MS fragment table

	Mass	Intensity	Weight(%)	No. of candid.	Best score
1	323.1272	1184.94	22.1	2	90.4
2	291.1008	828.28	12.5	2	62.8
3	271.0957	662.82	8.7	4	93.9
4	270.0881	499.22	6.5	2	92.0
5	137.0592	1833.68	6.1	4	98.3
6	311.1274	285.20	4.9	2	95.2
7	263.1075	284.55	3.5	0	0.0
8	187.0746	471.51	2.9	4	79.7
9	255.0643	235.51	2.7	6	54.7
10	227.0699	273.58	2.5	2	34.0

3) Fragmentation pathway

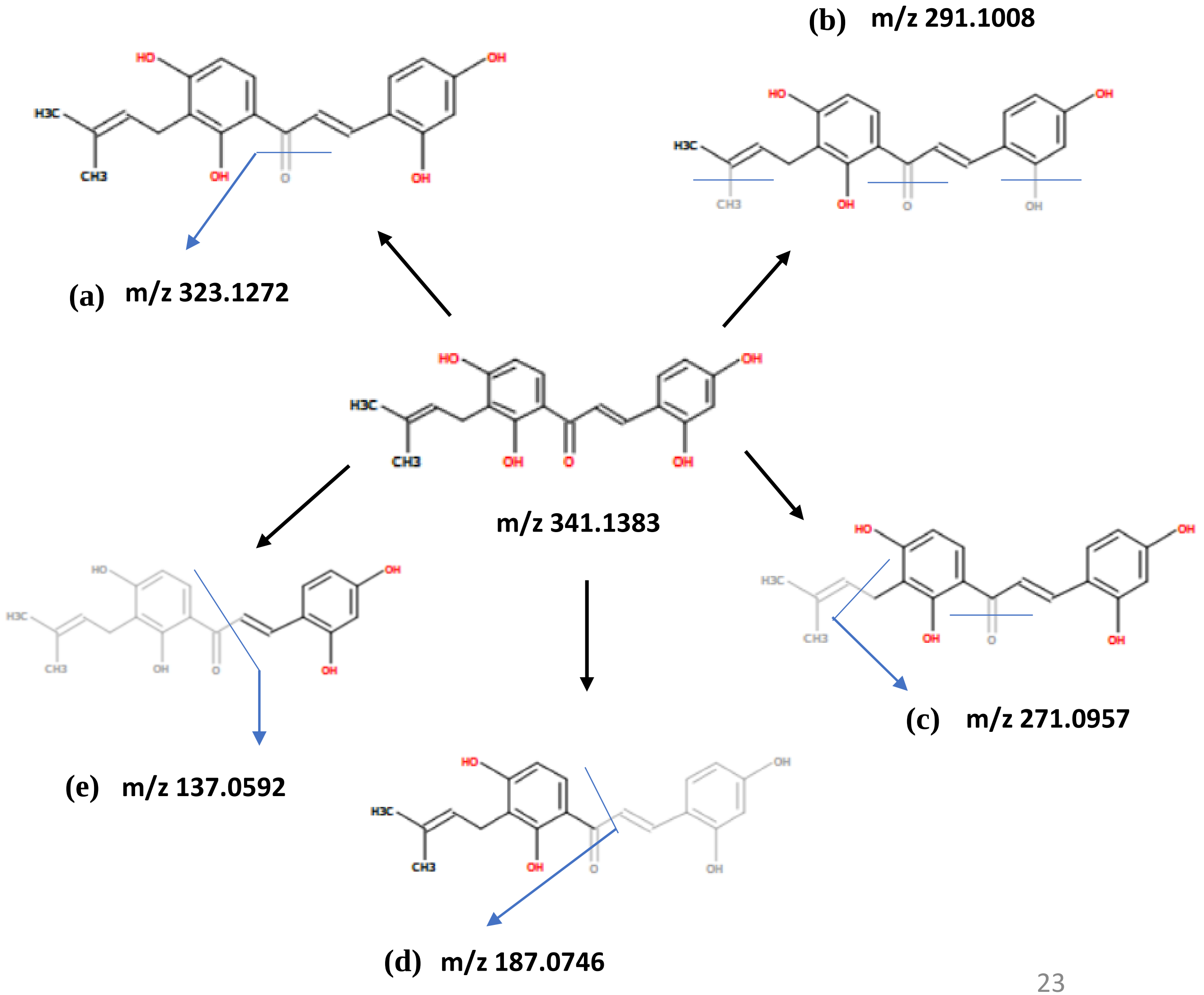

#26 Brusatol

Brusatol
$[M+NH_4]^+$

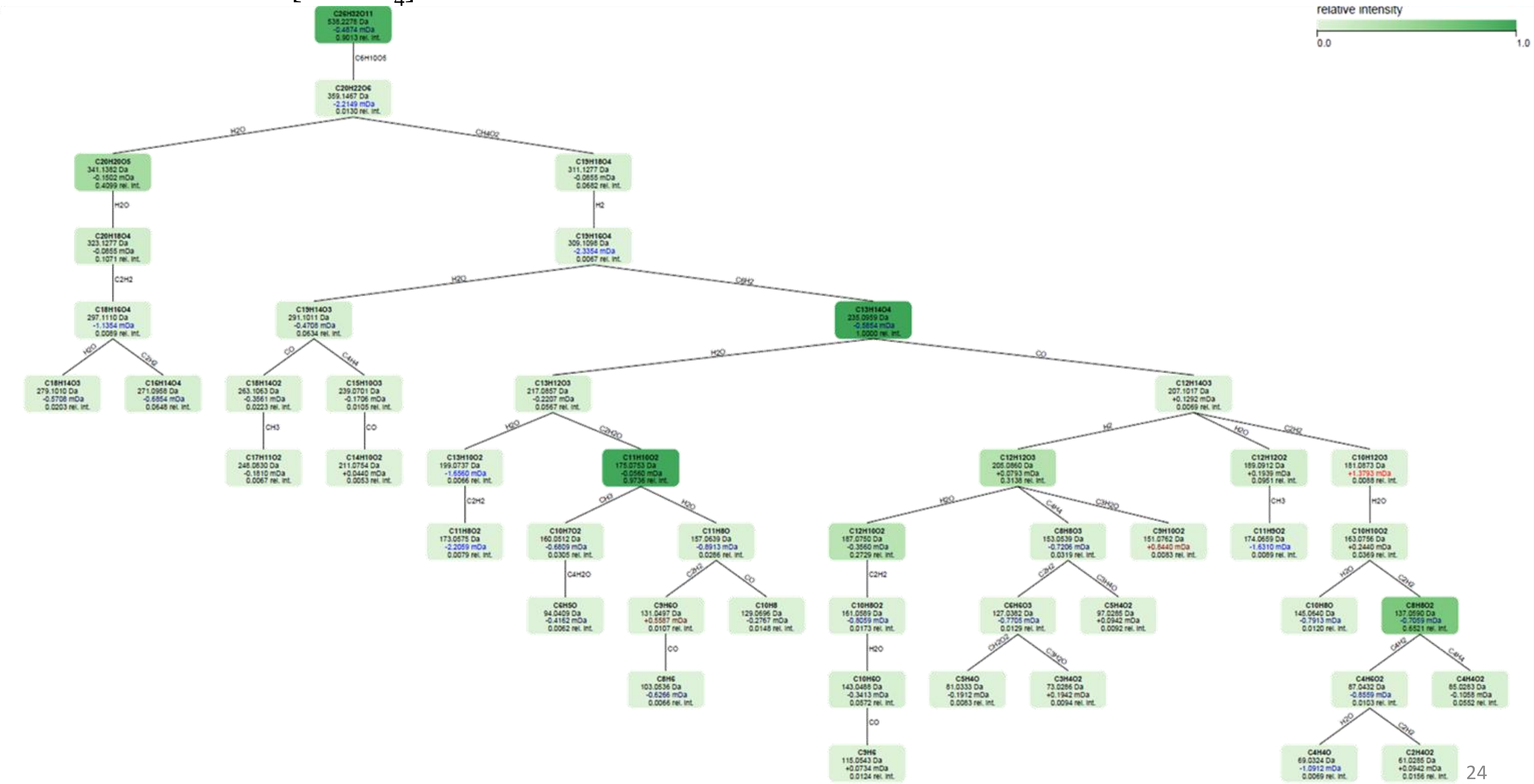

#27 Isofurcatain 7-O-glucoside

1) MS/MS spectrum

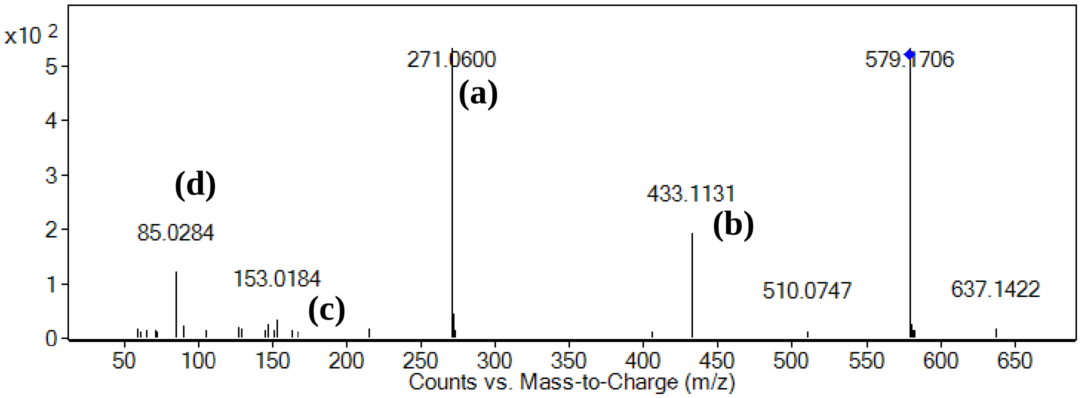

2) MS/MS fragment table

	Mass	Intensity	Weight(%)	No. of candid.	Best score
1	271.0601	12093.64	93.2	3	71.5
2	433.1119	192.06	3.8	10	87.3
3	85.0284	119.53	0.1	7	78.8
4	271.0131	54.63	0.4	0	0.0
5	271.0267	53.14	0.4	0	0.0
6	153.0187	34.16	0.1	1	60.7
7	271.1314	31.05	0.2	0	0.0
8	271.3014	30.34	0.2	0	0.0
9	271.3488	25.93	0.2	0	0.0
10	147.0656	24.12	0.1	8	98.1

3) Fragmentation pathway

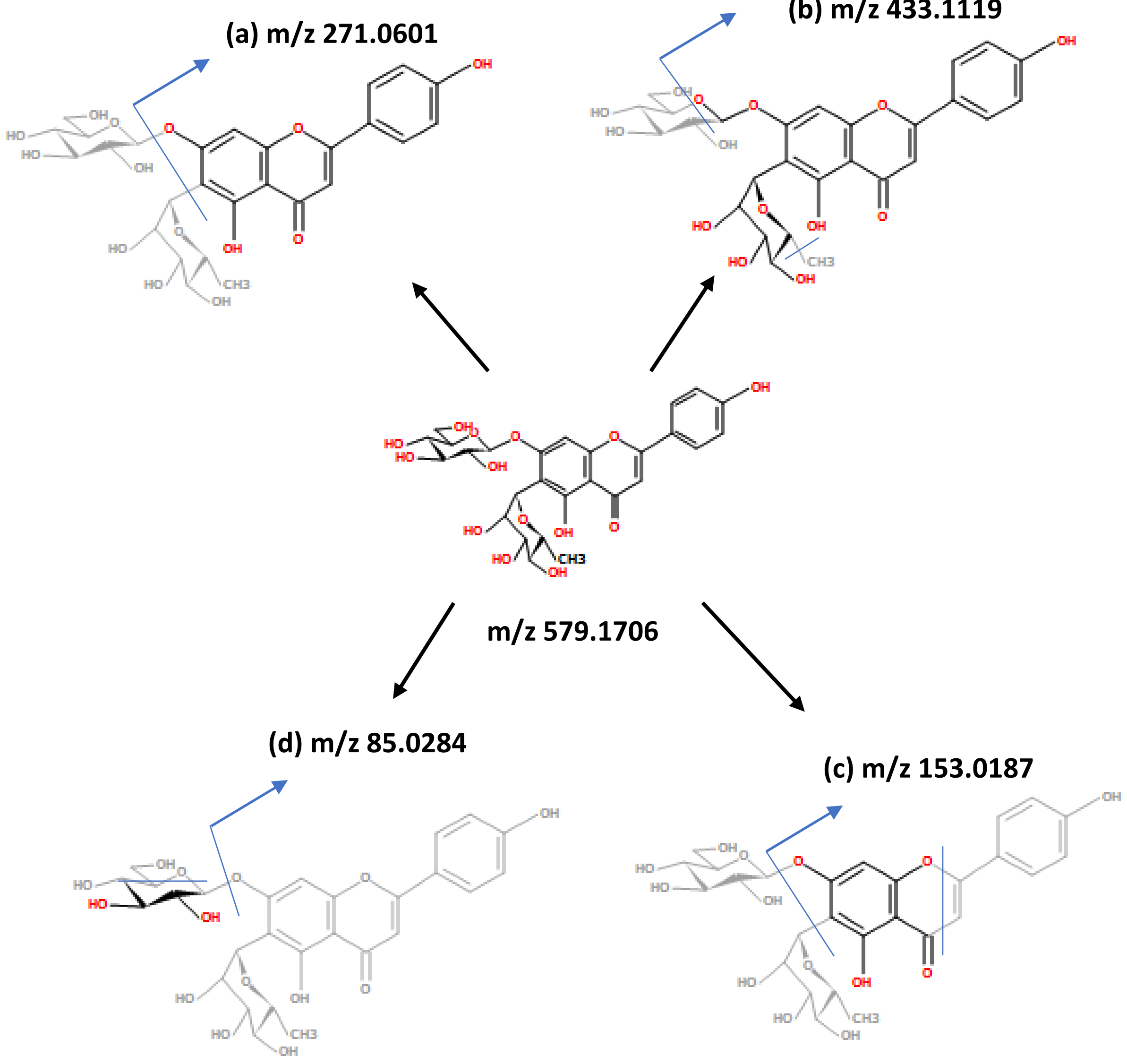

#28 Apigenin 7-(3'',4''-diacetylglucoside)

1) MS/MS spectrum

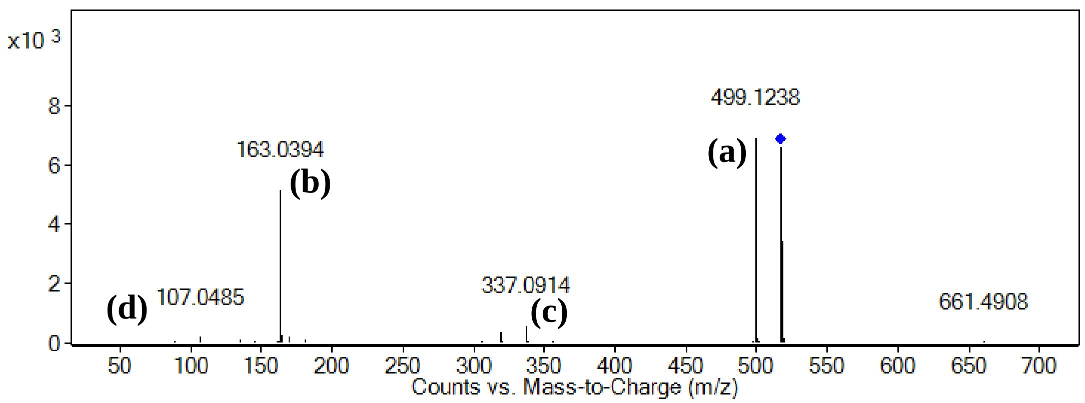

2) MS/MS fragment table

	Mass	Intensity	Weight(%)	No. of candid.	Best score
1	163.0387	13295.19	20.7	4	94.0
2	499.1228	4743.84	69.2	7	97.6
3	145.0280	1198.22	1.5	3	86.8
4	135.0433	947.25	1.0	4	92.3
5	117.0330	584.35	0.5	4	86.5
6	337.0913	365.00	2.4	26	92.3
7	89.0382	329.69	0.2	1	63.4
8	319.0811	283.36	1.7	7	78.8
9	355.1003	119.28	0.9	28	84.8
10	107.0493	88.61	0.1	4	89.3

3) Fragmentation pathway

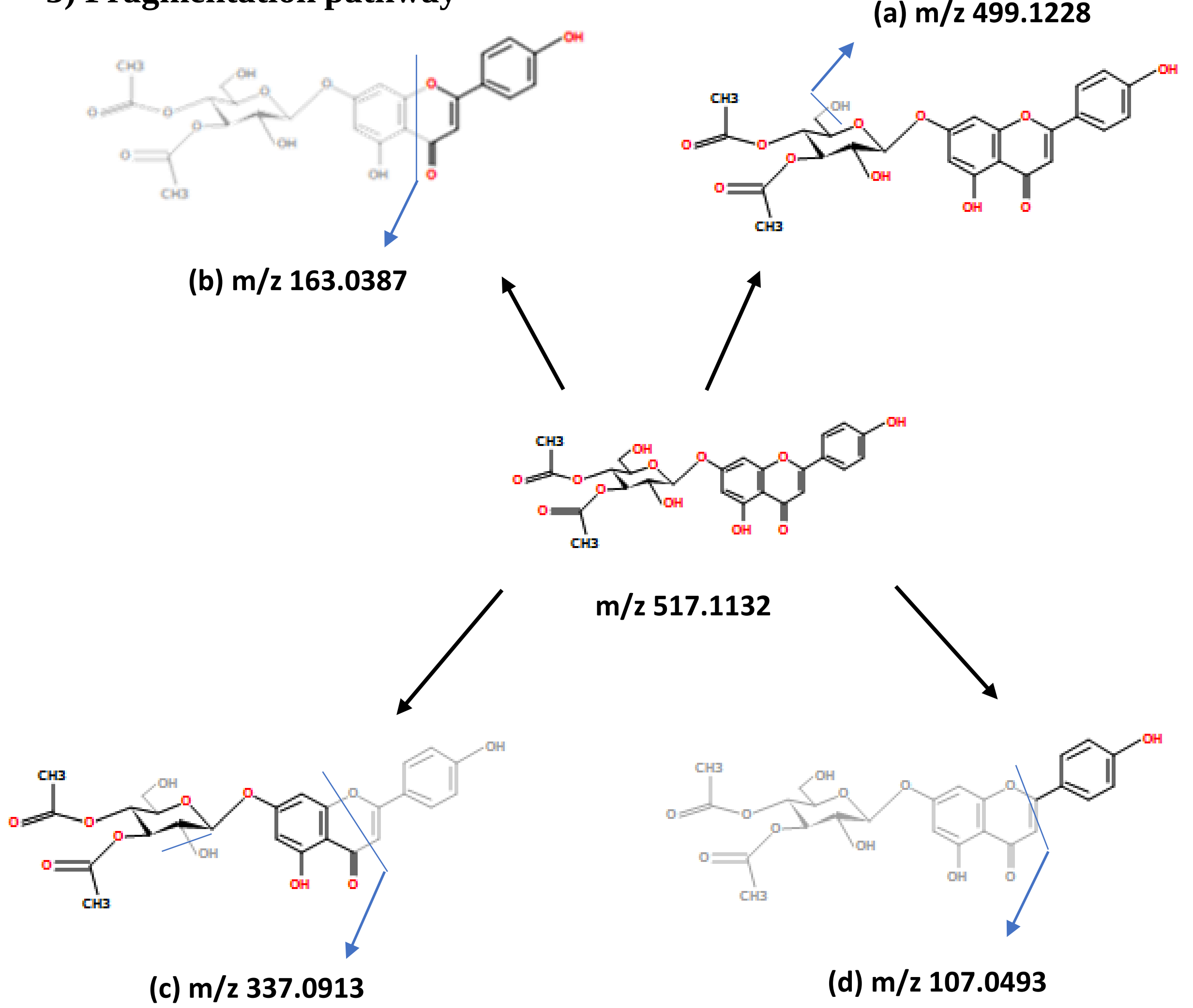

#29 Isovitexin

1) MS/MS spectrum

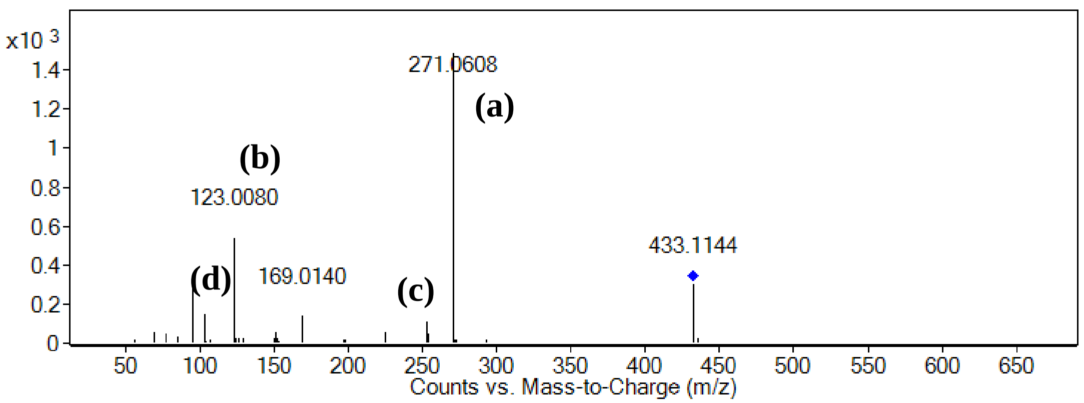

2) MS/MS fragment table

	Mass	Intensity	Weight(%)	No. of candid.	Best score
1	271.0602	3771.00	90.2	4	76.4
2	123.0077	419.98	2.1	1	50.8
3	169.0126	118.43	1.1	0	0.0
4	253.0490	113.17	2.4	4	63.3
5	103.0551	80.89	0.3	2	78.7
6	95.0125	80.42	0.2	4	34.0
7	129.0329	62.93	0.3	3	73.1
8	68.9965	42.09	0.1	4	50.0
9	153.0338	30.72	0.2	0	0.0
10	104.0573	29.65	0.1	2	33.7

3) Fragmentation pathway

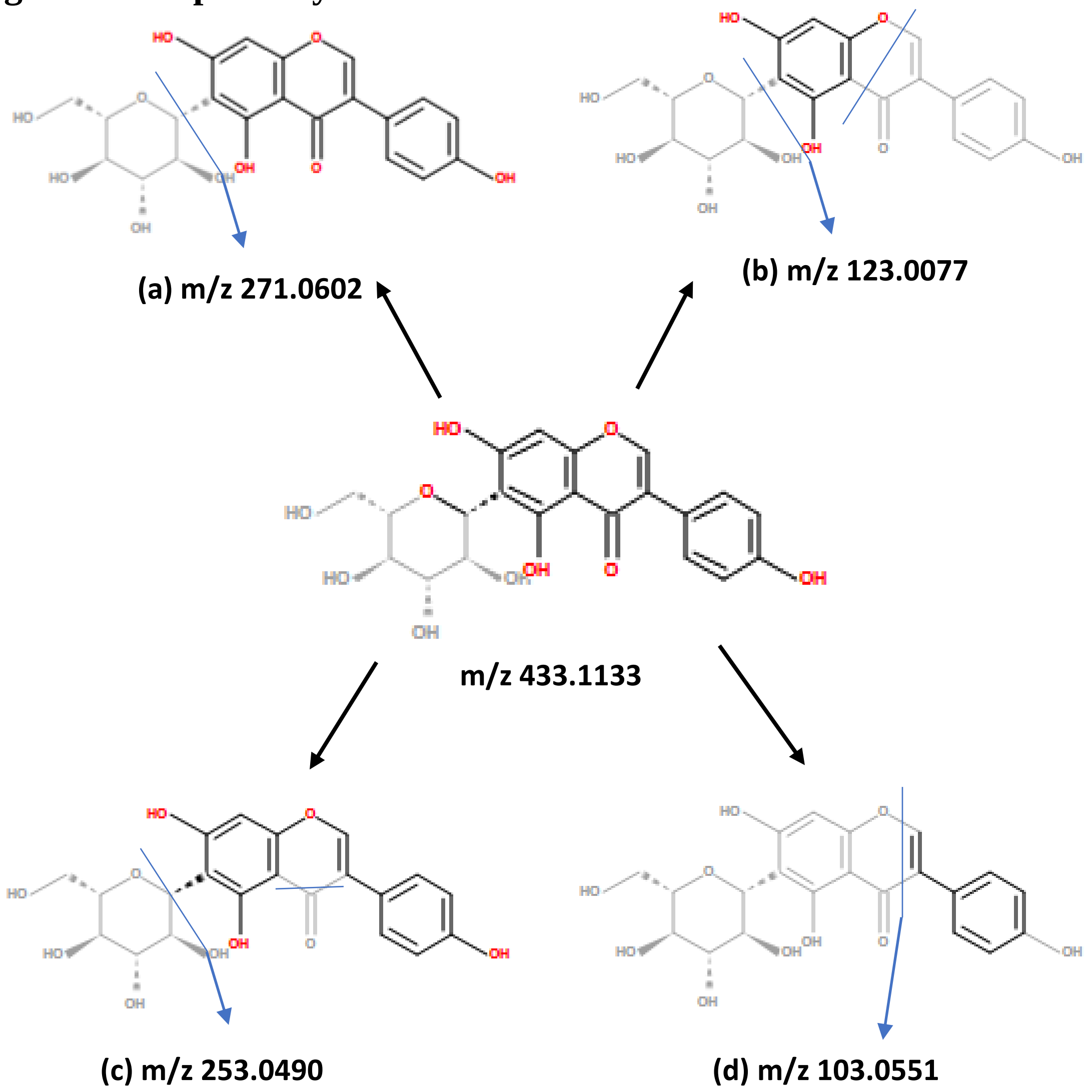

#30 Undulatone

1) MS/MS spectrum

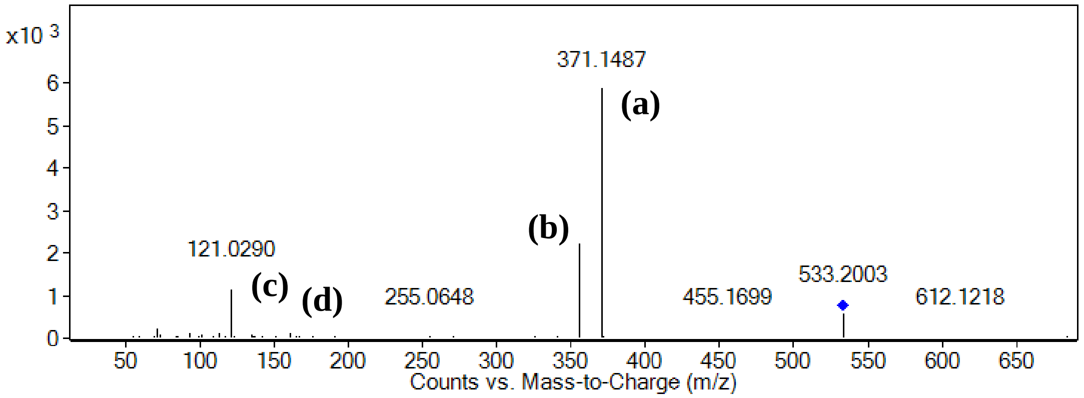

2) MS/MS fragment table

	Mass	Intensity	Weight(%)	No. of candid.	Best score
1	371.1488	9595.82	68.2	5	78.5
2	356.1253	4429.32	29.0	3	87.6
3	121.0291	1061.00	0.8	3	80.7
4	161.0444	354.60	0.5	1	73.1
5	122.0357	232.01	0.2	6	77.1
6	101.0239	210.44	0.1	5	93.4
7	71.0137	204.12	0.1	14	94.2
8	83.0139	150.62	0.1	12	97.7
9	113.0236	116.27	0.1	5	87.2
10	73.0291	112.18	0.0	13	90.4

3) Fragmentation pathway

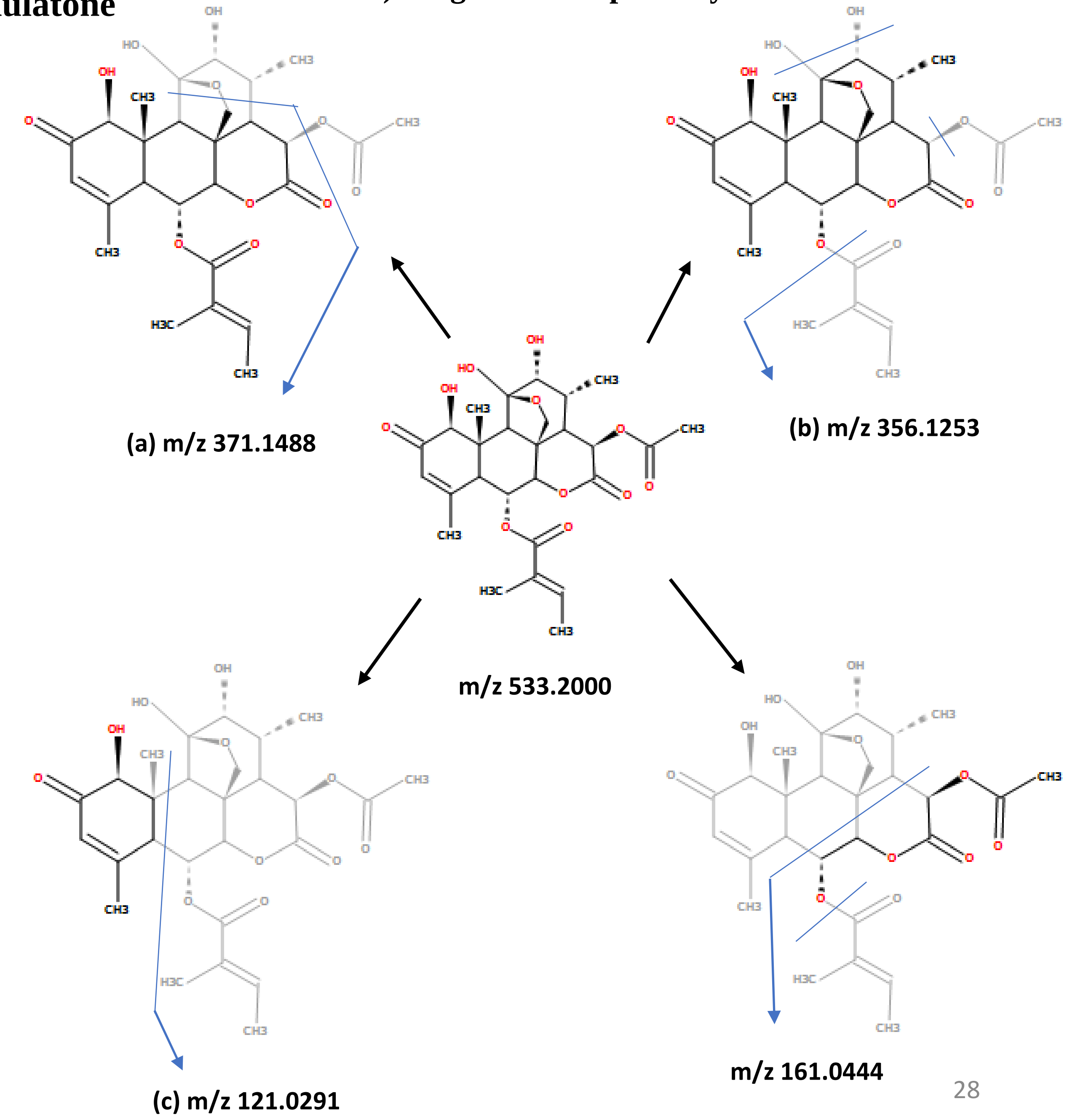

#31 Baicalin

1) MS/MS spectrum

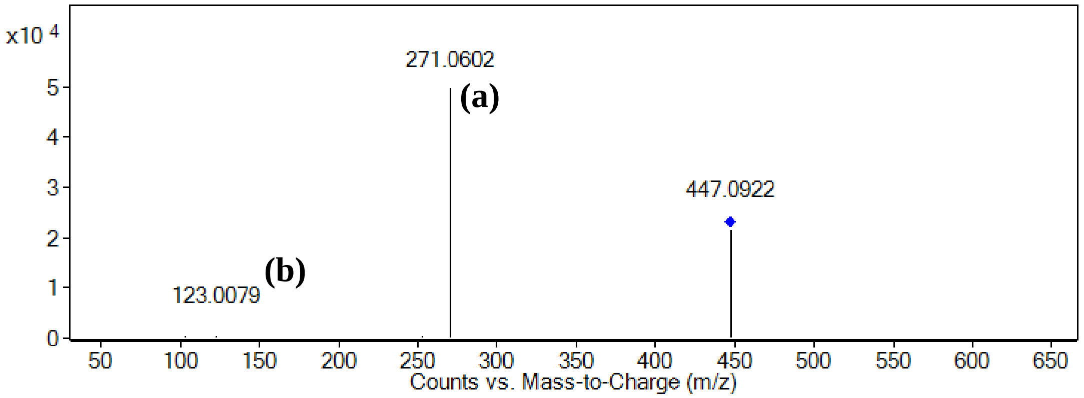

2) MS/MS fragment table

	Mass	Intensity	Weight(%)	No. of candid.	Best score
1	271.0606	4236.98	91.7	4	98.9
2	123.0080	205.76	0.9	2	87.1
3	169.0126	187.95	1.6	2	93.2
4	103.0541	113.22	0.4	1	94.2
5	77.0380	55.85	0.1	1	70.6
6	105.0332	54.33	0.2	1	80.9
7	95.0131	52.42	0.1	3	34.0
8	68.9968	45.32	0.1	4	78.5
9	71.0127	44.02	0.1	15	87.5
10	253.0474	43.07	0.8	4	80.4

3) Fragmentation pathway

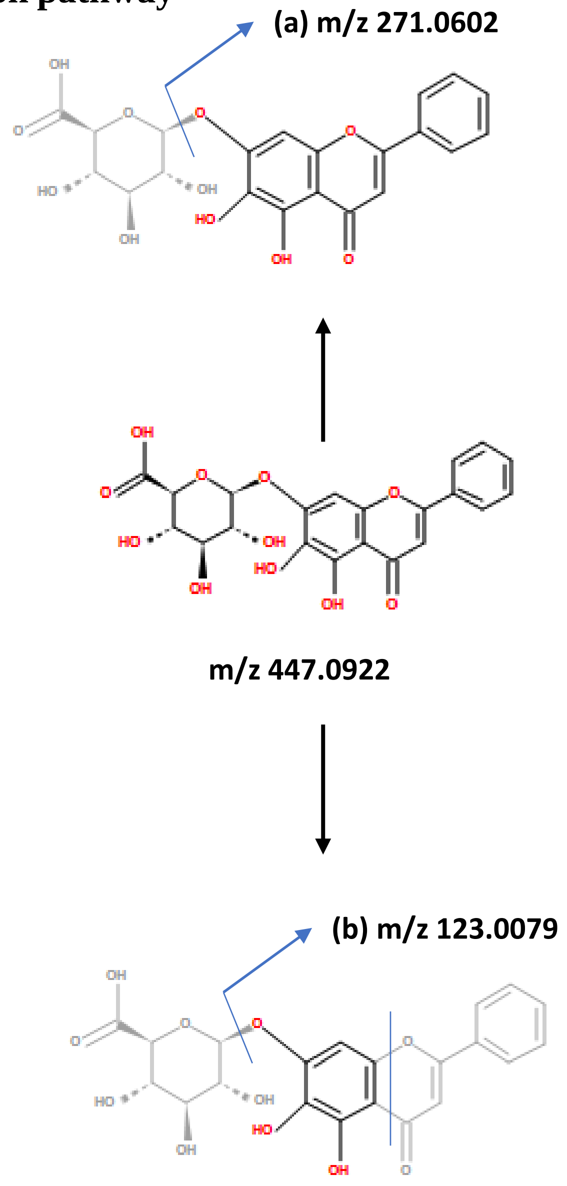

#33 Hispidulin 7-glucuronide

Hispidulin 7-glucuronide
$[M+H]^+$

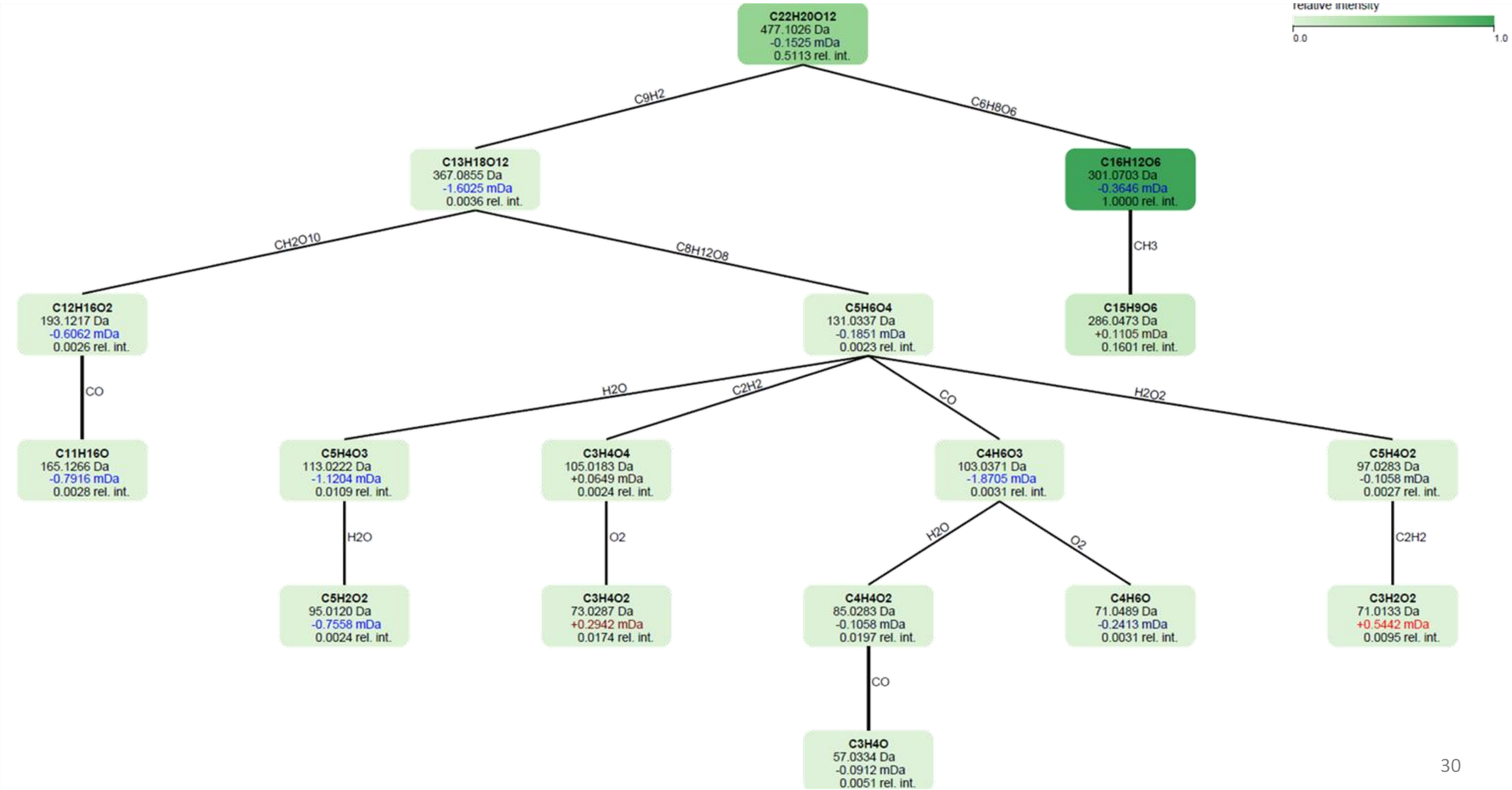

#34 Chrysin 7-glucuronide

1) MS/MS spectrum

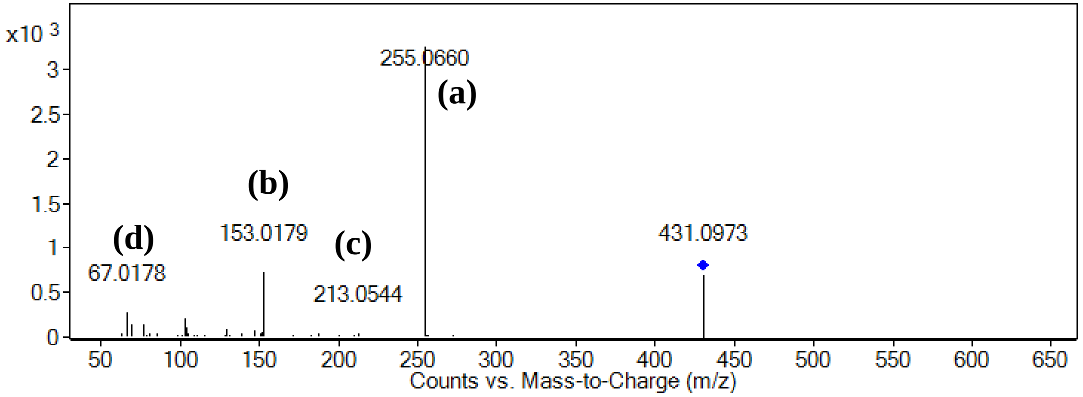

2) MS/MS fragment table

	Mass	Intensity	Weight(%)	No. of candid.	Best score
1	255.0647	20261.33	97.3	4	98.8
2	153.0169	283.68	0.5	2	88.6
3	255.1344	68.08	0.3	0	0.0
4	213.0542	63.63	0.2	4	76.1
5	68.9971	53.93	0.0	4	78.8
6	67.0181	50.37	0.0	5	40.9
7	103.0546	44.60	0.0	1	93.7
8	255.3010	42.42	0.2	0	0.0
9	255.0382	41.91	0.2	0	0.0
10	77.0386	41.03	0.0	2	71.5

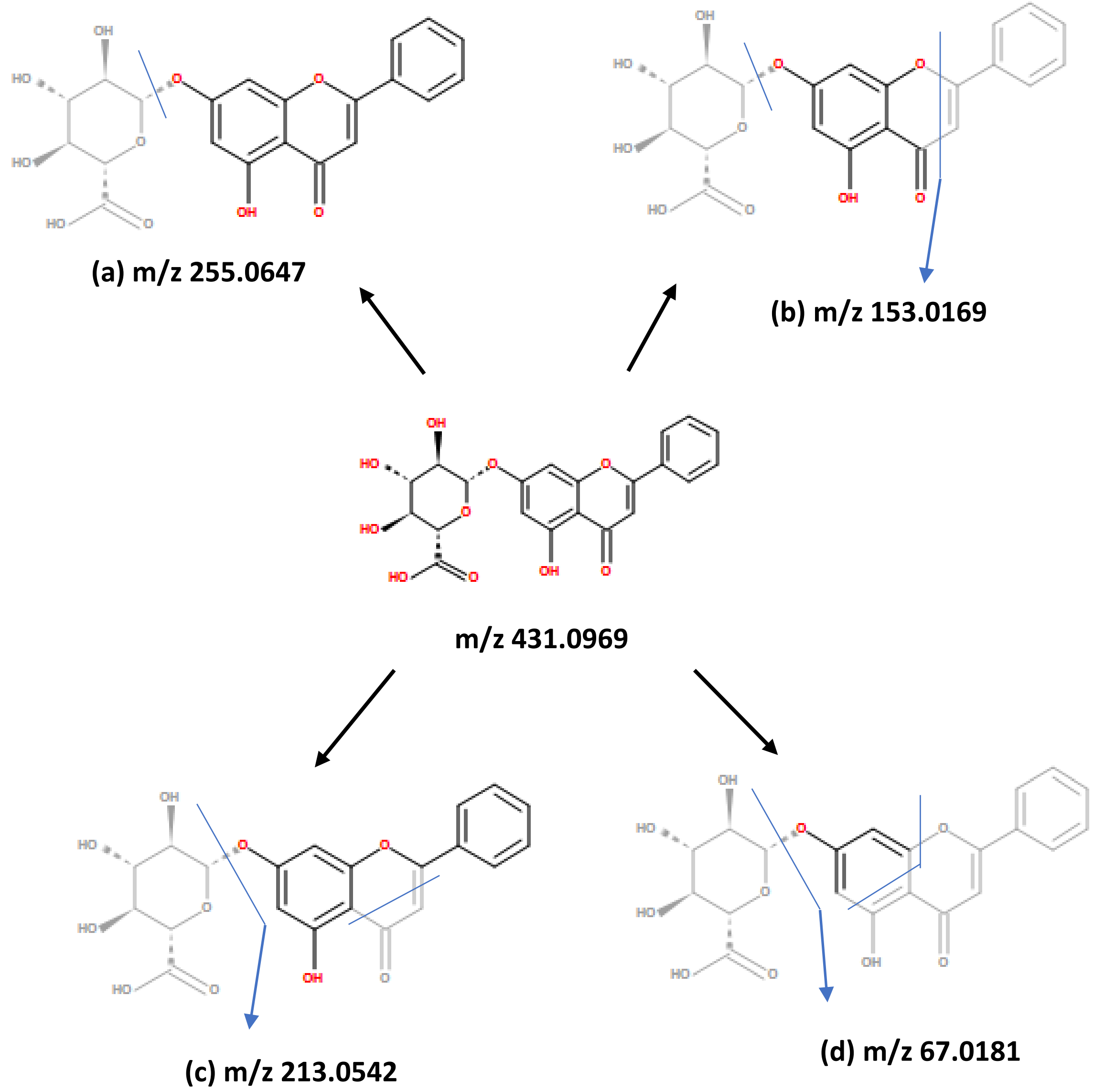

#35 Wogonin 7-glucuronide

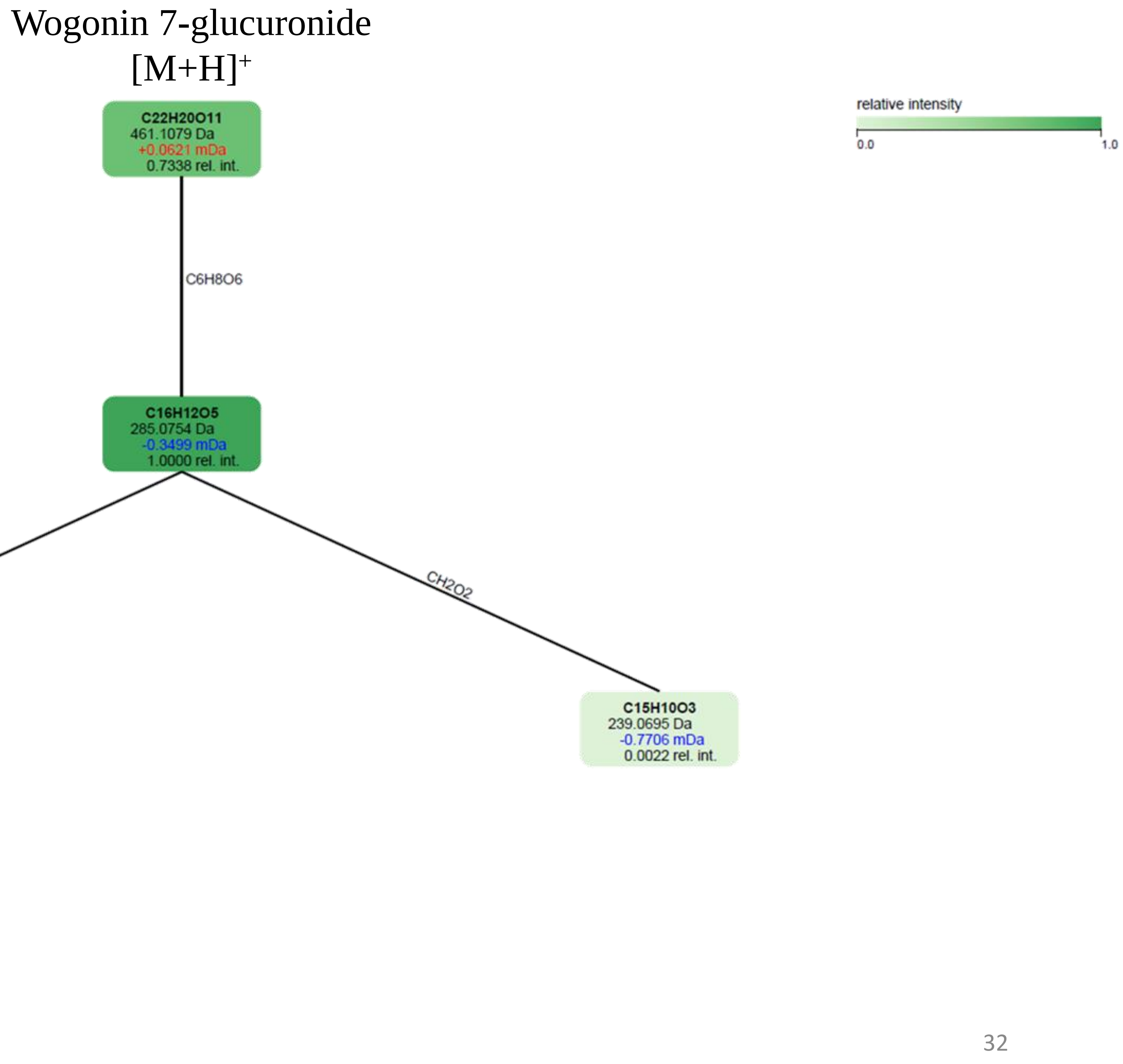

#36 Apigenin 7-glucuronide

1) MS/MS spectrum

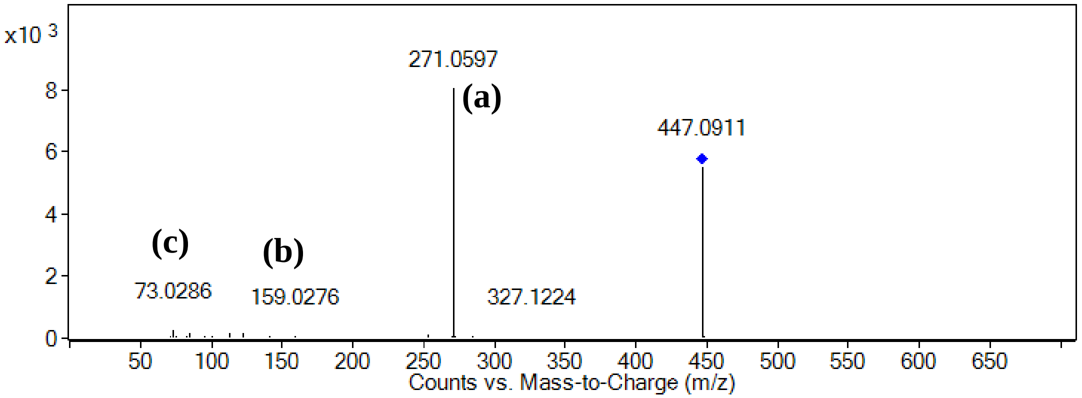

2) MS/MS fragment table

	Mass	Intensity	Weight(%)	No. of candid.	Best score
1	271.0598	8531.35	93.0	4	99.3
2	269.0436	28.95	0.3	3	96.6
3	73.0288	129.28	0.1	14	93.6
4	159.0293	77.19	0.3	10	93.4
5	285.0768	75.60	0.9	5	91.6
6	253.0497	50.92	0.5	4	91.1
7	103.0376	26.38	0.0	20	87.7
8	71.0128	72.03	0.1	15	87.5
9	85.0285	160.45	0.2	9	87.4
10	141.0178	72.56	0.2	4	87.0

3) Fragmentation pathway

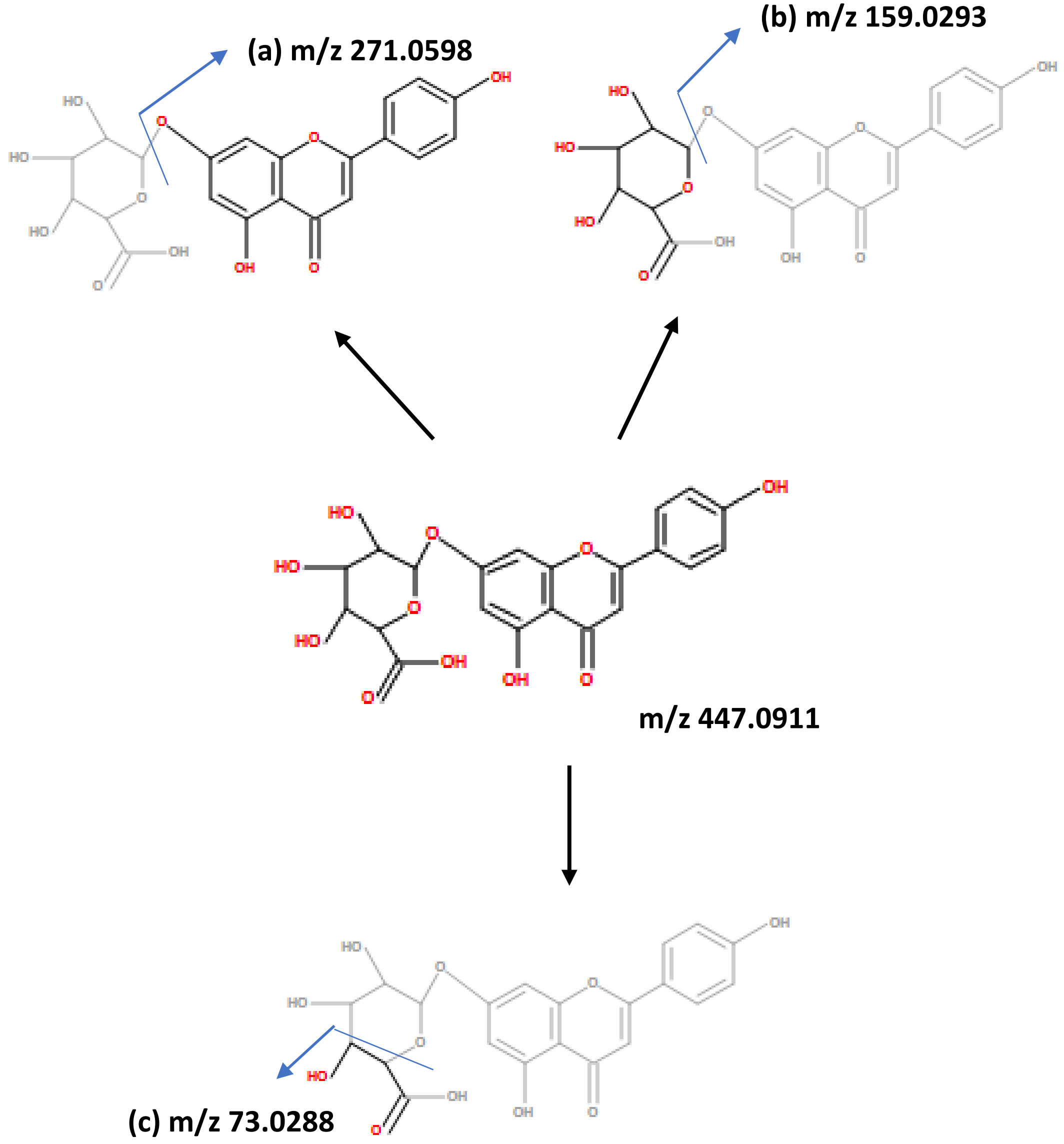

#37 Kaempferide

1) MS/MS spectrum

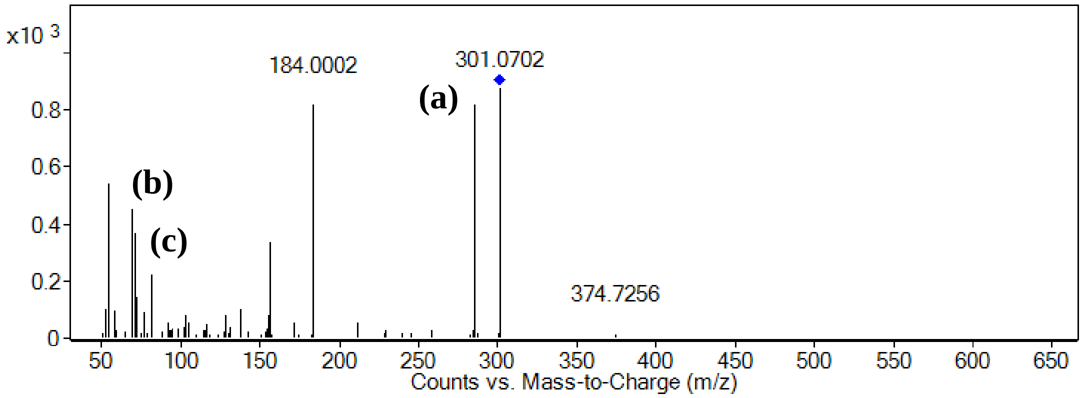

2) MS/MS fragment table

	Mass	Intensity	Weight(%)	No. of candid.	Best score
1	286.0452	873.46	54.7	1	89.9
2	183.9999	698.26	18.1	0	0.0
3	54.0101	540.25	1.2	4	55.9
4	71.0126	341.91	1.3	4	61.1
5	68.9968	286.09	1.0	4	50.6
6	77.0382	273.55	1.2	2	63.4
7	156.0047	269.50	5.0	0	0.0
8	82.0046	213.45	1.1	4	38.5
9	72.0205	198.14	0.8	4	61.1
10	58.0053	177.92	0.5	2	80.6

3) Fragmentation pathway

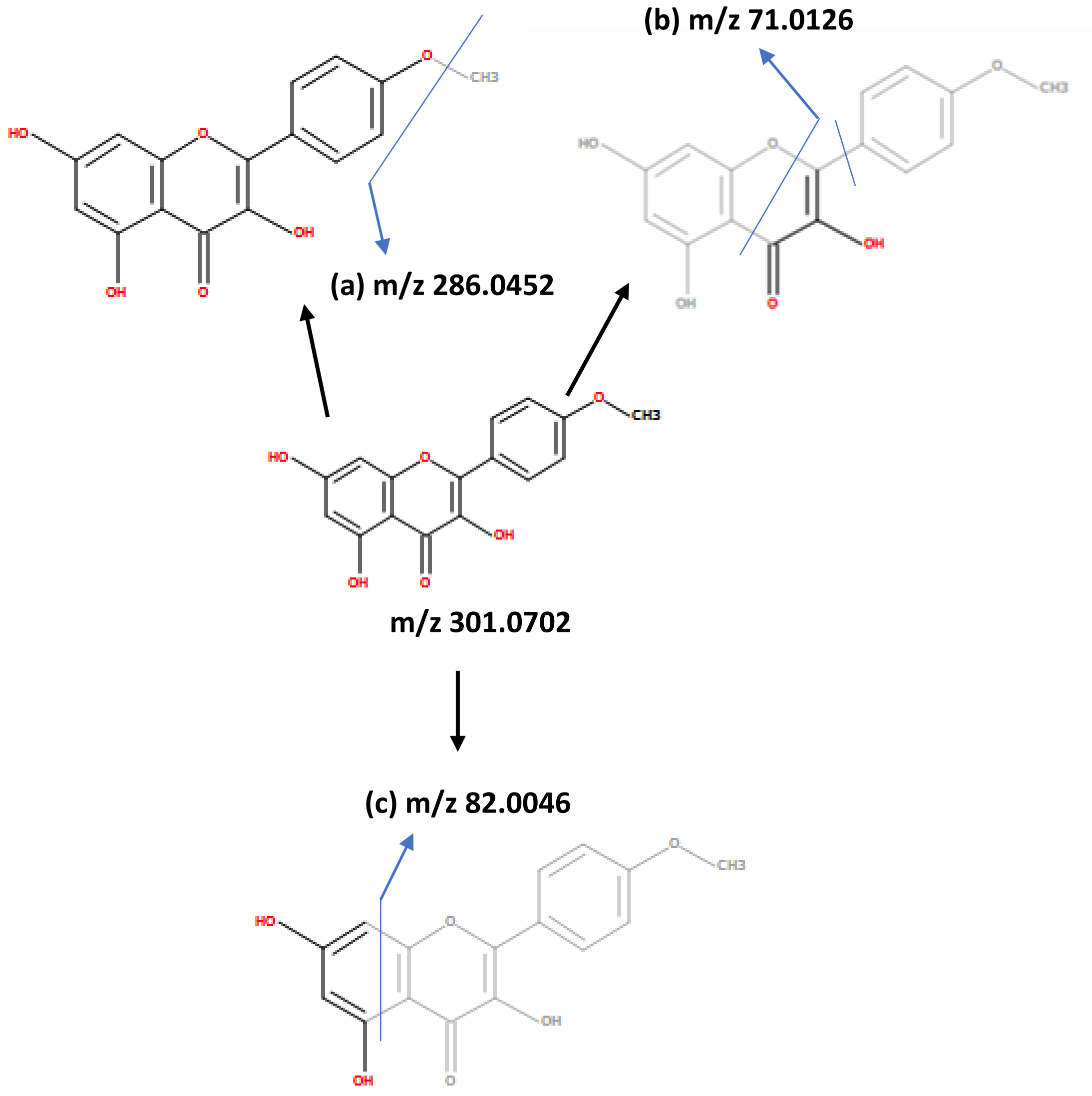

#38 Baicalein

1) MS/MS spectrum

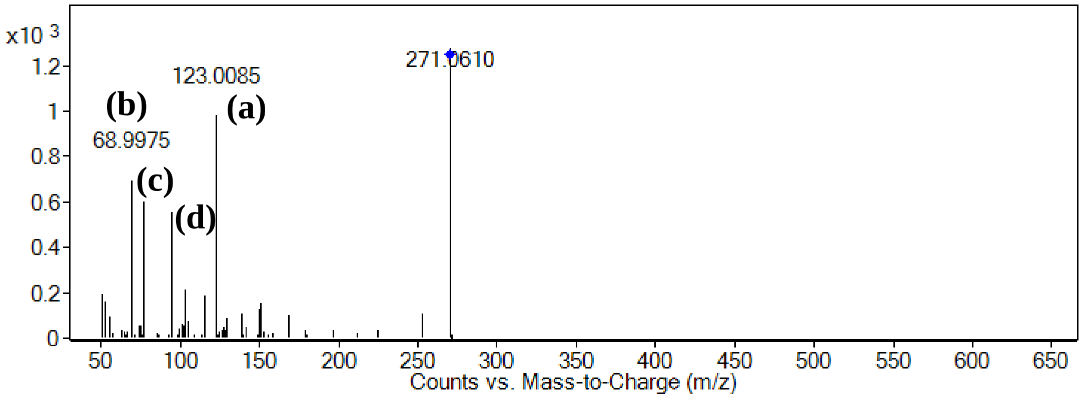

2) MS/MS fragment table

	Mass	Intensity	Weight(%)	No. of candid.	Best score
1	123.0086	977.69	24.9	2	85.0
2	68.9976	668.37	5.4	4	50.3
3	77.0390	583.11	5.8	1	71.1
4	95.0132	540.34	8.2	4	34.0
5	51.0231	191.62	0.8	4	43.3
6	103.0548	172.26	3.1	1	92.9
7	53.0024	154.79	0.7	4	50.7
8	151.0533	150.45	5.8	0	0.0
9	115.0547	148.44	3.3	1	82.7
10	150.0463	125.75	4.8	0	0.0

3) Fragmentation pathway

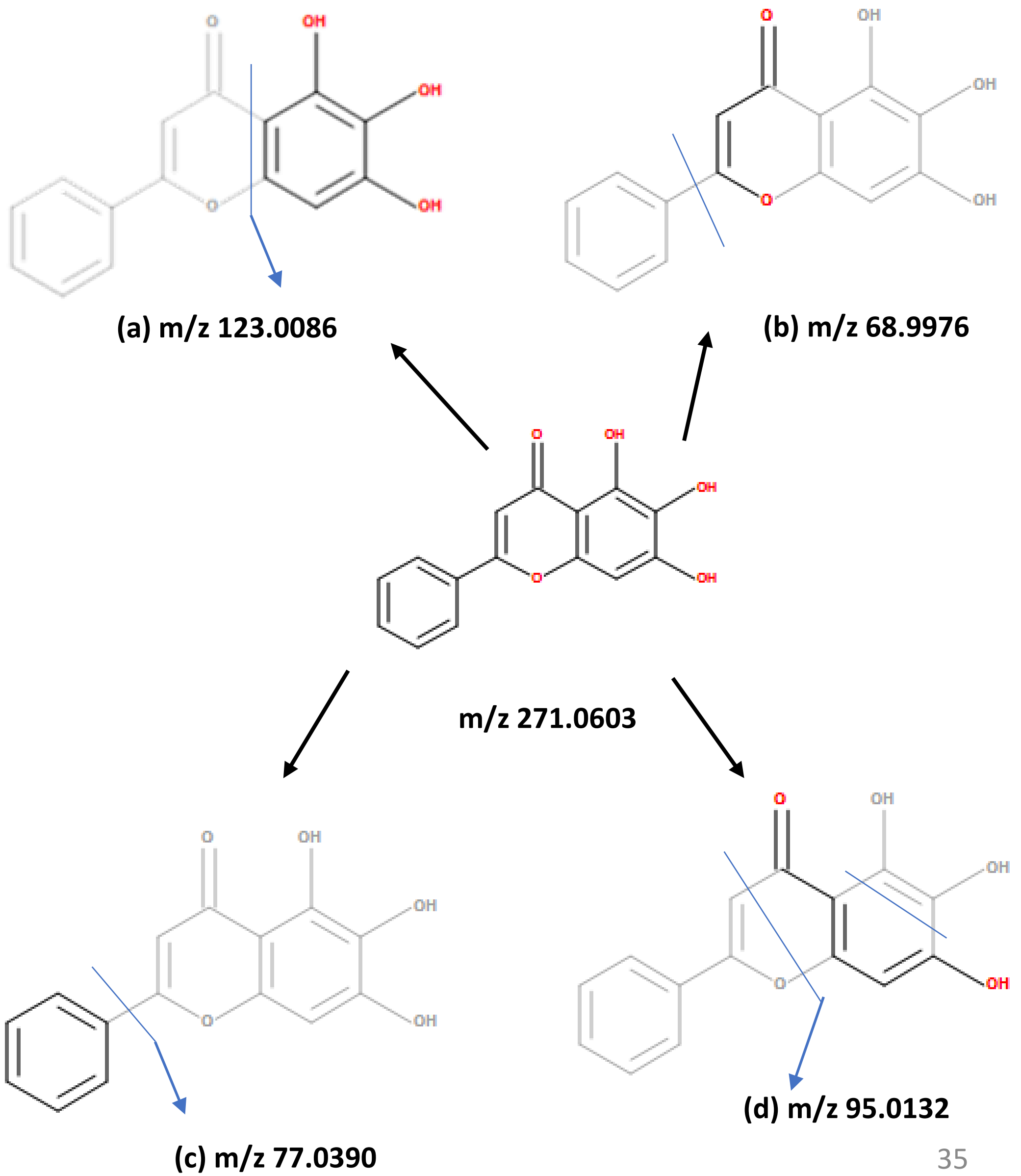

#39 Kadsurin A

Kadsurin A

$[M+NH_4]^+$

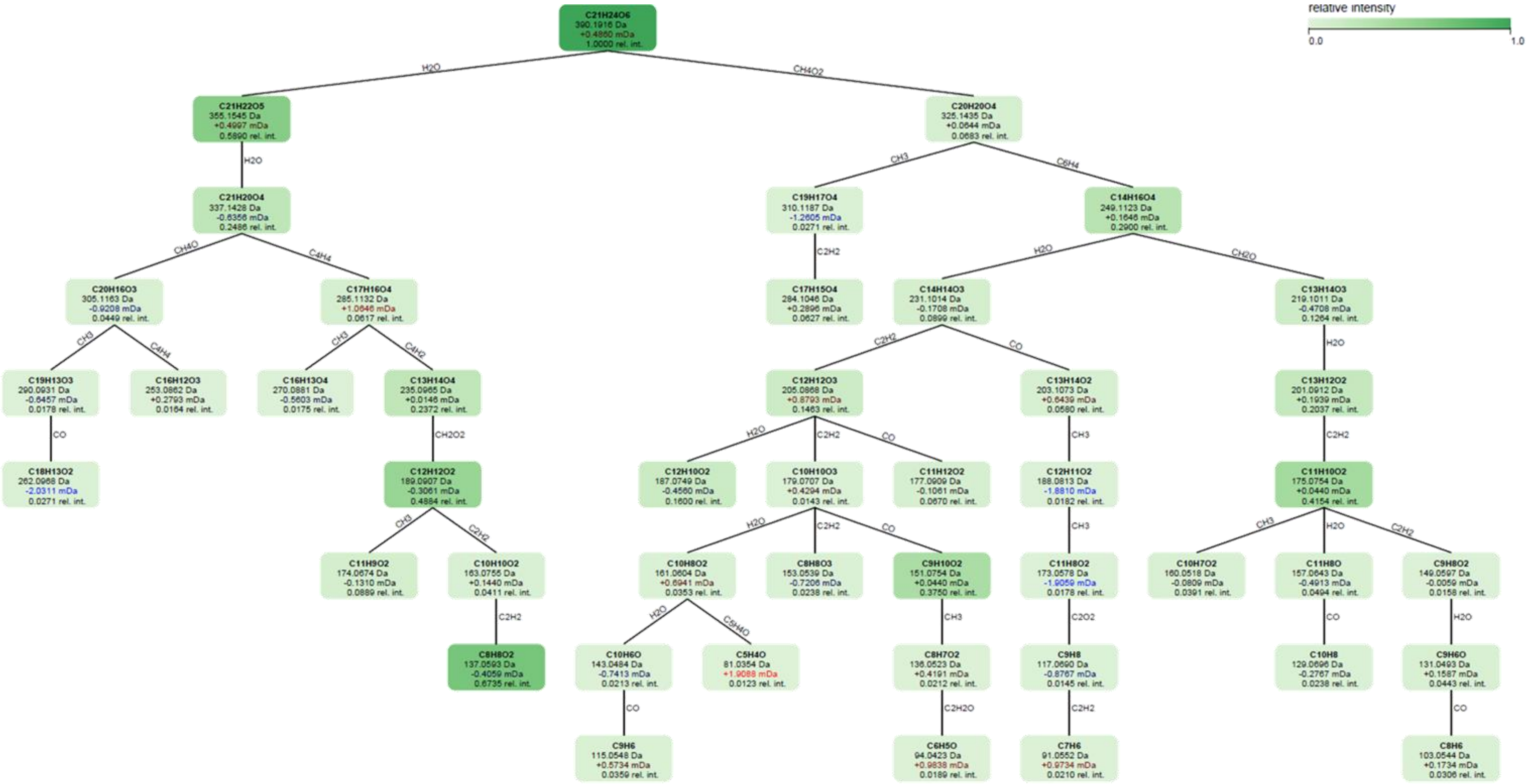

#40 Wogonin

1) MS/MS spectrum

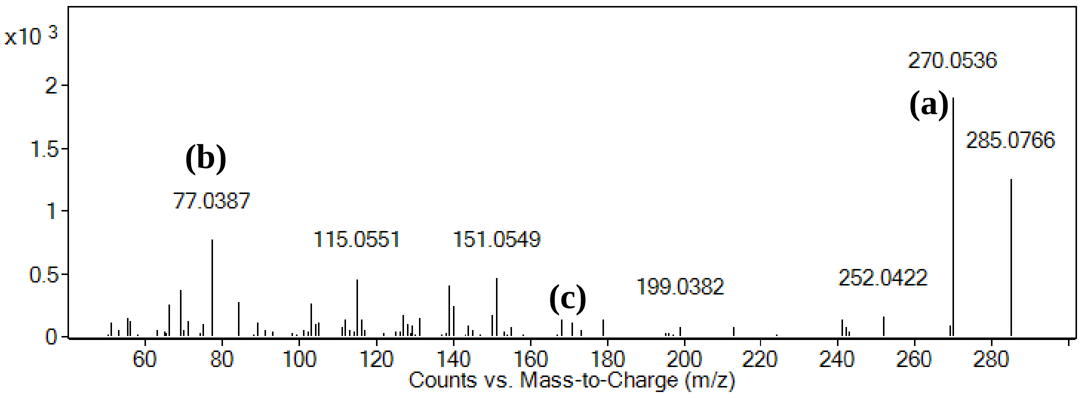

2) MS/MS fragment table

	Mass	Intensity	Weight(%)	No. of candid.	Best score
▶ 1	270.0530	5574.21	78.1	2	98.3
2	104.0625	43.15	0.1	1	96.0
3	269.0433	105.90	1.5	2	95.0
4	131.0495	105.76	0.3	2	93.8
5	103.0548	109.66	0.2	1	93.1
6	168.0050	290.64	1.6	1	90.8
7	116.0621	97.32	0.3	1	87.5
8	242.0581	193.47	2.2	4	86.1
9	140.0090	181.70	0.7	1	85.0
10	139.0037	54.65	0.2	1	83.7

3) Fragmentation pathway

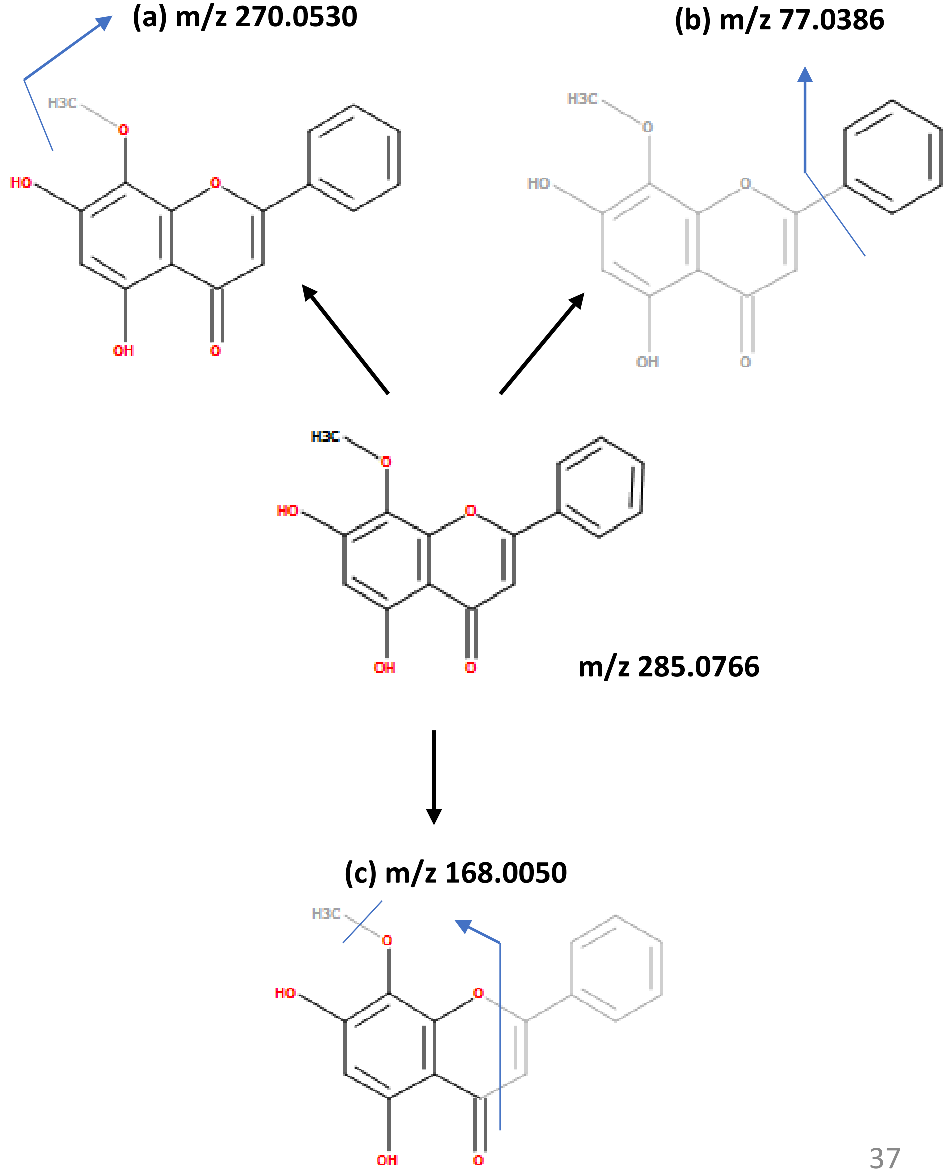

#41 5,3'-Dihydroxy-7,4'-dimethoxy-4-phenylcoumarin

1) MS/MS spectrum

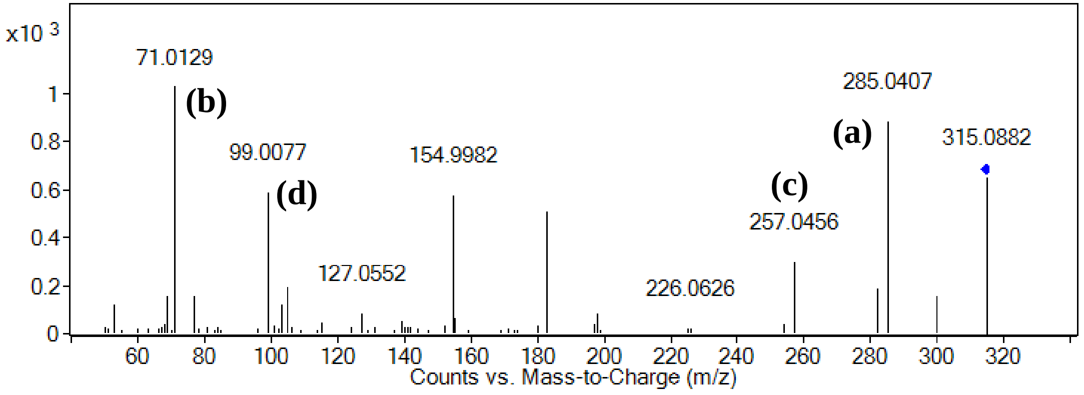

2) MS/MS fragment table

	Mass	Intensity	Weight(%)	No. of candid.	Best score
1	285.0397	866.82	43.5	2	98.2
2	154.9971	742.30	11.0	0	0.0
3	71.0126	723.20	2.3	4	61.1
4	182.9917	512.22	10.6	0	0.0
5	99.0075	512.05	3.1	2	38.6
6	53.0030	183.28	0.3	4	49.5
7	282.0520	162.93	8.0	3	87.3
8	257.0438	135.69	5.5	6	86.4
9	77.0383	126.87	0.5	2	63.6
10	103.0547	96.65	0.6	1	58.1

3) Fragmentation pathway

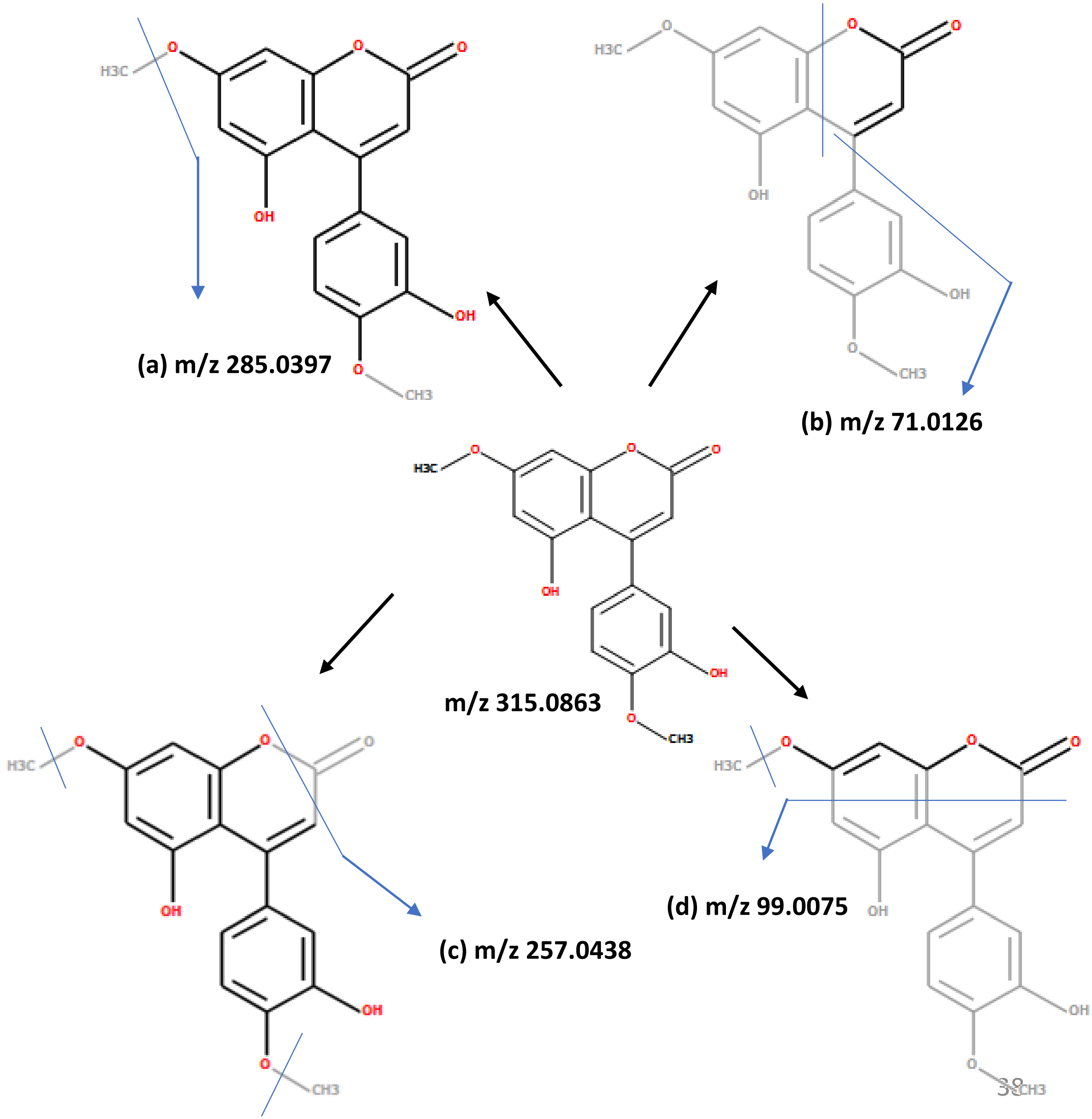

#42 Skullcapflavone II

1) MS/MS spectrum

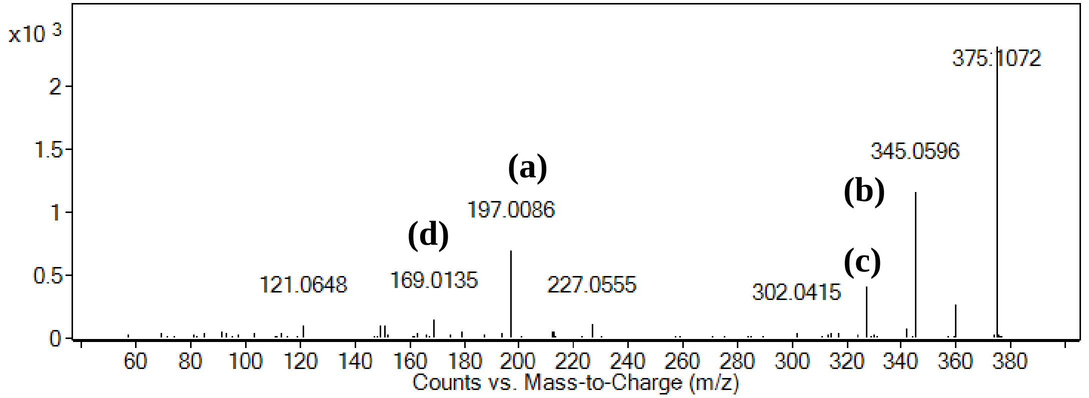

2) MS/MS fragment table

	Mass	Intensity	Weight(%)	No. of candid.	Best score
1	197.0080	900.56	13.4	1	87.4
2	345.0605	791.04	36.2	2	98.5
3	169.0127	657.42	7.2	1	86.8
4	85.0283	562.31	1.6	4	43.3
5	327.0500	389.06	16.0	3	83.4
6	55.0174	258.34	0.3	4	60.6
7	113.0236	203.22	1.0	4	38.5
8	121.0651	170.83	1.0	4	76.2
9	68.9969	159.91	0.3	4	50.7
10	151.0386	148.84	1.3	4	93.8

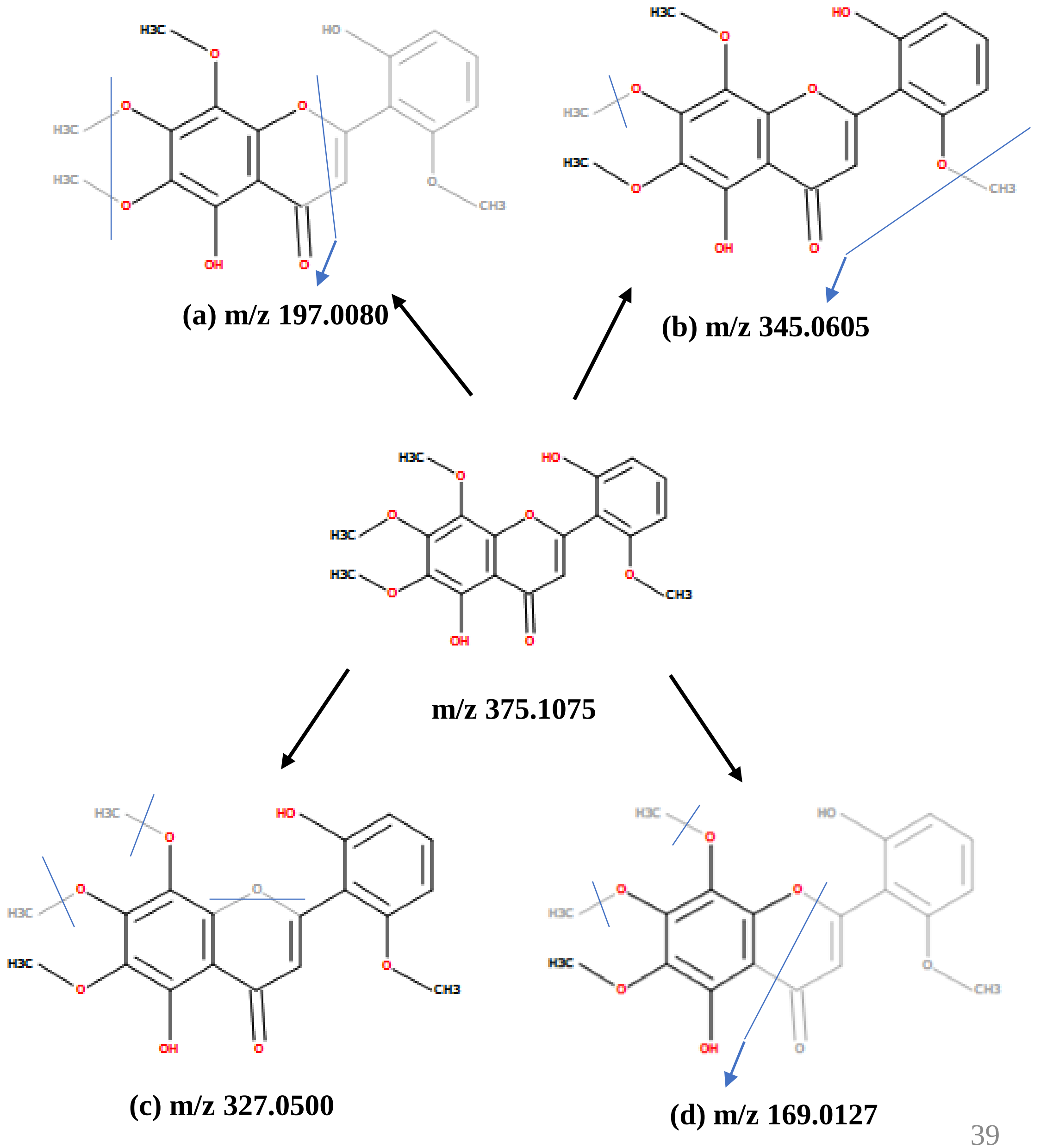

#44 PE (19:0/0:0)

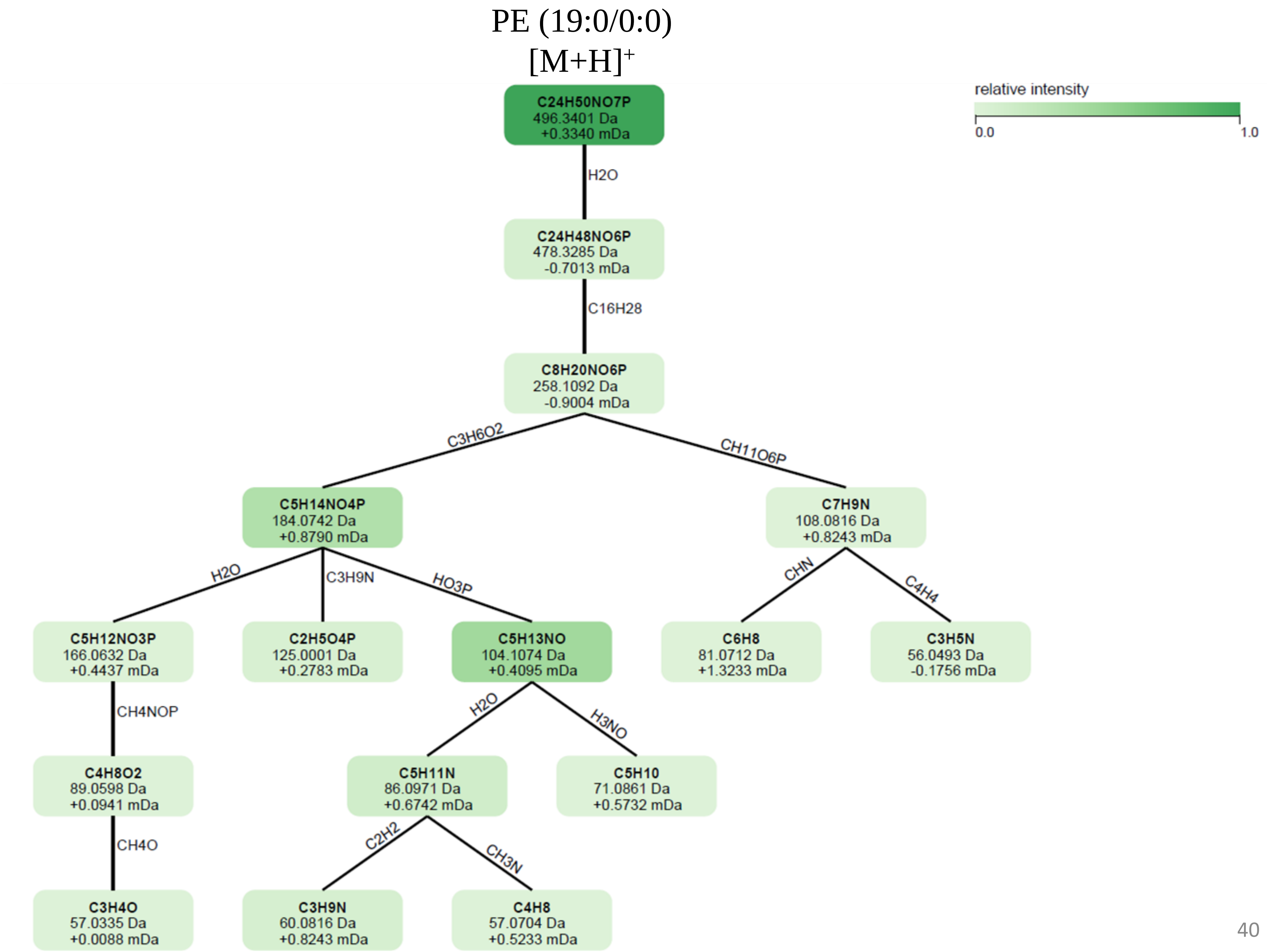

PE (19:0/0:0)
[M+H]+
relative intensity
0.0
1.0
C24H50NO7P
496.3401 Da
+0.3340 mDa
H2O
C24H48NO6P
478.3285 Da
-0.7013 mDa
C16H28
C8H20NO6P
258.1092 Da
-0.9004 mDa
C3H6O2
CH11O6P
C5H14NO4P
184.0742 Da
+0.8790 mDa
C7H9N
108.0816 Da
+0.8243 mDa
H2O
C3H9N
HO3P
CHN
C4H4
C5H12NO3P
166.0632 Da
+0.4437 mDa
C2H5O4P
125.0001 Da
+0.2783 mDa
C5H13NO
104.1074 Da
+0.4095 mDa
C6H8
81.0712 Da
+1.3233 mDa
C3H5N
56.0493 Da
-0.1756 mDa
CH4NOP
H2O
H3NO
C4H8O2
89.0598 Da
+0.0941 mDa
C5H11N
86.0971 Da
+0.6742 mDa
C5H10
71.0861 Da
+0.5732 mDa
CH4O
C2H2
CH3N
C3H4O
57.0335 Da
+0.0088 mDa
C3H9N
60.0816 Da
+0.8243 mDa
C4H8
57.0704 Da
+0.5233 mDa

#47 1,2 di-(9Z, 12, 15Z-octadecatrienoyl)-3-O-beta-D-galactosyl-sn-glycerol

1,2 di-(9Z, 12, 15Z-octadecatrienoyl)-3-O-Beta-D-galactosyl-sn-glycerol $[M+NH_4]^+$

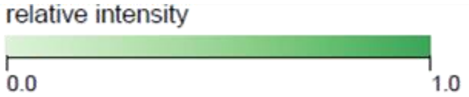

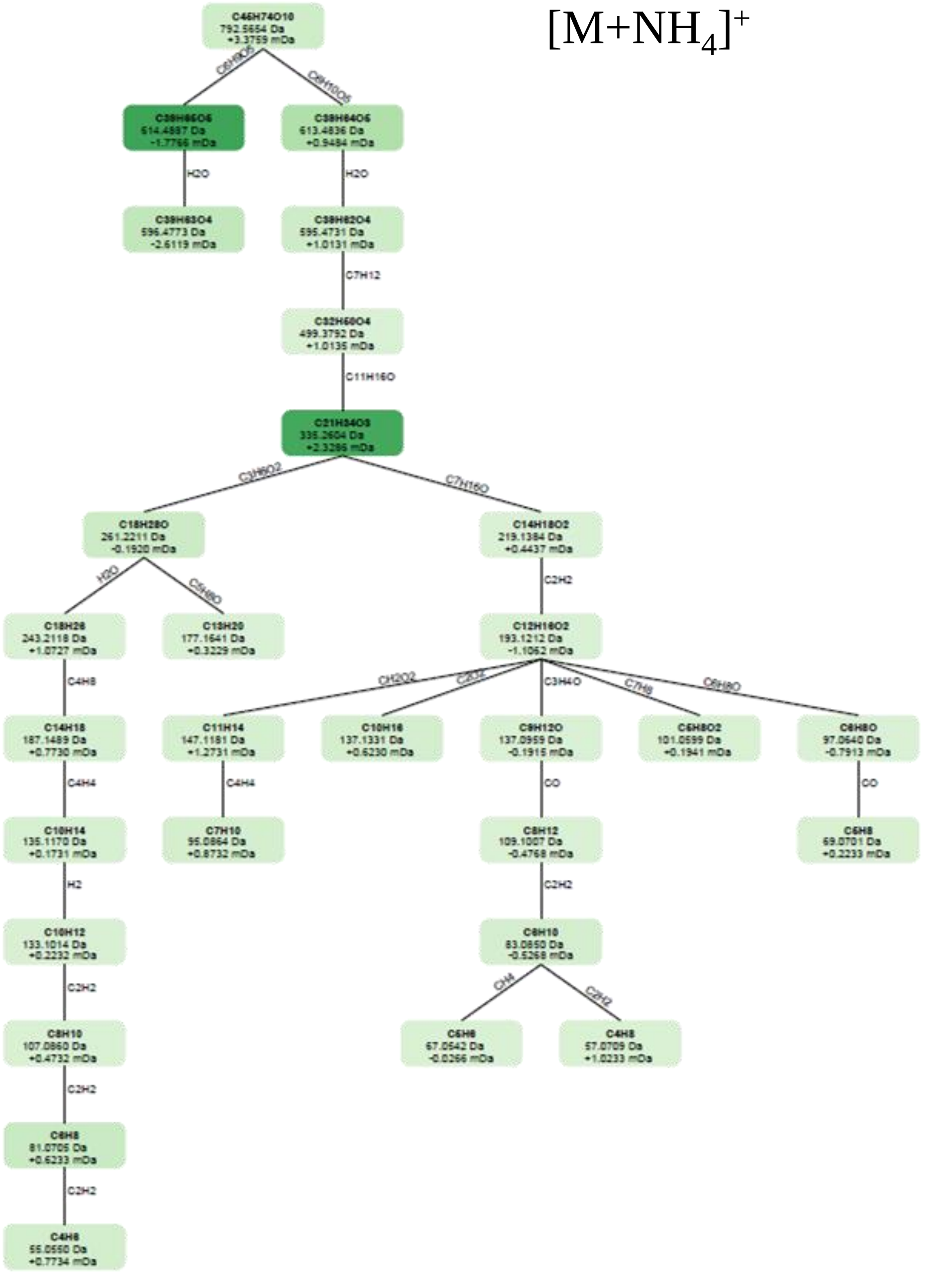

Supplement: Supplementary file 3 — Additional file 3: Figure S2. The proposed fragmentation pathways of the commonly identified compounds. [file 13020_2022_610_MOESM3_ESM.pdf]
