# Supplementary material for: Metabolomics analyses of traditional Chinese medicine formula Shuang Huang Lian by UHPLC-QTOF-MS/MS
Source: Chin Med. 2022 May 30;17:62. doi: 10.1186/s13020-022-00610-x (PMC9150355; doi:10.1186/s13020-022-00610-x)
Supplement: Supplementary file 4 — Additional file 4: Table S1. The chemical components identified with both names and formulas in SHL granule preparation form. [file 13020_2022_610_MOESM4_ESM.docx]

**Table S1. The chemical components identified with both names and formulas in SHL granule preparation form (n = 3)**

| **No.** | **Formula** | **Name** | **t_R_ (min)**  **(Mean ± SD)** | **Observed Mass (Mean ± SD)** | **Database Mass** | **Precursor ion, m/z** |
| --- | --- | --- | --- | --- | --- | --- |
| 1 | C_30_H_26_O_10_ | Guibourtinidol-(4alpha-6)-catechin | 0.93 ± 0.01 | 546.1525 ± 0.0006 | 546.1524 | 545.1465, [M-H]¯ |
| 2 | C_20_H_24_O_5_S | Ethynylestradiol 3-sulfate | 1.07 ± 0.00 | 376.1333 ± 0.0004 | 376.1314 | 375.1259, [M-H]¯ |
| 3 | C_15_H_18_O_10_ | Veratric acid glucuronide | 1.38 ± 0.02 | 358.0909 ± 0.0001 | 358.0900 | 381.0807, [M+Na]⁺ |
| 4 | C_12_H_22_O_11_ | Gentiobiose | 1.42 ± 0.02 | 342.1174 ± 0.0003 | 342.1162 | 365.1069, [M+Na]⁺ |
| 5 | C_20_H_21_ClN_2_O_4_ | Fipexide | 1.46 ± 0.01 | 388.1192 ± 0.0012 | 388.1195 | 387.1107, [M-H]¯ |
| 6 | C_21_H_20_O_13_ | Myricetin 3'-glucoside | 1.71 ± 0.00 | 480.0895 ± 0.0009 | 480.0916 | 479.0815, [M-H]¯ |
| 7 | C_9_H_17_NO_8_ | Neuraminic acid | 1.83 ± 0.03 | 267.0964 ± 0.0006 | 267.0971 | 268.1043, [M+H]⁺ |
| 8 | C_23_H_40_N_2_O_18_ | 3'-Sialyllactosamine | 1.89 ± 0.01 | 632.2274 ± 0.0021 | 632.2276 | 631.2184, [M-H]¯ |
| 9 | C_33_H_40_O_22_ | Quercetin 3,7,4'-triglucoside | 1.92 ± 0.04 | 788.1966 ± 0.0016 | 788.1956 | 787.1907, [M-H]¯ |
| 10 | C_8_H_10_O_3_ | 3,4-Dihydroxyphenyl ethanol | 2.89 ± 0.00 | 154.0628 ± 0.0004 | 154.0617 | 153.0552, [M-H]¯ |
| 11 | C_28_H_16_O_5_ | Naphthofluorescein | 4.61 ± 0.02 | 432.1022 ± 0.0012 | 432.1031 | 431.0940, [M-H]¯ |
| 12 | C_13_H_22_N_4_O_7_S | Met-Asp-Asn | 7.09 ± 0.06 | 378.1215 ± 0.0003 | 378.1197 | 377.1145, [M-H]¯ |
| 13 | C_21_H_26_N_4_O_8_ | Trp-Glu-Glu | 7.88 ± 0.01 | 462.1731 ± 0.0009 | 462.1751 | 485.1633, [M+Na]⁺ |
| 14 | C_10_H_16_N_4_O_7_ | Asn-Asp-Gly | 7.98 ± 0.03 | 304.1031 ± 0.0003 | 304.1029 | 303.0962, [M-H]¯ |
| 15 | C_16_H_18_O_9_ | Chlorogenic Acid* | 8.15 ± 0.04 | 354.0950 ± 0.0000 | 354.0951 | 355.1023, [M+H]⁺ |
| 16 | C_9_H_6_O_3_ | Umbelliferone | 8.15 ± 0.04 | 162.0316 ± 0.0000 | 162.0316 | 163.0388, [M+H]⁺ |
| 17 | C_32_H_30_O_7_ | Kurzichalcolactone | 9.22 ± 0.02 | 526.2001 ± 0.0017 | 526.2005 | 525.1948, [M-H]¯ |
| 18 | C_10_H_10_O_4_ | Methyl caffeate | 9.75 ± 0.01 | 194.0579 ± 0.0001 | 194.0582 | 195.0652, [M+H]⁺ |
| 19 | C_10_H_12_O_5_ | Danielone | 9.75 ± 0.02 | 212.0683 ± 0.0001 | 212.0682 | 213.0755, [M+H]⁺ |
| 20 | C_16_H_22_O_10_ | Geniposidic acid | 9.76 ± 0.01 | 374.1211 ± 0.0001 | 374.1213 | 397.1106, [M+Na]⁺ |
| 21 | C_8_H_6_O_3_ | Piperonal | 9.76 ± 0.01 | 150.0318 ± 0.0000 | 150.0319 | 151.0391, [M+H]⁺ |
| 22 | C_19_H_34_N_2_O_2_S_4_ | AN-7 | 10.24 ± 0.05 | 450.1494 ± 0.0006 | 450.1490 | 449.1420, [M-H]¯ |
| 23 | C_16_H_18_O_8_ | p-Coumaroyl quinic acid | 10.48 ± 0.02 | 338.1003 ± 0.0002 | 338.1002 | 339.1078, [M+H]⁺ |
| 24 | C_22_H_27_F_3_O_4_S | Fluticasone | 10.63 ± 0.03 | 444.1596 ± 0.0002 | 444.1595 | 443.1522, [M-H]¯ |
| 25 | C_19_H_25_N_5_O_6_ | Asn-Thr-Trp | 11.10 ± 0.00 | 419.1793 ± 0.0001 | 419.1791 | 420.1866, [M+H]⁺ |
| 26 | C_16_H_22_O_9_ | Tarennoside | 11.72 ± 0.04 | 358.1264 ± 0.0000 | 358.1264 | 359.1337, [M+H]⁺ |
| 27 | C_16_H_28_N_6_O_8_ | Arg-Glu-Glu | 11.72 ± 0.07 | 432.1969 ± 0.0001 | 432.1960 | 431.1894, [M-H]¯ |
| 28 | C_15_H_26_N_6_O_6_ | Asp-Arg-Pro | 11.72 ± 0.07 | 386.1912 ± 0.0011 | 386.1916 | 385.1831, [M-H]¯ |
| 29 | C_10_H_12_O_4_ | Paeonilactone B | 11.78 ± 0.05 | 196.0736 ± 0.0001 | 196.0732 | 197.0808, [M+H]⁺ |
| 30 | C_21_H_36_N_2_O_14_ | Galβ1-3GalNAcα-Thr | 11.91 ± 0.02 | 540.2159 ± 0.0007 | 540.2162 | 539.2091, [M-H]¯ |
| 31 | C_11_H_16_O_5_ | Depdecin | 11.98 ± 0.07 | 228.0986 ± 0.0003 | 228.0994 | 227.0910, [M-H]¯ |
| 32 | C_20_H_27_N_5_O_6_ | Thr-Gln-Trp | 15.15 ± 0.00 | 433.1945 ± 0.0001 | 433.1943 | 434.2017, [M+H]⁺ |
| 33 | C_20_H_24_N_4_O_6_ | Pro-Trp-Asp | 15.15 ± 0.01 | 416.1680 ± 0.0000 | 416.1696 | 434.2017, [M+NH_4_]⁺ |
| 34 | C_14_H_18_N_2_O_5_ | Glutamylphenylalanine | 15.65 ± 0.07 | 294.1218 ± 0.0001 | 294.1216 | 295.1290, [M+H]⁺ |
| 35 | C_18_H_26_N_4_O_6_ | Tyr-Val-Asn | 16.34 ± 0.03 | 394.1842 ± 0.0001 | 394.1852 | 412.2178, [M+NH_4_]⁺ |
| 36 | C_15_H_21_N_5_O_8_ | Asp-Glu-His | 16.34 ± 0.03 | 399.1396 ± 0.0000 | 399.1390 | 417.1734, [M+NH_4_]⁺ |
| 37 | C_21_H_27_FO_6_ | Triamcinolone | 16.34 ± 0.04 | 394.1807 ± 0.0003 | 394.1794 | 393.1736, [M-H]¯ |
| 38 | C_16_H_18_N_6_O_4_ | 2-Phenylaminoadenosine | 16.5 ± 0.1 | 358.1394 ± 0.0003 | 358.1387 | 357.1315, [M-H]¯ |
| 39 | C_27_H_30_O_16_ | Luteolin 7-galactoside-4'-glucoside | 17.73 ± 0.00 | 610.1536 ± 0.0002 | 610.1537 | 611.1611, [M+H]⁺ |
| 40 | C_15_H_10_O_7_ | Hypolaetin | 17.73 ± 0.00 | 302.0427 ± 0.0004 | 302.0426 | 303.0495, [M+H]⁺ |
| 41 | C_21_H_18_O_12_ | Luteolin 3'-glucuronide | 18.13 ± 0.02 | 462.0814 ± 0.0000 | 462.0797 | 463.0872, [M+H]⁺ |
| 42 | C_20_H_22_O_6_ | Pedicellin | 18.93 ± 0.01 | 358.1397 ± 0.0012 | 358.1374 | 357.1314, [M-H]¯ |
| 43 | C_21_H_20_O_11_ | Luteolin-7-O-glucoside* | 19.30 ± 0.02 | 448.1007 ± 0.0001 | 448.1006 | 449.1080, [M+H]⁺ |
| 44 | C_21_H_26_O_12_ | Plumieride | 21.08 ± 0.04 | 470.1424 ± 0.0004 | 470.1423 | 471.1499, [M+H]⁺ |
| 45 | C_29_H_36_O_15_ | Forsythoside A* | 21.08 ± 0.04 | 624.2046 ± 0.0000 | 624.2054 | 642.2394, [M+NH_4_]⁺ |
| 46 | C_13_H_28_N_6_O_8_ | Zwittermicin A | 22.64 ± 0.05 | 396.1975 ± 0.0006 | 396.1979 | 395.1906, [M-H]¯ |
| 47 | C_20_H_20_O_5_ | Morachalcone A | 23.24 ± 0.06 | 340.1309 ± 0.0003 | 340.1313 | 341.1384, [M+H]⁺ |
| 48 | C_26_H_32_O_11_ | Brusatol | 23.24 ± 0.06 | 520.1943 ± 0.0002 | 520.1945 | 538.2284, [M+NH_4_]⁺ |
| 49 | C_25_H_31_N_3_O_4_ | Lunarine | 24.06 ± 0.03 | 437.2313 ± 0.0001 | 437.2312 | 438.2384, [M+H]⁺ |
| 50 | C_27_H_30_O_14_ | Isofurcatain 7-O-glucoside | 25.01 ± 0.00 | 578.1637 ± 0.0004 | 578.1637 | 579.1708, [M+H]⁺ |
| 51 | C_25_H_24_O_12_ | Apigenin 7-(3'',4''-diacetylglucoside) | 25.96 ± 0.03 | 516.1268 ± 0.0002 | 516.1269 | 517.1342, [M+H]⁺ |
| 52 | C_23_H_24_O_11_ | Luteolin 7,4'-dimethyl ether 3'-glucoside | 25.97 ± 0.01 | 476.1341 ± 0.0001 | 476.1319 | 499.1237, [M+Na]⁺ |
| 53 | C_21_H_20_O_10_ | Neovitexin | 29.87 ± 0.05 | 432.1059 ± 0.0001 | 432.1058 | 433.1135, [M+H]⁺ |
| 54 | C_19_H_29_N_5_O_6_ | Tyr-Asn-Lys | 30.30 ± 0.06 | 423.2123 ± 0.0001 | 423.2118 | 441.2460, [M+NH_4_]⁺ |
| 55 | C_27_H_34_O_11_ | Undulatone | 30.67 ± 0.06 | 534.2073 ± 0.0003 | 534.2065 | 533.2000, [M-H]¯ |
| 56 | C_14_H_19_N_5_O_8_ | His-Asp-Asp | 31.64 ± 0.02 | 385.1224 ± 0.0008 | 385.1216 | 384.1154, [M-H]¯ |
| 57 | C_21_H_18_O_11_ | Baicalin* | 32.01 ± 0.01 | 446.0846 ± 0.0000 | 446.0849 | 447.0918, [M+H]⁺ |
| 58 | C_27_H_34_O_11_ | Forsythin* | 32.31 ± 0.00 | 534.2097 ± 0.0004 | 534.2101 | 552.2445, [M+NH_4_]⁺ |
| 59 | C_16_H_13_NO_4_ | 4-Naphthalimidobutyric Acid | 32.65 ± 0.06 | 283.0830 ± 0.0008 | 283.0819 | 282.0764, [M-H]¯ |
| 60 | C_22_H_20_O_12_ | Hispidulin 7-glucuronide | 33.08 ± 0.03 | 476.0957 ± 0.0001 | 476.0957 | 477.1030, [M+H]⁺ |
| 61 | C_21_H_18_O_10_ | Chrysin 7-glucuronide | 33.44 ± 0.01 | 430.0901 ± 0.0000 | 430.0902 | 431.0974, [M+H]⁺ |
| 62 | C_22_H_20_O_11_ | Wogonin 7-glucuronide | 33.69 ± 0.05 | 460.1007 ± 0.0000 | 460.1008 | 461.1080, [M+H]⁺ |
| 63 | C_18_H_28_N_10_O_4_ | His-His-Arg | 34.21 ± 0.04 | 448.2289 ± 0.0004 | 448.2275 | 447.2216, [M-H]¯ |
| 64 | C_21_H_18_O_11_ | Apigenin 7-glucuronide | 34.37 ± 0.00 | 446.0845 ± 0.0001 | 446.0845 | 447.0918, [M+H]⁺ |
| 65 | C_14_H_28_O_2_ | 9-methyl-tridecanoic acid | 36.13 ± 0.03 | 228.2083 ± 0.0002 | 228.2088 | 227.2008, [M-H]¯ |
| 66 | C_16_H_12_O_6_ | Kaempferide | 36.46 ± 0.04 | 300.0638 ± 0.0001 | 300.0629 | 301.0710, [M+H]⁺ |
| 67 | C_15_H_10_O_5_ | 2'-Hydroxydaidzein | 36.68 ± 0.00 | 270.0531 ± 0.0001 | 270.0530 | 271.0604, [M+H]⁺ |
| 68 | C_18_H_32_O_2_ | 8,11-octadecadienoic acid | 37.73 ± 0.00 | 280.2402 ± 0.0011 | 280.2386 | 279.2336, [M-H]¯ |
| 69 | C_21_H_24_O_6_ | Kadsurin A | 37.82 ± 0.00 | 372.1575 ± 0.0001 | 372.1573 | 390.1916, [M+NH_4_]⁺ |
| 70 | C_21_H_22_O_5_ | 6'',6''-Dimethyl-5''-hydroxy-4'',5''-dihydropyrano [2'',3'':2',3']-4'-hydroxy-6'-methoxychalcone | 37.94 ± 0.01 | 354.1472 ± 0.0004 | 354.1469 | 355.1542, [M+H]⁺ |
| 71 | C_16_H_32_O_2_ | 4-hexyl-decanoic acid | 38.22 ± 0.07 | 256.2392 ± 0.0011 | 256.2382 | 255.2312, [M-H]¯ |
| 72 | C_16_H_12_O_5_ | 5-O-Methylgenistein | 39.51 ± 0.01 | 284.0686 ± 0.0001 | 284.0674 | 285.0758, [M+H]⁺ |
| 73 | C_30_H_18_O_10_ | Sennidin B | 39.53 ± 0.01 | 538.0895 ± 0.0004 | 538.0897 | 539.0971, [M+H]⁺ |
| 74 | C_17_H_14_O_6_ | 5,3'-Dihydroxy-7,4'-dimethoxy-4-phenylcoumarin | 39.88 ± 0.02 | 314.0791 ± 0.0001 | 314.0792 | 315.0863, [M+H]⁺ |
| 75 | C_19_H_18_O_8_ | Skullcapflavone II | 40.19 ± 0.00 | 374.1001 ± 0.0002 | 374.0999 | 375.1075, [M+H]⁺ |
| 76 | C_15_H_22_O_2_ | Eremophilenolide | 45.47 ± 0.04 | 234.1622 ± 0.0001 | 234.1623 | 235.1696, [M+H]⁺ |
| 77 | C_24_H_50_NO_7_P | PE (19:0/0:0) | 46.58 ± 0.01 | 495.3325 ± 0.0000 | 495.3329 | 496.3399, [M+H]⁺ |
| 78 | C_19_H_38_O_4_ | 1-Monopalmitin | 50.89 ± 0.01 | 330.2774 ± 0.0003 | 330.2769 | 331.2846, [M+H]⁺ |
| 79 | C_51_H_84_O_15_ | 1,2-Di-(9Z,12Z,15Z-octadecatrienoyl)-3-(Galactosyl-alpha-1-6-Galactosyl-beta-1)-glycerol | 51.06 ± 0.02 | 936.5809 ± 0.0005 | 936.5810 | 954.6148, [M+NH_4_]⁺ |
| 80 | C_39_H_64_O_5_ | DG (18:4(6Z,9Z,12Z,15Z)/18:2(9Z,12Z)/0:0) | 51.19 ± 0.07 | 612.4751 ± 0.0004 | 612.4772 | 613.4815, [M+H]⁺ |
| 81 | C_45_H_74_O_10_ | 1,2 di-(9Z,12Z,15Z-octadecatrienoyl)-3-O-Beta-D-galactosyl-sn-glycerol | 51.30 ± 0.02 | 774.5282 ± 0.0006 | 774.5282 | 792.5616, [M+NH_4_]⁺ |
| 82 | C_40_H_77_O_13_P | PI (14:0/17:0) | 51.30 ± 0.02 | 796.5102 ± 0.0006 | 796.5090 | 797.5169, [M+H]⁺ |
| 83 | C_43_H_70_O_10_ | MGDG (18:3(9Z,12Z,15Z)/16:3(7Z,10Z,13Z)) | 51.48 ± 0.05 | 746.4973 ± 0.0005 | 746.4969 | 769.4865, [M+Na]⁺ |
| 84 | C_37_H_60_O_5_ | DG (14:1(9Z)/20:5(5Z,8Z,11Z,14Z,17Z)/0:0) | 51.48 ± 0.05 | 584.4443 ± 0.0005 | 584.4443 | 585.4514, [M+H]⁺ |

*Marker components.
