# Supplementary material for: Metabolomics analyses of traditional Chinese medicine formula Shuang Huang Lian by UHPLC-QTOF-MS/MS
Source: Chin Med. 2022 May 30;17:62. doi: 10.1186/s13020-022-00610-x (PMC9150355; doi:10.1186/s13020-022-00610-x)
Supplement: Supplementary file 5 — Additional file 5: Table S2. The chemical components identified only with formulas in SHL granule preparation form. [file 13020_2022_610_MOESM5_ESM.docx]

**Table S2. The chemical components identified only with formulas in SHL granule preparation form (n = 3)**

| **NO** | **Formula** | **t_R_ (min) (Mean ± SD)** | **Observed Mass (Mean ± SD)** | **Mass (MFG)** | **Precursor ion, m/z** |
| --- | --- | --- | --- | --- | --- |
| 1 | C_15_H_14_F_6_NO_2_ | 0.93 ± 0.01 | 354.0926 ± 0.0005 | 354.0907 | 353.0855, [M-H]¯ |
| 2 | C_12_H_17_F_2_N_8_O_3_P_3_ | 0.93 ± 0.01 | 452.0591 ± 0.0001 | 452.0577 | 451.0519, [M-H]¯ |
| 3 | C_6_HFN_4_O_3_ | 0.97 ± 0.01 | 196.0030 ± 0.0001 | 196.0031 | 194.9961, [M-H]¯ |
| 4 | C_26_H_30_F_3_NO_3_P_3_ | 0.97 ± 0.01 | 546.1525 ± 0.0028 | 554.1387 | 553.1331, [M-H]¯ |
| 5 | C_8_H_10_F_3_N_8_O | 1.00 ± 0.04 | 291.0936 ± 0.0004 | 291.0926 | 290.0863, [M-H]¯ |
| 6 | C_15_H_14_F_6_NO | 1.05 ± 0.03 | 338.0977 ± 0.0003 | 338.0964 | 337.0904, [M-H]¯ |
| 7 | C_28_H_57_F_3_NO_3_P_8_Si | 1.30 ± 0.00 | 788.1951 ± 0.0020 | 788.1949 | 787.1889, [M-H]¯ |
| 8 | C_26_H_30_F_2_N_6_O_10_ | 1.34 ± 0.00 | 624.2003 ± 0.0014 | 624.1997 | 623.1939, [M-H]¯ |
| 9 | C_9_H_14_F_9_N_3_OP | 1.38 ± 0.02 | 382.0736 ± 0.0004 | 382.0727 | 383.0809, [M+H]⁺ |
| 10 | C_13_H_20_F_2_O_8_ | 1.58 ± 0.00 | 342.1141 ± 0.0006 | 342.1134 | 341.1064, [M-H]¯ |
| 11 | C_30_H_52_FN_9_OP_8_ | 1.66 ± 0.00 | 821.2162 ± 0.0014 | 821.2162 | 820.2107, [M-H]¯ |
| 12 | C_18_H_20_FN_5_O_3_P | 1.71 ± 0.00 | 404.1285 ± 0.0016 | 404.1279 | 403.1218, [M-H]¯ |
| 13 | C_24_H_37_F_14_N_3_O_7_PSi | 1.71 ± 0.00 | 804.1923 ± 0.0010 | 804.1936 | 803.1842, [M-H]¯ |
| 14 | C_11_H_19_F_2_N_7_O_4_P_3_ | 1.89 ± 0.01 | 444.0672 ± 0.0011 | 444.0693 | 443.0591, [M-H]¯ |
| 15 | C_28_H_39_F_12_N_5_P_3_ | 2.12 ± 0.01 | 766.2260 ± 0.0016 | 766.2253 | 765.2191, [M-H]¯ |
| 16 | C_34_H_46_N_5_O_24_ | 2.15 ± 0.03 | 908.2538 ± 0.0024 | 908.2512 | 907.2485, [M-H]¯ |
| 17 | C_18_H_20_F_6_N_8_ | 2.22 ± 0.06 | 462.1706 ± 0.0013 | 462.1695 | 461.1641, [M-H]¯ |
| 18 | C_35_H_42_F_14_N_18_P_2_Si | 2.42 ± 0.07 | 1070.2886 ± 0.0040 | 1070.2861 | 1069.2847, [M-H]¯ |
| 19 | C_26_H_30_F_2_N_6_O_10_ | 2.44 ± 0.04 | 624.2015 ± 0.0025 | 624.2003 | 623.1955, [M-H]¯ |
| 20 | C_10_H_20_F_5_N_2_O_3_P | 2.47 ± 0.00 | 342.1142 ± 0.0012 | 342.1134 | 341.1060, [M-H]¯ |
| 21 | C_41_H_54_F_3_N_11_O_13_P_3_Si | 2.47 ± 0.07 | 1086.2804 ± 0.0035 | 1086.2812 | 1085.2764, [M-H]¯ |
| 22 | C_8_H_12_O_3_ | 2.65 ± 0.01 | 156.0780 ± 0.0004 | 156.0777 | 155.0709, [M-H]¯ |
| 23 | C_23_H_18_F_2_N_2_P_4_ | 3.06 ± 0.01 | 484.0391 ± 0.0005 | 484.0387 | 483.0319, [M-H]¯ |
| 24 | C_14_H_24_F_4_OP_2_ | 3.18 ± 0.00 | 346.1237 ± 0.0005 | 346.1233 | 345.1167, [M-H]¯ |
| 25 | C_13_H_16_F_6_N | 3.22 ± 0.04 | 300.1188 ± 0.0006 | 300.1202 | 299.1120, [M-H]¯ |
| 26 | C_25_H_30_N_12_O_4_P_2_ | 3.25 ± 0.01 | 624.2007 ± 0.0004 | 624.2013 | 623.1929, [M-H]¯ |
| 27 | C_18_H_17_FN_6_O_3_P_2_ | 3.43 ± 0.06 | 446.0842 ± 0.0004 | 446.0819 | 222.0343, [M-2H]²¯ |
| 28 | C_20_H_22_N_2_O_6_P_2_ | 3.71 ± 0.04 | 448.0965 ± 0.0004 | 448.0980 | 447.0897, [M-H]¯ |
| 29 | C_12_H_17_F_3_N_2_O_3_ | 4.29 ± 0.09 | 294.1195 ± 0.0004 | 294.1196 | 293.1125, [M-H]¯ |
| 30 | C_20_H_33_F_7_N_12_P_2_ | 4.29 ± 0.09 | 636.2319 ± 0.0003 | 636.2321 | 635.2248, [M-H]¯ |
| 31 | C_29_H_38_F_6_O_3_P_2_ | 4.29 ± 0.09 | 610.2193 ± 0.0009 | 610.2192 | 609.2128, [M-H]¯ |
| 32 | C_11_H_12_N_5_O_3_ | 4.29 ± 0.09 | 262.0936 ± 0.0004 | 262.0938 | 261.0865, [M-H]¯ |
| 33 | C_28_H_38_N_2_O_10_P_2_ | 5.7 ± 0.1 | 624.2021 ± 0.0016 | 624.2001 | 623.1931, [M-H]¯ |
| 34 | C_41_H_36_F_16_N_2_S | 6.91 ± 0.05 | 892.2359 ± 0.0001 | 892.2347 | 891.2288, [M-H]¯ |
| 35 | C_27_H_39_N_4_O_7_P_3_ | 7.00 ± 0.05 | 624.2012 ± 0.0002 | 624.2003 | 623.1937, [M-H]¯ |
| 36 | C_55_H_60_F_2_NO_5_P_8_ | 8.15 ± 0.04 | 1100.2321 ± 0.0004 | 1100.2317 | 551.1232, [M+2H]²⁺ |
| 37 | C_17_H_26_FN_5_OP | 8.34 ± 0.04 | 366.1866 ± 0.0006 | 366.1859 | 365.1797, [M-H]¯ |
| 38 | C_22_H_18_F_2_N_16_O_4_ | 8.34 ± 0.04 | 608.1702 ± 0.0000 | 608.1703 | 607.1630, [M-H]¯ |
| 39 | C_22_H_12_N_6_O_5_P_2_Si | 9.34 ± 0.05 | 530.0107 ± 0.0011 | 530.0110 | 529.0041, [M-H]¯ |
| 40 | C_30_H_3_F_5_N_5_O_2_ | 9.78 ± 0.06 | 560.0198 ± 0.0008 | 560.0205 | 559.0134, [M-H]¯ |
| 41 | C_20_H_18_F_5_N_2_O_3_P | 9.85 ± 0.06 | 460.0968 ± 0.0006 | 460.0961 | 459.0893, [M-H]¯ |
| 42 | C_29_H_54_FN_10_O_5_P_7_Si_3_ | 9.89 ± 0.01 | 942.1687 ± 0.0027 | 942.1723 | 941.1661, [M-H]¯ |
| 43 | C_27_H_29_F_4_N_2_O_3_P | 10.50 ± 0.03 | 536.1854 ± 0.0014 | 536.1822 | 535.1779, [M-H]¯ |
| 44 | C_22_H_17_FN_6_OP | 11.10 ± 0.04 | 431.1174 ± 0.0008 | 431.1176 | 430.1111, [M-H]¯ |
| 45 | C_20_H_40_FP_5_ | 11.23 ± 0.08 | 454.1802 ± 0.0005 | 454.1792 | 453.1732, [M-H]¯ |
| 46 | C_20_H_19_F_3_O_9_ | 11.49 ± 0.08 | 460.0971 ± 0.0008 | 460.0979 | 459.0892, [M-H]¯ |
| 47 | C_35_H_42_N_15_O_7_P_2_ | 11.60 ± 0.09 | 846.2865 ± 0.0029 | 846.2871 | 845.2816, [M-H]¯ |
| 48 | C_14_H_10_N_3_O_4_ | 12.1 ± 0.1 | 284.0666 ± 0.0006 | 284.0662 | 283.0596, [M-H]¯ |
| 49 | C_48_H_68_F_12_O_6_P_4_ | 12.8 ± 0.1 | 1092.3778 ± 0.0006 | 1092.3820 | 1091.3703, [M-H]¯ |
| 50 | C_19_H_30_F_5_N_2_O_2_P | 13.90 ± 0.08 | 444.1964 ± 0.0005 | 444.1958 | 443.1894, [M-H]¯ |
| 51 | C_23_H_39_O_7_P_3_ | 15.05 ± 0.05 | 520.1908 ± 0.0004 | 520.1916 | 519.1838, [M-H]¯ |
| 52 | C_52_H_58_F_11_O_7_PSi | 15.09 ± 0.01 | 1062.3537 ± 0.0045 | 1062.3636 | 1061.3507, [M-H]¯ |
| 53 | C_21_H_28_N_3_O_2_P_2_ | 15.2 ± 0.2 | 416.1654 ± 0.0008 | 416.1642 | 415.1575, [M-H]¯ |
| 54 | C_19_H_24_N_3_O_2_P_2_ | 15.66 ± 0.02 | 388.1344 ± 0.0003 | 388.1345 | 387.1274, [M-H]¯ |
| 55 | C_41_H_68_F_2_N_4_O_2_P_8_ | 15.78 ± 0.08 | 934.3215 ± 0.0001 | 934.3207 | 935.3286, [M+H]⁺ |
| 56 | C_19_H_25_FN_6_OP_2_ | 16.10 ± 0.00 | 434.1543 ± 0.0008 | 434.1556 | 433.1475, [M-H]¯ |
| 57 | C_27_H_45_P_5_ | 18.24 ± 0.03 | 524.2213 ± 0.0011 | 524.2213 | 523.2147, [M-H]¯ |
| 58 | C_27_H_29_F_4_N_2_O_2_P | 18.42 ± 0.01 | 520.1912 ± 0.0013 | 520.1911 | 519.1831, [M-H]¯ |
| 59 | C_24_H_32_F_5_N_5_O_4_P | 19.23 ± 0.05 | 580.2113 ± 0.0007 | 580.2111 | 579.2042, [M-H]¯ |
| 60 | C_16_H_18_N_6_O_3_ | 20.0 ± 0.1 | 342.1443 ± 0.0004 | 342.1445 | 341.1374, [M-H]¯ |
| 61 | C_53_H_69_F_4_NO_7_P_5_ | 23.24 ± 0.06 | 1062.3703 ± 0.0000 | 1062.3700 | 1063.3777, [M+H]⁺ |
| 62 | C_25_H_33_F_12_P | 23.44 ± 0.01 | 592.2129 ± 0.0004 | 592.2126 | 593.2201, [M+H]⁺ |
| 63 | C_26_H_32_FNO_7_P | 23.91 ± 0.07 | 520.1904 ± 0.0005 | 520.1904 | 519.1828, [M-H]¯ |
| 64 | C_21_H_39_F_5_N_4_P_2_ | 24.99 ± 0.01 | 504.2572 ± 0.0002 | 504.2569 | 527.2462, [M+Na]⁺ |
| 65 | C_18_H_21_F_3_O_3_ | 26.83 ± 0.07 | 342.1440 ± 0.0003 | 342.1442 | 341.1370, [M-H]¯ |
| 66 | C_17_H_21_F_3_O_3_ | 26.83 ± 0.07 | 330.1441 ± 0.0004 | 330.1442 | 329.1371, [M-H]¯ |
| 67 | C_25_H_32_N_5_O_2_P_4_ | 27.50 ± 0.06 | 558.1490 ± 0.0001 | 558.1508 | 559.1573, [M+H]⁺ |
| 68 | C_31_H_48_F_8_O_6_P_2_Si | 27.66 ± 0.07 | 758.2576 ± 0.0002 | 758.2575 | 757.2505, [M-H]¯ |
| 69 | C_26_H_36_F_8_N_14_P_2_ | 29.72 ± 0.06 | 758.2575 ± 0.0011 | 758.2553 | 757.2506, [M-H]¯ |
| 70 | C_27_H_27_F_3_N_13_O_4_ | 29.98 ± 0.03 | 654.2265 ± 0.0003 | 654.2259 | 653.2186, [M-H]¯ |
| 71 | C_21_H_24_F_7_N_12_ | 32.38 ± 0.02 | 577.2132 ± 0.0008 | 577.2119 | 576.2063, [M-H]¯ |
| 72 | C_29_H_28_N_9_O_4_P | 32.4 ± 0.1 | 597.2020 ± 0.0008 | 597.2017 | 596.1954, [M-H]¯ |
| 73 | C_22_H_30_N_9_O_10_ | 32.40 ± 0.05 | 580.2125 ± 0.0013 | 580.2111 | 579.2060, [M-H]¯ |
| 74 | C_19_H_23_F_3_O_4_ | 32.40 ± 0.05 | 372.1556 ± 0.0008 | 372.1553 | 371.1489, [M-H]¯ |
| 75 | C_30_H_43_F_6_N_5_OP_3_ | 32.40 ± 0.05 | 696.2591 ± 0.0008 | 696.2587 | 695.2524, [M-H]¯ |
| 76 | C_23_H_29_F_2_N_8_O_3_P | 32.40 ± 0.05 | 534.2080 ± 0.0008 | 534.2067 | 533.2001, [M-H]¯ |
| 77 | C_48_H_58_F_6_O_6_P_2_ | 32.40 ± 0.05 | 906.3619 ± 0.0011 | 906.3616 | 905.3540, [M-H]¯ |
| 78 | C_57_H_64_F_9_O_7_PSi | 32.41 ± 0.00 | 1090.4031 ± 0.0005 | 1090.4019 | 1091.4096, [M+H]⁺ |
| 79 | C_22_H_25_F_9_N_16_O_9_Si_2_ | 33.45 ± 0.01 | 884.1380 ± 0.0001 | 884.1377 | 885.1457, [M+H]⁺ |
| 80 | C_12_H_27_N | 35.17 ± 0.02 | 185.2144 ± 0.0000 | 185.2144 | 186.2217, [M+H]⁺ |
| 81 | C_19_H_23_F_3_O_4_ | 35.30 ± 0.01 | 372.1557 ± 0.0005 | 372.1549 | 371.1488, [M-H]¯ |
| 82 | C_30_H_37_FN_9_O_4_P_2_ | 36.59 ± 0.01 | 668.2419 ± 0.0014 | 668.2402 | 667.2355, [M-H]¯ |
| 83 | C_25_H_15_N_10_OP_3_ | 36.68 ± 0.00 | 564.0637 ± 0.0001 | 564.0635 | 565.0709, [M+H]⁺ |
| 84 | C_14_H_28_N_3_O | 37.17 ± 0.01 | 254.2236 ± 0.0008 | 254.2228 | 253.2168, [M-H]¯ |
| 85 | C_59_H_93_N_4_P_7_ | 37.41 ± 0.01 | 1074.5543 ± 0.0006 | 1074.5531 | 1073.5468, [M-H]¯ |
| 86 | C_31_H_45_F_2_N_4_O_7_P_2_ | 37.49 ± 0.00 | 685.2733 ± 0.0001 | 685.2727 | 686.2804, [M+H]⁺ |
| 87 | C_16_H_28_N_3_O_2_S | 38.54 ± 0.00 | 326.1900 ± 0.0003 | 326.1899 | 325.1825, [M-H]¯ |
| 88 | C_13_H_31_FN_3_O_4_Si | 39.39 ± 0.09 | 340.2064 ± 0.0008 | 340.2051 | 339.1984, [M-H]¯ |
| 89 | C_27_H_28_N_2_O_4_ | 42.36 ± 0.00 | 444.2047 ± 0.0000 | 444.2049 | 467.1939, [M+Na]⁺ |
| 90 | C_32_H_46_F_5_N_9_OP | 44.00 ± 0.02 | 698.3485 ± 0.0000 | 698.3463 | 699.3557, [M+H]⁺ |
| 91 | C_26_H_50_NO_7_P | 45.58 ± 0.00 | 519.3323 ± 0.0000 | 519.3324 | 520.3397, [M+H]⁺ |
| 92 | C_29_H_51_F_3_N_4_P_4_ | 50.49 ± 0.01 | 636.2985 ± 0.0006 | 636.2975 | 637.3055, [M+H]⁺ |
| 93 | C_27_H_42_F_2_NO_2_ | 51.32 ± 0.01 | 450.3172 ± 0.0019 | 450.3164 | 449.3120, [M-H]¯ |
| 94 | C_30_H_48_F_2_NO_2_ | 51.40 ± 0.02 | 492.3642 ± 0.0021 | 492.3619 | 491.3560, [M-H]¯ |
