# Supplementary material for: Metabolomics analyses of traditional Chinese medicine formula Shuang Huang Lian by UHPLC-QTOF-MS/MS
Source: Chin Med. 2022 May 30;17:62. doi: 10.1186/s13020-022-00610-x (PMC9150355; doi:10.1186/s13020-022-00610-x)
Supplement: Supplementary file 6 — Additional file 6: Table S3. The chemical components identified with both names and formulas in SHL oral liquid preparation form. [file 13020_2022_610_MOESM6_ESM.docx]

**Table S3. The chemical components identified with both names and formulas in SHL oral liquid preparation form (n = 3)**

| **No.** | **Formula** | **Name** | **t_R_ (min) (Mean ± SD)** | **Observed Mass (Mean ± SD)** | **Database Mass** | **Precursor ion, m/z** |
| --- | --- | --- | --- | --- | --- | --- |
| 1 | C_30_H_26_O_10_ | Guibourtinidol-(4alpha-6)-catechin | 0.98 ± 0.00 | 546.1538 ± 0.0011 | 546.1524 | 545.1473, [M-H]¯ |
| 2 | C_11_H_17_NO_8_ | 2,7-Anhydro-alpha-N-acetylneuraminic acid | 1.03 ± 0.00 | 291.0937 ± 0.0004 | 291.0926 | 290.0867, [M-H]¯ |
| 3 | C_26_H_25_FO_8_S | Sulindac sulfide glucuronide | 1.08 ± 0.00 | 516.1227 ± 0.0013 | 516.1234 | 257.0555, [M-2H]²¯ |
| 4 | C_23_H_20_O_5_ | 5-O-Methylchamanetin | 1.08 ± 0.00 | 376.1330 ± 0.0013 | 376.1314 | 375.1266, [M-H]¯ |
| 5 | C_14_H_19_N_2_O_7_P | N1-(5-Phospho-a-D-ribosyl)-5,6-dimethylbenzimidazole | 1.37 ± 0.02 | 358.0925 ± 0.0001 | 358.0930 | 381.0818, [M+Na]⁺ |
| 6 | C_15_H_16_N_4_O_7_ | His-Ser-OH | 1.45 ± 0.02 | 364.1008 ± 0.0001 | 364.0988 | 365.1079, [M+H]⁺ |
| 7 | C_21_H_20_O_13_ | Tagetiin | 1.68 ± 0.03 | 480.0884 ± 0.0008 | 480.0916 | 479.0817, [M-H]¯ |
| 8 | C_18_H_18_N_6_O_5_S_2_ | Cefamandole | 1.70 ± 0.00 | 462.0778 ± 0.0008 | 462.0774 | 461.0706, [M-H]¯ |
| 9 | C_24_H_20_O_6_ | Calomelanol C | 1.70 ± 0.00 | 404.1289 ± 0.0005 | 404.1279 | 403.1219, [M-H]¯ |
| 10 | C_33_H_40_O_23_ | Gossypetin 3-sophoroside-8-glucoside | 1.70 ± 0.00 | 804.1919 ± 0.0010 | 804.1936 | 803.1857, [M-H]¯ |
| 11 | C_13_H_15_N_3_O_2_ | Acetyltryptophanamide | 1.85 ± 0.00 | 245.1156 ± 0.0007 | 245.1164 | 268.1054, [M+Na]⁺ |
| 12 | C_23_H_40_N_2_O_18_ | 3'-Sialyllactosamine | 1.92 ± 0.00 | 632.2264 ± 0.0002 | 632.2276 | 631.2188, [M-H]¯ |
| 13 | C_27_H_26_O_7_ | Euchrenone b3 | 2.11 ± 0.02 | 462.1696 ± 0.0008 | 462.1695 | 461.1630, [M-H]¯ |
| 14 | C_8_H_10_O_3_ | 3,4-Dihydroxyphenyl ethanol | 2.91 ± 0.02 | 154.0622 ± 0.0008 | 154.0617 | 153.0555, [M-H]¯ |
| 15 | C_28_H_16_O_5_ | Naphthofluorescein | 4.63 ± 0.01 | 432.1028 ± 0.0012 | 432.1031 | 431.0961, [M-H]¯ |
| 16 | C_24_H_27_NO_6_S | Troglitazone quinone | 5.11 ± 0.04 | 457.1553 ± 0.0008 | 457.1547 | 456.1484, [M-H]¯ |
| 17 | C_13_H_22_N_4_O_7_S | Met-Asp-Asn | 7.03 ± 0.03 | 378.1205 ± 0.0002 | 378.1197 | 377.1136, [M-H]¯ |
| 18 | C_21_H_26_N_4_O_8_ | Trp-Glu-Glu | 7.86 ± 0.01 | 462.1739 ± 0.0001 | 462.1751 | 480.2075, [M+NH_4_]⁺ |
| 19 | C_16_H_18_O_9_ | Chlorogenic Acid* | 8.16 ± 0.02 | 354.0953 ± 0.0001 | 354.0951 | 355.1025, [M+H]⁺ |
| 20 | C_9_H_6_O_3_ | Umbelliferone | 8.16 ± 0.02 | 162.0315 ± 0.0001 | 162.0316 | 163.0387, [M+H]⁺ |
| 21 | C_10_H_12_O_5_ | Danielone | 9.69 ± 0.00 | 212.0681 ± 0.0000 | 212.0682 | 213.0753, [M+H]⁺ |
| 22 | C_16_H_22_O_10_ | Geniposidic acid | 9.69 ± 0.00 | 374.1213 ± 0.0001 | 374.1213 | 397.1108, [M+Na]⁺ |
| 23 | C_8_H_6_O_3_ | Piperonal | 9.69 ± 0.00 | 150.0317 ± 0.0001 | 150.0319 | 151.039, [M+H]⁺ |
| 24 | C_10_H_10_O_4_ | Methyl caffeate | 9.69 ± 0.00 | 194.0581 ± 0.0000 | 194.0582 | 195.0653, [M+H]⁺ |
| 25 | C_32_H_40_O_18_ | Isoliquiritigenin 4'-O-glucoside 4-O-apiofuranosyl-(1'''-2''')-glucoside | 9.69 ± 0.00 | 712.2206 ± 0.0002 | 712.2215 | 713.2282, [M+H]⁺ |
| 26 | C_33_H_40_O_21_ | Kaempferol 3-glucosyl-(1->2)-galactosyl-(1->2)-glucoside | 9.74 ± 0.07 | 772.2015 ± 0.0006 | 772.2014 | 773.2095, [M+H]⁺ |
| 27 | C_19_H_34_N_2_O_2_S_4_ | AN-7 | 10.24 ± 0.01 | 450.1505 ± 0.0002 | 450.1490 | 449.1428, [M-H]¯ |
| 28 | C_16_H_18_O_8_ | p-Coumaroyl quinic acid | 10.50 ± 0.02 | 338.1004 ± 0.0002 | 338.1002 | 339.1073, [M+H]⁺ |
| 29 | C_22_H_27_F_3_O_4_S | Fluticasone | 10.61 ± 0.01 | 444.1600 ± 0.0004 | 444.1595 | 443.1530, [M-H]¯ |
| 30 | C_17_H_27_N_5_O_6_ | Glu-His-Leu | 11.05 ± 0.01 | 397.1972 ± 0.0000 | 397.1961 | 420.1866, [M+Na]⁺ |
| 31 | C_26_H_24_O_7_ | Cycloartomunoxanthone | 11.11 ± 0.05 | 448.1544 ± 0.0011 | 448.1534 | 447.1479, [M-H]¯ |
| 32 | C_15_H_10_O_4_ | 5,7-Dihydroxyisoflavone | 11.38 ± 0.04 | 254.0565 ± 0.0013 | 254.0539 | 253.0502, [M-H]¯ |
| 33 | C_11_H_14_O_5_ | Genipin | 11.53 ± 0.05 | 226.0829 ± 0.0006 | 226.0824 | 225.0760, [M-H]¯ |
| 34 | C_15_H_26_N_6_O_6_ | Asp-Arg-Pro | 11.72 ± 0.00 | 386.1924 ± 0.0008 | 386.1916 | 385.1854, [M-H]¯ |
| 35 | C_16_H_28_N_6_O_8_ | Arg-Glu-Glu | 11.72 ± 0.00 | 432.1971 ± 0.0007 | 432.1960 | 431.1898, [M-H]¯ |
| 36 | C_16_H_22_O_9_ | Tarennoside | 11.75 ± 0.01 | 358.1263 ± 0.0004 | 358.1264 | 359.1333, [M+H]⁺ |
| 37 | C_10_H_12_O_4_ | Paeonilactone B | 11.75 ± 0.01 | 196.0734 ± 0.0001 | 196.0732 | 197.0807, [M+H]⁺ |
| 38 | C_28_H_48_N_2_O_21_ | MID58090:O-b-D-Gal-(1-3)-O-[O-b-D-Gal-(1-4)-2-(acetylamino)-2-deoxy-b-D-Glc-(1-6)]-2-(acetylamino | 11.84 ± 0.06 | 748.2734 ± 0.0027 | 748.2729 | 747.2676, [M-H]¯ |
| 39 | C_33_H_32_O_7_ | Leucadenone C | 11.88 ± 0.01 | 540.2167 ± 0.0013 | 540.2162 | 539.2108, [M-H]¯ |
| 40 | C_11_H_16_O_5_ | Depdecin | 12.01 ± 0.06 | 228.0989 ± 0.0009 | 228.0994 | 227.0922, [M-H]¯ |
| 41 | C_16_H_12_O_5_ | Negletein | 12.10 ± 0.08 | 284.0669 ± 0.0010 | 284.0662 | 283.0602, [M-H]¯ |
| 42 | C_40_H_50_O_20_ | Sempervirenoside B | 12.20 ± 0.01 | 850.2896 ± 0.0001 | 850.2895 | 868.3235, [M+NH_4_]⁺ |
| 43 | C_21_H_36_N_2_O_14_ | Galβ1-3GalNAcα-Thr | 12.33 ± 0.00 | 540.2200 ± 0.0001 | 540.2178 | 563.2095, [M+Na]⁺ |
| 44 | C_25_H_31_NO_10_ | 6β-Naloxol-3-glucuronide | 13.20 ± 0.06 | 505.1920 ± 0.0008 | 505.1910 | 504.1853, [M-H]¯ |
| 45 | C_9_H_8_O_3_ | cis-2-Hydroxycinnamate | 14.63 ± 0.00 | 164.0470 ± 0.0001 | 164.0469 | 165.0542, [M+H]⁺ |
| 46 | C17H24O11 | Gardenoside | 14.63 ± 0.00 | 404.1315 ± 0.0001 | 404.1319 | 427.1208, [M+Na]⁺ |
| 47 | C_10_H_10_O_5_ | Vanilpyruvic acid | 14.63 ± 0.00 | 210.0530 ± 0.0003 | 210.0530 | 211.0602, [M+H]⁺ |
| 48 | C_50_H_34_O_11_ | Catechin Pentabenzoate | 14.63 ± 0.00 | 810.2095 ± 0.0009 | 810.2101 | 828.2425, [M+NH_4_]⁺ |
| 49 | C_35_H_44_O_23_ | Limocitrol 3-[alpha-L-arabinopyranosyl-(1-3) [galactosyl-(1-6)]-galactoside] | 14.63 ± 0.00 | 832.2280 ± 0.0003 | 832.2291 | 833.2369, [M+H]⁺ |
| 50 | C_20_H_27_N_5_O_6_ | Thr-Gln-Trp | 15.11 ± 0.02 | 433.1945 ± 0.0001 | 433.1943 | 434.2016, [M+H]⁺ |
| 51 | C_20_H_24_N_4_O_6_ | Pro-Trp-Asp | 15.15 ± 0.04 | 416.1677 ± 0.0001 | 416.1696 | 439.1571, [M+Na]⁺ |
| 52 | C_18_H_20_N_2_O_10_ | p-Hydroxyphenobarbital glucuronide | 15.76 ± 0.04 | 424.1116 ± 0.0017 | 424.1105 | 423.1057, [M-H]¯ |
| 53 | C_22_H_26_O_9_ | Neoraufuracin | 16.15 ± 0.01 | 434.1553 ± 0.0008 | 434.1556 | 433.1483, [M-H]¯ |
| 54 | C_15_H_21_N_5_O_8_ | Asp-Glu-His | 16.31 ± 0.02 | 399.1387 ± 0.0010 | 399.1390 | 417.1732, [M+NH_4_]⁺ |
| 55 | C_16_H_31_N_5_O_6_ | Asp-Lys-Lys | 16.32 ± 0.01 | 389.2284 ± 0.0001 | 389.2274 | 412.2175, [M+Na]⁺ |
| 56 | C_16_H_18_N_6_O_4_ | 2-Phenylaminoadenosine | 16.43 ± 0.00 | 358.1352 ± 0.0053 | 358.1387 | 357.1313, [M-H]¯ |
| 57 | C_27_H_30_O_16_ | Rutin | 17.72 ± 0.02 | 610.1524 ± 0.0001 | 610.1537 | 611.1596, [M+H]⁺ |
| 58 | C_15_H_10_O_7_ | Hypolaetin | 17.72 ± 0.02 | 302.0423 ± 0.0001 | 302.0426 | 303.0495, [M+H]⁺ |
| 59 | C_21_H_18_O_12_ | Luteolin 3'-glucuronide | 18.17 ± 0.04 | 462.0805 ± 0.0001 | 462.0797 | 463.0868, [M+H]⁺ |
| 60 | C_21_H_20_O_11_ | Luteolin-7-O-glucoside* | 19.30 ± 0.06 | 448.1009 ± 0.0002 | 448.1006 | 449.1083, [M+H]⁺ |
| 61 | C_20_H_27_NO_11_ | Amygdalin | 20.74 ± 0.02 | 457.1584 ± 0.0001 | 457.1585 | 458.1658, [M+H]⁺ |
| 62 | C_21_H_26_O_12_ | Plumieride | 21.08 ± 0.03 | 470.1426 ± 0.0001 | 470.1423 | 471.1499, [M+H]⁺ |
| 63 | C_29_H_36_O_15_ | Forsythoside A* | 21.08 ± 0.03 | 624.2057 ± 0.0002 | 624.2054 | 642.2395, [M+NH_4_]⁺ |
| 64 | C_9_H_14_N_3_O_7_P | dCMP | 21.11 ± 0.00 | 307.0581 ± 0.0001 | 307.0569 | 325.0916, [M+NH_4_]⁺ |
| 65 | C_13_H_28_N_6_O_8_ | Zwittermicin A | 22.67 ± 0.07 | 396.1970 ± 0.0010 | 396.1979 | 395.1904, [M-H]¯ |
| 66 | C_28_H_31_ClN_2_O_2_ | Desmethylloperamide | 22.87 ± 0.00 | 462.2074 ± 0.0013 | 462.2064 | 461.2004, [M-H]¯ |
| 67 | C_20_H_20_O_5_ | Morachalcone A | 23.24 ± 0.03 | 340.1310 ± 0.0001 | 340.1313 | 341.1383, [M+H]⁺ |
| 68 | C_26_H_32_O_11_ | Brusatol | 23.24 ± 0.03 | 520.1939 ± 0.0002 | 520.1945 | 538.2278, [M+NH_4_]⁺ |
| 69 | C_28_H_37_FO_7_ | βmethasone dipropionate | 24.47 ± 0.02 | 504.2529 ± 0.0016 | 504.2530 | 503.2466, [M-H]¯ |
| 70 | C_27_H_30_O_14_ | Isofurcatain 7-O-glucoside | 25.08 ± 0.04 | 578.1632 ± 0.0004 | 578.1637 | 579.17, [M+H]⁺ |
| 71 | C_25_H_24_O_12_ | Apigenin 7-(3'',4''-diacetylglucoside) | 25.99 ± 0.03 | 516.1265 ± 0.0001 | 516.1269 | 517.1338, [M+H]⁺ |
| 72 | C_21_H_20_O_10_ | Isovitexin | 29.87 ± 0.02 | 432.1056 ± 0.0003 | 432.1058 | 433.1126, [M+H]⁺ |
| 73 | C_27_H_34_O_11_ | Undulatone | 30.69 ± 0.06 | 534.1992 ± 0.0001 | 534.2067 | 533.1993, [M-H]¯ |
| 74 | C_21_H_18_O_11_ | Baicalin* | 32.01 ± 0.00 | 446.0849 ± 0.0001 | 446.0849 | 447.092, [M+H]⁺ |
| 75 | C_27_H_34_O_11_ | Forsythin* | 32.32 ± 0.01 | 534.2097 ± 0.0002 | 534.2101 | 552.2442, [M+NH_4_]⁺ |
| 76 | C_21_H_22_O_5_ | Xanthogalenol | 32.32 ± 0.01 | 354.1469 ± 0.0004 | 354.1471 | 355.1545, [M+H]⁺ |
| 77 | C_22_H_20_O_12_ | Hispidulin 7-glucuronide | 33.10 ± 0.00 | 476.0955 ± 0.0001 | 476.0957 | 477.1027, [M+H]⁺ |
| 78 | C_21_H_18_O_10_ | Chrysin 7-glucuronide | 33.46 ± 0.03 | 430.0906 ± 0.0000 | 430.0902 | 431.098, [M+H]⁺ |
| 79 | C_22_H_20_O_11_ | Wogonin 7-glucuronide | 33.68 ± 0.01 | 460.1009 ± 0.0003 | 460.1008 | 461.108, [M+H]⁺ |
| 80 | C_12_H_24_N_6_O_4_ | Ala-Arg-Ala | 34.21 ± 0.02 | 316.1864 ± 0.0000 | 316.1865 | 315.1791, [M-H]¯ |
| 81 | C_21_H_18_O_11_ | Apigenin 7-glucuronide | 34.38 ± 0.01 | 446.0849 ± 0.0002 | 446.0845 | 447.0923, [M+H]⁺ |
| 82 | C_30_H_44_O_2_ | Demethylphylloquinone | 36.29 ± 0.01 | 436.3352 ± 0.0006 | 436.3340 | 437.3429, [M+H]⁺ |
| 83 | C_16_H_12_O_6_ | Kaempferide | 36.46 ± 0.01 | 300.0644 ± 0.0006 | 300.0629 | 301.0721, [M+H]⁺ |
| 84 | C_15_H_10_O_5_ | Baicalein | 36.68 ± 0.02 | 270.0535 ± 0.0007 | 270.0530 | 271.0603, [M+H]⁺ |
| 85 | C_21_H_24_O_6_ | Kadsurin A | 37.84 ± 0.00 | 372.1581 ± 0.0012 | 372.1573 | 390.1926, [M+NH_4_]⁺ |
| 86 | C_21_H_22_O_5_ | Xanthohumol | 37.98 ± 0.00 | 354.1476 ± 0.0011 | 354.1469 | 355.1541, [M+H]⁺ |
| 87 | C_21_H_26_O_3_ | 2-Hydroxymestranol | 38.59 ± 0.02 | 326.1889 ± 0.0006 | 326.1899 | 325.1815, [M-H]¯ |
| 88 | C_16_H_12_O_5_ | Wogonin | 39.51 ± 0.01 | 284.0695 ± 0.0005 | 284.0674 | 285.0764, [M+H]⁺ |
| 89 | C_30_H_18_O_10_ | Sennidin B | 39.57 ± 0.01 | 538.0916 ± 0.0008 | 538.0897 | 539.0996, [M+H]⁺ |
| 90 | C_17_H_14_O_6_ | 5,3'-Dihydroxy-7,4'-dimethoxy-4-phenylcoumarin | 39.88 ± 0.00 | 314.0801 ± 0.0011 | 314.0792 | 315.0884, [M+H]⁺ |
| 91 | C_19_H_18_O_8_ | Skullcapflavone II | 40.17 ± 0.01 | 374.1017 ± 0.0010 | 374.0999 | 375.1099, [M+H]⁺ |
| 92 | C_15_H_22_O_2_ | Eremophilenolide | 45.45 ± 0.00 | 234.1623 ± 0.0006 | 234.1623 | 235.17, [M+H]⁺ |
| 93 | C_29_H_44_O_9_ | rhodexin A | 45.56 ± 0.00 | 536.2974 ± 0.0009 | 536.2945 | 537.3054, [M+H]⁺ |
| 94 | C_24_H_50_NO_7_P | PE (19:0/0:0) | 46.59 ± 0.00 | 495.3329 ± 0.0013 | 495.3329 | 496.3392, [M+H]⁺ |
| 95 | C_19_H_38_O_4_ | 1-Monopalmitin | 50.89 ± 0.00 | 330.2782 ± 0.0000 | 330.2769 | 331.2855, [M+H]⁺ |
| 96 | C_51_H_84_O_15_ | 1,2-Di-(9Z,12Z,15Z-octadecatrienoyl)-3-(Galactosyl-alpha-1-6-Galactosyl-beta-1)-glycerol | 51.10 ± 0.02 | 936.5844 ± 0.0008 | 936.5810 | 959.5726, [M+Na]⁺ |
| 97 | C_45_H_74_O_10_ | 1,2 di-(9Z,12Z,15Z-octadecatrienoyl)-3-O-Beta-D-galactosyl-sn-glycerol | 51.34 ± 0.04 | 774.5315 ± 0.0006 | 774.5282 | 792.5645, [M+NH_4_]⁺ |
| 98 | C_47_H_72_O_10_ | MGDG (20:5(5Z,8Z,11Z,14Z,17Z)/18:4(6Z,9Z,12Z,15Z)) | 51.34 ± 0.04 | 796.5133 ± 0.0003 | 796.5090 | 797.5204, [M+H]⁺ |

*Q-markers
