# Supplementary material for: Metabolomics analyses of traditional Chinese medicine formula Shuang Huang Lian by UHPLC-QTOF-MS/MS
Source: Chin Med. 2022 May 30;17:62. doi: 10.1186/s13020-022-00610-x (PMC9150355; doi:10.1186/s13020-022-00610-x)
Supplement: Supplementary file 7 — Additional file 7: Table S4. The chemical components identified with only formulas in SHL oral liquid preparation form. [file 13020_2022_610_MOESM7_ESM.docx]

**Table S4. The chemical components identified with only formulas in SHL oral liquid preparation form (n = 3)**

| **No.** | **Formula** | **t_R_ (min) (Mean ± SD)** | **Observed Mass (Mean ± SD)** | **Mass (MFG)** | **Precursor ion, m/z** |
| --- | --- | --- | --- | --- | --- |
| 1 | C_17_H_16_F_5_N_9_OP | 0.89 ± 0.01 | 488.1140 ± 0.0013 | 488.1108 | 487.1071, [M-H]¯ |
| 2 | C_15_H_14_F_6_NO_2_ | 0.93 ± 0.01 | 354.0922 ± 0.0011 | 354.0907 | 353.0854, [M-H]¯ |
| 3 | C_12_H_17_F_2_N_8_O_3_P_3_ | 0.93 ± 0.01 | 452.0591 ± 0.0017 | 452.0577 | 451.0531, [M-H]¯ |
| 4 | C_21_H_34_F_4_N_11_O_4_P_4_ | 1.08 ± 0.00 | 704.1672 ± 0.0015 | 704.1665 | 703.1609, [M-H]¯ |
| 5 | C_15_H_14_F_6_NO | 1.08 ± 0.00 | 338.0974 ± 0.0011 | 338.0964 | 337.0915, [M-H]¯ |
| 6 | C_29_H_52_F_2_N_2_O_4_P_6_ | 1.17 ± 0.00 | 716.2312 ± 0.0017 | 716.2303 | 715.2248, [M-H]¯ |
| 7 | C_23_H_36_F_4_N_3_P_4_ | 1.17 ± 0.00 | 554.1809 ± 0.0009 | 554.1785 | 553.1738, [M-H]¯ |
| 8 | C_28_H_57_F_3_NO_3_P_8_Si | 1.31 ± 0.02 | 788.1960 ± 0.0021 | 788.1949 | 787.1898, [M-H]¯ |
| 9 | C_26_H_30_F_2_N_6_O_10_ | 1.36 ± 0.02 | 624.2005 ± 0.0021 | 624.1997 | 623.1948, [M-H]¯ |
| 10 | C_11_H_15_F_6_N_3_O_3_P | 1.37 ± 0.02 | 382.0756 ± 0.0001 | 382.0727 | 383.0828, [M+H]⁺ |
| 11 | C_10_H_19_F_2_N_5_O_4_P | 1.60 ± 0.05 | 342.1137 ± 0.0008 | 342.1134 | 341.1068, [M-H]¯ |
| 12 | C_54_H_49_F_5_N_6_O_9_Si | 1.63 ± 0.03 | 1048.3244 ± 0.0002 | 1048.3221 | 525.1698, [M+2H]²⁺ |
| 13 | C_43_H_43_F_3_O_7_P_3_ | 1.68 ± 0.03 | 821.2159 ± 0.0009 | 821.2162 | 820.2101, [M-H]¯ |
| 14 | C_40_H_58_F_3_N_3_O_3_P_4_Si_4_ | 1.74 ± 0.00 | 921.2421 ± 0.0011 | 921.2433 | 920.2385, [M-H]¯ |
| 15 | C_9_H_11_NO_3_ | 1.85 ± 0.00 | 181.0751 ± 0.0005 | 181.0749 | 182.0827, [M+H]⁺ |
| 16 | C_32_H_42_N_2_O_17_P_2_ | 1.94 ± 0.03 | 788.1960 ± 0.0006 | 788.1956 | 787.1895, [M-H]¯ |
| 17 | C_34_H_46_N_5_O_24_ | 2.09 ± 0.02 | 908.2524 ± 0.0011 | 908.2512 | 907.2460, [M-H]¯ |
| 18 | C_28_H_39_F_12_N_5_P_3_ | 2.11 ± 0.02 | 766.2253 ± 0.0010 | 766.2253 | 765.2187, [M-H]¯ |
| 19 | C_46_H_44_F_4_N_19_OPSi_3_ | 2.38 ± 0.01 | 1069.2924 ± 0.0004 | 1069.2987 | 1068.2897, [M-H]¯ |
| 20 | C_26_H_30_F_2_N_6_O_10_ | 2.42 ± 0.01 | 624.2001 ± 0.0009 | 624.2003 | 623.1930, [M-H]¯ |
| 21 | C_29_H_63_F_11_N_11_O_4_P_8_ | 2.42 ± 0.01 | 1086.2786 ± 0.0007 | 1086.2812 | 1085.2725, [M-H]¯ |
| 22 | C_10_H_25_N_4_O_3_P_3_ | 2.47 ± 0.01 | 342.1134 ± 0.0006 | 342.1134 | 341.1065, [M-H]¯ |
| 23 | C_13_H_8_N_3_O_4_ | 2.78 ± 0.05 | 270.0508 ± 0.0008 | 270.0506 | 269.0439, [M-H]¯ |
| 24 | C_14_H_24_F_4_OP_2_ | 3.17 ± 0.00 | 346.1236 ± 0.0014 | 346.1233 | 345.1172, [M-H]¯ |
| 25 | C_25_H_30_N_12_O_4_P_2_ | 3.36 ± 0.00 | 624.2004 ± 0.0024 | 624.2013 | 623.1944, [M-H]¯ |
| 26 | C_21_H_16_N_6_O_2_P_2_ | 3.42 ± 0.00 | 446.0823 ± 0.0016 | 446.0819 | 222.0344, [M-2H]²¯ |
| 27 | C_20_H_22_N_2_O_6_P_2_ | 3.65 ± 0.05 | 448.0970 ± 0.0016 | 448.0980 | 447.0909, [M-H]¯ |
| 28 | C_28_H_38_N_2_O_10_P_2_ | 5.71 ± 0.05 | 624.2010 ± 0.0004 | 624.2001 | 623.1933, [M-H]¯ |
| 29 | C_27_H_39_N_4_O_7_P_3_ | 6.90 ± 0.00 | 624.2010 ± 0.0009 | 624.2003 | 311.0943, [M-2H]²¯ |
| 30 | C_41_H_36_F_16_N_2_S | 6.93 ± 0.05 | 892.2351 ± 0.0004 | 892.2347 | 891.2276, [M-H]¯ |
| 31 | C_22_H_28_F_4_N_6_O_5_P_2_ | 7.10 ± 0.02 | 594.1539 ± 0.0008 | 594.1542 | 593.1470, [M-H]¯ |
| 32 | C_39_H_36_N_5_O_16_S | 7.10 ± 0.02 | 862.1890 ± 0.0011 | 862.1893 | 861.1817, [M-H]¯ |
| 33 | C_19_H_24_F_3_N_4_P_2_S | 7.32 ± 0.02 | 459.1157 ± 0.0008 | 459.1167 | 458.1087, [M-H]¯ |
| 34 | C_24_H_38_FO_7_P_3_Si | 7.76 ± 0.00 | 578.1591 ± 0.0005 | 578.1586 | 577.1519, [M-H]¯ |
| 35 | C_27_H_44_FN_15_O_2_P_7_ | 7.76 ± 0.00 | 846.1954 ± 0.0006 | 846.1952 | 845.1884, [M-H]¯ |
| 36 | C_31_H_59_F_4_NO_4_P_9_ | 7.76 ± 0.00 | 864.2027 ± 0.0004 | 864.2026 | 863.1954, [M-H]¯ |
| 37 | C_22_H_40_F_5_N_10_OP_5_ | 7.82 ± 0.08 | 710.1997 ± 0.0005 | 710.2008 | 709.1922, [M-H]¯ |
| 38 | C_55_H_60_F_2_NO_5_P_8_ | 8.16 ± 0.02 | 1100.2319 ± 0.0018 | 1100.2317 | 551.1228, [M+2H]²⁺ |
| 39 | C_28_H_35_F_8_N_9_O_4_P | 8.30 ± 0.02 | 744.2414 ± 0.0017 | 744.2414 | 743.2349, [M-H]¯ |
| 40 | C_22_H_18_F_2_N_16_O_4_ | 8.37 ± 0.02 | 608.1693 ± 0.0012 | 608.1703 | 607.1627, [M-H]¯ |
| 41 | C_24_H_33_F_4_N_2_P_2_ | 8.71 ± 0.01 | 487.2057 ± 0.0002 | 487.2047 | 488.2127, [M+H]⁺ |
| 42 | C_22_H_13_F_3_N_3_O_4_P_2_Si | 9.36 ± 0.04 | 530.0105 ± 0.0001 | 530.0110 | 529.0030, [M-H]¯ |
| 43 | C_58_H_99_N_10_O_16_P_9_S_2_ | 9.69 ± 0.00 | 1534.4330 ± 0.0002 | 1534.4299 | 768.2233, [M+2H]²⁺ |
| 44 | C_28_H_26_F_4_N_8_O_4_PSi_2_ | 9.89 ± 0.00 | 701.1297 ± 0.0011 | 701.1255 | 700.1232, [M-H]¯ |
| 45 | C_29_H_54_FN_10_O_5_P_7_Si_3_ | 9.89 ± 0.00 | 942.1730 ± 0.0018 | 942.1723 | 941.1686, [M-H]¯ |
| 46 | C_37_H_48_F_7_N_7_O_2_P_5_ | 10.12 ± 0.01 | 910.2462 ± 0.0015 | 910.2462 | 909.2398, [M-H]¯ |
| 47 | C_27_H_44_NP_5_ | 10.70 ± 0.01 | 537.2150 ± 0.0008 | 537.2157 | 536.2088, [M-H]¯ |
| 48 | C_25_H_16_N_6_P | 11.08 ± 0.01 | 431.1161 ± 0.0006 | 431.1176 | 430.1117, [M-H]¯ |
| 49 | C_19_H_24_F_2_O_7_ | 11.11 ± 0.05 | 402.1494 ± 0.0010 | 402.1492 | 401.1429, [M-H]¯ |
| 50 | C_26_H_24_F_2_N_7_O_4_ | 11.30 ± 0.05 | 536.1853 ± 0.0016 | 536.1820 | 535.1786, [M-H]¯ |
| 51 | C_47_H_26_F_4_N_13_O_2_P_2_ | 11.49 ± 0.00 | 942.1738 ± 0.0001 | 942.1755 | 941.1683, [M-H]¯ |
| 52 | C_9_H_22_F_2_N_9_O_2_P_2_ | 11.53 ± 0.05 | 388.1349 ± 0.0001 | 388.1331 | 387.1279, [M-H]¯ |
| 53 | C_35_H_42_N_15_O_7_P_2_ | 11.58 ± 0.01 | 846.2899 ± 0.0015 | 846.2871 | 845.2824, [M-H]¯ |
| 54 | C_26_H_49_F_3_N_11_O_2_P_6_Si | 11.62 ± 0.05 | 818.2220 ± 0.0001 | 818.2197 | 817.2145, [M-H]¯ |
| 55 | C_18_H_26_F_4_N_4_P_2_ | 11.96 ± 0.00 | 436.1554 ± 0.0024 | 436.1545 | 435.1497, [M-H]¯ |
| 56 | C_20_H_46_FN_3_OP_5_ | 12.38 ± 0.04 | 518.2331 ± 0.0019 | 518.2314 | 517.2268, [M-H]¯ |
| 57 | C_53_H_67_F_5_N_3_O_8_P_4_ | 12.85 ± 0.08 | 1092.3827 ± 0.0036 | 1092.3820 | 1091.3775, [M-H]¯ |
| 58 | C_16_H_22_N_13_O_3_ | 13.86 ± 0.00 | 444.1960 ± 0.0006 | 444.1958 | 443.1900, [M-H]¯ |
| 59 | C_44_H_30_F_8_N_4_O_5_ | 14.56 ± 0.01 | 846.2099 ± 0.0006 | 846.2103 | 424.1126, [M+2H]²⁺ |
| 60 | C_43_H_73_F_19_N_3_OP_6_Si_2_ | 14.63 ± 0.00 | 1250.3426 ± 0.0005 | 1250.3428 | 626.178, [M+2H]²⁺ |
| 61 | C_23_H_40_F_14_N_8_OP_4_Si | 14.63 ± 0.00 | 862.1833 ± 0.0001 | 862.1837 | 863.1898, [M+H]⁺ |
| 62 | C_52_H_58_F_11_O_7_PSi | 15.07 ± 0.05 | 1062.3579 ± 0.0030 | 1062.3636 | 1061.3544, [M-H]¯ |
| 63 | C_28_H_26_F_4_N_7_OP | 15.07 ± 0.05 | 583.1863 ± 0.0020 | 583.1846 | 582.1800, [M-H]¯ |
| 64 | C_15_H_28_N_3_O_9_ | 16.40 ± 0.05 | 394.1815 ± 0.0011 | 394.1794 | 393.1749, [M-H]¯ |
| 65 | C_20_H_11_F_2_NO_4_ | 17.71 ± 0.08 | 367.0671 ± 0.0016 | 367.0682 | 366.0610, [M-H]¯ |
| 66 | C_27_H_45_P_5_ | 18.30 ± 0.03 | 524.2210 ± 0.0009 | 524.2213 | 523.2144, [M-H]¯ |
| 67 | C_25_H_34_N_2_O_6_P_2_ | 18.87 ± 0.02 | 520.1908 ± 0.0020 | 520.1898 | 519.1849, [M-H]¯ |
| 68 | C_26_H_41_N_3_OP_5_ | 18.87 ± 0.02 | 566.1957 ± 0.0025 | 566.1981 | 565.1900, [M-H]¯ |
| 69 | C_24_H_32_F_5_N_5_O_4_P | 19.23 ± 0.01 | 580.2107 ± 0.0023 | 580.2111 | 579.2052, [M-H]¯ |
| 70 | C_26_H_29_FN_5_O_5_S | 19.98 ± 0.07 | 542.1867 ± 0.0013 | 542.1853 | 541.1800, [M-H]¯ |
| 71 | C_16_H_18_N_6_O_3_ | 19.98 ± 0.07 | 342.1451 ± 0.0011 | 342.1445 | 341.1386, [M-H]¯ |
| 72 | C_19_H_20_F_9_N_14_OP | 21.64 ± 0.02 | 662.1566 ± 0.0010 | 662.1547 | 332.0837, [M+2H]²⁺ |
| 73 | C_27_H_32_FN_2_O_3_P | 22.56 ± 0.05 | 482.2118 ± 0.0021 | 482.2100 | 481.2060, [M-H]¯ |
| 74 | C_24_H_18_F_8_N_6_O | 23.19 ± 0.05 | 558.1459 ± 0.0001 | 558.1460 | 280.0782, [M+2H]²⁺ |
| 75 | C_53_H_69_F_4_NO_7_P_5_ | 23.24 ± 0.03 | 1062.3697 ± 0.0001 | 1062.3700 | 1063.3772, [M+H]⁺ |
| 76 | C_25_H_33_F_12_P | 23.40 ± 0.08 | 592.2134 ± 0.0003 | 592.2126 | 593.2204, [M+H]⁺ |
| 77 | C_22_H_28_F_2_O_2_ | 23.84 ± 0.04 | 362.2066 ± 0.0012 | 362.2076 | 361.2002, [M-H]¯ |
| 78 | C_26_H_32_FNO_7_P | 23.93 ± 0.04 | 520.1899 ± 0.0010 | 520.1904 | 519.1835, [M-H]¯ |
| 79 | C_21_H_39_F_5_N_4_P_2_ | 25.08 ± 0.04 | 504.2568 ± 0.0000 | 504.2569 | 527.2459, [M+Na]⁺ |
| 80 | C_21_H_18_F_4_N_4_O_6_ | 25.97 ± 0.01 | 498.1163 ± 0.0002 | 498.1162 | 499.1232, [M+H]⁺ |
| 81 | C_26_H_47_FN_20_P_5_ | 26.35 ± 0.01 | 813.2985 ± 0.0023 | 813.3003 | 812.2925, [M-H]¯ |
| 82 | C_18_H_21_F_3_O_3_ | 26.82 ± 0.08 | 342.1443 ± 0.0008 | 342.1442 | 341.1375, [M-H]¯ |
| 83 | C_17_H_21_F_3_O_3_ | 26.86 ± 0.02 | 330.1446 ± 0.0010 | 330.1442 | 329.1380, [M-H]¯ |
| 84 | C_25_H_32_N_5_O_2_P_4_ | 27.46 ± 0.01 | 558.1491 ± 0.0001 | 558.1508 | 559.1578, [M+H]⁺ |
| 85 | C_17_H_34_F_2_S_2_Si | 27.57 ± 0.03 | 368.1844 ± 0.0001 | 368.1831 | 369.1914, [M+H]⁺ |
| 86 | C_31_H_48_F_8_O_6_P_2_Si | 27.64 ± 0.00 | 758.2572 ± 0.0004 | 758.2575 | 757.2496, [M-H]¯ |
| 87 | C_18_H_31_F_3_O | 28.18 ± 0.02 | 320.2331 ± 0.0001 | 320.2329 | 319.2259, [M-H]¯ |
| 88 | C_26_H_36_F_8_N_14_P_2_ | 29.72 ± 0.01 | 758.2566 ± 0.0005 | 758.2553 | 757.2484, [M-H]¯ |
| 89 | C_30_H_65_F_4_N_10_OP_3_ | 29.82 ± 0.03 | 750.4493 ± 0.0000 | 750.4456 | 374.2173, [M-2H]²¯ |
| 90 | C_27_H_27_F_3_N_13_O_4_ | 29.97 ± 0.03 | 654.2257 ± 0.0002 | 654.2259 | 653.2176, [M-H]¯ |
| 91 | C_28_H_31_F_4_N_2_O_2_P | 30.69 ± 0.06 | 534.2057 ± 0.0000 | 534.2065 | 533.1986, [M-H]¯ |
| 92 | C_17_H_20_N_6_O_4_ | 30.69 ± 0.06 | 372.1546 ± 0.0000 | 372.1550 | 371.1473, [M-H]¯ |
| 93 | C_57_H_64_F_9_O_7_PSi | 32.32 ± 0.01 | 1090.4022 ± 0.0007 | 1090.4019 | 1091.4098, [M+H]⁺ |
| 94 | C_24_H_35_F_2_NO_8_P | 32.39 ± 0.03 | 534.2073 ± 0.0005 | 534.2067 | 533.1996, [M-H]¯ |
| 95 | C_29_H_33_F_4_N_2_O_4_P | 32.39 ± 0.03 | 580.2119 ± 0.0004 | 580.2111 | 579.2042, [M-H]¯ |
| 96 | C_19_H_23_F_3_O_4_ | 32.39 ± 0.03 | 372.1552 ± 0.0001 | 372.1553 | 371.1478, [M-H]¯ |
| 97 | C_30_H_43_F_6_N_5_OP_3_ | 32.39 ± 0.03 | 696.2584 ± 0.0006 | 696.2587 | 695.2508, [M-H]¯ |
| 98 | C_29_H_28_N_9_O_4_P | 32.39 ± 0.03 | 597.2021 ± 0.0004 | 597.2017 | 596.1941, [M-H]¯ |
| 99 | C_29_H_31_FN_6_O_4_P | 32.42 ± 0.02 | 577.2121 ± 0.0002 | 577.2119 | 576.2048, [M-H]¯ |
| 100 | C_48_H_58_F_6_O_6_P_2_ | 32.42 ± 0.06 | 906.3615 ± 0.0008 | 906.3616 | 905.3533, [M-H]¯ |
| 101 | C_22_H_25_F_9_N_16_O_9_Si_2_ | 33.45 ± 0.03 | 884.1394 ± 0.0005 | 884.1377 | 885.1458, [M+H]⁺ |
| 102 | C_18_H_42_F_2_N_2_P_4_ | 34.31 ± 0.02 | 448.2269 ± 0.0004 | 448.2275 | 447.2194, [M-H]¯ |
| 103 | C_12_H_27_N | 35.08 ± 0.01 | 185.2145 ± 0.0005 | 185.2144 | 186.222, [M+H]⁺ |
| 104 | C_19_H_23_F_3_O_4_ | 35.28 ± 0.00 | 372.1547 ± 0.0004 | 372.1549 | 371.1470, [M-H]¯ |
| 105 | C_12_H_26_N_3_O | 36.15 ± 0.01 | 228.2076 ± 0.0002 | 228.2088 | 227.2003, [M-H]¯ |
| 106 | C_30_H_37_FN_9_O_4_P_2_ | 36.62 ± 0.05 | 668.2411 ± 0.0009 | 668.2402 | 667.2327, [M-H]¯ |
| 107 | C_25_H_15_N_10_OP_3_ | 36.68 ± 0.02 | 564.0652 ± 0.0010 | 564.0635 | 565.073, [M+H]⁺ |
| 108 | C_14_H_28_N_3_O | 37.16 ± 0.03 | 254.2228 ± 0.0001 | 254.2228 | 253.2153, [M-H]¯ |
| 109 | C_31_H_45_F_2_N_4_O_7_P_2_ | 37.48 ± 0.00 | 685.2745 ± 0.0021 | 685.2727 | 686.2829, [M+H]⁺ |
| 110 | C_14_H_28_N_6_ | 37.75 ± 0.02 | 280.2379 ± 0.0005 | 280.2386 | 279.2301, [M-H]¯ |
| 111 | C_12_H_28_N_6_ | 38.25 ± 0.00 | 256.2387 ± 0.0008 | 256.2382 | 255.2307, [M-H]¯ |
| 112 | C_14_H_30_N_6_ | 38.80 ± 0.02 | 282.2542 ± 0.0004 | 282.2542 | 281.2463, [M-H]¯ |
| 113 | C_17_H_30_N_3_O_2_S | 39.37 ± 0.04 | 340.2056 ± 0.0008 | 340.2051 | 339.1976, [M-H]¯ |
| 114 | C_32_H_46_F_5_N_9_OP | 44.00 ± 0.01 | 698.3499 ± 0.0018 | 698.3463 | 699.3584, [M+H]⁺ |
| 115 | C_26_H_50_NO_7_P | 45.61 ± 0.00 | 519.3330 ± 0.0013 | 519.3324 | 520.3394, [M+H]⁺ |
| 116 | C_29_H_51_F_3_N_4_P_4_ | 50.49 ± 0.00 | 636.3006 ± 0.0013 | 636.2975 | 637.3088, [M+H]⁺ |
| 117 | C_27_H_42_F_2_NO_2_ | 51.35 ± 0.05 | 450.3162 ± 0.0024 | 450.3164 | 449.3079, [M-H]¯ |
| 118 | C_30_H_48_F_2_NO_2_ | 51.37 ± 0.04 | 492.3629 ± 0.0028 | 492.3619 | 491.3543, [M-H]¯ |
