# Supplementary material for: Metabolomics analyses of traditional Chinese medicine formula Shuang Huang Lian by UHPLC-QTOF-MS/MS
Source: Chin Med. 2022 May 30;17:62. doi: 10.1186/s13020-022-00610-x (PMC9150355; doi:10.1186/s13020-022-00610-x)
Supplement: Supplementary file 8 — Additional file 8: Table S5. The chemical components identified with both names and formulas in SHL tablet preparation form. [file 13020_2022_610_MOESM8_ESM.docx]

**Table S5. The chemical components identified with both names and formulas in SHL tablet preparation form (n = 3)**

| **No.** | **Formula** | **Name** | **t_R_ (min) (Mean ± SD)** | **Observed Mass (Mean ± SD)** | **Database Mass** | **Precursor ion, m/z** |
| --- | --- | --- | --- | --- | --- | --- |
| 1 | C_23_H_20_O_5_ | 5-O-Methylchamanetin | 1.15 ± 0.01 | 376.1314 ± 0.0003 | 376.1314 | 375.1238, [M-H]¯ |
| 2 | C_27_H_26_O_7_ | Euchrenone b3 | 1.31 ± 0.02 | 462.1683 ± 0.0013 | 462.1700 | 461.1621, [M-H]¯ |
| 3 | C_21_H_20_O_13_ | Myricetin 3'-glucoside | 1.62 ± 0.03 | 480.0876 ± 0.0018 | 480.0916 | 479.0817, [M-H]¯ |
| 4 | C_24_H_20_O_6_ | Calomelanol C | 1.71 ± 0.01 | 404.1281 ± 0.0006 | 404.1279 | 403.1204, [M-H]¯ |
| 5 | C_20_H_32_Br_2_O_2_ | Iriediol | 1.73 ± 0.02 | 462.0766 ± 0.0015 | 462.0774 | 461.0699, [M-H]¯ |
| 6 | C_9_H_11_NO_3_ | DL-o-Tyrosine | 1.86 ± 0.00 | 181.0738 ± 0.0003 | 181.0749 | 182.0813, [M+H]⁺ |
| 7 | C_10_H_13_N_5_O_4_ | Adenosine | 1.86 ± 0.00 | 267.0970 ± 0.0004 | 267.0968 | 268.1046, [M+H]⁺ |
| 8 | C_18_H_16_O_5_ | 5,6,2'-Trimethoxyflavone | 2.97 ± 0.08 | 312.0967 ± 0.0039 | 624.2025 | 311.0924, [M-H]¯ |
| 9 | C_28_H_16_O_5_ | Naphthofluorescein | 4.66 ± 0.05 | 432.1021 ± 0.0008 | 432.1031 | 431.0955, [M-H]¯ |
| 10 | C_33_H_24_O_10_ | Sciadopitysin | 4.8 ± 0.1 | 580.1375 ± 0.0011 | 580.1385 | 579.1310, [M-H]¯ |
| 11 | C_24_H_27_NO_6_S | Troglitazone quinone | 5.25 ± 0.04 | 457.1544 ± 0.0006 | 457.1547 | 456.1474, [M-H]¯ |
| 12 | C_21_H_26_N_4_O_8_ | Trp-Glu-Glu | 7.91 ± 0.00 | 462.1735 ± 0.0000 | 462.1751 | 480.2073, [M+NH_4_]⁺ |
| 13 | C_16_H_18_O_9_ | Chlorogenic Acid* | 8.11 ± 0.02 | 354.0952 ± 0.0001 | 354.0951 | 355.1025, [M+H]⁺ |
| 14 | C_9_H_6_O_3_ | Umbelliferone | 8.11 ± 0.02 | 162.0316 ± 0.0000 | 162.0316 | 163.0389, [M+H]⁺ |
| 15 | C_23_H_26_O_4_ | 2,2-Dimethyl-3-(4-methoxyphenyl)-4-propyl-2H-1-benzopyran-7-ol acetate | 8.41 ± 0.02 | 366.1849 ± 0.0005 | 366.1859 | 365.1771, [M-H]¯ |
| 16 | C_32_H_30_O_7_ | Kurzichalcolactone | 9.28 ± 0.00 | 526.1995 ± 0.0013 | 526.2005 | 525.1914, [M-H]¯ |
| 17 | C_8_H_6_O_3_ | Piperonal | 9.69 ± 0.01 | 150.0320 ± 0.0000 | 150.0317 | 151.0393, [M+H]⁺ |
| 18 | C_10_H_12_O_5_ | Danielone | 9.71 ± 0.03 | 212.0683 ± 0.0001 | 212.0682 | 213.0754, [M+H]⁺ |
| 19 | C_10_H_10_O_4_ | Methyl caffeate | 9.71 ± 0.03 | 194.0581 ± 0.0001 | 194.0582 | 195.0651, [M+H]⁺ |
| 20 | C_16_H_22_O_10_ | Geniposidic acid | 9.71 ± 0.03 | 374.1213 ± 0.0006 | 374.1213 | 397.1104, [M+Na]⁺ |
| 21 | C_16_H_18_O_8_ | p-Coumaroyl quinic acid | 10.48 ± 0.01 | 338.1005 ± 0.0001 | 338.1002 | 339.1076, [M+H]⁺ |
| 22 | C_17_H_27_N_5_O_6_ | Glu-His-Leu | 11.08 ± 0.02 | 397.1973 ± 0.0001 | 397.1961 | 420.1864, [M+Na]⁺ |
| 23 | C_26_H_24_O_7_ | Cycloartomunoxanthone | 11.11 ± 0.07 | 448.1528 ± 0.0004 | 448.1534 | 447.1458, [M-H]¯ |
| 24 | C_25_H_22_O_5_ | Ulexone B | 11.16 ± 0.01 | 402.1485 ± 0.0001 | 402.1492 | 401.1412, [M-H]¯ |
| 25 | C_16_H_28_N_6_O_8_ | Arg-Glu-Glu | 11.76 ± 0.02 | 432.1958 ± 0.0002 | 432.1960 | 431.1883, [M-H]¯ |
| 26 | C_16_H_22_O_9_ | Tarennoside | 11.79 ± 0.04 | 358.1265 ± 0.0002 | 358.1264 | 359.1338, [M+H]⁺ |
| 27 | C_10_H_12_O_4_ | Paeonilactone B | 11.79 ± 0.04 | 196.0738 ± 0.0001 | 196.0732 | 197.0811, [M+H]⁺ |
| 28 | C_15_H_26_N_6_O_6_ | Asp-Arg-Pro | 11.79 ± 0.05 | 386.1908 ± 0.0004 | 386.1916 | 385.1835, [M-H]¯ |
| 29 | C_28_H_48_N_2_O_21_ | MID58090:O-b-D-Gal-(1-3)-O-[O-b-D-Gal-(1-4)-2-(acetylamino)-2-deoxy-b-D-Glc-(1-6)]-2-(acetylamino) | 11.90 ± 0.04 | 748.2719 ± 0.0001 | 748.2729 | 747.2647, [M-H]¯ |
| 30 | C_33_H_32_O_7_ | Leucadenone C | 11.93 ± 0.00 | 540.2153 ± 0.0005 | 540.2162 | 539.2077, [M-H]¯ |
| 31 | C_25_H_24_O_7_ | Artonin J | 11.97 ± 0.06 | 436.1536 ± 0.0007 | 436.1545 | 435.1468, [M-H]¯ |
| 32 | C_40_H_50_O_20_ | Sempervirenoside B | 12.25 ± 0.01 | 850.2890 ± 0.0001 | 850.2895 | 868.3227, [M+NH_4_]⁺ |
| 33 | C_21_H_36_N_2_O_14_ | Galβ1-3GalNAcα-Thr | 12.34 ± 0.01 | 540.2211 ± 0.0004 | 540.2178 | 563.2093, [M+Na]⁺ |
| 34 | C_24_H_30_N_8_O_9_ | Tetrahydrofolyl-[Glu](2) | 12.75 ± 0.02 | 574.2106 ± 0.0006 | 574.2107 | 573.2037, [M-H]¯ |
| 35 | C_10_H_10_O_5_ | Vanilpyruvic acid | 14.55 ± 0.00 | 210.0532 ± 0.0001 | 210.0530 | 211.0605, [M+H]⁺ |
| 36 | C_9_H_8_O_3_ | cis-2-Hydroxycinnamate | 14.62 ± 0.00 | 164.0472 ± 0.0001 | 164.0469 | 165.0543, [M+H]⁺ |
| 37 | C_17_H_24_O_11_ | Gardenoside | 14.62 ± 0.00 | 404.1319 ± 0.0001 | 404.1319 | 427.1211, [M+Na]⁺ |
| 38 | C_50_H_34_O_11_ | Catechin Pentabenzoate | 14.62 ± 0.00 | 810.2114 ± 0.0007 | 810.2101 | 828.2453, [M+NH_4_]⁺ |
| 39 | C_35_H_44_O_23_ | Limocitrol 3-[alpha-L-arabinopyranosyl-(1-3)[galactosyl-(1-6)]-galactoside] | 14.62 ± 0.00 | 832.2261 ± 0.0002 | 832.2291 | 833.2349, [M+H]⁺ |
| 40 | C_20_H_22_O_6_ | Pedicellin | 15.03 ± 0.05 | 358.1403 ± 0.0021 | 358.1375 | 357.1315, [M-H]¯ |
| 41 | C_27_H_34_F_6_O_3_ | MID42020:26,26,26,27,27,27-hexafluoro-1α,25-dihydroxy-23,23,24,24-tetradehydrovitamin D3 / 26,26,26, | 15.07 ± 0.00 | 520.2433 ± 0.0007 | 520.2440 | 519.2355, [M-H]¯ |
| 42 | C_20_H_24_N_4_O_6_ | Pro-Trp-Asp | 15.09 ± 0.00 | 416.1679 ± 0.0001 | 416.1696 | 439.1573, [M+Na]⁺ |
| 43 | C_20_H_27_N_5_O_6_ | Thr-Gln-Trp | 15.09 ± 0.00 | 433.1946 ± 0.0001 | 433.1943 | 434.2019, [M+H]⁺ |
| 44 | C_16_H_31_N_5_O_6_ | Asp-Lys-Lys | 16.33 ± 0.00 | 389.2287 ± 0.0001 | 389.2274 | 412.2178, [M+Na]⁺ |
| 45 | C_15_H_21_N_5_O_8_ | Asp-Glu-His | 16.35 ± 0.03 | 399.1395 ± 0.0001 | 399.1390 | 417.1734, [M+NH_4_]⁺ |
| 46 | C_16_H_18_N_6_O_4_ | 2-Phenylaminoadenosine | 16.43 ± 0.02 | 358.1394 ± 0.0008 | 358.1387 | 357.1313, [M-H]¯ |
| 47 | C_27_H_30_O_16_ | Luteolin 7-galactoside-4'-glucoside | 17.69 ± 0.01 | 610.1534 ± 0.0003 | 610.1537 | 611.1609, [M+H]⁺ |
| 48 | C_21_H_18_O_12_ | Luteolin 3'-glucuronide | 18.08 ± 0.02 | 462.0804 ± 0.0001 | 462.0797 | 463.0870, [M+H]⁺ |
| 49 | C_21_H_20_O_11_ | Luteolin-7-O-glucoside* | 19.29 ± 0.03 | 448.1008 ± 0.0001 | 448.1006 | 449.1082, [M+H]⁺ |
| 50 | C_20_H_27_NO_11_ | Amygdalin | 20.71 ± 0.01 | 457.1584 ± 0.0001 | 457.1585 | 458.1658, [M+H]⁺ |
| 51 | C_21_H_26_O_12_ | Plumieride | 21.00 ± 0.02 | 470.1424 ± 0.0001 | 470.1423 | 471.1495, [M+H]⁺ |
| 52 | C_29_H_36_O_15_ | Forsythoside A* | 21.07 ± 0.02 | 624.2052 ± 0.0001 | 624.2054 | 642.2391, [M+NH_4_]⁺ |
| 53 | C_9_H_14_N_3_O_7_P | dCMP | 21.07 ± 0.02 | 307.0579 ± 0.0001 | 307.0569 | 325.0917, [M+NH_4_]⁺ |
| 54 | C_13_H_28_N_6_O_8_ | Zwittermicin A | 22.67 ± 0.00 | 396.1968 ± 0.0004 | 396.1979 | 395.1898, [M-H]¯ |
| 55 | C_28_H_31_ClN_2_O_2_ | Desmethylloperamide | 22.88 ± 0.00 | 462.2077 ± 0.0006 | 462.2064 | 461.1991, [M-H]¯ |
| 56 | C_20_H_20_O_5_ | Morachalcone A | 23.19 ± 0.02 | 340.1309 ± 0.0001 | 340.1313 | 341.1382, [M+H]⁺ |
| 57 | C_26_H_32_O_11_ | Brusatol | 23.19 ± 0.02 | 520.1944 ± 0.0000 | 520.1945 | 538.2282, [M+NH_4_]⁺ |
| 58 | C_28_H_37_FO_7_ | βmethasone dipropionate | 24.46 ± 0.04 | 504.2530 ± 0.0004 | 504.2530 | 503.2452, [M-H]¯ |
| 59 | C_27_H_30_O_14_ | Isofurcatain 7-O-glucoside | 25.05 ± 0.03 | 578.1637 ± 0.0000 | 578.1637 | 579.1711, [M+H]⁺ |
| 60 | C_25_H_24_O_12_ | Apigenin 7-(3'',4''-diacetylglucoside) | 25.94 ± 0.04 | 516.1264 ± 0.0004 | 516.1269 | 517.1341, [M+H]⁺ |
| 61 | C_21_H_20_O_10_ | Neovitexin | 29.87 ± 0.01 | 432.1057 ± 0.0000 | 432.1058 | 433.1132, [M+H]⁺ |
| 62 | C_19_H_29_N_5_O_6_ | Tyr-Asn-Lys | 30.28 ± 0.03 | 423.2122 ± 0.0001 | 423.2118 | 441.2460, [M+NH_4_]⁺ |
| 63 | C_27_H_34_O_11_ | Undulatone | 30.69 ± 0.03 | 534.1990 ± 0.0001 | 534.2067 | 533.1977, [M-H]¯ |
| 64 | C_21_H_18_O_11_ | Baicalin* | 31.99 ± 0.03 | 446.0846 ± 0.0001 | 446.0849 | 447.0918, [M+H]⁺ |
| 65 | C_27_H_34_O_11_ | Forsythin* | 32.31 ± 0.00 | 534.2097 ± 0.0001 | 534.2101 | 552.2437, [M+NH_4_]⁺ |
| 66 | C_21_H_22_O_5_ | Xanthogalenol | 32.35 ± 0.05 | 354.1469 ± 0.0003 | 354.1471 | 355.1544, [M+H]⁺ |
| 67 | C_28_H_36_O_13_ | (+)-Syringaresinol O-beta-D-glucoside | 32.42 ± 0.00 | 580.2115 ± 0.0014 | 580.2111 | 579.2032, [M-H]¯ |
| 68 | C_11_H_13_N_3_O_6_ | Cucumopine | 32.70 ± 0.03 | 283.0814 ± 0.0011 | 283.0819 | 282.0757, [M-H]¯ |
| 69 | C_22_H_20_O_12_ | Hispidulin 7-glucuronide | 33.09 ± 0.01 | 476.0955 ± 0.0001 | 476.0957 | 477.1028, [M+H]⁺ |
| 70 | C_21_H_18_O_10_ | Chrysin 7-glucuronide | 33.45 ± 0.01 | 430.0898 ± 0.0001 | 430.0902 | 431.0969, [M+H]⁺ |
| 71 | C_22_H_20_O_11_ | Wogonin 7-glucuronide | 33.68 ± 0.01 | 460.1006 ± 0.0001 | 460.1008 | 461.1078, [M+H]⁺ |
| 72 | C_18_H_28_N_10_O_4_ | His-His-Arg | 34.23 ± 0.01 | 448.2277 ± 0.0001 | 448.2275 | 447.2202, [M-H]¯ |
| 73 | C_21_H_18_O_11_ | Apigenin 7-glucuronide | 34.38 ± 0.00 | 446.0844 ± 0.0001 | 446.0845 | 447.0916, [M+H]⁺ |
| 74 | C_30_H_44_O_2_ | Demethylphylloquinone | 36.27 ± 0.01 | 436.3340 ± 0.0001 | 436.3340 | 437.3410, [M+H]⁺ |
| 75 | C_16_H_12_O_6_ | Kaempferide | 36.48 ± 0.02 | 300.0637 ± 0.0001 | 300.0629 | 301.0710, [M+H]⁺ |
| 76 | C_15_H_10_O_5_ | 2'-Hydroxydaidzein | 36.71 ± 0.04 | 270.0531 ± 0.0001 | 270.0530 | 271.0604, [M+H]⁺ |
| 77 | C_21_H_24_O_6_ | Kadsurin A | 37.82 ± 0.03 | 372.1572 ± 0.0001 | 372.1573 | 390.1912, [M+NH_4_]⁺ |
| 78 | C_21_H_26_O_3_ | 2-Hydroxymestranol | 38.58 ± 0.01 | 326.1883 ± 0.0006 | 326.1899 | 325.1811, [M-H]¯ |
| 79 | C_22_H_28_O_3_ | Canrenone | 39.41 ± 0.04 | 340.2042 ± 0.0006 | 340.2051 | 339.1978, [M-H]¯ |
| 80 | C_16_H_12_O_5_ | 5-O-Methylgenistein | 39.50 ± 0.02 | 284.0686 ± 0.0000 | 284.0674 | 285.0759, [M+H]⁺ |
| 81 | C_17_H_14_O_6_ | 5,3'-Dihydroxy-7,4'-dimethoxy-4-phenylcoumarin | 39.87 ± 0.02 | 314.0791 ± 0.0001 | 314.0792 | 315.0863, [M+H]⁺ |
| 82 | C_19_H_18_O_8_ | Skullcapflavone II | 40.18 ± 0.03 | 374.1001 ± 0.0004 | 374.0999 | 375.1072, [M+H]⁺ |
| 83 | C_15_H_22_O_2_ | Eremophilenolide | 45.44 ± 0.01 | 234.1623 ± 0.0000 | 234.1623 | 235.1696, [M+H]⁺ |
| 84 | C_46_H_73_O_10_P | PG (22:6(4Z,7Z,10Z,13Z,16Z,19Z)/18:3(6Z,9Z,12Z)) | 46.55 ± 0.01 | 816.4978 ± 0.0030 | 816.5010 | 815.4931, [M-H]¯ |
| 85 | C_24_H_50_NO_7_P | PE (19:0/0:0) | 46.60 ± 0.00 | 495.3325 ± 0.0001 | 495.3329 | 518.3220, [M+Na]⁺ |
| 86 | C_19_H_38_O_4_ | 1-Monopalmitin | 50.89 ± 0.01 | 330.2776 ± 0.0001 | 330.2769 | 331.2849, [M+H]⁺ |
| 87 | C_51_H_84_O_15_ | 1,2-Di-(9Z,12Z,15Z-octadecatrienoyl)-3-(Galactosyl-alpha-1-6-Galactosyl-beta-1)-glycerol | 51.02 ± 0.03 | 936.5819 ± 0.0004 | 936.5810 | 954.6159, [M+NH_4_]⁺ |
| 88 | C_45_H_74_O_10_ | 1,2 di-(9Z,12Z,15Z-octadecatrienoyl)-3-O-Beta-D-galactosyl-sn-glycerol | 51.25 ± 0.00 | 774.5288 ± 0.0005 | 774.5282 | 792.5621, [M+NH_4_]⁺ |
| 89 | C_37_H_60_O_5_ | DG (14:1(9Z)/20:5(5Z,8Z,11Z,14Z,17Z)/0:0) | 51.44 ± 0.00 | 584.4442 ± 0.0005 | 584.4443 | 585.4511, [M+H]⁺ |
| 90 | C_38_H_73_O_13_P | PI (16:0/13:0) | 51.47 ± 0.04 | 768.4797 ± 0.0005 | 768.4794 | 769.4865, [M+H]⁺ |
| 91 | C_43_H_70_O_10_ | MGDG (18:3(9Z,12Z,15Z)/16:3(7Z,10Z,13Z)) | 51.48 ± 0.03 | 746.4974 ± 0.0006 | 746.4969 | 769.4865, [M+Na]⁺ |

*Marker components.
