# Supplementary material for: Metabolomics analyses of traditional Chinese medicine formula Shuang Huang Lian by UHPLC-QTOF-MS/MS
Source: Chin Med. 2022 May 30;17:62. doi: 10.1186/s13020-022-00610-x (PMC9150355; doi:10.1186/s13020-022-00610-x)
Supplement: Supplementary file 9 — Additional file 9: Table S6. The chemical components identified with only formulas in SHL tablet preparation form. [file 13020_2022_610_MOESM9_ESM.docx]

**Table S6. The chemical components identified with only formulas in SHL tablet preparation form (n = 3)**

| **No.** | **Formula** | **t_R_ (min) (Mean ± SD)** | **Observed Mass (Mean ± SD)** | **Mass (MFG)** | **Precursor ion, m/z** |
| --- | --- | --- | --- | --- | --- |
| 1 | C_12_H_17_F_2_N_8_O_3_P_3_ | 1.03 ± 0.02 | 452.0589 ± 0.0013 | 452.0577 | 451.0528, [M-H]¯ |
| 2 | C_7_H_7_F_3_O_2_ | 1.03 ± 0.02 | 180.0401 ± 0.0003 | 180.0400 | 179.0331, [M-H]¯ |
| 3 | C_15_H_14_F_6_NO_2_ | 1.10 ± 0.01 | 354.0913 ± 0.0004 | 354.0907 | 353.0837, [M-H]¯ |
| 4 | C_20_H_24_F_2_N_9_O_4_P_2_ | 1.10 ± 0.01 | 554.1386 ± 0.0011 | 554.1387 | 553.1308, [M-H]¯ |
| 5 | C_15_H_14_F_6_NO | 1.15 ± 0.01 | 338.0962 ± 0.0008 | 338.0964 | 337.0884, [M-H]¯ |
| 6 | C_25_H_20_F_4_N_16_O_6_ | 1.18 ± 0.03 | 716.1698 ± 0.0021 | 716.1706 | 715.1633, [M-H]¯ |
| 7 | C_24_H_36_N_2_O_2_P_4_ | 1.20 ± 0.01 | 508.1719 ± 0.0018 | 508.1731 | 507.1653, [M-H]¯ |
| 8 | C_21_H_33_FN_9_P_4_ | 1.20 ± 0.01 | 554.1799 ± 0.0016 | 554.1785 | 553.1699, [M-H]¯ |
| 9 | C_28_H_57_F_3_NO_3_P_8_Si | 1.25 ± 0.01 | 788.1938 ± 0.0020 | 788.1949 | 787.1846, [M-H]¯ |
| 10 | C_19_H_16_FN_4_O_6_P | 1.29 ± 0.01 | 446.0807 ± 0.0013 | 446.0794 | 445.0720, [M-H]¯ |
| 11 | C_26_H_30_F_2_N_6_O_10_ | 1.29 ± 0.01 | 624.1991 ± 0.0019 | 624.1997 | 623.1903, [M-H]¯ |
| 12 | C_5_H_4_F_6_N | 1.44 ± 0.01 | 192.0250 ± 0.0009 | 192.0255 | 191.0169, [M-H]¯ |
| 13 | C_15_H_29_N_2_O_3_P_5_ | 1.46 ± 0.02 | 440.0880 ± 0.0018 | 440.0888 | 439.0819, [M-H]¯ |
| 14 | C_9_H_19_F_8_N_4_OP_3_ | 1.94 ± 0.01 | 444.0654 ± 0.0013 | 444.0693 | 443.0590, [M-H]¯ |
| 15 | C_34_H_46_N_5_O_24_ | 2.05 ± 0.00 | 908.2517 ± 0.0019 | 908.2512 | 907.2426, [M-H]¯ |
| 16 | C_28_H_39_F_12_N_5_P_3_ | 2.10 ± 0.00 | 766.2239 ± 0.0012 | 766.2253 | 765.2154, [M-H]¯ |
| 17 | C_31_H_52_F_8_N_19_O_3_P_4_Si_2_ | 2.42 ± 0.03 | 1070.2847 ± 0.0018 | 1069.2987 | 1069.2755, [M-H]¯ |
| 18 | C_26_H_30_F_2_N_6_O_10_ | 2.47 ± 0.03 | 624.1994 ± 0.0006 | 624.2003 | 623.1929, [M-H]¯ |
| 19 | C_6_H_10_N_3_O_2_ | 2.61 ± 0.04 | 156.0774 ± 0.0002 | 156.0777 | 155.0701, [M-H]¯ |
| 20 | C_14_H_24_F_4_OP_2_ | 3.1 ± 0.1 | 346.1231 ± 0.0009 | 346.1233 | 345.1164, [M-H]¯ |
| 21 | C_25_H_30_N_12_O_4_P_2_ | 3.22 ± 0.00 | 624.1983 ± 0.0000 | 624.1994 | 623.1911, [M-H]¯ |
| 22 | C_20_H_22_N_2_O_6_P_2_ | 3.68 ± 0.01 | 448.0966 ± 0.0016 | 448.0980 | 447.0903, [M-H]¯ |
| 23 | C_24_H_41_FN_2_OP_4_ | 5.31 ± 0.04 | 516.2156 ± 0.0004 | 516.2159 | 515.2087, [M-H]¯ |
| 24 | C_28_H_38_N_2_O_10_P_2_ | 5.88 ± 0.01 | 624.1994 ± 0.0013 | 624.2001 | 311.0921, [M-2H]²¯ |
| 25 | C_27_H_39_N_4_O_7_P3 | 6.97 ± 0.03 | 624.1998 ± 0.0002 | 624.2003 | 623.1922, [M-H]¯ |
| 26 | C_41_H_36_F_16_N_2_S | 7.04 ± 0.03 | 892.2332 ± 0.0011 | 892.2347 | 891.2262, [M-H]¯ |
| 27 | C_56_H_60_F_19_O_2_P_3_ | 7.07 ± 0.08 | 1218.3491 ± 0.0001 | 1218.3505 | 1217.3431, [M-H]¯ |
| 28 | C_43_H_42_F_2_N_2_O_7_P_2_S_2_ | 7.15 ± 0.08 | 862.1896 ± 0.0005 | 862.1893 | 861.1811, [M-H]¯ |
| 29 | C_20_H_29_F_10_N_2_O_3_PSi | 7.23 ± 0.07 | 594.1534 ± 0.0005 | 594.1542 | 296.0695, [M-2H]²¯ |
| 30 | C_20_H_29_O_6_P_2_S | 7.41 ± 0.07 | 459.1154 ± 0.0008 | 459.1167 | 458.1087, [M-H]¯ |
| 31 | C_29_H_45_F_8_N_6_O_5_P_5_ | 7.74 ± 0.02 | 864.2016 ± 0.0004 | 864.2026 | 863.1942, [M-H]¯ |
| 32 | C_25_H_27_F_3_N_6_O_3_P_2_ | 7.77 ± 0.06 | 578.1578 ± 0.0007 | 578.1586 | 577.1508, [M-H]¯ |
| 33 | C_34_H_32_F_15_N_3_O_3_P | 7.77 ± 0.06 | 846.1941 ± 0.0005 | 846.1952 | 845.1873, [M-H]¯ |
| 34 | C_36_H_31_N_11_P_3_ | 7.82 ± 0.06 | 710.1990 ± 0.0010 | 710.2008 | 709.1916, [M-H]¯ |
| 35 | C_55_H_60_F_2_NO_5_P_8_ | 8.11 ± 0.02 | 1100.2321 ± 0.0002 | 1100.2317 | 551.1231, [M+2H]²⁺ |
| 36 | C_22_H_18_F_2_N_16_O_4_ | 8.28 ± 0.06 | 608.1680 ± 0.0016 | 608.1703 | 607.1607, [M-H]¯ |
| 37 | C_23_H_33_F_7_N_16_OP_2_ | 8.41 ± 0.02 | 744.2400 ± 0.0013 | 744.2414 | 743.2314, [M-H]¯ |
| 38 | C_22_H_25_F_6_N_6_ | 8.72 ± 0.00 | 487.2051 ± 0.0008 | 487.2047 | 488.2117, [M+H]⁺ |
| 39 | C_25_H_27_F_3_OP_2_ | 8.81 ± 0.05 | 462.1493 ± 0.0007 | 462.1493 | 461.1402, [M-H]¯ |
| 40 | C_29_H_34_F_6_N_10_O_2_P_2_ | 8.93 ± 0.02 | 730.2260 ± 0.0001 | 730.2249 | 364.1045, [M-2H]²¯ |
| 41 | C_56_H_48_F_12_N_5_O_3_P_2_ | 9.49 ± 0.04 | 1128.3062 ± 0.0028 | 1128.3081 | 1127.3006, [M-H]¯ |
| 42 | C_57_H_76_F_29_N_5_P_3_SSi | 9.71 ± 0.03 | 1534.4336 ± 0.0002 | 1534.4299 | 768.2243, [M+2H]²⁺ |
| 43 | C_24_H_39_FN_15_O_4_P_4_Si | 9.71 ± 0.03 | 772.2019 ± 0.0001 | 772.2014 | 773.2085, [M+H]⁺ |
| 44 | C_30_H_8_N_7_O_2_P_2_ | 9.85 ± 0.00 | 560.0197 ± 0.0019 | 560.0205 | 559.0142, [M-H]¯ |
| 45 | C_21_H_16_F_2_N_3_O_7_ | 9.92 ± 0.01 | 460.0967 ± 0.0016 | 460.0961 | 459.0900, [M-H]¯ |
| 46 | C_29_H_54_FN_10_O_5_P_7_Si_3_ | 9.92 ± 0.01 | 942.1696 ± 0.0006 | 942.1723 | 941.1643, [M-H]¯ |
| 47 | C_21_H_22_F_2_N_3_O_6_ | 10.27 ± 0.01 | 450.1484 ± 0.0008 | 450.1490 | 449.1416, [M-H]¯ |
| 48 | C_21_H_26_F_9_N_4_P | 10.50 ± 0.02 | 536.1756 ± 0.0001 | 536.1822 | 535.1680, [M-H]¯ |
| 49 | C_39_H_36_F_4_N_4_O_2_P_2_ | 11.26 ± 0.05 | 730.2253 ± 0.0002 | 730.2255 | 364.1048, [M-2H]²¯ |
| 50 | C_25_H_34_F_5_NP_3_ | 11.26 ± 0.05 | 536.1842 ± 0.0003 | 536.1820 | 535.1760, [M-H]¯ |
| 51 | C_22_H_29_F_3_N_3_P_2_ | 11.29 ± 0.00 | 454.1794 ± 0.0002 | 454.1792 | 453.1718, [M-H]¯ |
| 52 | C_14_H_33_F_3_N_2_O_3_P_4_ | 11.32 ± 0.02 | 458.1396 ± 0.0000 | 458.1396 | 459.1468, [M+H]⁺ |
| 53 | C_19_H_28_ClFNS_2_ | 11.48 ± 0.01 | 388.1341 ± 0.0005 | 388.1331 | 387.1262, [M-H]¯ |
| 54 | C_35_H_42_N_15_O_7_P_2_ | 11.76 ± 0.02 | 846.2868 ± 0.0004 | 846.2871 | 845.2794, [M-H]¯ |
| 55 | C_48_H_62_F_5_O_7_P_5_ | 11.81 ± 0.01 | 1000.3068 ± 0.0062 | 1000.3113 | 999.3041, [M-H]¯ |
| 56 | C_17_H_20_F_2_O_6_ | 11.84 ± 0.05 | 358.1232 ± 0.0003 | 358.1237 | 357.1162, [M-H]¯ |
| 57 | C_26_H_57_N_8_P_9_ | 12.64 ± 0.03 | 760.2350 ± 0.0007 | 760.2351 | 759.2280, [M-H]¯ |
| 58 | C_27_H_57_F_3_N_6_O_3_P_8_Si | 14.59 ± 0.05 | 846.2123 ± 0.0002 | 846.2103 | 847.2178, [M+H]⁺ |
| 59 | C_41_H_82_F_4_N_6_O_15_P_8_Si | 14.62 ± 0.00 | 1250.3441 ± 0.0001 | 1250.3428 | 626.1795, [M+2H]²⁺ |
| 60 | C_24_H_41_N_17_O_7_P_5_Si | 14.62 ± 0.00 | 862.1840 ± 0.0006 | 862.1837 | 863.1905, [M+H]⁺ |
| 61 | C_25_H_34_N_2_O_6_P_2_ | 15.07 ± 0.00 | 520.1903 ± 0.0013 | 520.1916 | 519.1839, [M-H]¯ |
| 62 | C_52_H_58_F_11_O_7_PSi | 15.07 ± 0.00 | 1062.3572 ± 0.0016 | 1062.3636 | 1061.3519, [M-H]¯ |
| 63 | C_24_H_24_F_5_N_8_O_4_ | 15.07 ± 0.00 | 583.1854 ± 0.0015 | 583.1846 | 582.1790, [M-H]¯ |
| 64 | C_17_H_25_F_2_N_5_O_3_P | 15.18 ± 0.02 | 416.1654 ± 0.0013 | 416.1642 | 415.1570, [M-H]¯ |
| 65 | C_18_H_22_N_3_O_2_P_2_ | 15.64 ± 0.05 | 374.1187 ± 0.0004 | 374.1191 | 373.1117, [M-H]¯ |
| 66 | C_17_H_28_F_4_O_4_P_2_ | 15.64 ± 0.05 | 434.1422 ± 0.0005 | 434.1412 | 433.1352, [M-H]¯ |
| 67 | C_36_H_45_NO_15_P | 15.64 ± 0.05 | 762.2530 ± 0.0001 | 762.2530 | 761.2453, [M-H]¯ |
| 68 | C_30_H_35_F_15_N_6_O | 15.64 ± 0.05 | 780.2638 ± 0.0004 | 780.2641 | 779.2565, [M-H]¯ |
| 69 | C_15_H_23_F_3_O_8_ | 15.68 ± 0.00 | 388.1343 ± 0.0004 | 388.1345 | 387.1267, [M-H]¯ |
| 70 | C_16_H_26_F_6_NO_3_ | 16.39 ± 0.04 | 394.1813 ± 0.0011 | 394.1794 | 393.1747, [M-H]¯ |
| 71 | C_27_H_45_P_5_ | 18.27 ± 0.04 | 524.2210 ± 0.0003 | 524.2213 | 523.2141, [M-H]¯ |
| 72 | C_27_H_29_F_4_N_2_O_2_P | 18.39 ± 0.00 | 520.1906 ± 0.0005 | 520.1911 | 519.1829, [M-H]¯ |
| 73 | C_25_H_34_N_2_O_6_P_2_ | 18.86 ± 0.00 | 520.1910 ± 0.0004 | 520.1898 | 519.1838, [M-H]¯ |
| 74 | C_20_H_22_N_16_O_5_ | 18.86 ± 0.00 | 566.1961 ± 0.0005 | 566.1981 | 565.1887, [M-H]¯ |
| 75 | C_24_H_32_F_5_N_5_O_4_P | 19.21 ± 0.01 | 580.2112 ± 0.0006 | 580.2111 | 579.2041, [M-H]¯ |
| 76 | C_16_H_18_N_6_O_3_ | 20.00 ± 0.01 | 342.1443 ± 0.0001 | 342.1445 | 341.1369, [M-H]¯ |
| 77 | C_23_H_33_FN_4_O_6_P_2_ | 20.16 ± 0.04 | 542.1854 ± 0.0007 | 542.1853 | 541.1786, [M-H]¯ |
| 78 | C_22_H_19_F_11_N_11_O | 21.61 ± 0.00 | 662.1548 ± 0.0003 | 662.1547 | 663.1671, [M+H]⁺ |
| 79 | C_53_H_69_F_4_NO_7_P_5_ | 23.19 ± 0.02 | 1062.3710 ± 0.0003 | 1062.3700 | 1063.3778, [M+H]⁺ |
| 80 | C_17_H_39_F_3_N_4_OP_6_ | 23.24 ± 0.02 | 558.1454 ± 0.0001 | 558.1460 | 559.1577, [M+H]⁺ |
| 81 | C_25_H_33_F_12_P | 23.38 ± 0.01 | 592.2128 ± 0.0002 | 592.2126 | 593.2201, [M+H]⁺ |
| 82 | C_26_H_32_FNO_7_P | 23.89 ± 0.01 | 520.1907 ± 0.0002 | 520.1904 | 519.1833, [M-H]¯ |
| 83 | C_21_H_39_F_5_N_4_P_2_ | 25.04 ± 0.05 | 504.2569 ± 0.0001 | 504.2569 | 505.2644, [M+H]⁺ |
| 84 | C_22_H_28_FNO_5_P_3_ | 25.92 ± 0.07 | 498.1164 ± 0.0000 | 498.1162 | 499.1237, [M+H]⁺ |
| 85 | C_35_H_48_F_8_N_6_OP_3_ | 26.36 ± 0.00 | 813.2990 ± 0.0018 | 813.3003 | 812.2928, [M-H]¯ |
| 86 | C_18_H_21_F_3_O_3_ | 26.79 ± 0.02 | 342.1444 ± 0.0000 | 342.1442 | 341.1372, [M-H]¯ |
| 87 | C_17_H_21_F_3_O_3_ | 26.79 ± 0.02 | 330.1444 ± 0.0001 | 330.1442 | 329.1371, [M-H]¯ |
| 88 | C_20_H_36_N_6_O_2_ | 27.11 ± 0.00 | 392.2908 ± 0.0007 | 392.2913 | 391.2830, [M-H]¯ |
| 89 | C_25_H_32_N_5_O_2_P_4_ | 27.45 ± 0.06 | 558.1494 ± 0.0015 | 558.1508 | 559.1593, [M+H]⁺ |
| 90 | C_13_H_33_N_4_O_2_PSSi | 27.57 ± 0.05 | 368.1839 ± 0.0003 | 368.1831 | 369.1902, [M+H]⁺ |
| 91 | C_31_H_48_F_8_O_6_P_2_Si | 27.60 ± 0.04 | 758.2577 ± 0.0010 | 758.2575 | 757.2509, [M-H]¯ |
| 92 | C_22_H_39_F_3_O_3_ | 28.37 ± 0.01 | 408.2846 ± 0.0004 | 408.2844 | 407.2771, [M-H]¯ |
| 93 | C_26_H_36_F_8_N_14_P_2_ | 29.71 ± 0.00 | 758.2590 ± 0.0011 | 758.2553 | 757.2522, [M-H]¯ |
| 94 | C_42_H_63_F_8_P | 29.90 ± 0.00 | 750.4512 ± 0.0030 | 750.4456 | 374.2197, [M-2H]²¯ |
| 95 | C_27_H_27_F_3_N_13_O_4_ | 30.00 ± 0.01 | 654.2283 ± 0.0012 | 654.2259 | 653.2209, [M-H]¯ |
| 96 | C_17_H_20_N_6_O_4_ | 30.64 ± 0.04 | 372.1548 ± 0.0001 | 372.1550 | 371.1475, [M-H]¯ |
| 97 | C_24_H_34_F_4_N_3_O_2_P_2_ | 30.69 ± 0.03 | 534.2055 ± 0.0004 | 534.2065 | 533.199, [M-H]¯ |
| 98 | C_25_H_46_N_14_OP_6_ | 31.26 ± 0.04 | 744.2422 ± 0.0013 | 744.2421 | 743.2331, [M-H]¯ |
| 99 | C_57_H_64_F_9_O_7_PSi | 32.35 ± 0.05 | 1090.4012 ± 0.0001 | 1090.4019 | 1091.4084, [M+H]⁺ |
| 100 | C_29_H_28_N_9_O_4_P | 32.37 ± 0.06 | 597.2015 ± 0.0016 | 597.2017 | 596.1952, [M-H]¯ |
| 101 | C_30_H_43_F_6_N_5_OP_3_ | 32.42 ± 0.00 | 696.2582 ± 0.0019 | 696.2587 | 695.2495, [M-H]¯ |
| 102 | C_48_H_58_F_6_O_6_P_2_ | 32.42 ± 0.00 | 906.3606 ± 0.0026 | 906.3616 | 905.3516, [M-H]¯ |
| 103 | C_22_H_25_F_9_N_16_O_9_Si_2_ | 33.47 ± 0.01 | 884.1376 ± 0.0003 | 884.1377 | 885.1446, [M+H]⁺ |
| 104 | C_42_H_92_F_2_N_19_O_11_S | 34.09 ± 0.01 | 1108.6929 ± 0.0008 | 1108.6909 | 555.3537, [M+2H]²⁺ |
| 105 | C_19_H_23_F_3_O_4_ | 35.23 ± 0.00 | 372.1549 ± 0.0005 | 372.1549 | 371.1479, [M-H]¯ |
| 106 | C_12_H_27_N | 35.24 ± 0.04 | 185.2144 ± 0.0000 | 185.2144 | 186.2217, [M+H]⁺ |
| 107 | C_12_H_26_N_3_O | 36.16± 0.01 | 228.2077 ± 0.0006 | 228.2088 | 227.1999, [M-H]¯ |
| 108 | C_30_H_37_FN_9_O_4_P_2_ | 36.62 ± 0.03 | 668.2419 ± 0.0017 | 668.2402 | 667.236, [M-H]¯ |
| 109 | C_25_H_15_N_10_OP_3_ | 36.71 ± 0.04 | 564.0637 ± 0.0002 | 564.0635 | 565.0710, [M+H]⁺ |
| 110 | C_14_H_28_N_3_O | 37.20 ± 0.01 | 254.2230 ± 0.0003 | 254.2228 | 253.2155, [M-H]¯ |
| 111 | C_54_H_81_F_4_N_8_O_4_P_3_ | 37.38 ± 0.05 | 1074.5528 ± 0.0008 | 1074.5531 | 1073.5456, [M-H]¯ |
| 112 | C_31_H_45_F_2_N_4_O_7_P_2_ | 37.49 ± 0.00 | 685.2734 ± 0.0001 | 685.2727 | 686.2804, [M+H]⁺ |
| 113 | C_16_H_31_F_3_ | 37.80 ± 0.01 | 280.2383 ± 0.0007 | 280.2386 | 279.2305, [M-H]¯ |
| 114 | C_12_H_28_N_6_ | 38.25 ± 0.02 | 256.2385 ± 0.0014 | 256.2382 | 255.2301, [M-H]¯ |
| 115 | C_16_H_32_N_3_O | 38.84 ± 0.00 | 282.2538 ± 0.0004 | 282.2542 | 281.2467, [M-H]¯ |
| 116 | C_15_H_32_N_3_O | 38.84 ± 0.00 | 270.2535 ± 0.0004 | 270.2540 | 269.2465, [M-H]¯ |
| 117 | C_16_H_34_N_3_O | 40.01 ± 0.02 | 284.2698 ± 0.0004 | 284.2697 | 283.2627, [M-H]¯ |
| 118 | C_22_H_27_FN_5_O_4_ | 42.37 ± 0.01 | 444.2047 ± 0.0000 | 444.2049 | 445.2122, [M+H]⁺ |
| 119 | C_32_H_46_F_5_N_9_OP | 43.98 ± 0.01 | 698.3485 ± 0.0001 | 698.3463 | 699.3558, [M+H]⁺ |
| 120 | C_28_H_46_F_4_OP_2_ | 45.55 ± 0.03 | 536.2959 ± 0.0001 | 536.2945 | 537.3031, [M+H]⁺ |
| 121 | C_26_H_50_NO_7_P | 45.59 ± 0.03 | 519.3322 ± 0.0004 | 519.3324 | 520.3399, [M+H]⁺ |
| 122 | C_29_H_51_F_3_N_4_P_4_ | 50.47 ± 0.01 | 636.2988 ± 0.0004 | 636.2975 | 659.2871, [M+Na]⁺ |
| 123 | C_30_H_48_F_2_NO_2_ | 51.35 ± 0.00 | 492.3623 ± 0.0022 | 492.3619 | 491.3542, [M-H]¯ |
| 124 | C_27_H_42_F_2_NO_2_ | 51.37 ± 0.03 | 450.3158 ± 0.0018 | 450.3164 | 449.3105, [M-H]¯ |
