# Supplementary material for: Metabolomics analyses of traditional Chinese medicine formula Shuang Huang Lian by UHPLC-QTOF-MS/MS
Source: Chin Med. 2022 May 30;17:62. doi: 10.1186/s13020-022-00610-x (PMC9150355; doi:10.1186/s13020-022-00610-x)
Supplement: Supplementary file 10 — Additional file 10: Table S7. The chemical components found in each SHL preparation forms. [file 13020_2022_610_MOESM10_ESM.docx]

**Table S7. The chemical components found in each SHL preparation forms**

| **SHL** | **Components** | | |  | **Common components** | | | |
| --- | --- | --- | --- | --- | --- | --- | --- | --- |
| **Forms** | **ESI mode** | **Identified** | **Unidentified** | **Total** | **ESI mode** | **Identified** | **Unidentified** | **Total** |
| Granules | $+$ | 56 | 16 | 178 | $+$ | 40 | 13 | 95 |
|  | $-$ | 28 | 78 |  |  |  |  |  |
| Oral liquid | $+$ | 63 | 25 | 216 |  |  |  |  |
|  | $-$ | 35 | 93 |  | $-$ | 7 | 35 |  |
| Tablet | $+$ | 59 | 27 | 215 |  |  |  |  |
|  | $-$ | 32 | 97 |  |  |  |  |  |
