# Supplementary material for: Metabolomics analyses of traditional Chinese medicine formula Shuang Huang Lian by UHPLC-QTOF-MS/MS
Source: Chin Med. 2022 May 30;17:62. doi: 10.1186/s13020-022-00610-x (PMC9150355; doi:10.1186/s13020-022-00610-x)
Supplement: Supplementary file 11 — Additional file 11: Table S8. The common chemical components unidentified (or identified with formulas only) in all three SHL preparation forms. [file 13020_2022_610_MOESM11_ESM.docx]

**Table S8. The common chemical components unidentified (or identified with formulas only) in all three SHL preparation forms (n = 3)**

| **No.** | **Formula** | **t_R_ (min)** | **Observed Mass** | **Mass (MFG)** | **Precursor ion, m/z** |
| --- | --- | --- | --- | --- | --- |
| 1 | C_15_H_14_F_6_NO_2_ | 0.93 | 354.0926 | 354.0907 | 353.0855, [M-H]¯ |
| 2 | C_12_H_17_F_2_N_8_O_3_P_3_ | 0.93 | 452.0591 | 452.0577 | 451.0519, [M-H]¯ |
| 3 | C_15_H_14_F_6_NO | 1.05 | 338.0977 | 338.0964 | 337.0904, [M-H]¯ |
| 4 | C_28_H_57_F_3_NO_3_P_8_Si | 1.30 | 788.1951 | 788.1949 | 787.1889, [M-H]¯ |
| 5 | C_26_H_30_F_2_N_6_O_10_ | 1.34 | 624.2003 | 624.1997 | 623.1939, [M-H]¯ |
| 6 | C_28_H_39_F_12_N_5_P_3_ | 2.12 | 766.2260 | 766.2253 | 765.2191, [M-H]¯ |
| 7 | C_34_H_46_N_5_O_24_ | 2.15 | 908.2538 | 908.2512 | 907.2485, [M-H]¯ |
| 8 | C_26_H_30_F_2_N_6_O_10_ | 2.44 | 624.2015 | 624.2003 | 623.1955, [M-H]¯ |
| 9 | C_14_H_24_F_4_OP_2_ | 3.18 | 346.1237 | 346.1233 | 345.1167, [M-H]¯ |
| 10 | C_25_H_30_N_12_O_4_P_2_ | 3.25 | 624.2007 | 624.2013 | 623.1929, [M-H]¯ |
| 11 | C_20_H_22_N_2_O_6_P_2_ | 3.71 | 448.0965 | 448.0980 | 447.0897, [M-H]¯ |
| 12 | C_28_H_38_N_2_O_10_P_2_ | 5.70 | 624.2021 | 624.2001 | 623.1931, [M-H]¯ |
| 13 | C_41_H_36_F_16_N_2_S | 6.91 | 892.2359 | 892.2347 | 891.2288, [M-H]¯ |
| 14 | C_27_H_39_N_4_O_7_P_3_ | 7.00 | 624.2012 | 624.2003 | 623.1937, [M-H]¯ |
| 15 | C_55_H_60_F_2_NO_5_P_8_ | 8.15 | 1100.2321 | 1100.2317 | 551.1232, [M+2H]²⁺ |
| 16 | C_22_H_18_F_2_N_16_O_4_ | 8.34 | 608.1702 | 608.1703 | 607.1630, [M-H]¯ |
| 17 | C_29_H_54_FN_10_O_5_P_7_Si_3_ | 9.89 | 942.1687 | 942.1723 | 941.1661, [M-H]¯ |
| 18 | C_35_H_42_N_15_O_7_P_2_ | 11.60 | 846.2865 | 846.2871 | 845.2816, [M-H]¯ |
| 19 | C_52_H_58_F_11_O_7_PSi | 15.09 | 1062.3537 | 1062.3636 | 1061.3507, [M-H]¯ |
| 20 | C_27_H_45_P_5_ | 18.24 | 524.2213 | 524.2213 | 523.2147, [M-H]¯ |
| 21 | C_24_H_32_F_5_N_5_O_4_P | 19.23 | 580.2113 | 580.2111 | 579.2042, [M-H]¯ |
| 22 | C_16_H_18_N_6_O_3_ | 20.00 | 342.1443 | 342.1445 | 341.1374, [M-H]¯ |
| 23 | C_53_H_69_F_4_NO_7_P_5_ | 23.24 | 1062.3703 | 1062.3700 | 1063.3777, [M+H]⁺ |
| 24 | C_25_H_33_F_12_P | 23.44 | 592.2129 | 592.2126 | 593.2201, [M+H]⁺ |
| 25 | C_26_H_32_FNO_7_P | 23.91 | 520.1904 | 520.1904 | 519.1828, [M-H]¯ |
| 26 | C_21_H_39_F_5_N_4_P_2_ | 24.99 | 504.2572 | 504.2569 | 527.2462, [M+Na]⁺ |
| 27 | C_18_H_21_F_3_O_3_ | 26.83 | 342.1440 | 342.1442 | 341.1370, [M-H]¯ |
| 28 | C_17_H_21_F_3_O_3_ | 26.83 | 330.1441 | 330.1442 | 329.1371, [M-H]¯ |
| 29 | C_25_H_32_N_5_O_2_P_4_ | 27.50 | 558.1490 | 558.1508 | 559.1573, [M+H]⁺ |
| 30 | C_31_H_48_F_8_O_6_P_2_Si | 27.66 | 758.2576 | 758.2575 | 757.2505, [M-H]¯ |
| 31 | C_26_H_36_F_8_N_14_P_2_ | 29.72 | 758.2575 | 758.2553 | 757.2506, [M-H]¯ |
| 32 | C_27_H_27_F_3_N_13_O_4_ | 29.98 | 654.2265 | 654.2259 | 653.2186, [M-H]¯ |
| 33 | C_30_H_43_F_6_N_5_OP_3_ | 32.40 | 696.2591 | 696.2587 | 695.2524, [M-H]¯ |
| 34 | C_48_H_58_F_6_O_6_P_2_ | 32.40 | 906.3619 | 906.3616 | 905.3540, [M-H]¯ |
| 35 | C_29_H_28_N_9_O_4_P | 32.40 | 597.2020 | 597.2017 | 596.1954, [M-H]¯ |
| 36 | C_57_H_64_F_9_O_7_PSi | 32.41 | 1090.4031 | 1090.4019 | 1091.4096, [M+H]⁺ |
| 37 | C_22_H_25_F_9_N_16_O_9_Si_2_ | 33.45 | 884.1380 | 884.1377 | 885.1457, [M+H]⁺ |
| 38 | C_12_H_27_N | 35.17 | 185.2144 | 185.2144 | 186.2217, [M+H]⁺ |
| 39 | C_19_H_23_F_3_O_4_ | 35.30 | 372.1557 | 372.1549 | 371.1488, [M-H]¯ |
| 40 | C_30_H_37_FN_9_O_4_P_2_ | 36.59 | 668.2419 | 668.2402 | 667.2355, [M-H]¯ |
| 41 | C_25_H_15_N_10_OP_3_ | 36.68 | 564.0637 | 564.0635 | 565.0709, [M+H]⁺ |
| 42 | C_14_H_28_N_3_O | 37.17 | 254.2236 | 254.2228 | 253.2168, [M-H]¯ |
| 43 | C_31_H_45_F_2_N_4_O_7_P_2_ | 37.49 | 685.2733 | 685.2727 | 686.2804, [M+H]⁺ |
| 44 | C_32_H_46_F_5_N_9_OP | 44.00 | 698.3485 | 698.3463 | 699.3557, [M+H]⁺ |
| 45 | C_26_H_50_NO_7_P | 45.58 | 519.3323 | 519.3324 | 520.3397, [M+H]⁺ |
| 46 | C_29_H_51_F_3_N_4_P_4_ | 50.49 | 636.2985 | 636.2975 | 637.3055, [M+H]⁺ |
| 47 | C_27_H_42_F_2_NO_2_ | 51.32 | 450.3172 | 450.3164 | 449.3120, [M-H]¯ |
| 48 | C_30_H_48_F_2_NO_2_ | 51.40 | 492.3642 | 492.3619 | 491.3560, [M-H]¯ |
