# Supplementary material for: Metabolomics analyses of traditional Chinese medicine formula Shuang Huang Lian by UHPLC-QTOF-MS/MS
Source: Chin Med. 2022 May 30;17:62. doi: 10.1186/s13020-022-00610-x (PMC9150355; doi:10.1186/s13020-022-00610-x)
Supplement: Supplementary file 12 — Additional file 12: Table S9. The common components found in all three SHL preparation forms with VIP scores > 1.00. [file 13020_2022_610_MOESM12_ESM.docx]

**Table S9. The common components found in all three SHL preparation forms with VIP scores > 1.00**

| **No.** | **Compound** | **ESI mode** | **VIP score** |
| --- | --- | --- | --- |
| 1 | C_29_H_51_F_3_N_4_P_4_ | + | 1.0331 |
| 2 | C_25_H_33_F_12_P | + | 1.0546 |
| 3 | C_15_H_14_F_6_NO | - | 1.0340 |
| 4 | C_25_H_30_N_12_O_4_P_2_ | - | 1.0401 |
| 5 | C_30_H_37_FN_9_O_4_P_2_ | - | 1.0687 |
| 6 | Hispidulin 7-glucuronide | + | 1.0922 |
| 7 | C_35_H_42_N_15_O_7_P_2_ | - | 1.1020 |
| 8 | C_14_H_24_F_4_OP_2_ | - | 1.1125 |
| 9 | C_20_H_22_N_2_O_6_P_2_ | - | 1.1156 |
| 10 | 5,3'-Dihydroxy-7,4'-dimethoxy-4-phenylcoumarin | + | 1.1253 |
| 11 | C_31_H_45_F_2_N_4_O_7_P_2_ | + | 1.1483 |
| 12 | C_26_H_32_FNO_7_P | - | 1.1504 |
| 13 | C_27_H_27_F_3_N_13_O_4_ | - | 1.1578 |
| 14 | Danielone | + | 1.1612 |
| 15 | Rutin | + | 1.1776 |
| 16 | Methyl caffeate | + | 1.1851 |
| 17 | Piperonal | + | 1.1863 |
| 18 | Apigenin 7-(3'',4''-diacetylglucoside) | + | 1.2207 |
| 19 | C_52_H_58_F_11_O_7_PSi | - | 1.2496 |
| 20 | C_30_H_48_F_2_NO_2_ | - | 1.2897 |
| 21 | C_26_H_36_F_8_N_14_P_2_ | - | 1.2929 |
| 22 | Forsythin | + | 1.3154 |
| 23 | C_25_H_15_N_10_OP_3_ | + | 1.3235 |
| 24 | C_28_H_57_F_3_NO_3_P_8_Si | - | 1.3377 |
| 25 | Tagetiin | - | 1.3684 |
| 26 | C_25_H_32_N_5_O_2_P_4_ | + | 1.3785 |
| 27 | Luteolin-7-O-glucoside | + | 1.3946 |
| 28 | C_27_H_45_P_5_ | - | 1.3948 |
| 29 | Plumieride | + | 1.3970 |
| 30 | C_24_H_32_F_5_N_5_O_4_P | - | 1.3990 |
| 31 | 1-Monopalmitin | + | 1.4039 |
| 32 | C_12_H_27_N | + | 1.4057 |
| 33 | Tarennoside | + | 1.4142 |
| 34 | Isofurcatain 7-O-glucoside | + | 1.4161 |
| 35 | Paeonilactone B | + | 1.4188 |
| 36 | C_17_H_21_F_3_O_3_ | - | 1.4276 |
| 37 | Arg-Glu-Glu | - | 1.4277 |
| 38 | Asp-Arg-Pro | - | 1.4343 |
| 39 | Isovitexin | + | 1.4445 |
| 40 | Pro-Trp-Asp | + | 1.4511 |
| 41 | C_21_H_39_F_5_N_4_P_2_ | + | 1.4568 |
